# Merging Pyrrole with Boron into Versatile Di(2‐pyrryl)borane Building Blocks: π‐Extension, Polymerization, and Coordination

**DOI:** 10.1002/anie.202516982

**Published:** 2025-11-17

**Authors:** Daniel Göbel, Andreas Helbig, Alexandra Friedrich, Jonas Bachmann, Manuel Buckel, Johannes Chorbacher, Holger Helten

**Affiliations:** ^1^ Julius‐Maximilians‐Universität Würzburg Institute of Inorganic Chemistry and Institute for Sustainable Chemistry & Catalysis with Boron (ICB) Am Hubland 97074 Würzburg Germany

**Keywords:** Boron, Hybrid polymers, Organoboranes, Pyrrole, Twisted intramolecular charge transfer

## Abstract

Conjugated di(hetaryl)boranes—particularly those of thiophene, but also of furan—have recently proven to be extremely useful building blocks for organic optoelectronic materials. The importance of the combination of boron with pyrrole, on the other hand, becomes particularly apparent when considering the well‐known, versatile BODIPY dyes, in which the boron atom is tetracoordinated. However, synergistic effects of combining pyrrole with tricoordinate boron have been hardly exploited to date, which is probably due to synthetic challenges associated with the special reactivity of this five‐membered heterocycle. Herein, a high‐yield, gram‐scale synthesis of two di(2‐pyrryl)boranes is presented, including the previously elusive unprotected *N*H derivative. Their versatility is demonstrated by their application as building blocks for π‐extended tetrahetarene boranes, a polymer, and a zirconium(IV) complex. The crystallographically determined molecular structures of both di(2‐pyrryl)boranes and the Zr complex show largely planar dipyrrylborane moieties. Compared to their thiophene and furan congeners, the new conjugated boranes show bathochromically shifted absorption bands and more intense fluorescence emission, with solvatochromic behavior, aggregation‐induced emission enhancement (AIEE), and solid‐state emission. Time‐dependent DFT calculations reveal that this is due to the population of twisted intramolecular charge transfer (TICT) states, giving rise to dual emission in the unprotected triarylborane.

## Graphical abstract

The synthesis of di(2‐pyrryl)boranes, including the previously elusive unprotected *N*–H derivative, has been achieved that serve as versatile building blocks for π‐extended tetrahetarene boranes, a poly(dipyrrole borane), and a zirconium(IV) complex. The new π‐conjugated boranes are efficient blue‐light emitters, due to a twisted intramolecular charge transfer (TICT) character of the excited state. Aggregation causes emission enhancement, resulting from the formation of highly emissive nanoaggregates.

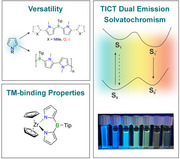

## Introduction

**Figure 1:**
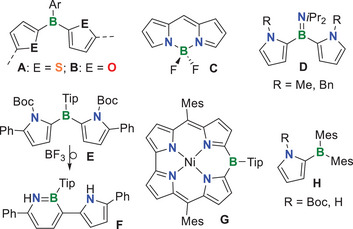
Di(2‐thienyl)borane (A) and di(2‐furyl)borane building blocks (B) (Ar denotes a bulky aryl group) and known compounds wherein boron is merged with pyrrole rings (**C–H**) (Bn = benzyl; Boc = *tert*‐butyloxycarbonyl; Mes = mesityl; Tip = 2,4,6‐triisopropylphenyl).

Recent years have witnessed the advent of organoborane‐based functional materials in which trivalent boron is integrated into an extended conjugated π‐system. Their uses span an enormous range from organic electronics to sensors and theranostics, to name just a few examples.^[^ ^1^, ^2^, ^3^, ^4^, ^5^, ^6^, ^7^, ^8^ ^]^ The combination of an electron‐deficient tricoordinate boron center with electron‐rich aromatic heterocycles has proven particularly advantageous due to pronounced p–π* interactions between the boron's vacant p‐orbital and unoccupied π‐orbitals of the conjugated system, resulting in significantly decreased LUMO energy levels.^[^ ^9^, ^10^ ^]^ Jäkle and colleagues have established di(2‐thienyl)boranes (**A**, Figure 1) as versatile building blocks for various molecular and polymeric hybrid materials with intriguing properties and functions.^[^ ^11^, ^12^, ^13^, ^14^, ^15^, ^16^, ^17^, ^18^, ^19^, ^20^, ^21^, ^22^, ^23^, ^24^, ^25^, ^26^, ^27^, ^28^, ^29^, ^30^, ^31^, ^32^, ^33^ ^]^ Recent developments include, for example, sulfur‐bridged dithienylboranes as polymer building blocks, as reported by Adachi and Ohshita.^[^ ^34^ ^]^ A bulky aryl group (Ar) as the third boron‐substituent, typically mesityl (Mes), 2,4,6‐triisopropylphenyl (Tip), 2,4,6‐tri‐*tert*‐butylphenyl (Mes*), or 2,4,6‐tris(trifluoromethyl)phenyl (^F^Mes), provides kinetic stabilization and makes the compounds air‐ and water‐stable. Our group has developed an environmentally benign organocatalytic Si/B exchange condensation procedure for the synthesis of thienylborane polymers and oligomers.^[^ ^35^, ^36^, ^37^ ^]^ Recently, we presented novel porphyrinoid macrocycles with redox‐switchable aromaticity that are comprised of the structural unit **A**.^[^ ^38^ ^]^ We also introduced di(2‐furyl)borane (**B**) as a further building block for extended π‐conjugated compounds with favorable features, particularly, improved fluorescence emission efficiency, probably due to the exclusion of heavy‐atom effects.^[^ ^35^, ^36^, ^37^, ^39^, ^40^, ^41^, ^42^ ^]^

In contrast, comparable chemistry using the combination of boron with pyrrole is largely unexplored, probably due to synthetic challenges. This is partly due to the reactive *N*H group if it is unprotected. At *N*‐methylpyrrole, Braunschweig and co‐workers observed a spontaneous migration of the dichloroboryl group from the 2‐ to the 3‐position.^[^ ^43^ ^]^ Pyrrole shares some common features with thiophene and furan. It is likewise electron‐rich and aromatic; its aromatic character has been ranked intermediate between that of the other two heteroarenes. However, the *N*H group offers some additional opportunities such as H‐bonding and special coordination capabilities. When pyrrole groups are connected in pairs via a methylene or an *N*H group, they can also exist in a dehydrated form known as (aza)dipyrrin. This, along with the possibility of *N*–H deprotonation, makes various pyrrole‐based scaffolds excellent ligands for numerous transition metals as well as main‐group elements (including boron).^[^ ^44^, ^45^, ^46^, ^47^, ^48^, ^49^ ^]^ This is evident, for example, in the substance class of porphyrins, which are found in nature and have been used as functional materials.^[^ ^50^, ^51^, ^52^, ^53^, ^54^, ^55^, ^56^, ^57^, ^58^ ^]^

The synergy of pyrrole and boron is best reflected in the significance of BODIPY dyes (**C**), which have an enormous scope of applications that range from laser dyes to bioimaging and the generation of singlet oxygen.^[^ ^59^, ^60^, ^61^, ^62^, ^63^, ^64^, ^65^, ^66^, ^67^ ^]^ The boron center in **C** is tetrahedrally coordinated, involving chelation by the dipyrromethene ligand, thus ensuring pronounced stability.^[^ ^59^, ^60^, ^61^, ^62^, ^63^, ^64^, ^65^ ^]^ Regarding the merging of tricoordinate boron with pyrrole moieties, some examples have been reported where a boryl group (typically dimesitylboryl) has been placed in the periphery of a porphyrin or a BODIPY, having a major influence on the compound's photophysical properties.^[^ ^68^, ^69^, ^70^, ^71^, ^72^, ^73^, ^74^, ^75^, ^76^, ^77^, ^78^, ^79^, ^80^ ^]^ A few di(2‐pyrryl)boranes were prepared and briefly investigated by NMR spectroscopy only,^[^ ^81^, ^82^ ^]^ while Siebert and coworkers characterized the amino‐substituted derivatives **D** more extensively.^[^ ^83^ ^]^ In the latter, the boron center is stabilized by π‐donation from the lone electron pair of nitrogen, although this does not render the compounds air‐ or water‐stable. In addition, it largely diminishes the potential interaction of the pyrryl groups with the boron center. No photophysical properties have thus been reported for these compounds.^[^ ^83^ ^]^ The pyrrolic nitrogen centers of each di(2‐pyrryl)borane reported so far^[^ ^81^, ^82^, ^83^ ^]^ are blocked, and deprotection without decomposition has not yet been achieved. Yamaguchi and coworker reported the triarylborane **E** featuring two *N*‐Boc‐protected 2‐pyrryl groups on boron. Their 5,5′‐positions are additionally substituted with flanking phenyl groups, and the boron center is kinetically stabilized by Tip. In an attempt to remove the N‐protecting Boc groups, insertion of the boron atom into one pyrrole ring occurred instead, thus yielding 1,2‐azaborinine derivative **F**.^[^ ^84^ ^]^ So, N‐unprotected di(2‐pyrryl)boranes still remain elusive.

Shinokubo and coworkers reported on the synthesis of Ni(II) 10‐boracorrole **G** by transmetalation of the corresponding Ni(II)‐coordinated 10‐silacorrole. The trivalent boron atom showed a strong effect on the absorption properties of this exciting antiaromatic macrocycle.^[^ ^85^ ^]^ Thilagar and coworkers succeeded in isolating mono(2‐pyrryl)boranes **H**, including the N–H derivative, which showed intense charge transfer and solid‐state emission due to intermolecular *N*–H···π interactions. Steric hindrance by the two bulky mesityl groups, however, prevented further transformations involving the nitrogen center.^[^ ^86^ ^]^

In this study, we report the successful synthesis of two di(2‐pyrryl)boranes, including the previously elusive unprotected *N*–H derivative **4**, and transformation thereof into π‐extended compounds **7a‐c** and a polymer **8**. These compounds are intense blue‐light emitters. Their special photophysical behavior originates from the TICT character of their excited state. The free *N*H group of **4** is further available for transition metal coordination, which we demonstrated by the synthesis of zirconium(IV) complex **9**.

## Results and Discussion

**Scheme 1:**
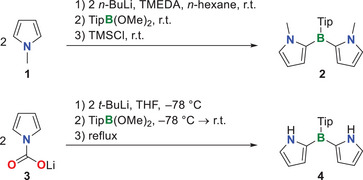
Synthesis of di(2‐pyrryl)boranes **2** and **4**.

We synthesized di(1‐methyl‐2‐pyrryl)borane **2** via lithiation of *N‐*methylpyrrole with *n*‐BuLi in the presence of TMEDA and subsequent addition of 0.55 equiv of TipB(OMe)_2_ (Scheme 1). After quenching with TMSCl to cleave residual borates formed, **2** was purified by column chromatography and isolated in 59% yield. For the synthesis of the *N*–H derivative **4**, we decided to use lithium carboxylate as the protecting group on the pyrrole nitrogen. This group should be easily removable after borylation; additionally, it aids in lithiation at the 2‐position through lithium coordination by the oxygen donor centers.^[^ ^87^ ^]^ Reacting **3** with *t*‐BuLi in THF at −78 °C, addition of 0.5 equiv of TipB(OMe)_2_, and subsequent heating at reflux, directly afforded deprotected **4**, which was isolated in 47% yield. The ^1^H NMR spectrum of the product in C_6_D_6_ showed a broad resonance at 8.04 ppm assigned to the *N*–H group, showing that the protecting group had been removed during the refluxing. The ^11^B{^1^H} NMR spectra of **2** and **4** showed a broad signal at 51.8 and 47.9 ppm, respectively, which is in the typical region for dihetarene boranes.^[^ ^35^, ^36^, ^37^ ^]^

**Scheme 2:**
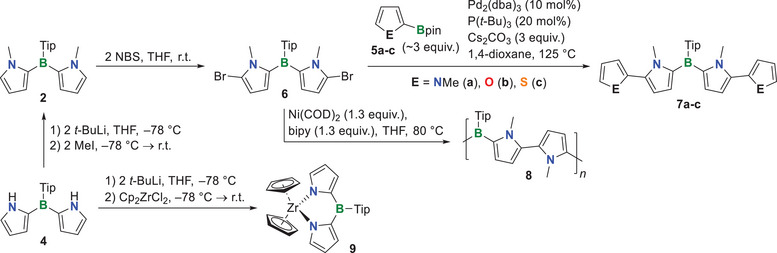
Synthesis of π‐extended boranes **7a‐c**, polymer **8**, and Zr complex **9**.

Next, we used the dipyrrylboranes **2** and **4** as building blocks for more complex architectures. To this end, we functionalized **2** at the 5,5′‐positions of its pyrryl groups to give the dibrominated species **6** (Scheme 2), of which we were able to obtain single crystals suitable for X‐ray crystallography (see Figure S3). This compound was subsequently subjected to Suzuki–Miyaura cross‐coupling with the borylated five‐membered heteroaromatics **5a‐c** to give the π‐extended boranes **7a‐c**. The tetrapyrrole derivative **7a** and compounds **7b‐c** were purified by column chromatography or flash column chromatography, respectively, and isolated in 17%–28% yield; losses of yield mainly occur during the purification process. The ^11^B{^1^H} NMR spectra of **7a‐c** showed a broad signal at about 50 ppm, which is in the same region as the starting material **6** and typical for related literature‐known aryldihetarene boranes.^[^ ^35^, ^36^, ^37^ ^]^

We also subjected the dibromide **6** to a Yamamoto‐type polymerization. To this end, 2,2′‐bipyridine and bis(cyclooctadiene)nickel(0) were added to **6** in THF and stirred for 3 days at 80 °C. After precipitation of the product into cold methanol (−78 °C), we obtained the polymer **8** in 48% yield. Gel permeation chromatography (GPC) suggested a number average molecular weight *M*_n_ = 5.51 kDa and a weight average molecular weight *M*_w_ = 9.85 kDa, corresponding to a polydispersity index (PDI) of 1.79. The ^1^H NMR spectrum of **8** showed all expected signals, which could be unambiguously assigned by comparison with the corresponding signals of compound **7a**.

Then, we investigated the reactivity of the *N*–H proton of compound **4** to explore its potential for further transformations via the nitrogen center. To find the best reaction conditions for *N*‐lithiation, follow‐up reactions to give the *N*‐methyl derivative **2** were performed (Scheme 2). We found that the best conditions encompass lithiation with *t*‐BuLi in THF at −78 °C for 1.5 h, followed by the addition of methyl iodide. With the optimized lithiation procedure in hand, we reacted dilithiated **4** with Cp_2_ZrCl_2_ at −78 °C to give zirconium complex **9**, which was purified by sublimation.

**Figure 2:**
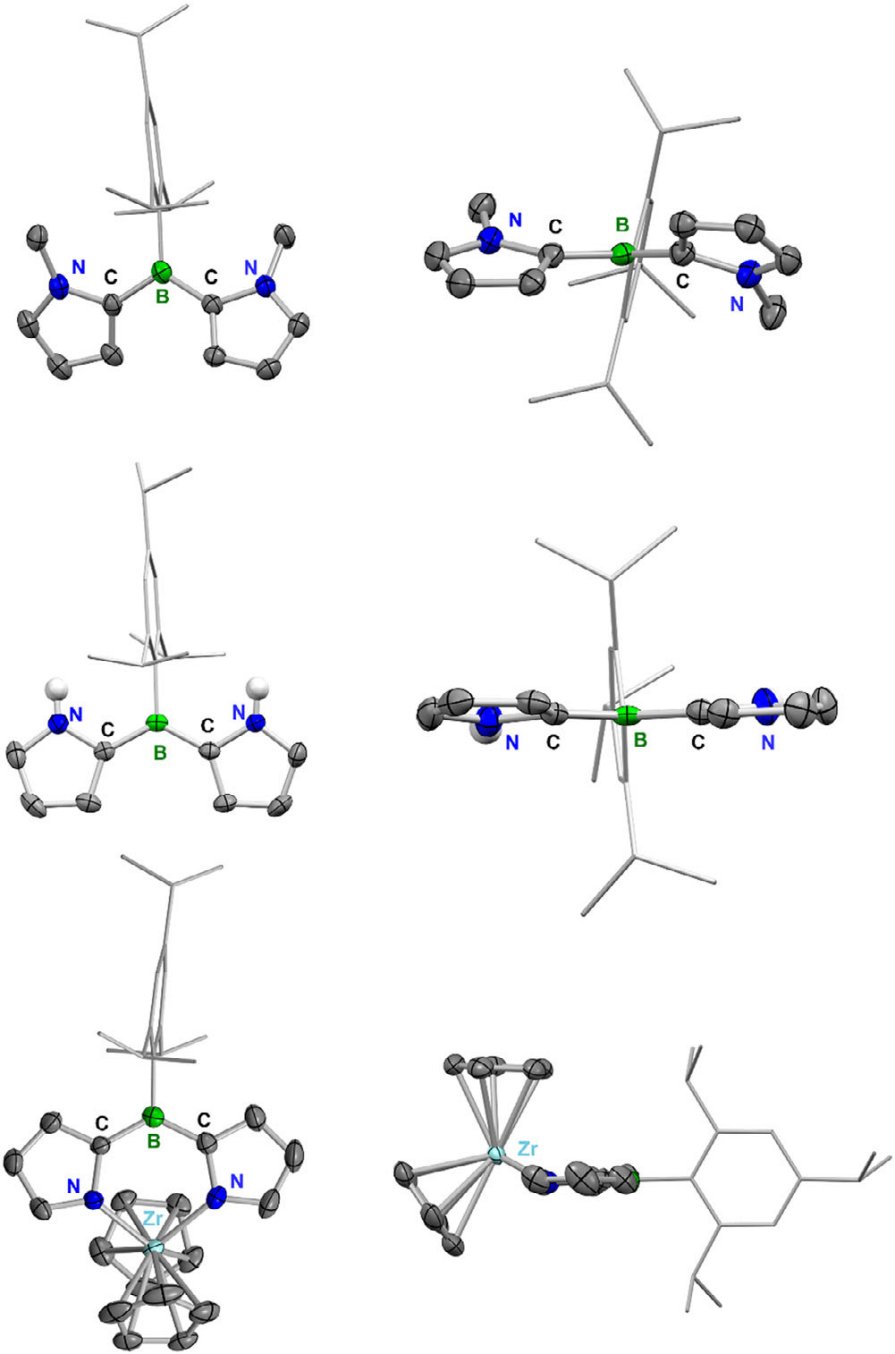
Molecular structures of **2** (top), **4** (middle), shown from above (left) and along the B–C_Tip_ axis (right), and **9** (bottom), shown from above (left) and side on (right) in the solid state from single‐crystal X‐ray diffraction at 100 K. All ellipsoids are drawn at the 50% probability level. C‐bonded H atoms are omitted, and the ellipsoids of the Tip substituents are omitted and depicted as capped sticks for clarity.

The structures of **2**, **4**, and **9** in the solid state were additionally determined by single‐crystal X‐ray diffraction (Figure 2 and Table S2). They feature a largely planar system involving the two pyrryl groups and the trigonal‐planar BC_3_ moiety, whereas the Tip group is almost perpendicular to that plane, like in the thiophene and furan analogues of these compounds (see Table S2). Interestingly, different from the latter two, the pyrryl rings of **2** and **4** show a *syn*‐arrangement, with both *N*–R groups (R = H or Me) pointing toward the Tip substituent. For compound **2**, the twist angles between the pyrrole units and the BC_3_ plane are 22.81(8)° and 21.93(8)°, respectively. In the *N*–H derivative **4**, the coplanarity is even more pronounced, with torsion angles of only 4.82(7)° and 7.05(8)°, indicating improved p‐π‐conjugation compared to *N*‐methyl derivative **2**. The shorter B─C bonds of **4** further support this interaction (see Table S2). Compared to 2‐pyrryl‐BMes_2_ (**F**), reported by Thilagar,^[^ ^86^ ^]^ the B─C bonds of **4** are slightly longer. The solid‐state structure furthermore shows relatively short *N*–H···π distances (*N*–H···centroid: 3.272 and 3.452 Å; and *N*–H···C*_ipso_* (Tip): 2.649 and 2.731 Å). According to our DFT calculations (B3‐LYP‐D3(BJ)/def2‐SV(P)), the *syn*‐conformer of **4** should also be favored in the gas phase. It is predicted to be more stable than the *anti*‐conformer by 3.7 kJ mol^−1^, and with respect to the conformer with both *N*–H groups pointing away from the Tip group, it is computed to be more stable by even 17.3 kJ mol^−1^.

In contrast to **2** and **4**, in the solid‐state molecular structure of complex **9**, both pyrrole rings face away from the Tip moiety, and the two nitrogen atoms bind to the zirconium atom in a chelating way. The coplanarity of the pyrrole rings of **9** (∠(hetaryl‐BC_3_): 3.02°/2.78°) is even more pronounced than in its precursor **4**, due to the formed six‐membered ring via the metallacyclic substructure. The Tip moiety is slightly less orthogonal to the dipyrrylborane plane compared to the unsubstituted triarylborane **4**. The torsion angles of the Tip plane and the adjacent pyrrole planes in **9** are 78.37/79.87°, which is 7–10° smaller compared to compound **4** (∠(hetaryl‐Tip): 88.54(7)°/85.46(7)°) and can be explained by lower steric repulsion. Each Cp ring coordinates η^5^, with Zr–C distances ranging from 2.489 to 2.552 Å, which is in the range of comparable reported distances of 2.49(2)–2.59(2) Å observed in Cp_2_Zr(pyrryl)_2_ and Cp_2_Zr(2,5‐Me_2_pyrryl)_2_.^[^ ^88^, ^89^ ^]^ The Zr–N distances in **9** (2.157(1) and 2.164(1) Å) are comparable to the distances observed for representative η^1^‐N‐pyrryl and η^1^‐N‐indolyl complexes of zirconium (2.07–2.33 Å).^[^ ^90^ ^]^

**Figure 3:**
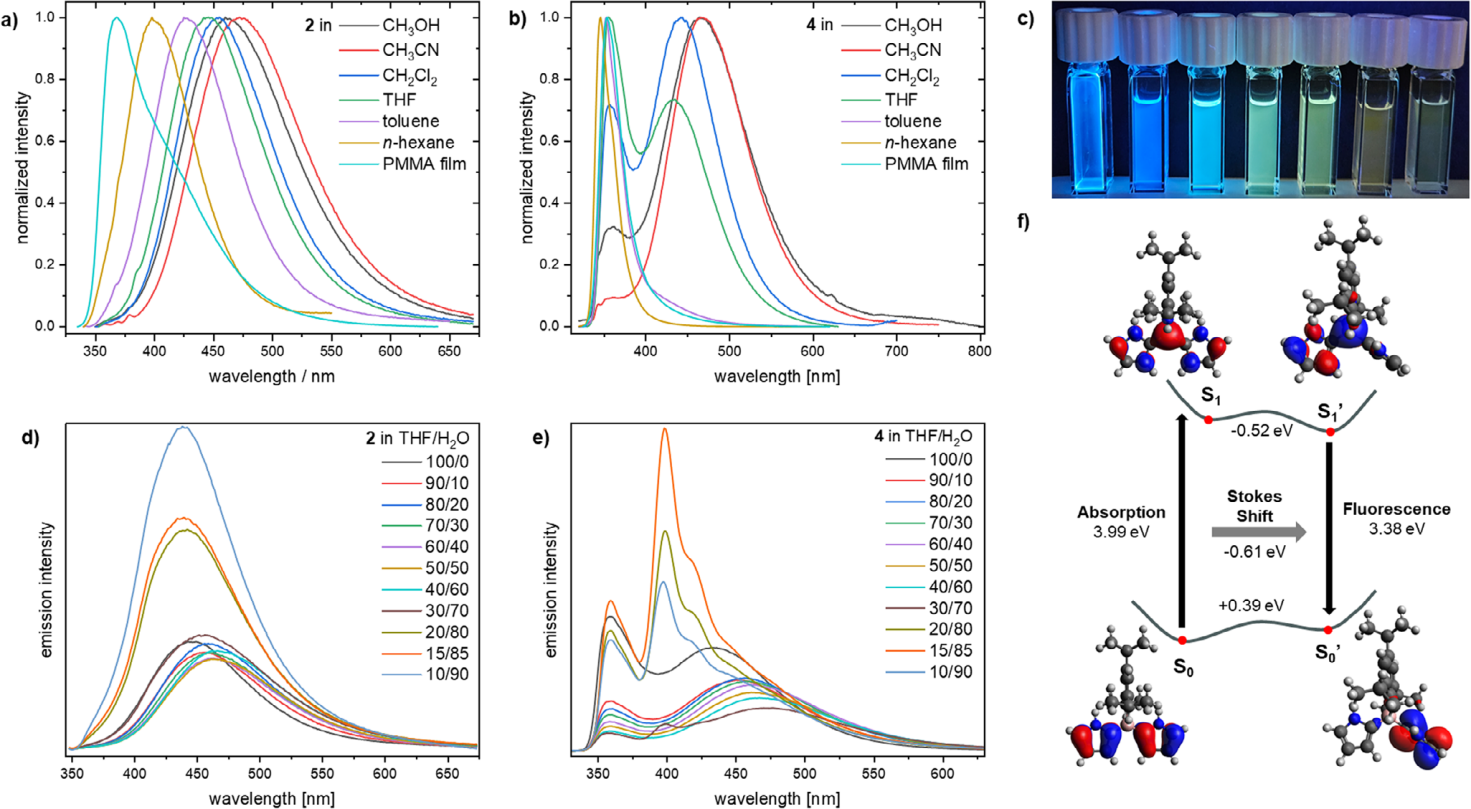
Emission spectra in different solvents a) of **2**, b) of **4**, and c) cuvettes of **7a** in different solvents under UV‐irradiation (*λ* = 365 nm), from PMMA‐film (left) to methanol (right). d) Emission spectra in THF/water mixtures (conc. 5 ∙ 10^−5^ M) with different water fractions (0–90%) of **2**, and e) **4**. f) Graphical representation for the TICT emission of **4** with optimized S_0_ and S_1_’ structures at the B3‐LYP/6–31 + G* level of theory and their orbital contributions at an isovalue of 0.035.

The UV/vis spectra of **2**, **4**, and **9** in THF show a low‐energy absorption band at about 340 nm, which is red‐shifted compared to the corresponding band of the furan and thiophene analogues (λ_abs,max_ ≈ 325 nm; Table 1).^[^ ^35^, ^36^, ^37^ ^]^ Our TD‐DFT calculations revealed that this band is assigned to a π–π*‐transition within the dipyrrylborane system, occurring from the HOMO to the LUMO in both cases. While the HOMO is delocalized over both pyrrole rings and has a nodal plane at the boron position, the boron atom has the largest contribution to the LUMO (see Figure 3f for the orbital representations of **4**). Interestingly, the pyrrole compounds show significant blue fluorescence, whereas the other dihetarylboranes showed weak to no emission in solution (Table 1). The emission maximum of **2** in THF shows a large Stokes shift of 104 nm (96 153.85 cm^−1^). For **4**, two emission maxima were observed, a broad one in the same region as for **2** and a narrower one at higher energy (357 nm; Table 1). The quantum yields (Φ_F_) in THF were higher for **2** (Φ_F_ = 14%) and **4** (Φ_F_ = 29%) than for the corresponding furan derivative. This experimental data is well reproduced by TD‐DFT calculations (B3‐LYP/6–31 + G*). The orange‐colored complex **9** is non‐emissive.^[^ ^91^, ^92^, ^93^ ^]^

**Table 1:** Experimental and calculated photophysical data^a)^ for **2** and **4** in comparison to the thiophene (TipBThi_2_) and furan (TipBFur_2_) analogues,^[^ ^35^, ^36^, ^37^ ^]^ and **7a‐c** in comparison to its tetrafuryl‐ and difuryldithienylborane analogues.^[^ ^39^ ^]^

Compound	*λ*_abs,max_ [nm]	*λ*_calc,ex_ [nm]	*λ*_em,max_ [nm]	Φ_F_ [%]	τ [ns]
**2**	342	322	446	14	4.06
**4**	336	311	357, 434	26	0.50, 7.47
TipBThi_2_^[^ ^35^, ^36^, ^37^ ^]^	325	316	410	3	–
TipBFur_2_^[^ ^35^, ^36^, ^37^ ^]^	324	305	–	–	–
**7a**	374	389	511	46	4.12, 9.01
**7b**	386	369	503	34	3.82, 9.18
**7c**	388	344	495	42	1.67, 4.03
Mes*B(FurThi)_2_^[^ ^39^ ^]^	400	418	433	87	–
Mes*B(Fur_2_)_2_^[^ ^39^ ^]^	394	411	427	67	–

^a)^ Measurements in THF solution. Calculated data obtained with B3‐LYP‐D3(BJ)/def2‐SV(P), and for the thiophene and furan compounds, mesityl substituents have been used for computational convenience.^[^ ^35^, ^36^, ^37^ ^]^ Excited‐state calculations were performed on the B3‐LYP/6–31 + G* level of theory.

The UV/vis spectra of **7a‐c** show an absorption band with a maximum at 374, 386, and 388 nm (Table 1), respectively, which, according to our TD‐DFT calculations, is assigned to a π–π* transition involving the HOMO and LUMO. While the former extends over the four hetarene rings, the LUMO shows the largest contribution from the boron center (see Figures S143–S145). Tetrapyrrylborane **7a** shows a 32 nm‐redshift of this specific band in comparison to that of **2**, confirming an extended π‐conjugated system over the appended pyrrole rings. Compared to its tetrafuryl‐ and difuryldithienylborane analogues, which were reported by us previously,^[^ ^39^ ^]^ the absorption band of **7a** is at a shorter wavelength. This is presumably ascribed to the fact that the coplanarity of the pyrrole rings in **7a** (twist angles in the calculated structure: 44.9–48.1°) is less pronounced than that of the furan and thiophene rings of the reference compounds. This, in turn, can be attributed to steric congestion imparted by the methyl groups on nitrogen. The absorption bands of compounds **7b‐c** are between those of tetrapyrrylborane **7a**, Mes*B(Fur_2_)_2_, and Mes*B(FurThi)_2_, suggesting a higher coplanarity of the hetaryl rings in **7b‐c** than in its pure pyrryl analogue. Interestingly, out of the tetrahetarene boranes compared in Table 1, compound **7a** shows the most bathochromically shifted emission band (511 nm versus ca. 500 nm for **7b‐c** versus ca. 430 nm for the furyl‐ and thienylborane derivatives).

The emission properties of all compounds were investigated in different solvents and in homogeneous PMMA films. The spectra showed bathochromic shifts with increasing polarity of the solvent. For compound **4**, this covered a spectral range of about 120 nm between the PMMA matrix and acetonitrile. Moreover, the dual emission of **4** is most prominent in THF and DCM, whereas in nonpolar solvents only the narrow higher‐energy emission band occurs. In a more polar environment, the broad, bathochromically shifted emission band is enhanced (Figure 3b). This suggests a more polar nature in the excited state. The dual emission of **4** in THF and DCM indicates two competing emission pathways. Therefore, we optimized the excited state of **4** by TD‐DFT. This predicted an emission from the relaxed S_1_’ state at 367 nm. In the optimized structure, one pyrrole unit is twisted by about 90° out of the conjugated plane, suggesting *twisted intramolecular charge transfer* (TICT) emission, with the orbital contributions shown in Figure 3f. Due to the strongly polar nature of S_1_’, this emission pathway becomes dominant in polar solvents, resulting in a large Stokes shift, while in less polar solvents, a direct emission from the S_1_ state is observed. The dual emission in THF and DCM can be explained by considering the lifetimes τ of **4** in THF, which are 0.50 and 7.47 ns, respectively (Table 1). As the first lifetime is very short compared to the second one, it is reasonable to assume that over the time scale of the fluorescence measurements, emissions from both states (LE band and TICT band) are detected, resulting in the emergence of both bands in these solvents. Lifetime measurements of **4** in *n*‐hexane and methanol resulted only in one value, reflected by a single band in the fluorescence spectra in these solvents, confirming that therein only one excited‐state structure is dominant.

Compound **2** showed a partly similar behavior. Its fluorescence spectrum underwent a bathochromic shift of 100 nm when increasing the solvent polarity, resulting from enhancement of the TICT emission compared to the LE band in polar solvents (Figure 3a). The quantum yields in the PMMA film range from 19% for **2** to 70% for **4**. In solution, the highest quantum yields were observed in *n*‐hexane (Φ_F_ = 21% for **2** and 38% for **4**; see Table S3). Also, the effect of the substituent at the nitrogen atom becomes apparent. In compound **2**, the influence of the solvent polarity is comparatively low, which is probably ascribed to a shielding effect from the methyl group. In the case of **4**, especially polar solvents can stabilize the twisted excited state structure particularly well. Thilagar and coworkers observed an analogous effect for the *N*‐unprotected pyrrylborane **H** with protic solvents, due to the polar nature of the excited state.^[^ ^86^ ^]^

The observation that compounds **2** and **4** show their highest quantum yields in the PMMA matrix prompted us to investigate their photophysical characteristics upon aggregation in solution. To this end, we prepared solutions of **2** and **4** in THF and successively increased the content of admixed water from 0% to 90% (Figure 3d,e). For compound **2**, the fluorescence intensity decreased slightly until a water content of 50% was reached. At higher water content, an increasingly intense luminescence was observed up to a 90% H_2_O ratio, which is threefold higher than that in pure THF. Furthermore, the spectra of **2** undergo a slight bathochromic shift as the water content is increased from 0% to 60% by 21 nm (from 445 to 466 nm), followed by a hypsochromic shift with higher percentages of water by 25 nm (to 439 nm; see Table S4). This can be explained by two opposing effects. The enhanced polarity of the solvent environment with increasing water content leads to a positive solvatochromic effect in donor–acceptor species that form charge‐separated excited states, as the ones that are subject of this study. On the other hand, hypsochromic effects can be attributed to the physical restriction of the intramolecular vibrations due to the increase in the hydrophobicity of the local environment of the molecule.^[^ ^94^, ^95^ ^]^ The observation of this aggregation‐induced emission enhancement (AIEE) effect indicates the formation of strongly emissive nanoaggregates. To confirm this hypothesis, we additionally performed dynamic light scattering (DLS) measurements in THF/water (10/90) mixtures for **2**. This confirmed the formation of particles of **2** with an average hydrodynamic radius (*R*_h_) of 64.5 nm (see Figure S101).

The *N*–H derivative **4** shows a similar behavior in that the emission intensity decreased until a water content of 60% was reached. Then, the fluorescence intensity increased again while forming a new, well‐resolved emission band at 398 nm (Figure 3e). DLS measurements confirmed the formation of particles with a hydrodynamic radius of 45.4 nm (see Figure S101). Additionally, only above 80% water content significant aggregation occurs, which agrees with the observed emission spectra, where the vibrational progression is dominant for THF/water mixtures between 20/80 and 10/90 (see Figure S102).

Like compounds **2** and **4**, the π‐extended derivatives **7a‐c** show strong solvent dependency in the emission. Of these three compounds, **7a** exhibits the strongest shift, ranging from 446 nm in *n*‐hexane to 565 nm in acetonitrile (see Figure S66). The emission maxima of **7b‐c**, however, range from 429 (**7b**) and 433 nm (**7c**) in *n*‐hexane to 546 nm (**7b‐c**) in acetonitrile (see Figures S67–S82). Compound **7c** shows the highest quantum yields out of the tetrahetarene boranes, ranging from 7% in methanol to 42% in THF, up to 84% in *n*‐hexane. Tetrapyrrylborane **7a**, for instance, exhibits quantum yields of 4% in methanol, 46% in THF, and 60% in *n*‐hexane, which are considerably higher than those of triarylborane **2** due to the larger conjugated π‐system of the tetrapyrrylborane (see Table S3). Excited‐state calculations on compounds **7a‐c** suggest a twisting of one of the dihetarene groups out of the conjugated π‐plane, in the case of the bipyrryl groups of **7a** by 86° in the relaxed S_1_’ state (see Figure S149).

Due to the TICT characteristic of the excited state, the emission of **7a** is shifted to significantly longer wavelength. The quantum yield of the emission of **7a** in THF, however, is lower than that for its tetrafuryl‐ and difuryldithienylborane congeners. In less polar solvents or in the PMMA film, the quantum yield increases significantly (see Table S3, for an overall comparison of quantum yields and lifetimes for **2**, **4**, and **7a‐c** in different solvents and PMMA film).

The poly(dipyrrole borane) **8** shows an absorption band at 415 nm, which is significantly bathochromically shifted compared to its monomer **2** or even compound **7a**, attributable to the larger conjugated π‐system. Its emission maximum at 487 nm, however, is hypsochromically shifted in comparison to **2** and **7a** (see Figure S83). This is probably due to the effect that the formation of the relaxed TICT state is significantly hindered for the long polymer chains.

Cyclic voltammetry (CV) of the triarylboranes **2**, **4**, **7a**, and **9** revealed a series of irreversible oxidation events. In addition, **2**, **7a**, and **9** each show a partially reversible reduction wave in THF. The halfwave potential of the irreversible reduction event of **4**, however, was determined by squarewave voltammetry. The reduction potentials follow the order: *E*_1/2_ = −3.17 V (**2**) ≈ −3.12 V (**4**) < −3.00 V (**7a**) < −2.13 V (**9**). CV of polymer **8** revealed three irreversible reduction events ranging from −2.30 to −2.75 V and two semi‐reversible oxidation events at 0.02 V and 0.33 V.

## Conclusion

We have introduced di(2‐pyrryl)boranes **2** and **4** as versatile building blocks for π‐extended molecular compounds, polymers, and coordination compounds. The latter is made possible by the first‐time synthesis of the previously elusive unprotected *N*–H derivative **4**. The molecular structures of both di(2‐pyrryl)boranes have been determined by single‐crystal X‐ray diffraction, thus revealing a largely planar structure of their dipyrrylborane moieties, which is particularly pronounced in the case of the *N*H compound **4**. This leads to interesting photophysical properties and stimuli‐responsive behavior. Their absorption and emission features are significantly bathochromically shifted compared to their previously known thiophene and furan congeners. The new compounds are intense blue‐light emitters, due to the TICT‐character of the excited state structure, as suggested by TD‐DFT calculations. Aggregation experiments revealed that the compounds show pronounced AIEE effects, and the formation of emissive nanoaggregates was verified by DLS measurements. We demonstrated the versatility of the new organoborane building blocks by the synthesis of π‐extended pyrrolylboranes **7a‐c**, the first example of a poly(dipyrrole borane) **8**, and the zirconium(IV) complex **9**. The tetrahetarene boranes **7a‐c** and the polymer **8** show the expected bathochromic shifts in their low‐energy absorption and emission bands due to their extended π‐conjugated systems. We are currently exploring the use of the newly developed building blocks as components for optoelectronic and stimuli‐responsive materials.

## Supporting Information

The authors have cited additional references within the Supporting Information.^[^ ^96^, ^97^, ^98^, ^99^, ^100^, ^101^, ^102^, ^103^, ^104^, ^105^, ^106^, ^107^, ^108^, ^109^, ^110^, ^111^, ^112^, ^113^, ^114^, ^115^ ^]^

## Conflict of Interests

The authors declare no conflict of interest.

## Supporting information



Supporting Information

Supporting Information

## Data Availability

The data that support the findings of this study are available in the Supporting Information of this article.

## References

[1] E. von Grotthuss , A. John , T. Kaese , M. Wagner , Asian J. Org. Chem. 2018, 7, 37–53, 10.1002/ajoc.201700495.

[2] M. Hirai , N. Tanaka , M. Sakai , S. Yamaguchi , Chem. Rev. 2019, 119, 8291–8331, 10.1021/acs.chemrev.8b00637. 30860363

[3] H. Helten , Chem. Asian J. 2019, 14, 919–935, 10.1002/asia.201900016. 30731024

[4] X. Yin , J. Liu , F. Jäkle , Chem. ‐ Eur. J. 2021, 27, 2973–2986, 10.1002/chem.202003481. 32852793

[5] H. Helten , in Comprehensive Organometallic Chemistry IV (Eds: D. O'Hare , K. Meyer , G. Parkin ), Elsevier, Amsterdam 2022, pp. 71–134;

[6] J. Miao , Y. Wang , J. Liu , L. Wang , Chem. Soc. Rev. 2022, 51, 153–187, 10.1039/D1CS00974E. 34851333

[7] J. Shi , Z. Ran , F. Peng , M. Chen , L. Li , L. Ji , W. Huang , J. Mater. Chem. C 2022, 10, 9165–9191, 10.1039/D2TC01243J.

[8] A. Borissov , Y. K. Maurya , L. Moshniaha , W.‐S. Wong , M. Żyła‐Karwowska , M. Stępień , Chem. Rev. 2022, 122, 565–788, 10.1021/acs.chemrev.1c00449. 34850633 PMC8759089

[9] Y. Ren , F. Jäkle , Dalton Trans. 2016, 45, 13996–14007, 10.1039/C6DT02756C. 27491626

[10] A. Dhiman , L. Giribabu , R. Trivedi , Chem. Rec. 2021, 21, 1738–1770, 10.1002/tcr.202100039. 33844422

[11] A. Sundararaman , M. Victor , R. Varughese , F. Jäkle , J. Am. Chem. Soc. 2005, 127, 13748–13749, 10.1021/ja0537880. 16201769

[12] X. Yin , J. Chen , R. A. Lalancette , T. B. Marder , F. Jäkle , Angew. Chem. Int. Ed. 2014, 53, 9761–9765, 10.1002/anie.201403700. 25044554

[13] X. Yin , F. Guo , R. A. Lalancette , F. Jäkle , Macromolecules 2016, 49, 537–546, 10.1021/acs.macromol.5b02446.

[14] X. Yin , K. Liu , Y. Ren , R. A. Lalancette , Y.‐L. Loo , F. Jäkle , Chem. Sci. 2017, 8, 5497–5505, 10.1039/C6SC03097A. 30155227 PMC6103004

[15] B. Meng , Y. Ren , J. Liu , F. Jäkle , L. Wang , Angew. Chem. Int. Ed. 2018, 57, 2183–2187, 10.1002/anie.201712598. 29314598

[16] Y. Adachi , Y. Ooyama , Y. Ren , X. Yin , F. Jäkle , J. Ohshita , Polym. Chem. 2018, 9, 291–299, 10.1039/C7PY01790A.

[17] Y. Yu , C. Dong , A. F. Alahmadi , B. Meng , J. Liu , F. Jäkle , L. Wang , J. Mater. Chem. C 2019, 7, 7427–7432, 10.1039/C9TC01562K.

[18] T. A. Welsh , A. Laventure , A. F. Alahmadi , G. Zhang , T. Baumgartner , Y. Zou , F. Jäkle , G. C. Welch , ACS Appl. Energy Mater. 2019, 2, 1229–1240, 10.1021/acsaem.8b01793.

[19] Y. Yu , B. Meng , F. Jäkle , J. Liu , L. Wang , Chem. ‐ Eur. J. 2020, 26, 873–880, 10.1002/chem.201904178. 31691387

[20] Y. Adachi , T. Nabeya , K. Kawakami , K. Yamaji , F. Jäkle , J. Ohshita , Chem. ‐ Eur. J. 2021, 27, 3306–3314, 10.1002/chem.202004643. 33314389

[21] A. F. Alahmadi , X. Yin , R. A. Lalancette , F. Jäkle , Chem. ‐ Eur. J. 2023, 29, e202203619, 10.1002/chem.202203619. 36562302

[22] A. Wakamiya , K. Mori , T. Araki , S. Yamaguchi , J. Am. Chem. Soc. 2009, 131, 10850–10851, 10.1021/ja905007s. 19618953

[23] A. Iida , S. Yamaguchi , J. Am. Chem. Soc. 2011, 133, 6952–6955, 10.1021/ja2019977. 21504199

[24] T. Araki , A. Wakamiya , K. Mori , S. Yamaguchi , Chem. Asian J. 2012, 7, 1594–1603, 10.1002/asia.201200055. 22473971

[25] L. G. Mercier , W. E. Piers , R. W. Harrington , W. Clegg , Organometallics 2013, 32, 6820–6826, 10.1021/om4004187.

[26] D. R. Levine , M. A. Siegler , J. D. Tovar , J. Am. Chem. Soc. 2014, 136, 7132–7139, 10.1021/ja502644e. 24738628

[27] Y. Cao , J. K. Nagle , M. O. Wolf , B. O. Patrick , J. Am. Chem. Soc. 2015, 137, 4888–4891, 10.1021/jacs.5b02078. 25860619

[28] Y. Yan , Z. Sun , C. Li , J. Zhang , L. Lv , X. Liu , X. Liu , Asian J. Org. Chem. 2017, 6, 496–502, 10.1002/ajoc.201700063.

[29] M. E. Cinar , T. Ozturk , Org. Commun. 2018, 11, 68–74, 10.25135/acg.oc.45.18.05.103.

[30] K. Mitsudo , K. Shigemori , H. Mandai , A. Wakamiya , S. Suga , Org. Lett. 2018, 20, 7336–7340, 10.1021/acs.orglett.8b03316. 30372077

[31] R. E. Messersmith , J. D. Tovar , J. Phys. Chem. A 2019, 123, 881–888, 10.1021/acs.jpca.9b00125. 30620595

[32] Y. Cao , N. E. Arsenault , D. Hean , M. O. Wolf , J. Org. Chem. 2019, 84, 5394–5403, 10.1021/acs.joc.9b00398. 31020846

[33] M. Sakai , M. Mori , M. Hirai , N. Ando , S. Yamaguchi , Chem. ‐ Eur. J. 2022, 28, e202200728, 10.1002/chem.202200728. 35412698

[34] Y. Adachi , R. Matsuura , M. Sakabe , H. Tobita , H. Murakami , J. Ohshita , Polym. Chem. 2025, 16, 2751–2756, 10.1039/D5PY00203F.

[35] A. Lik , L. Fritze , L. Müller , H. Helten , J. Am. Chem. Soc. 2017, 139, 5692–5695, 10.1021/jacs.7b01835. 28394590

[36] A. Lik , S. Jenthra , L. Fritze , L. Müller , K.‐N. Truong , H. Helten , Chem. ‐ Eur. J. 2018, 24, 11961–11972, 10.1002/chem.201706124. 29543358

[37] L. Fritze , N. A. Riensch , H. Helten , Synthesis 2019, 51, 399–406, 10.1055/s-0037-1610849.

[38] M. Buckel , J. Klopf , J. S. Schneider , A. Lik , N. A. Riensch , I. Krummenacher , H. Braunschweig , B. Engels , H. Helten , Nat. Commun. 2025, 16, 1775, 10.1038/s41467-025-56892-w. 39971952 PMC11840099

[39] N. A. Riensch , L. Fritze , T. Schindler , M. Kremer , H. Helten , Dalton Trans. 2018, 47, 10399–10403, 10.1039/C8DT01716F. 29850710

[40] N. A. Riensch , M. Fest , L. Fritze , A. Helbig , I. Krummenacher , H. Braunschweig , H. Helten , New J. Chem. 2021, 45, 14920–14924, 10.1039/D0NJ04297H.

[41] L. Fritze , M. Fest , A. Helbig , T. Bischof , I. Krummenacher , H. Braunschweig , M. Finze , H. Helten , Macromolecules 2021, 54, 7653–7665, 10.1021/acs.macromol.1c01267.

[42] L. Swoboda , J. Klopf , M. Buckel , B. Engels , H. Helten , J. Am. Chem. Soc. 2025, 147, 10629–10639, 10.1021/jacs.5c00849. 40053927

[43] H. Braunschweig , A. Damme , J. O. C. Jimenez‐Halla , C. Hörl , I. Krummenacher , T. Kupfer , L. Mailänder , K. Radacki , J. Am. Chem. Soc. 2012, 134, 20169–20177, 10.1021/ja309935t. 23171432

[44] A. Weiss , M. C. Hodgson , P. D. W. Boyd , W. Siebert , P. J. Brothers , Chem. ‐ Eur. J. 2007, 13, 5982–5993, 10.1002/chem.200700046. 17570718

[45] P. J. Brothers , Chem. Commun. 2008, 18, 2090–2102, 10.1039/b714894a. 18438481

[46] S. Shimizu , Chem. Rev. 2017, 117, 2730–2784, 10.1021/acs.chemrev.6b00403. 27779851

[47] A. C. Y. Tay , B. J. Frogley , D. C. Ware , P. J. Brothers , Dalton Trans. 2018, 47, 3388–3399, 10.1039/C7DT04575A. 29431798

[48] A. Sinha , T. Chatterjee , M. Ravikanth , Coord. Chem. Rev. 2022, 465, 214574, 10.1016/j.ccr.2022.214574.

[49] L. Liu , J. Kim , L. Xu , Y. Rao , M. Zhou , B. Yin , J. Oh , D. Kim , A. Osuka , J. Song , Angew. Chem. Int. Ed. 2022, 61, e202214342, 10.1002/anie.202214342. 36227657

[50] A. Yella , H.‐W. Lee , H. N. Tsao , C. Yi , A. K. Chandiran , M. K. Nazeeruddin , E. W.‐G. Diau , C.‐Y. Yeh , S. M. Zakeeruddin , M. Grätzel , Science 2011, 334, 629–634, 10.1126/science.1209688. 22053043

[51] S. Mathew , A. Yella , P. Gao , R. Humphry‐Baker , B. F. E. Curchod , N. Ashari‐Astani , I. Tavernelli , U. Rothlisberger , M. K. Nazeeruddin , M. Grätzel , Nat. Chem. 2014, 6, 242–247, 10.1038/nchem.1861. 24557140

[52] T. Ema , Y. Miyazaki , J. Shimonishi , C. Maeda , J.‐y. Hasegawa , J. Am. Chem. Soc. 2014, 136, 15270–15279, 10.1021/ja507665a. 25268908

[53] N. Heppe , C. Gallenkamp , S. Paul , N. Segura‐Salas , N. von Rhein , B. Kaiser , W. Jaegermann , A. Jafari , I. Sergueev , V. Krewald , U. I. Kramm , Chem. ‐ Eur. J. 2023, 29, e202202465, 10.1002/chem.202202465. 36301727

[54] H. Sheng , J. Wang , J. Huang , Z. Li , G. Ren , L. Zhang , L. Yu , M. Zhao , X. Li , G. Li , N. Wang , C. Shen , G. Lu , Nat. Commun. 2023, 14, 1528, 10.1038/s41467-023-37271-9. 36934092 PMC10024688

[55] S. Gu , A. N. Marianov , T. Lu , J. Zhong , Chem. Eng. J. 2023, 470, 144249, 10.1016/j.cej.2023.144249.

[56] J. C. Barona‐Castaño , C. C. Carmona‐Vargas , T. J. Brocksom , K. T. De Oliveira , Molecules 2016, 21, 310, 10.3390/molecules21030310. 27005601 PMC6273917

[57] M. Liu , Y.‐J. Chen , X. Huang , L.‐Z. Dong , M. Lu , C. Guo , D. Yuan , Y. Chen , G. Xu , S.‐L. Li , Y.‐Q. Lan , Angew. Chem. Int. Ed. 2022, 61, e202115308, 10.1002/anie.202115308. 35018705

[58] S. Rajasree , X. Li , P. Deria , Commun. Chem. 2021, 4, 47, 10.1038/s42004-021-00484-4. 36697594 PMC9814740

[59] A. Treibs , F.‐H. Kreuzer , Justus Liebigs Ann. Chem. 1968, 718, 208–223, 10.1002/jlac.19687180119. 5704489

[60] A. Loudet , K. Burgess , Chem. Rev. 2007, 107, 4891–4932, 10.1021/cr078381n. 17924696

[61] T. Kowada , H. Maeda , K. Kikuchi , Chem. Soc. Rev. 2015, 44, 4953–4972, 10.1039/C5CS00030K. 25801415

[62] D. Zhang , V. Martín , I. García‐Moreno , A. Costela , M. E. Pérez‐Ojeda , Y. Xaio , Phys. Chem. Chem. Phys. 2011, 13, 13026–13033, 10.1039/c1cp21038f. 21691659

[63] R. Toyoda , N. V. Hoang , K. G. Moghaddam , S. Crespi , D. R. S. Pooler , S. Faraji , M. S. Pshenichnikov , B. L. Feringa , Nat. Commun. 2022, 13, 5765, 10.1038/s41467-022-33177-0. 36180434 PMC9525625

[64] S. S. Rajasree , X. Li , P. Deria , Commun. Chem 2021, 4, 47, 10.1038/s42004-021-00484-4. 36697594 PMC9814740

[65] A. Kamkaew , S. H. Lim , H. B. Lee , L. V. Kiew , L. Y. Chung , K. Burgess , Chem. Soc. Rev. 2013, 42, 77–88, 10.1039/C2CS35216H. 23014776 PMC3514588

[66] J. Killoran , L. Allen , J. F. Gallagher , W. M. Gallagher , D. F. O′Shea , Chem. Commun. 2002, 1862–1863, 10.1039/B204317C. 12271646

[67] A. Gorman , J. Killoran , C. O'Shea , T. Kenna , W. M. Gallagher , D. F. O'Shea , J. Am. Chem. Soc. 2004, 126, 10619–10631, 10.1021/ja047649e. 15327320

[68] K. Fujimoto , H. Yorimitsu , A. Osuka , Chem. ‐ Eur. J. 2015, 21, 11311–11314, 10.1002/chem.201502215. 26177584

[69] K. Fujimoto , J. Oh , H. Yorimitsu , D. Kim , A. Osuka , Angew. Chem. Int. Ed. 2016, 55, 3196–3199, 10.1002/anie.201511981. 26821874

[70] W. Stawski , K. Hurej , J. Skonieczny , M. Pawlicki , Angew. Chem. Int. Ed. 2019, 58, 10946–10950, 10.1002/anie.201904819. 31141278

[71] G.‐L. Fu , H. Pan , Y.‐H. Zhao , C.‐H. Zhao , Org. Biomol. Chem. 2011, 9, 8141–8146, 10.1039/c1ob05959a. 22015954

[72] H. Sun , X. Dong , S. Liu , Q. Zhao , X. Mou , H. Y. Yang , W. Huang , J. Phys. Chem. C 2011, 115, 19947–19954, 10.1021/jp206396v.

[73] R. P. Nandi , C. A. Swamy P , P. Dhanalakshmi , S. K. Behera , P. Thilagar , Inorg. Chem. 2021, 60, 5452–5462, 10.1021/acs.inorgchem.0c02739. 33830747

[74] C. A. Swamy P , S. Mukherjee , P. Thilagar , Chem. Commun. 2013, 49, 993–995, 10.1039/C2CC38352G. 23254480

[75] R. Misra , T. Jadhav , B. Dhokale , S. M. Mobin , Dalton Trans. 2015, 44, 16052–16060, 10.1039/C5DT02356D. 26285867

[76] S. K. Sarkar , P. Thilagar , Chem. Commun. 2013, 49, 8558–8560, 10.1039/c3cc42979b. 23945837

[77] J. O. Huh , Y. Do , M. H. Lee , Organometallics 2008, 27, 1022–1025, 10.1021/om701237t.

[78] S. K. Sarkar , S. Jena , S. K. Behera , P. Thilagar , J. Org. Chem. 2022, 87, 3967–3977, 10.1021/acs.joc.1c02595. 35254826

[79] S. K. Sarkar , G. R. Kumar , P. Thilagar , Chem. Commun. 2016, 52, 4175–4178, 10.1039/C6CC00823B. 26906475

[80] C. A. Swamy P , R. N. Priyanka , S. Mukherjee , P. Thilagar , Eur. J. Inorg. Chem. 2015, 2015, 2338–2344, 10.1002/ejic.201500089.

[81] B. Wrackmeyer , H. Nöth , Chem. Ber. 1976, 109, 1075–1088, 10.1002/cber.19761090329.

[82] J. D. Odom , T. F. Moore , R. Goetze , H. Nöth , B. Wrackmeyer , J. Organomet. Chem. 1979, 173, 15–32, 10.1016/S0022-328X(00)91231-2.

[83] J. Faderl , B. Deobald , R. Guilard , H. Pritzkow , W. Siebert , Eur. J. Inorg. Chem. 1999, 1999, 399–404, 10.1002/(SICI)1099-0682(199903)1999:3<399::AID-EJIC399>3.0.CO;2-I.

[84] T. Taniguchi , S. Yamaguchi , Organometallics 2010, 29, 5732–5735, 10.1021/om100408m.

[85] H. Omori , H. Shinokubo , Organometallics 2019, 38, 2878–2882, 10.1021/acs.organomet.9b00303.

[86] N. K. Kalluvettukuzhy , S. Pagidi , R. Prasad Nandi , P. Thilagar , Asian J. Org. Chem. 2020, 9, 644–651, 10.1002/ajoc.201900756.

[87] A. R. Katritzky , K. Akutagawawa , Org. Prep. Proced. Int. 1988, 20, 585–590, 10.1080/00304948809356302.

[88] R. V. Bynum , W. E. Hunter , R. D. Rogers , J. L. Atwood , Inorg. Chem. 1980, 19, 2368–2374, 10.1021/ic50210a039.

[89] R. V. Bynum , H.‐M. Zhang , W. E. Hunter , J. L. Atwood , Can. J. Chem. 1986, 64, 1254–1257, 10.1139/v86-216.

[90] M. R. Mason , D. Ogrin , B. Fneich , T. S. Barnard , K. Kirschbaum , J. Organomet. Chem. 2005, 690, 157–162, 10.1016/j.jorganchem.2004.09.003.

[91] For emissive zirconium(IV) or titanium(IV) complexes, see, e.g.: E. L. Patrick , C. J. Ray , G. D. Meyer , T. P. Ortiz , J. A. Marshall , J. A. Brozik , M. A. Summers , J. W. Kenney , J. Am. Chem. Soc. 2003, 125, 5461–5470, 10.1021/ja028769u. 12720460

[92] G. V. Loukova , W. Huhn , V. P. Vasiliev , V. A. Smirnov , J. Phys. Chem. A 2007, 111, 4117–4121, 10.1021/jp0721797. 17474731

[93] B. Urbán , D. Dunlop , R. Gyepes , P. Kubát , K. Lang , M. Horáček , J. Pinkas , L. Šimková , M. Lamač , Organometallics 2023, 42, 1373–1385, 10.1021/acs.organomet.3c00082.

[94] C. Arivazhagan , A. Maity , K. Bakthavachalam , A. Jana , S. K. Panigrahi , E. Suresh , A. Das , S. Ghosh , Chem. ‐ Eur. J. 2017, 23, 7046–7051, 10.1002/chem.201700187. 28376247

[95] R. Hu , E. Lager , A. Aguilar‐Aguilar , J. Liu , J. W. Y. Lam , H. H. Y. Sung , I. D. Williams , Y. Zhong , K. S. Wong , E. Pena‐Cabrera , B. Z. Tang , J. Phys. Chem. C 2009, 113, 15845–15853, 10.1021/jp902962h.

[96] D. C. Ebner , J. T. Bagdanoff , E. M. Ferreira , R. M. McFadden , D. D. Caspi , R. M. Trend , B. M. Stoltz , Chem. ‐ Eur. J. 2009, 15, 12978–12992, 10.1002/chem.200902172. 19904777 PMC2862982

[97] Y. Dienes , S. Durben , T. Kárpáti , T. Neumann , U. Englert , L. Nyulászi , T. Baumgartner , Chem. ‐ Eur. J. 2007, 13, 7487–7500, 10.1002/chem.200700399. 17579902

[98] G. M. Sheldrick , Acta Crystallogr. A 2015, 71, 3–8, 10.1107/S2053273314026370. PMC4283466 25537383

[99] G. M. Sheldrick , Acta Crystallogr. A 2008, 64, 112–122, 10.1107/S0108767307043930. 18156677

[100] C. B. Hübschle , G. M. Sheldrick , B. Dittrich , J. Appl. Crystallogr. 2011, 44, 1281–1284, 10.1107/S0021889811043202. 22477785 PMC3246833

[101] R. Ahlrichs , M. Bär , M. Häser , H. Horn , C. Kölmel , Chem. Phys. Lett. 1989, 162, 165–169, 10.1016/0009-2614(89)85118-8.

[102] Gaussian 98 g16, Revision C.01, M. J. Frisch , G. W. Trucks , H. B. Schlegel , G. E. Scuseria , M. A. Robb , J. R. Cheeseman , G. Scalmani , V. Barone , G. A. Petersson , H. Nakatsuji , X. Li , M. Caricato , A. V. Marenich , J. Bloino , B. G. Janesko , R. Gomperts , B. Mennucci , H. P. Hratchian , J. V. Ortiz , A. F. Izmaylov , J. L. Sonnenberg , D. Williams‐Young , F. Ding , F. Lipparini , F. Egidi , J. Goings , B. Peng , A. Petrone , T. Henderson , D. Ranasinghe , et al, Gaussian, Inc., Wallingford CT, 2019.

[103] P. A. M. Dirac , Proc. R. Soc. London, Ser. A 1929, 123, 714–733.

[104] J. C. Slater , Phys. Rev. 1951, 81, 385–390, 10.1103/PhysRev.81.385.

[105] A. D. Becke , Phys. Rev. A 1988, 38, 3098–3100, 10.1103/PhysRevA.38.3098. 9900728

[106] C. Lee , W. Yang , R. G. Parr , Phys. Rev. B 1988, 37, 785–789, 10.1103/PhysRevB.37.785. 9944570

[107] A. D. Becke , J. Chem. Phys. 1993, 98, 5648–5652, 10.1063/1.464913.

[108] S. Grimme , J. Antony , S. Ehrlich , H. Krieg , J. Chem. Phys. 2010, 132, 154104, 10.1063/1.3382344. 20423165

[109] S. Grimme , S. Ehrlich , L. J. Goerigk , J. Comput. Chem. 2011, 32, 1456–1465, 10.1002/jcc.21759. 21370243

[110] P. Deglmann , F. Furche , R. Ahlrichs , Chem. Phys. Lett. 2002, 362, 511–518, 10.1016/S0009-2614(02)01084-9.

[111] P. Deglmann , F. Furche , J. Chem. Phys. 2002, 117, 9535–9538, 10.1063/1.1523393.

[112] R. Bauernschmitt , R. Ahlrichs , Chem. Phys. Lett. 1996, 256, 454–464, 10.1016/0009-2614(96)00440-X.

[113] R. Bauernschmitt , R. Ahlrichs , J. Chem. Phys. 1996, 104, 9047–9052, 10.1063/1.471637.

[114] F. Furche , D. Rappoport , in Computational Photochemistry (Ed M. Olivucci ), 16, Elsevier, Amsterdam 2005.

[115] A. Schäfer , H. Horn , R. Ahlrichs , J. Chem. Phys. 1992, 97, 2571–2577, 10.1063/1.463096.

[116] Deposition numbers 2476888 (for **2**), 2476889 (for **4**), 2476890 (for **6**) and 2476891 (for **9**) contain the supplementary crystallographic data for this paper. These data are provided free of charge by the joint Cambridge Crystallographic Data Centre and Fachinformationszentrum Karlsruhe Access Structures service.

