# Supplementary material for: Merging Pyrrole with Boron into Versatile Di(2‐pyrryl)borane Building Blocks: π‐Extension, Polymerization, and Coordination
Source: Angew Chem Int Ed Engl. 2025 Nov 17;65(2):e16982. doi: 10.1002/anie.202516982 (PMC12790361; doi:10.1002/anie.202516982)
Supplement: Supplementary file 1 — Supporting Information [file ANIE-65-e16982-s001.pdf]

Supporting Information
©Wiley-VCH 2021
69451 Weinheim, Germany

Merging Pyrrole with Boron into Versatile Di(2-pyrryl)borane Building Blocks: π-Extension, Polymerization, and Coordination

Daniel Göbel, Andreas Helbig, Alexandra Friedrich, Jonas Bachmann, Manuel Buckel, Johannes Chorbacher, and Holger Helten*

Julius-Maximilians-Universität Würzburg, Institute of Inorganic Chemistry and Institute for Sustainable Chemistry & Catalysis with Boron (ICB), Am Hubland, 97074 Würzburg, Germany

E-mail: *holger.helten@uni-wuerzburg.de*

Author Contributions

The manuscript was written through contributions of all authors.

D.G. and A.H. performed all experimental work, synthesis and characterization of the compounds.

The photophysical measurements and characterization were performed by D.G.

The analysis and evaluation of the XRD measurements were performed by A.F., J.B. and M.B.

DLS measurements were performed by J.C.

A.H. carried out all theoretical calculations.

H.H. led the whole project and wrote the manuscript together with D.G. and A.H.

Thanks to S.L. for contributions to the design of the TOC.

SUPPORTING INFORMATION

Table of Contents

1. Experimental Section 3
1.1 General procedures 3
1.2 Syntheses 4
1.3 Crystallographic data 10
1.4 NMR spectra 13
1.5 High resolution mass spectra 27
1.6 UV/vis and fluorescence spectra 31
1.6.1 General data 31
1.6.2 Lifetime measurements 54
1.6.3 Aggregation emission enhancement 68
1.7 Dynamic light scattering 69
1.8 Cyclic Voltammetry 71
1.9 Gel Permeation Chromatography 90
2. Computational Information 91
3. References 110

1. Experimental Section

1.1 General procedures

All manipulations before the aqueous workup were performed under an atmosphere of dry argon using standard Schlenk techniques or in an MBraun glovebox. Solvents (*n*-hexane, toluene, and THF) were dried and degassed by means of an MBraun SPS-800 solvent purification system. $CDCl_3$, C_6D_6, THF-d_8 and acetonitrile-d3 for NMR spectroscopy as well as 1,4-dioxane were dried and degassed at room temperature or reflux over CaH_2, Na or NaK, respectively, and freshly distilled prior to use. Solvents for aqueous work-up (diethyl ether, petroleum ether, ethyl acetate, *n*-pentane, *n*-hexane), *n*-butyllithium solutions (1.6 M and 2.5 M in *n*-hexane), *t*-butyllithium solutions (1.7 M in *n*-pentane), *N*-Methylpyrrole, 2,4,6-Tri-(*iso*-propyl)phenylbromid, Trimethoxyborane, 2-methoxy-4,4,5,5-dioxaborolane, *N*-bromosuccinimide, tris(dibenzylideneacetone)dipalladium(0), tri-*tert*-butylphosphine, caesium carbonate and zirconocene dichloride were commercially purchased and used as received. 1*H*-Pyrrole, trimethylsilyl chloride, *N*,*N*,*N*',*N*'- tetramethylethylenediamine was purified by inert-gas distillation. 2-(4,4,5,5-tetramethyl-1,3,2-dioxaborolan-2-yl)-furan[1] (**5b**) and 2-(4,4,5,5-tetramethyl-1,3,2-dioxaborolan-2-yl)-thiophene[2] (**5c**) were synthesized according to procedures described in the literature. NMR spectra were recorded at 25 °C on a Bruker Avance III HD spectrometer operating at 300 MHz or on a Bruker Avance 500 spectrometer operating at 500 MHz. Chemical shifts were referenced to residual protic impurities in the solvent (^{1}H) or the deuterio solvent itself (^{13}C) and reported relative to external $SiMe_4$ (^{1}H, ^{13}C), $BF_3{\cdot}OEt_2$ (^{11}B) or LiCl (^{7}Li) standards. Mass spectra were obtained with the use of a Thermo Scientific Exactive Plus Orbitrap MS system employing atmospheric sample analysis probe (ASAP) or liquid injection field desorption ionization (LIFDI). UV–vis spectra were obtained using a Jasco V-630 spectrophotometer. Emission spectra were recorded using an Edinburgh Instruments FLSP920 spectrometer equipped with a double monochromator for both excitation and emission, operating in right-angle geometry mode, and all spectra were fully corrected for the spectral response of the instrument. Fluorescence quantum yields were measured using a calibrated integrating sphere from Edinburgh Instruments combined with the FLSP920 spectrometer described above. Thin films of the (2-pyrryl)boranes were prepared from a solution of 60 mg poly(methyl methacrylate) (PMMA) and 0.25 mg of the compounds (stock solution) in 1.0 mL DCM by slow evaporation on the side of the cuvettes under normal conditions and then drying at 60 °C.

Dynamic light scattering (DLS) experiments were performed using a ALV/CGS-3 Compact Goniometer System with a scattering electronics and multiple tau digital correlator and a 22 mW HeNe-Laser (632.8 nm). Measurements were performed at 25 °C with a scattering angle of 90° and the concentration of the samples kept at $5 \cdot 10^{-5}$ M.

Cyclic voltammetry experiments were performed using a Gamry Interface 1010B potentiostat. A standard three-electrode cell configuration was employed using a platinum disk working electrode, a platinum wire counter electrode, and a silver wire, separated by a Vycor tip, serving as the reference electrode. Tetra-*n*-butylammonium hexafluorophosphate ([*n*-Bu_4N][PF_6]) was employed as the supporting electrolyte. The scans were referenced after the addition of a small amount of ferrocene as internal standard. The potentials are reported relative to the ferrocene/ferrocenium couple. All experiments were measured at room temperature under an argon atmosphere.

1.2 Syntheses

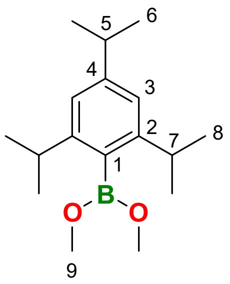

Synthesis of TipB(OMe)₂. To a solution of 2,4,6-Tri-(*iso*-propyl)phenyl bromide (10.00 mL, 11.20 g, 39.54 mmol, 1.0 eq.) in THF (100 ml) *n*-butyllithium (2.5 M, 23.80 ml, 59.50 mmol, 1.5 eq.) was added at –78 °C. The solution was stirred for 30 min at –78 °C and trimethoxy borane (6.60 mL, 6.15 g, 59.20 mmol, 1.5 eq.) was added and the mixture was allowed to warm to r.t. overnight. The solvent was removed under reduced pressure and filtrated using a filtration cannula and *n*-hexane. The solvent was removed again, and the remaining oil was distilled under inert atmosphere resulting in a colorless highly viscous oil. **Yield:** 8.06 g (29.18 mmol, 74 %) **^{1}H NMR** (300 MHz, $CDCl_3$): δ = 7.01 (s, 2H, *m*-Tip-C*H* (3)), 3.62 (s, 6H, O-CH_3 (9)), 2.93 (sept, $^{3}J_{HH}$ = 6.9 Hz, 1H, *p*-*i*Pr-C*H* (5)), 2.70 (sept, $^{3}J_{HH}$ = 6.8 Hz, 2H, *o*-*i*Pr-C*H* (7)), 1.30 (d, $^{3}J_{HH}$ = 6.9 Hz, 18H, *o*/*p*-*i*Pr-CH_3 (6/8)) ppm. **^{11}B{^{1}H} NMR** (96 MHz, $CDCl_3$): δ = 32.1 ppm.

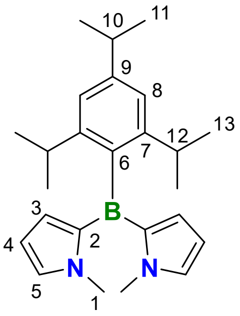

Synthesis of 2. To a solution of *N*-methylpyrrole (1.00 mL, 0.92 g, 11.30 mmol, 1.8 eq.) and *N*,*N*,*N*',*N*'-tetramethylethylenediamine (1.70 mL, 1.31 g, 11.30 mmol, 1.8 eq.) in *n*-hexane (11.4 ml) *n*-butyllithium (2.5 M, 4.52 ml, 11.30 mmol, 1.8 eq.) was added at r.t. and stirred for 3 h. Afterwards 2,4,6-Tri-(*iso*-propyl)phenyldimethoxy borane (1.73 g, 6.28 mmol, 1.0 eq.) was added and the mixture stirred at r.t. for 2 d. The reaction mixture was quenched with excess trimethylsilyl chloride and stirred for an additional 30 min. The brown suspension was extracted with diethyl ether and a saturated aqueous ammonium chloride solution, the organic phase was dried over $MgSO_4$, and the solvent was removed. The residue was purified *via* column chromatography (neutral AlO_x, eluent: *n*-hexane/ THF 3:2) as yellow oil, after crystallization in *n*-pentane as yellowish crystals. **Yield:** 1.24 g (3.31 mmol, 59 %) **^{1}H NMR** (300 MHz, C_6D_6): δ = 7.22 (dd, *J* = 3.8, 1.6 Hz, 2H, Pyr-C*H* (5)), 7.14 (s, 2H, *m*-Tip-C*H* (8)), 6.54 (dd, *J* = 2.5, 1.9 Hz, 2H, Pyr-C*H* (4)), 6.33 (dd, *J* = 3.8, 2.4 Hz, 2H, Pyr-C*H* (3)), 3.02 (s, 6H, N-CH_3 (1)), 2.87 (sept, $^{3}J_{HH}$ = 6.9 Hz, 1 H, *p*-*i*Pr-C*H* (10)), 2.86 (sept, $^{3}J_{HH}$ = 6.8 Hz, 2 H, *o*-*i*Pr-C*H* (12)), 1.28 (d, *J* = 6.9 Hz, 6H, *p*-*i*Pr-CH_3 (11)), 1.08 (d, *J* = 6.8 Hz, 12H, *o*-*i*Pr-CH_3 (13)) ppm. **^{11}B{^{1}H} NMR** (96 MHz, C_6D_6): δ = 51.8 ppm. **^{13}C{^{1}H} NMR** (75 MHz, C_6D_6): δ = 149.5 (s, *p*-Tip-C_q (9)), 149.3 (s, *o*-Tip-C_q (7)), 141.4 (s br, Pyr-C_q (2)), 131.5 (s, Pyr-CH (5)), 128.9 (s, Pyr-*C*H (4)), 120.7 (s, *m*-Tip-CH (8)), 109.7 (s, Pyr-*C*H (3)), 36.6 (s, N-CH_3 (1)), 35.5 (s, *p*-*i*Pr-CH (10)), 34.8 (s, *o*-*i*Pr-CH (12)), 24.5 (s, *p*-*i*Pr-CH_3 (11)), 24.3 (s, *o*-*i*Pr-CH_3 (13)) ppm (Ph-C_q-B (6) was not observed). **HRMS** (LIFDI): m/z ($[M]^+$, $C_{25}H_{35}BN_2$) = calcd. 374.2888; found 374.2885. **UV/Vis** (THF): $\lambda_{abs,max}$ = 342 nm. **Fluorescence** (THF): λ_{ex} = 345, λ_{em} = 446 nm (Φ_F = 14 %).

Alternative synthesis of 2. To a solution of **4** (34.6 mg, 0.10 mmol, 1.0 eq.) in 1 mL THF was added *t*-butyllithium (1.7 M, 0.12 mL, 0.20 mmol, 2.0 eq.) dropwise at –78 °C. Subsequently, the reaction mixture turned yellow. After stirring for 1.5 h at –78 °C, methyl iodide (31.2 mg, 0.22 mmol, 2.2 eq.) was added and the reaction mixture was allowed to slowly warm up to

room temperature and stirred overnight. Then, all volatiles were removed *in vacuo* and the residue was further purified *via* column chromatography (SiO_x, eluent: petroleum ether / ethyl acetate 20:1) to obtain compound **2** as an offwhite solid. **Yield:** 66 % (determined by NMR), 14.0 mg (37.4 μmol, 37 %, isolated).

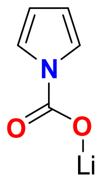

Synthesis of 3. A solution of 1*H*-pyrrole (6.94 mL, 6.71 g, 100.0 mmol, 1.0 eq.) in *n*-hexane (140 ml) was treated with *n*-butyllithium (2.5 M, 40 mL, 100.0 mmol, 1.0 eq.) at –78 °C and was allowed to warm up to r.t. within 15 min. Afterwards an excess of CO_2 was inserted via an inert canula, while stirring was maintained. The solvent was removed, and the residue washed with *n*-pentane to obtain a colorless solid. **Yield:** 10.76 g (91.9 mmol, 92 %) **^{1}H NMR** (300 MHz, THF-d_8): δ = 7.28 (t, $^3J_{HH}$ = 2.2 Hz, 2 H, Pyr-C*H*), 5.95 (t, $^3J_{HH}$ = 2.2 Hz, 2 H, Pyr-C*H*) ppm. **^{7}Li{^{1}H} NMR** (117 MHz, THF-d_8): δ = 0.32 (s) ppm.

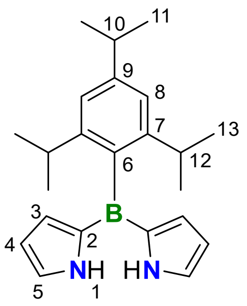

Synthesis of 4. To a solution of **3** (409.6 mg, 3.50 mmol, 2.0 eq.) in THF (70 ml) was added *t*-butyllithium (1.7 M, 2.06 mL, 3.50 mmol, 2.0 eq.) dropwise at –78 °C. Subsequently, the reaction mixture turned yellow. After stirring for 1.5 h at –78 °C, 2,4,6-Tri-(*iso*-propyl)phenyldimethoxy borane (483.4 mg, 1.75 mmol, 1.0 eq.) was added and the reaction mixture was allowed to slowly warm up to room temperature and stirred overnight. The reaction mixture turned beige, and a colorless precipitate was formed. After refluxing for 1 d, all volatiles were removed *in vacuo*. The residue was extracted with petroleum ether and water, the organic phase was dried over $MgSO_4$, and the solvent was removed under reduced pressure. The crude product was purified *via* column chromatography (SiO_x, eluent: petroleum ether / ethyl acetate 10:1) to obtain compound **4** as a colorless solid. **Yield:** 287.1 mg (829.0 μmol, 47 %) **^{1}H NMR** (500 MHz, C_6D_6): δ = 8.04 (br s, 2 H, N*H* (1)), 7.36 (m, 2 H, Pyr-C*H* (3)), 7.28 (s, 2 H, *m*-Tip-C*H* (8)), 6.42 (td, $^3J_{HH}$ = 2.6 Hz, $^4J_{HH}$ = 1.2 Hz, 2 H, Pyr-C*H* (5)), 6.38 (dt, $^3J_{HH}$ = 3.6 Hz, $^4J_{HH}$ = 2.3 Hz, 2 H, Pyr-C*H* (4)), 2.96 (sept, $^3J_{HH}$ = 7.0 Hz, 1 H, *p*-*i*Pr-C*H* (10)), 2.78 (sept, $^3J_{HH}$ = 6.8 Hz, 2 H, *o*-*i*Pr-C*H* (12)), 1.37 (d, $^3J_{HH}$ = 6.9 Hz, 6 H, *p*-*i*Pr-CH_3 (11)), 1.08 (d, $^3J_{HH}$ = 6.8 Hz, 12 H, *o*-*i*Pr-CH_3 (13)) ppm. **^{11}B{^{1}H} NMR** (160 MHz, C_6D_6): δ = 47.9 (br s) ppm. **^{13}C{^{1}H} NMR** (126 MHz, C_6D_6): δ = 151.0 (s, *o*-Tip-C_q (7)), 148.8 (s, *p*-Tip-C_q (9)), 138.5 (br s, Ph-C_q-B (6)), 136.5 (br s, Pyr-C_q (2)), 124.9 (s, Pyr-*C*H (5)), 123.7 (br s, Pyr-*C*H (3)), 120.4 (*m*-Tip-*C*H (8)), 111.1 (s, Pyr-*C*H (4)), 34.8 (s, *o*-*i*Pr-*C*H (12)), 34.7 (s, *p*-*i*Pr-*C*H (10)), 24.3 (s, *o*-*i*Pr-*C*H_3 (13)), 24.2 (s, *p*-*i*Pr-*C*H_3 (11)) ppm. **HRMS** (LIFDI): m/z ($C_{23}H_{31}BN_2$) = calcd. 346.2575, found: 346.2573. **UV/Vis** (THF): $\lambda_{abs,max}$ = 336 nm. **Fluorescence** (THF): λ_{ex} = 320, λ_{em1} = 357, λ_{em2} = 434 nm (Φ_F = 26 %).

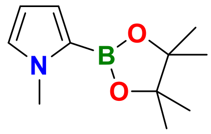

Synthesis of 5a. To a solution of *N*-methylpyrrole (0.36 mL, 324.5 mg, 4.00 mmol, 1.0 eq.) and *N*,*N*,*N'*,*N'*-tetramethylethylenediamine (0.66 mL, 511.3 mg, 4.40 mmol, 1.1 eq.) in *n*-hexane (40.0 mL) *n*-butyllithium (2.5 M, 1.76 ml, 4.40 mmol, 1.1 eq.) was added at r.t. and stirred overnight. Then, 2-Methoxy-4,4,5,5-dioxaborolane (1.00 mL, 964.0 mg,

6.1 mmol, 1.5 eq.) was added and the reaction mixture was stirred for 3 h. Subsequently, water was added, and the desired product was extracted with *n*-hexane. The organic phase was dried over $MgSO_4$, and the solvent was removed under reduced pressure to obtain compound **5a** as a colorless liquid. **Yield:** 419.0 mg (2.02 mmol, 51 %) **^{1}H NMR** (300 MHz, $CDCl_3$): δ = 6.81 (m, 2 H, Pyr-C*H*), 6.15 (dd, 1 H, Pyr-C*H*), 3.84 (s, 3 H, N-C*H*$_3$), 1.31 (s, 12 H, C-C*H*$_3$) ppm. **^{11}B{^{1}H} NMR** (96 MHz, $CDCl_3$): δ = 28.1 (br s) ppm.

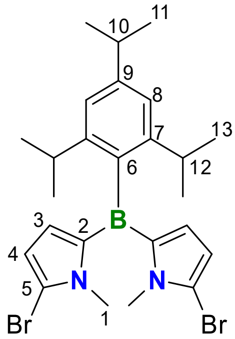

Synthesis of 6. To a solution of **2** (612.0 mg, 1.63 mmol, 1.0 eq.) in THF (0.1M) 581.9 mg (3.27 mmol, 2.0 eq.) of *N*-bromosuccinimide were added at r.t. and allowed to stir for 40 minutes in the absence of light. After removal of the solvent, the residue was treated with sodium hydrogencarbonate, extracted with *n*-hexane and washed with water and brine. The organic fractions were combined, dried over $MgSO_4$ and the solvent was removed, and the remaining oil was washed with *n*-hexane to get an off-white solid.

Yield: 712.6 mg (1.34 mmol, 82 %). **^{1}H NMR** (300 MHz, C_6D_6): δ = 7.10 (s, 2 H, *m*-Tip-C*H* (8)), 6.90 (d, *J* = 3.9 Hz, 2 H, Pyr-C*H* (4)), 6.34 (d, *J* = 4.0 Hz, 2 H, Pyr-C*H* (3)), 3.07 (s, 6 H, N-C*H*$_3$ (1)), 2.82 (dd, *J* = 13.9, 6.9 Hz, 1 H, *p*-*i*Pr-C*H* (10)), 2.72 (dd, *J* = 13.7, 6.9 Hz, 2 H, *o*-*i*Pr-C*H* (12)), 1.26 (d, J = 6.9 Hz, 6 H, *p*-*i*Pr-C*H*$_3$ (11)), 1.03 (d, J = 6.8 Hz, 12 H, *o*-*i*Pr-C*H*$_3$ (13)) ppm. **^{11}B{^{1}H} NMR** (96 MHz, C_6D_6): δ = 48.4 ppm. **^{13}C{^{1}H} NMR** (75 MHz, C_6D_6): δ = 149.8 (s, *p*-Tip-*C*$_q$ (9)), 149.8 (s, *o*-Tip-*C*$_q$ (7)), 128.6 (s, Pyr-*C*H (4)), 121.0 (s, *m*-Tip-CH (8)), 113.8 (s, Pyr-*C*$_q$ (5)), 112.8 (s, Pyr-*C*H (3)), 35.3 (s, N-*C*H$_3$ (1)), 35.2 (s, *p*-*i*Pr-*C*H (10)), 34.8 (s, *o*-*i*Pr-*C*H (12)), 24.0 (s, *p*-*i*Pr-*C*H$_3$ (11)), 23.9 (s, *o*-*i*Pr-*C*H$_3$ (13)) ppm (Pyr-*C*$_q$ (2) and Ph-*C*$_q$-B (6) were not observed). **HRMS** (LIFDI pos): m/z ([M]^0, $C_{25}H_{33}BBr_2N_2$) = calcd. 532.1078; found 532.1072.

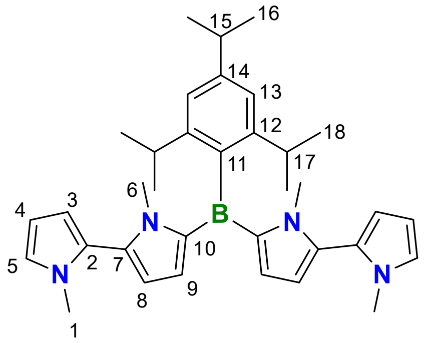

Synthesis of 7a. To a solution of **6** (133.0 mg, 0.25 mmol, 1.0 eq.), tris(dibenzylideneacetone)dipalladium(0) (22.9 mg, 25.0 µmol, 0.1 eq.), tri-*tert*-butylphosphine (10.1 mg, 50.0 µmol, 0.2 eq.) and caesium carbonate (244.4 mg, 0.75 mmol, 3.0 eq.) in toluene (9 mL) and 1,4-dioxane (9 mL) was added compound **5a** (181.2 mg, 0.88 mmol, 3.5 eq.). The reaction mixture was stirred at 125 °C for 20 h. After removal of the solvents *in vacuo*, the residue was extracted with *n*-hexane and brine. The organic fractions were combined, dried over $MgSO_4$ and the solvent was removed. The crude product was purified *via* column chromatography (SiO_x, eluent: *n*-hexane / ethyl acetate 100:0.5) to obtain compound **7a** as a yellow solid. **Yield:** 37.2 mg (69.9 µmol, 28 %) **^{1}H NMR** (500 MHz, CD_3CN): δ = 7.05 (s, 2 H, *m*-Tip-C*H* (13)), 6.78 (m, 4 H, Pyr-C*H* (5, 9)), 6.29 (d, *J* = 3.8 Hz, 2 H, Pyr-C*H* (8)), 6.12 (dd, *J* = 3.6, 2.7 Hz, 2 H, Pyr-C*H* (4)), 6.08 (dd, *J* = 3.6, 1.7 Hz, 2 H, Pyr-C*H* (3)), 3.49 (s, 6 H, N-C*H*$_3$

(1)), 3.19 (s, 6 H, N-C*H*$_3$ (6)), 2.89 (sept, *J* = 7.0 Hz, 1 H, *p*-*i*Pr-C*H* (15)), 2.68 (sept, *J* = 6.8 Hz, 2 H, *o*-*i*Pr-C*H* (17)), 1.25 (d, *J* = 6.9 Hz, 6 H, *p*-*i*Pr-C*H*$_3$ (16)), 1.01 (d, *J* = 6.8 Hz, 12 H, *o*-*i*Pr-C*H*$_3$ (18)) ppm. **^{11}B{^{1}H} NMR** (160 MHz, CD_3CN): δ = 50.5 (br s) ppm. **^{13}C{^{1}H} NMR** (126 MHz, CD_3CN): δ = 150.1 (s, *o*-Tip-*C*$_q$ (12)), 150.0 (s, *p*-Tip-*C*$_q$ (14)), 143.3 (s br, Pyr-*C*$_q$ (10)), 141.7 (s br, Ph-*C*$_q$-B (11)) 135.3 (s, Pyr-*C*$_q$ (7)), 127.5 (s, Pyr-*C*H (9)), 125.7 (s, Pyr-*C*$_q$ (2)), 124.5 (s, Pyr-*C*H (5)), 121.4 (s, *m*-Tip-*C*H (13)), 112.6 (s, Pyr-*C*H (8)), 111.8 (s, Pyr-*C*H (3)), 108.4 (s, Pyr-*C*H (4)), 35.8 (s, *o*-*i*Pr-*C*H (17)), 35.2 (s, *p*-*i*Pr-*C*H (15)), 35.1 (s, N-*C*H$_3$ (6)), 35.0 (s, N-*C*H$_3$ (1)), 24.5 (s, *p*-*i*Pr-*C*H$_3$ (16)), 24.3 (s, *o*-*i*Pr-*C*H$_3$ (18)) ppm. **HRMS** (APCI pos): m/z ($C_{35}H_{45}BN_4$) = calcd. 532.3732, found: 532.3731. **UV/Vis** (THF): $\lambda_{abs,max}$ = 374 nm. **Fluorescence** (THF): λ_{ex} = 370, λ_{em} = 511 nm (Φ_F = 46 %).

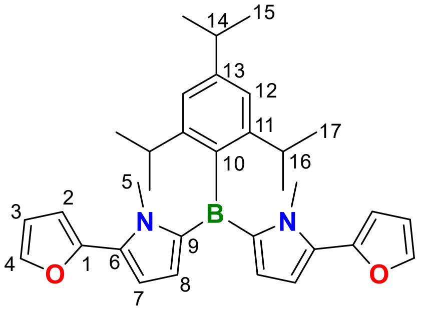

Synthesis of 7b. To a solution of **6** (106.4 mg, 0.20 mmol, 1.0 eq.), tris(dibenzylideneacetone)dipalladium(0) (18.3 mg, 20.0 µmol, 0.1 eq.), tri-*tert*-butylphosphine (8.09 mg, 40.0 µmol, 0.2 eq.) and caesium carbonate (195.5 mg, 0.60 mmol, 3.0 eq.) in 1,4-dioxane (6 mL) was added 2-(4,4,5,5-tetramethyl-1,3,2-dioxaborolan-2-yl)furan (116.4 mg, 0.60 mmol, 3.0 eq.). The reaction mixture was stirred at 125 °C for 3 d. After removal of the solvents *in vacuo*, the residue was extracted with petroleum ether and brine. The organic fractions were combined, dried over $MgSO_4$ and the solvent was removed. The crude product was purified *via* flash column chromatography (SiO_x, eluent: cyclohexane) to obtain compound **7b** as a yellow solid. **Yield:** 17.5 mg (34.6 µmol, 17 %) **^{1}H NMR** (500 MHz, CD_3CN): δ = 7.55 (dd, *J* = 1.8, 0.7 Hz, 2 H, Fur-C*H* (3)), 7.07 (s, 2 H, *m*-Tip-C*H* (12)), 6.66 (d, *J* = 3.9 Hz, 2 H, Pyr-C*H* (8)), 6.57 (dd, *J* = 3.4, 0.7 Hz, 2 H, Fur-C*H* (4)), 6.56 (d, *J* = 3.9 Hz, 2 H, Pyr-C*H* (7)), 6.54 (dd, *J* = 3.4, 1.8 Hz, 2 H, Fur-C*H* (2)), 3.51 (s, 6 H, N-C*H*$_3$ (5)), 2.91 (sept, *J* = 7.0 Hz, 1 H, *p*-*i*Pr-C*H* (14)), 2.65 (sept, *J* = 6.8 Hz, 2 H, *o*-*i*Pr-C*H* (16)), 1.27 (d, *J* = 6.9 Hz, 6 H, *p*-*i*Pr-C*H*$_3$ (15)), 1.00 (d, *J* = 6.8 Hz, 12 H, *o*-*i*Pr-C*H*$_3$ (17)) ppm. **^{11}B{^{1}H} NMR** (160 MHz, CD_3CN): δ = 50.4 (br s) ppm. **^{13}C{^{1}H} NMR** (126 MHz, CD_3CN): δ = 150.3 (s, *o*-Tip-*C*$_q$ (11)), 150.1 (s, *p*-Tip-*C*$_q$ (13)), 148.2 (s, Fur-*C*$_q$ (1)), 144.6 (s br, Pyr-*C*$_q$(9)), 143.4 (s, Fur-*C*H (3)), 141.4 (s br, Ph-*C*$_q$-B (10)), 134.2 (s, Pyr-*C*$_q$ (6)), 127.7 (s, Pyr-*C*H (8)), 121.5 (s, *m*-Tip-*C*H (12)), 112.5 (s, Fur-*C*H (2)), 110.6 (s, Pyr-*C*H (7)), 108.8 (s, Fur-*C*H (4)), 36.2 (s, N-*C*H$_3$ (5)), 35.8 (s, *o*-*i*Pr-*C*H (16)), 35.1 (s, *p*-*i*Pr-*C*H (14)), 24.4 (s, *p*-*i*Pr-*C*H$_3$ (15)), 24.3 (s, *o*-*i*Pr-*C*H$_3$ (17)) ppm. **HRMS** (APCI pos): m/z ($C_{33}H_{39}B\ N_2O_2$) = calcd. 506.3099, found: 506.3091. **UV/Vis** (THF): $\lambda_{abs,max}$ = 386 nm. **Fluorescence** (THF): λ_{ex} = 375, λ_{em} = 503 nm (Φ_F = 34 %).

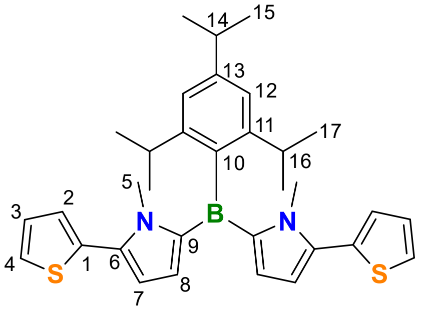

Synthesis of 7c. To a solution of **6** (106.4 mg, 0.20 mmol, 1.0 eq.), tris(dibenzylideneacetone)dipalladium(0) (18.3 mg, 20.0 µmol, 0.1 eq.), tri-*tert*-butylphosphine (8.09 mg, 40.0 µmol, 0.2 eq.) and caesium carbonate (195.5 mg, 0.60 mmol, 3.0 eq.) in 1,4-dioxane (6 mL) was added 2-(4,4,5,5-tetramethyl-1,3,2-dioxaborolan-2-yl)thiophene (116.4 mg, 0.60 mmol, 3.0 eq.). The reaction mixture was stirred at 125 °C for 3 d. After removal of the solvents *in vacuo*, the residue was extracted with petroleum

ether and brine. The organic fractions were combined, dried over $MgSO_4$ and the solvent was removed. The crude product was purified *via* flash column chromatography (SiO_x, eluent: cyclohexane) to obtain compound **7c** as a yellow solid. **Yield:** 28.8 mg (53.5 µmol, 27 %) **^{1}H NMR** (500 MHz, CD_3CN): δ = 7.42 (dd, *J* = 5.1, 1.1 Hz, 2 H, Thi-C*H* (3)), 7.15 (dd, *J* = 3.6 Hz, 1.1 Hz, 2 H, Thi-C*H* (4)), 7.11 (dd, *J* = 5.1, 3.6 Hz, 2 H, Thi-C*H* (2)), 7.07 (s, 2 H, *m*-Tip-C*H* (12)), 6.72 (d, *J* = 3.8 Hz, 2 H, Pyr-C*H* (8)), 6.46 (d, *J* = 3.8 Hz, 2 H, Pyr-C*H* (7)), 3.44 (s, 6 H, N-CH_3 (5)), 2.91 (sept, *J* = 6.9 Hz, 1 H, *p*-*i*Pr-C*H* (14)), 2.66 (sept, *J* = 6.8 Hz, 2 H, *o*-*i*Pr-C*H* (16)), 1.26 (d, *J* = 6.9 Hz, 6 H, *p*-*i*Pr-CH_3 (15)), 1.01 (d, *J* = 6.8 Hz, 12 H, *o*-*i*Pr-CH_3 (17)) ppm. **^{11}B{^{1}H} NMR** (160 MHz, CD_3CN): δ = 50.1 (br s) ppm. **^{13}C{^{1}H} NMR** (126 MHz, CD_3CN): δ = 150.3 (s, *o*-Tip-C_q (11)), 150.2 (s, *p*-Tip-C_q (13)), 144.8 (s br, Pyr-C_q(9)), 141.3 (s br, Ph-C_q-B (10)), 136.8 (s, Pyr-C_q (6)), 135.2 (s, Thi-C_q (1)), 128.8 (s, Thi-*C*H (2)), 127.9 (s, Pyr-*C*H (8)), 127.4 (s, Thi-*C*H (4)), 127.0 (s, Thi-*C*H (3)), 121.5 (s, *m*-Tip-*C*H (12)), 112.2 (s, Pyr-*C*H (7)), 35.9 (s, N-*C*H$_3$ (5)), 35.8 (s, *o*-*i*Pr-*C*H (16)), 35.1 (s, *p*-*i*Pr-*C*H (14)), 24.5 (s, *p*-*i*Pr-*C*H$_3$ (15)), 24.3 (s, *o*-*i*Pr-*C*H$_3$ (17)) ppm. **HRMS** (APCI pos): m/z ($C_{33}H_{39}BN_2S_2$) = calcd. 538.2642, found: 538.2635. **UV/Vis** (THF): $\lambda_{abs,max}$ = 388 nm. **Fluorescence** (THF): λ_{ex} = 390, λ_{em} = 495 nm (Φ_F = 42 %).

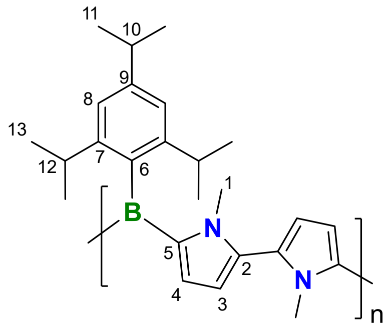

Synthesis of polymer 8. Compound **6** (26.6 mg, 0.05 mmol, 1.0 eq.), bis(cycloocta-1,5-dien)nickel (17.9 mg, 0.07 mmol, 1.3 eq.) and 2,2'-bipyridin (10.2 mg, 0.07 mmol, 1.3 eq.) were suspended in THF (1 mL). Subsequently, the mixture was heated to 80 °C and stirred for 3 d. The reaction mixture was filtered, and the filtrate was precipitated in cooled (–78 °C) methanol (6 mL) and centrifuged. The supernatant liquid was removed by decantation, and the product was dried *in vacuo* to give **8** as a yellow solid. **Yield:** 9.0 mg (24.7 µmol, 48 %) **^{1}H NMR** (300 MHz, C_6D_6): δ = 7.28 (br s, 2 H, Pyr-C*H* (4)), 6.37 (br s, 2 H, Pyr-C*H* (3)), 3.28 (br s, 6H, N-C*H*$_3$ (1)), 2.93 (m, 3H, *o/p*-*i*Pr-C*H* (10/12)), 1.29 (d, *J* = 6.4 Hz, 6H, *p*-*i*Pr-C*H*$_3$ (11)), 1.10 (d, *J* = 5.2 Hz, 12H, *o*-*i*Pr-C*H*$_3$ (13)) ppm (*m*-Tip-C*H* (8) was not observed due to the solvent peak). **GPC** (in THF, vs. polystyrene, detection by RI signal): M_n = 5 508; M_w = 9 845. **UV/Vis** (THF): $\lambda_{abs,max}$ = 415 nm. **Fluorescence** (THF): λ_{ex} = 410, λ_{em} = 487 nm (Φ_F = 39 %)

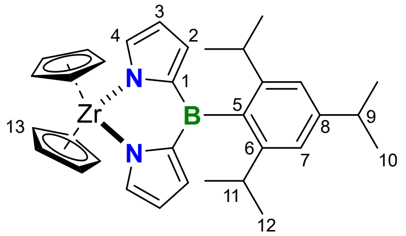

Synthesis of 9. To a solution of **4** (69.3 mg, 0.20 mmol, 1.0 eq.) in 2 mL THF was added *t*-butyllithium (1.7 M, 0.26 mL, 0.44 mmol, 2.2 eq.) dropwise at –78 °C. Subsequently, the reaction mixture turned yellow. After stirring for 1.5 h at –78 °C, zirconocene dichloride (58.5 mg, 0.20 mmol, 2.0 eq.) was added and the reaction mixture was allowed to slowly warm up to room temperature and stirred overnight. The reaction mixture turned red. Then, all volatiles were removed *in vacuo* and the residue was filtered and washed with *n*-pentane. The solvent was removed *in vacuo* and the residue was sublimed (110 °C, $7\cdot10^{-2}$ mbar, 20 h) for further purification to obtain compound **8** as an orange solid. **Yield:** 71.0 mg (125.5 µmol, 25 %) **^{1}H NMR** (500 MHz, C_6D_6): δ = 7.31 (s, 2 H, *m*-Tip-C*H* (7)), 7.10 (dd, *J* = 3.2, 1.1 Hz, 2 H, Pyr-C*H* (2)), 6.57 (dd, *J* = 3.2, 2.0 Hz, 2 H, Pyr-

C*H* (3)), 6.44 (dd, *J* = 2.0, 1.1 Hz, 2H, Pyr-C*H* (4)), 5.75 (s, 10 H, cp-C*H* (13)), 3.19 (sept, *J* = 6.9 Hz, 2H, *o*-*i*Pr-C*H* (11)), 3.01 (sept, *J* = 7.0 Hz, 1H, *p*-*i*Pr-C*H* (9)), 1.39 (d, *J* = 6.9 Hz, 6H, *p*-*i*Pr-CH_3 (10)), 1.36 (d, *J* = 6.8 Hz, 12H, *o*-*i*Pr-CH_3 (12)) ppm. **^{11}B{^{1}H} NMR** (160 MHz, C_6D_6): δ = 47.2 (br s) ppm. **^{13}C{^{1}H} NMR** (126 MHz, C_6D_6): δ = 150.7 (s, *o*-Tip-C_q(6)), 147.5 (s, *p*-Tip-C_q(8)), 132.3 (s, Pyr-*C*H (4)), 132.2 (s, Pyr-*C*H (2)), 128.6 (s, Pyr-C_q(1)), 120.0 (s, *m*-Tip-*C*H (7)), 115.2 (s, cp-*C*H (13)), 114.3 (s, Pyr-*C*H (3)), 35.2 (s, *o*-*i*Pr-*C*H (11)), 34.9 (s, *p*-*i*Pr-*C*H (9)), 24.7 (s, *o*-*i*Pr-CH_3 (12)), 24.7 (s, *p*-*i*Pr-CH_3 (10)) ppm (Ph-C_q-B (5) was not observed). **HRMS** (LIFDI): m/z ($C_{33}H_{39}BN_2Zr$) = calcd. 564.2248, found: 564.2245.

1.3 Crystallographic data

Crystals suitable for single-crystal X-ray diffraction were selected, coated in perfluoropolyether oil, and mounted on MiTeGen sample holders. The crystal data of **2** and **9** were collected on a Rigaku XtaLAB Synergy-R diffractometer with a HPA area detector and multi-layer mirror monochromated Cu_{Ka} radiation. Diffraction data for **4** and **6** were collected on Bruker X8 Apex II 4-circle diffractometers with CCD area detectors using Mo-Kα radiation. The crystals were cooled using an Oxford Cryostreams low-temperature device. Data were collected at 100 K. The images were processed and corrected for Lorentz-polarization effects and absorption as implemented in the Bruker software packages. The structures were solved using the intrinsic phasing method (SHELXT)[3] and Fourier expansion technique. All non-hydrogen atoms were refined in anisotropic approximation, with hydrogen atoms 'riding' in idealized positions, by full-matrix least squares against F^2 of all data, using SHELXL[4] software and the SHELXLE graphical user interface.[5]

Table S1. X-ray crystallographic information.

Compound	**2**	**4**	**6**	**9**
CCDC number	2476888	2476889	2476890	2476891
Empirical formula	C25 H35 B N2	C23 H31 B N2	C25 H33 B Br2 N2	C33 H39 B N2 Zr
M_r	374.36	346.31	532.16	565.69
T / K	100(2)	106(2)	100(2)	100(2)
Radiation, λ / Å	Cu_{Ka}, 1.54184	$Mo_{K\alpha}$, 0.71073	$Mo_{K\alpha}$, 0.71073	$Cu_{K\alpha}$, 1.54184
Crystall size / mm³	0.020 x 0.040 x 0.152	0.106 x 0.225 x 0.295	0.243 x 0.297 x 0.448	0.070 x 0.160 x 0.250
Crystal color, habit	Colorless, needle	colorless, block	orange, block	clear orange, plate
Crystal system	monoclinic	monoclinic	monoclinic	monoclinic
Space group	$P2_1/c$	$P2_1/c$	$P2_1/n$	$P2_1/c$
a / Å	18.1153(5)	25.078(5)	9.283(2)	9.98169(14)
b / Å	9.0125(3)	9.3406(18)	24.981(8)	17.9288(2)
c / Å	28.7383(8)	18.199(5)	11.425(3)	16.3002(2)
α / °	90	90	90	90
β / °	100.505(3)	101.051(8)	107.66(2)	99.8025(13)
γ / °	90	90	90	90
Volume / Å^3	4613.3(2)	4183.9(15)	2524.7(12)	2874.49(7)
Z	8	8	4	4
ρ_{calc} / g cm^{-3}	1.078	1.100	1.400	1.307
μ / mm^{-1}	0.461	0.063	3.225	3.295
F(000)	1632	1504	1088	1184
θ range / °	3.128 - 74.499	2.272 - 26.419	2.041 - 26.054	3.695 - 73.776
Completeness	0.991	0.998	0.997	0.974
Reflections collected	50457	88183	32070	30222
Unique reflections	9359	7200	4985	5676
R_{int}	0.0604	0.0726	0.0596	0.0192
Parameters / restraints	522 / 0	481 / 0	486 / 729	341 / 0
GooF on F^2	1.108	1.097	1.030	1.042
R_1 [I≥2σ(I)]	0.0609	0.0514	0.0587	0.0263
wR_2 [all data]	0.1822	0.1163	0.1722	0.0653
Max./min. residual electron density / e Å^{-3}	0.261 / –0.245	0.269 / –0.226	1.275 / –0.976	0.732 / –0.352

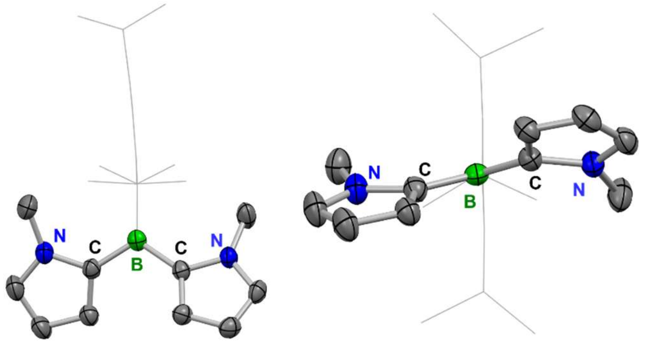

Figure S1. Molecular structures of **2** in the solid state from single-crystal X-ray diffraction at 100 K shown from above (left side) and along the B–C_{Tip} axis (right side). All ellipsoids are drawn at the 50 % probability level, (Tip groups and H atoms omitted for clarity).

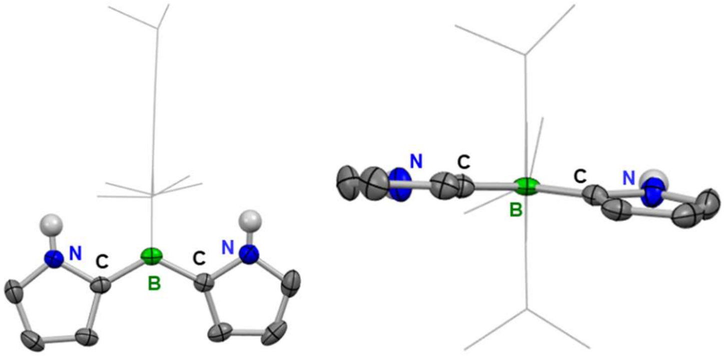

Figure S2. Molecular structures of **4** in the solid state from single-crystal X-ray diffraction at 100 K shown from above (left side) and along the B–C_{Tip} axis (right side). All ellipsoids are drawn at the 50 % probability level, (Tip groups and H atoms omitted for clarity, except for the N–H protons).

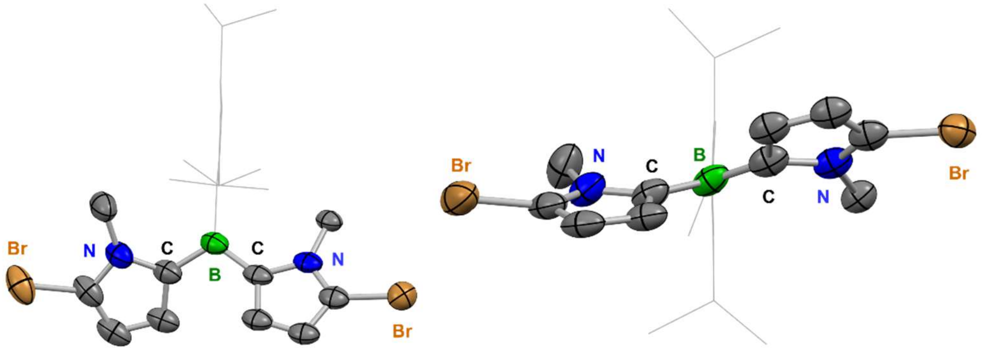

Figure S3. Molecular structure of **6** in the solid state determined by single-crystal X-ray diffraction at 100 K (ellipsoids drawn at the 50 % probability level; Tip group and H atoms omitted for clarity). Structures shown from above (left) and along the B–C_{Tip}-bond (right).

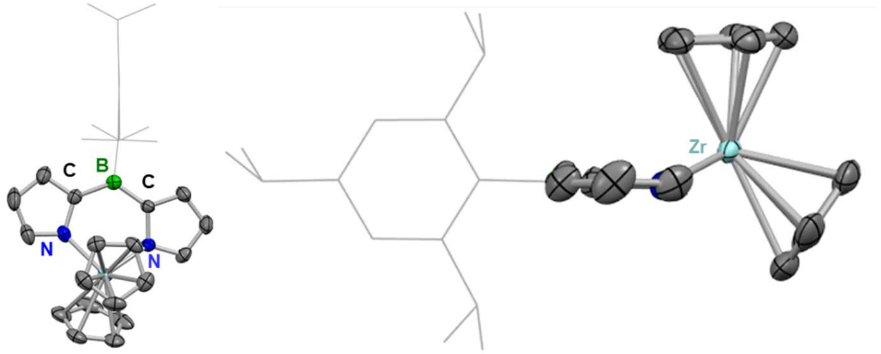

Figure S4. Molecular structure of **9** in the solid state from single-crystal X-ray diffraction at 100 K shown from above (left side) and side on (right side). All ellipsoids are drawn at the 50 % probability level, (Tip groups and H atoms omitted for clarity).

Table S2. Selected bond lengths (Å) and angles (°) in crystals of **2**, **4**, their thiophene and furan counterparts and **9** at 100 K. For pyrrole compounds **2** and **4** two symmetry-independent molecules are present each in the asymmetric unit of the unit cell, and the values for the second molecule are given in parentheses.

Bond lengths [Å] and angles [°]	2	4	TipBThi$_2$	TipBFur$_2$	6	9
Σ(∠(BR$_2$))	360.0(2) (360.0(2))	359.9(1) (360.0(1))	~360	~360	360.0	359.99
∠(Hetaryl-Tip)	89.57(7)/83.27(8) (89.51(6)/81.29(7))	88.54(7)/85.46(7) (91.12(6)/87.58(6))	86.0	85.5	88.4(5)/83.6(6)	78.37/79.87
∠(Hetaryl-BC$_3$)	28.81(8)/21.93(8) (26.16(8)/24.85(8))	4.82(7)/7.05(8) (8.11(8)/20.05(7))	30.4	5.1	26.9(8)/26.5(8)	3.02/2.78
B–C	1.537(2)/1.546(3) (1.541(3)/(1.543(2))	1.530(2)/1.536(2) (1.529(2)/(1.533(2))	1.532(3)/ 1.542(3)	1.523(4)/ 1.535(4)	1.538(9)/1.540(8)	1.530(3)/1.531(2)

1.4 NMR spectra

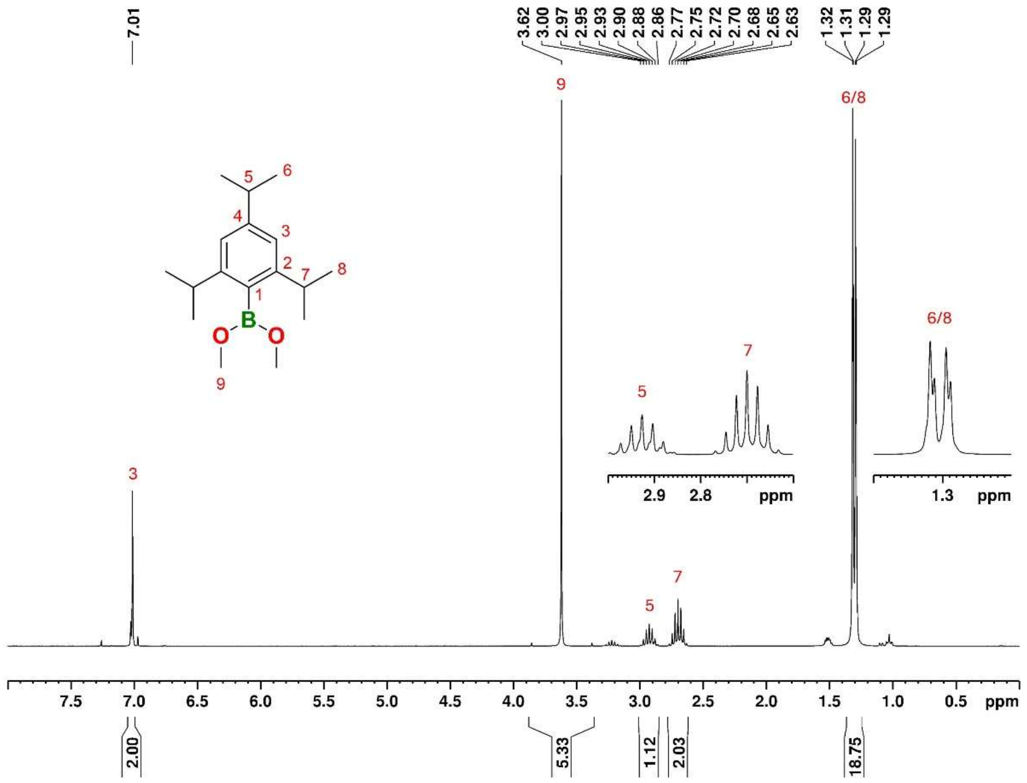

Figure S5. ^{1}H NMR spectrum of **TipB(OMe)₂** (300 MHz, in $CDCl_3$).

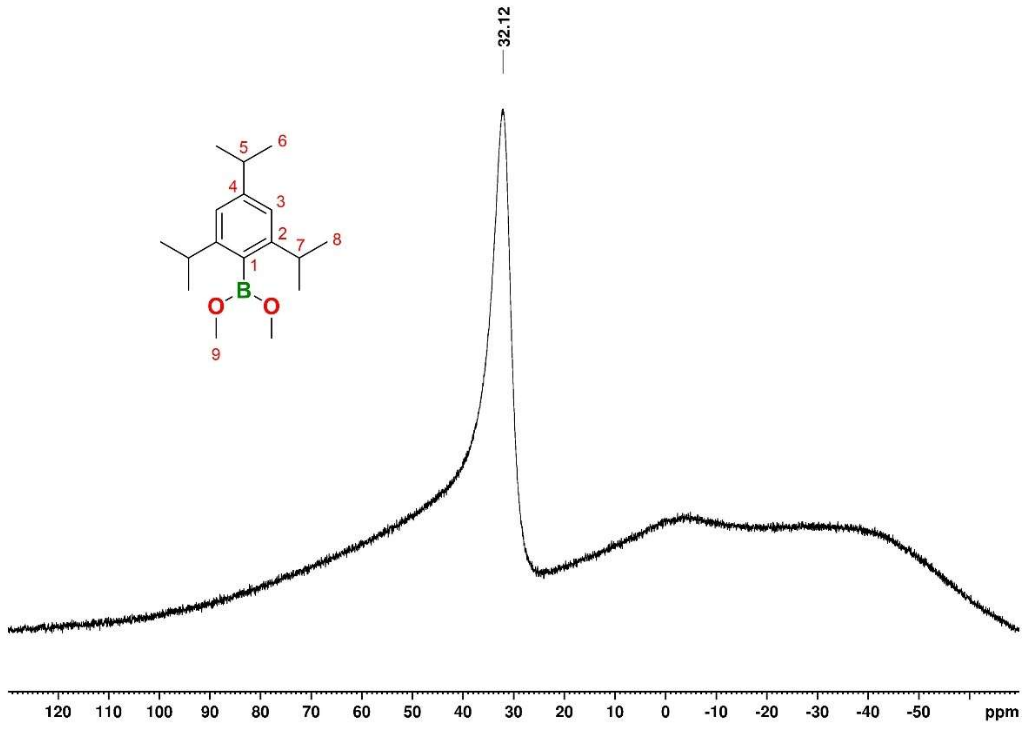

Figure S6. $^{11}B\{^{1}H\}$ NMR spectrum of **TipB(OMe)₂** (96 MHz, in $CDCl_3$).

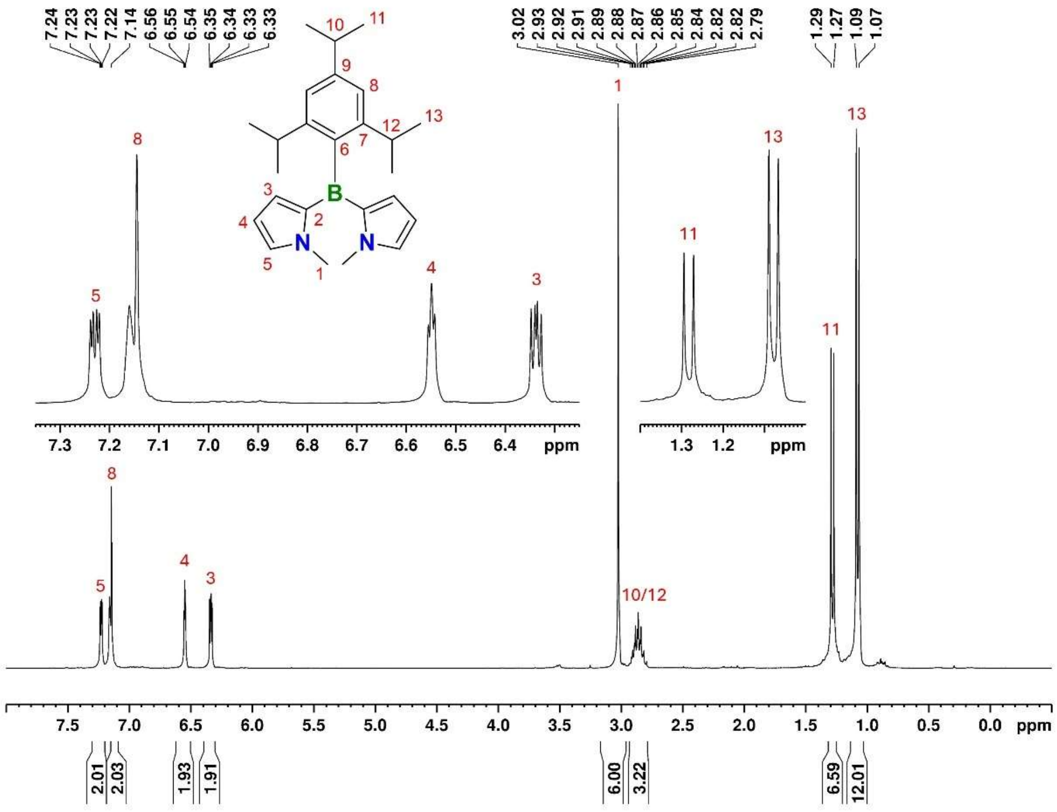

Figure S7. ^{1}H NMR spectrum of **2** (300 MHz, in C_6D_6).

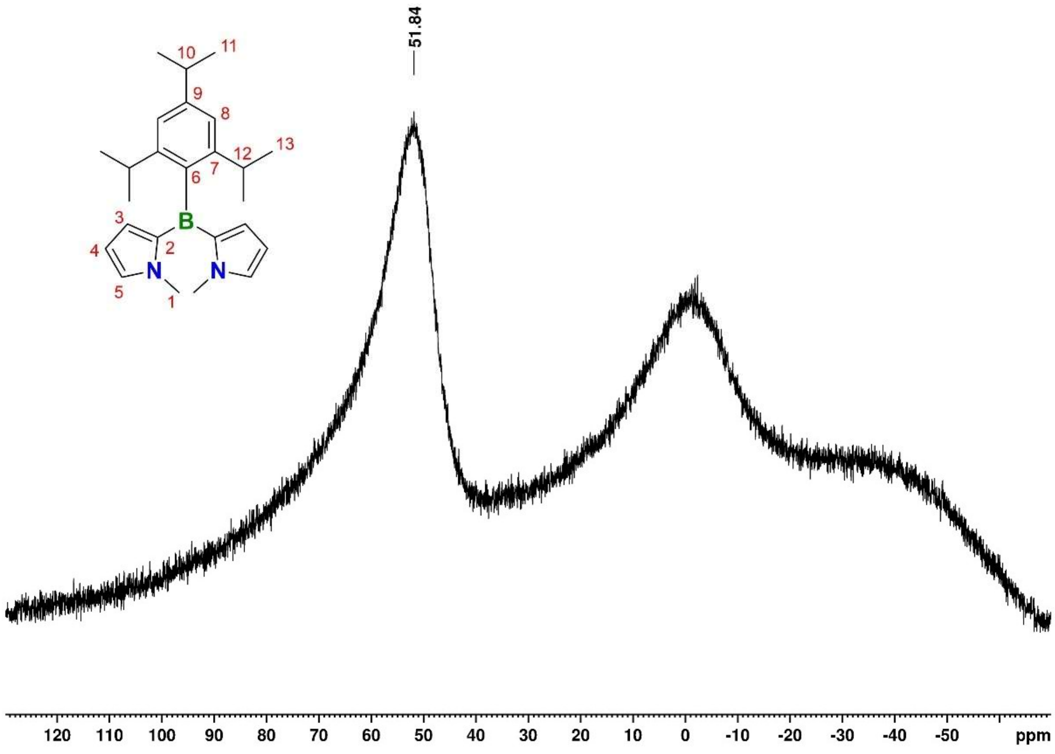

Figure S8. $^{11}B\{^{1}H\}$ NMR spectrum of **2** (96 MHz, in C_6D_6).

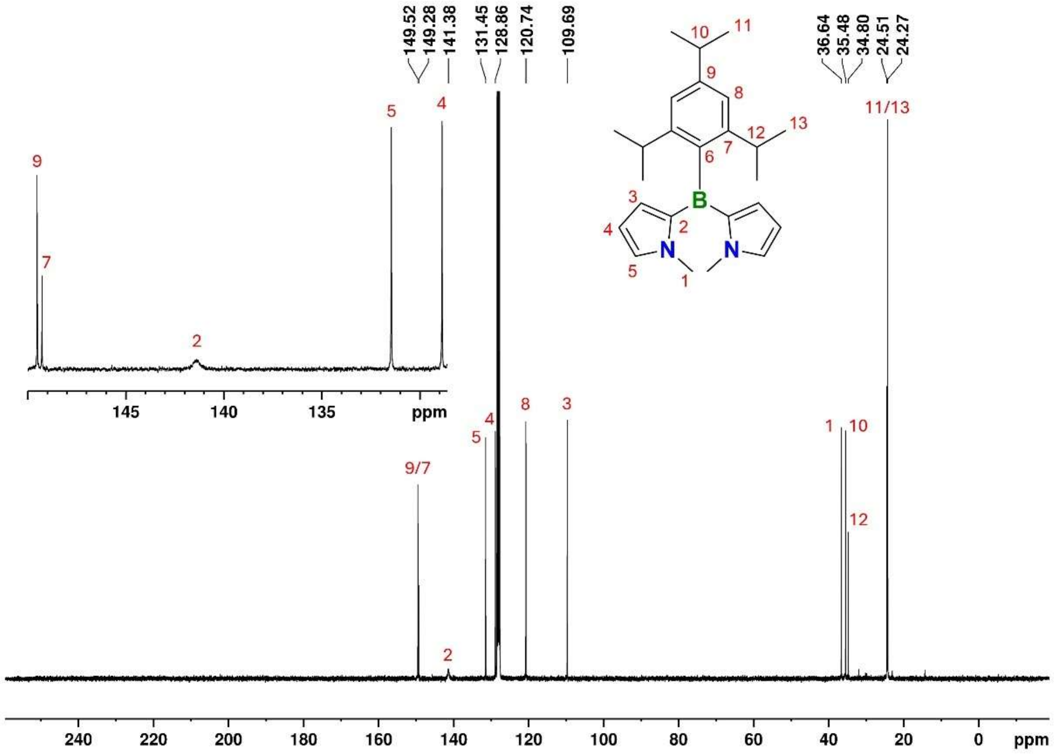

Figure S9. $^{13}C\{^1H\}$ NMR spectrum of **2** (75 MHz, in C_6D_6).

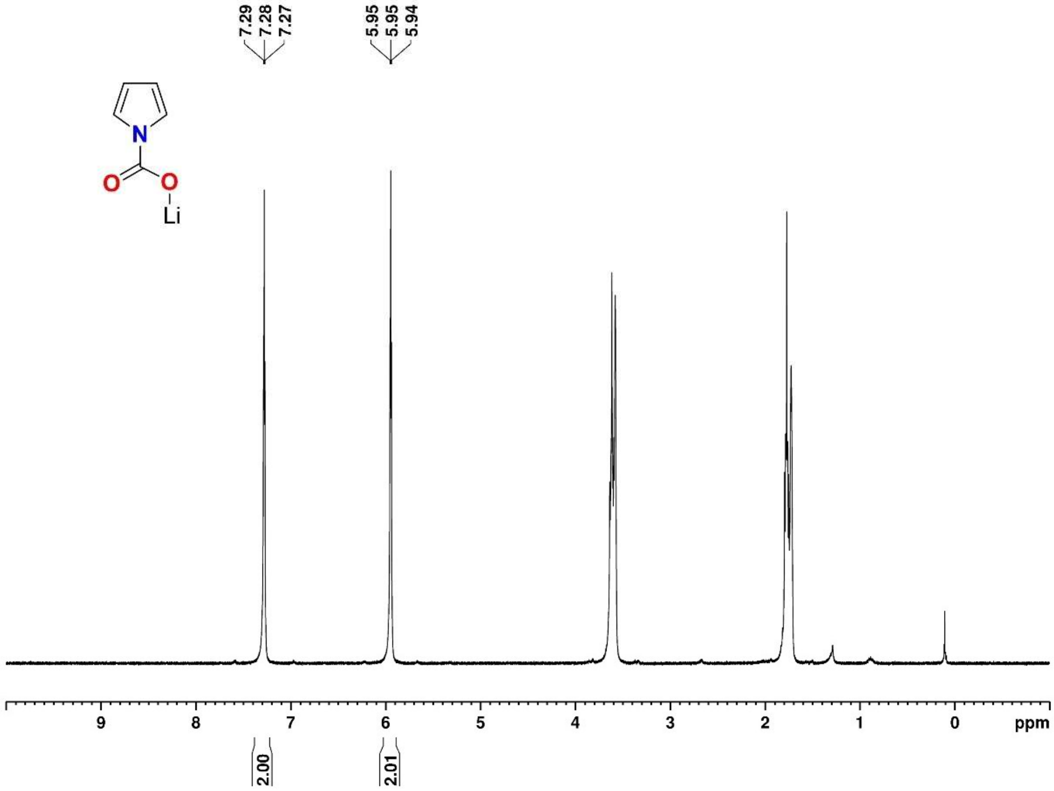

Figure S10. ^1H NMR spectrum of **3** (300 MHz, in THF-d_8).

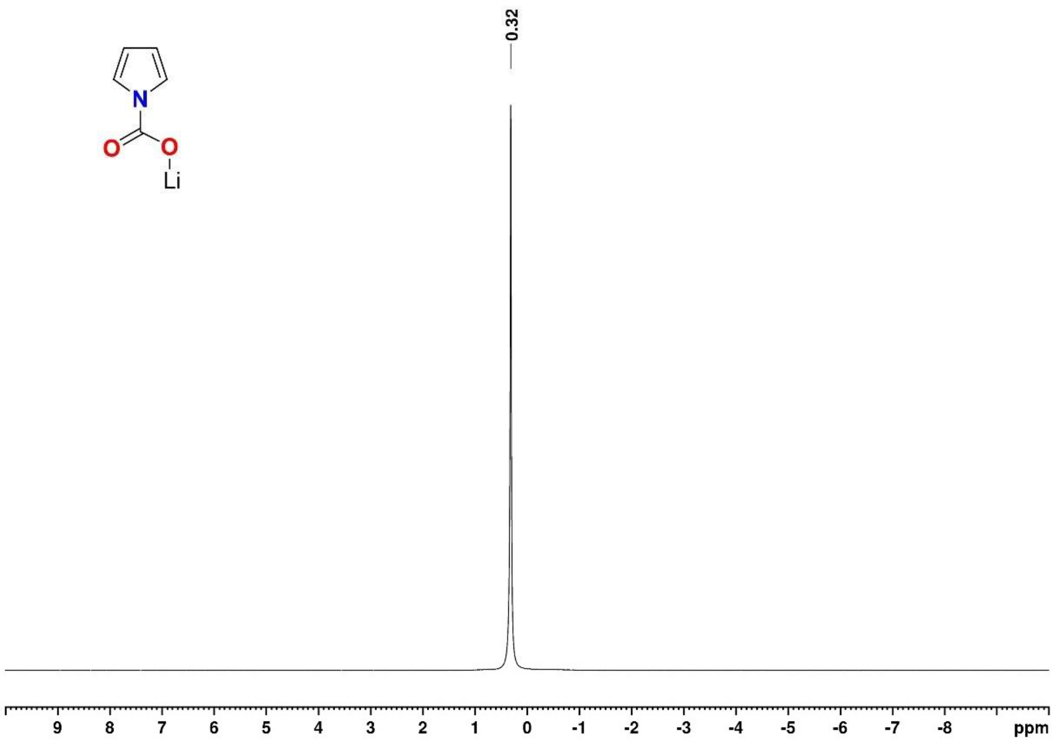

Figure S11. $^{7}Li\{^{1}H\}$ NMR spectrum of **3** (117 MHz, in THF-d_8).

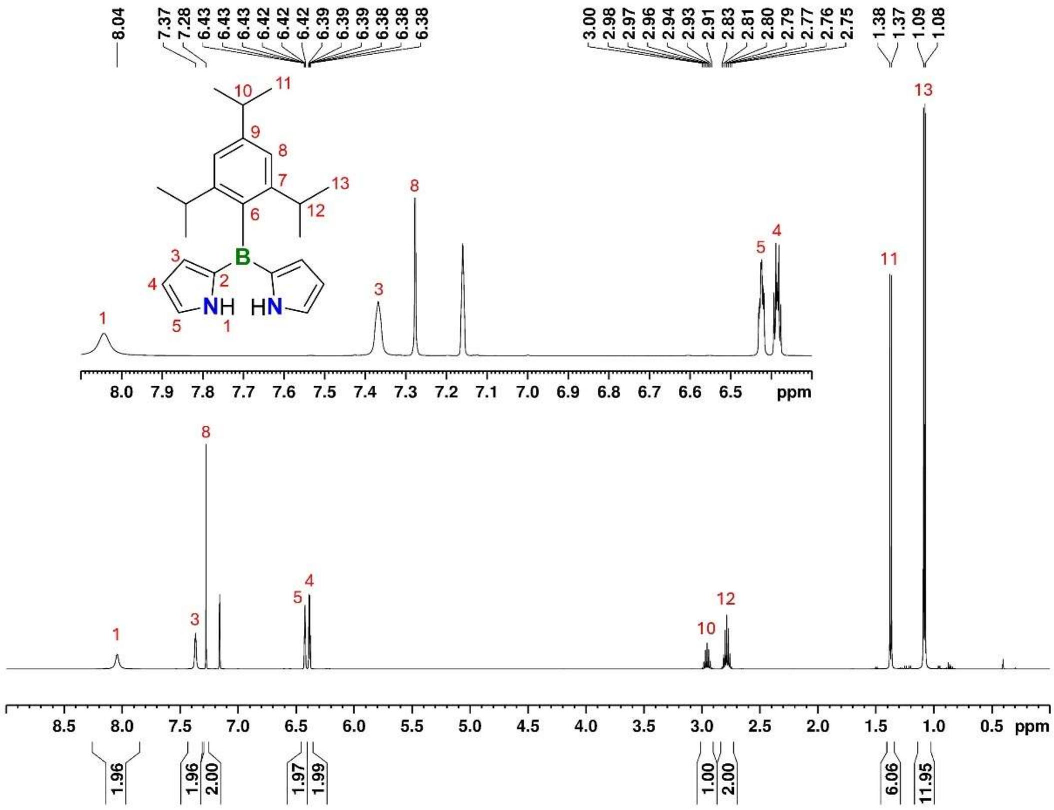

Figure S12. ^{1}H NMR spectrum of **4** (500 MHz, in C_6D_6).

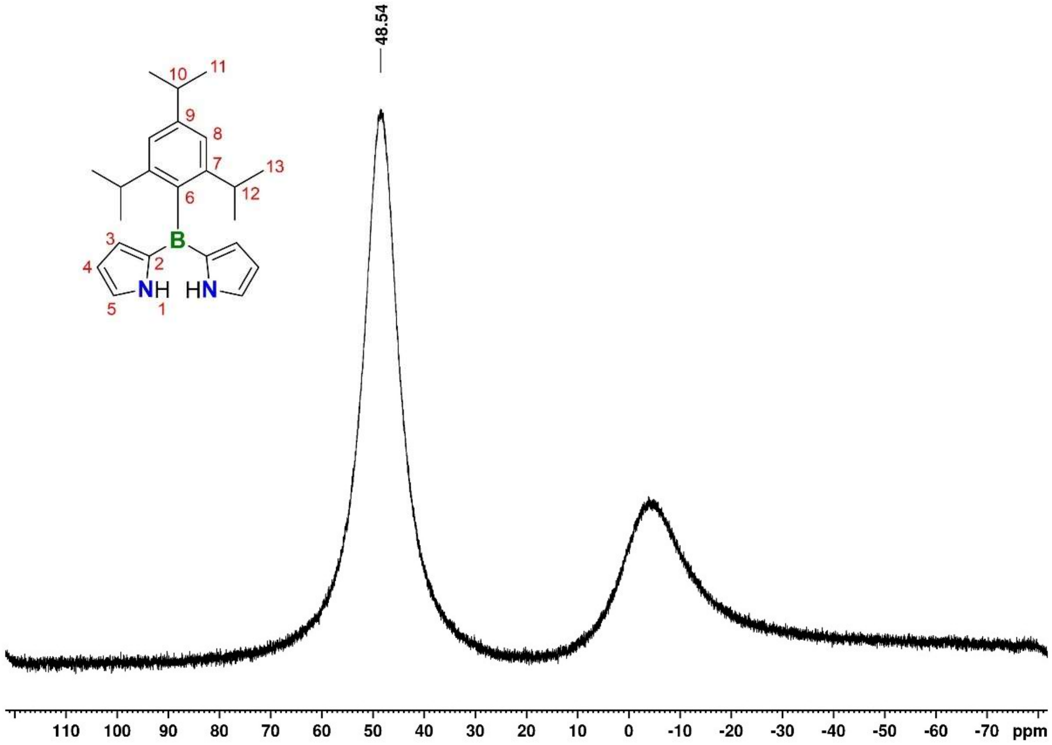

Figure S13. $^{11}B\{^{1}H\}$ NMR spectrum of **4** (160 MHz, in C_6D_6).

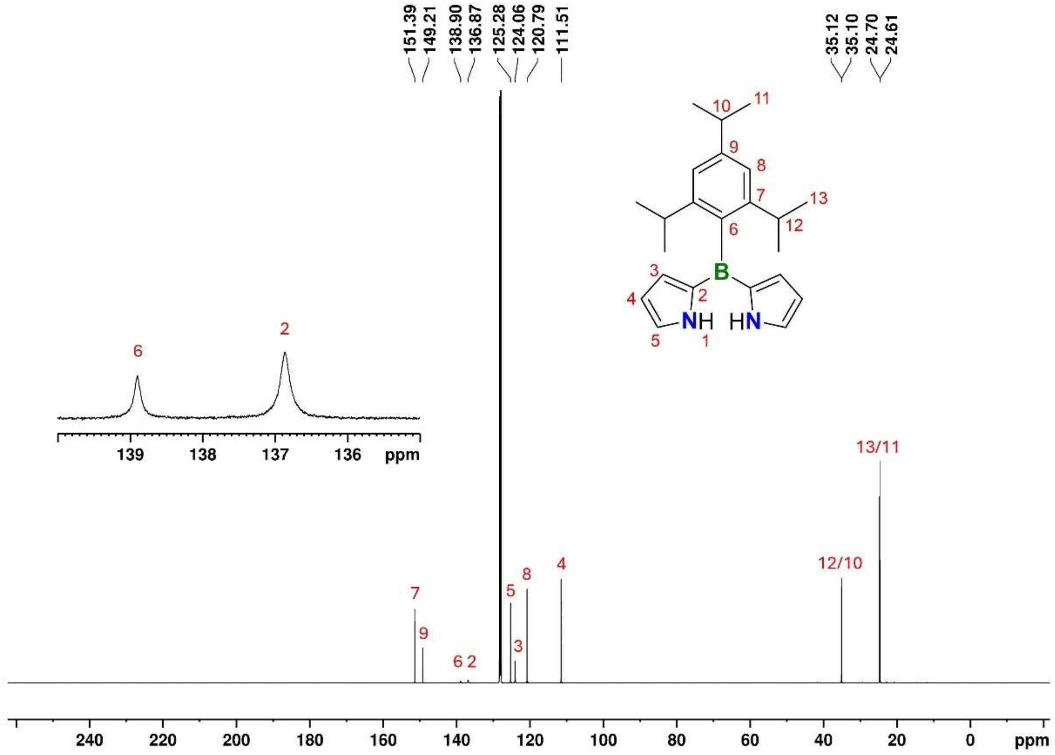

Figure S14. $^{13}C\{^{1}H\}$ NMR spectrum of **4** (126 MHz, in C_6D_6).

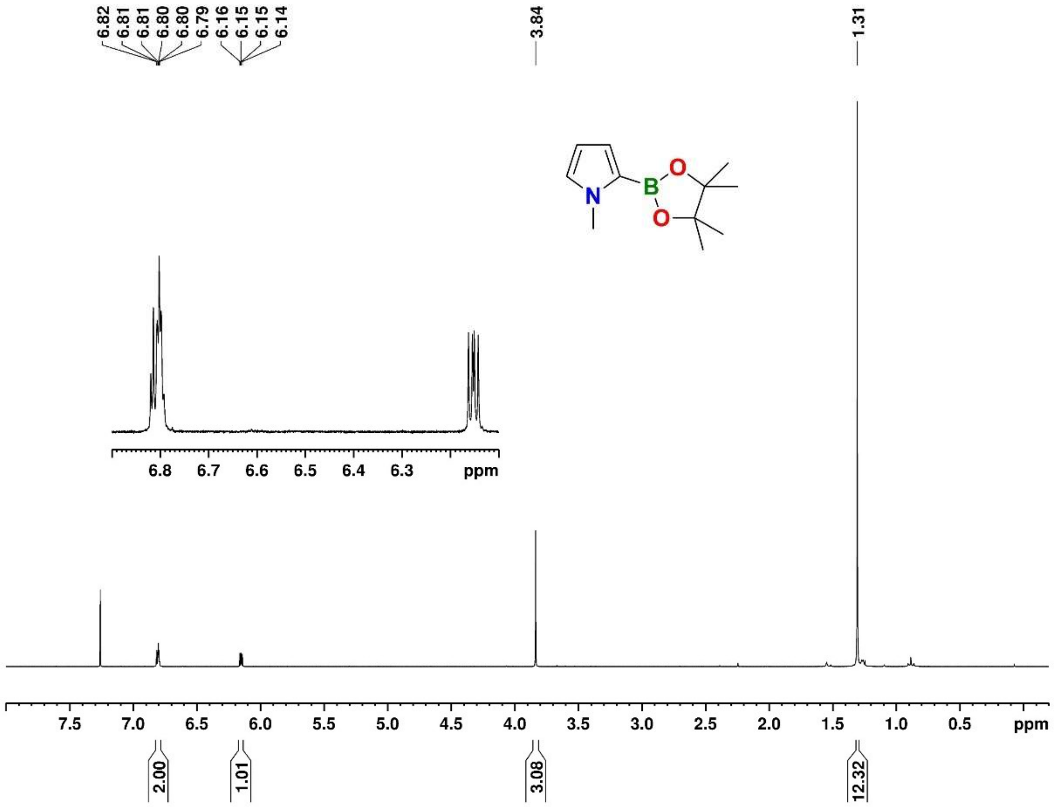

Figure S15. ^{1}H NMR spectrum of **5** (300 MHz, in $CDCl_3$).

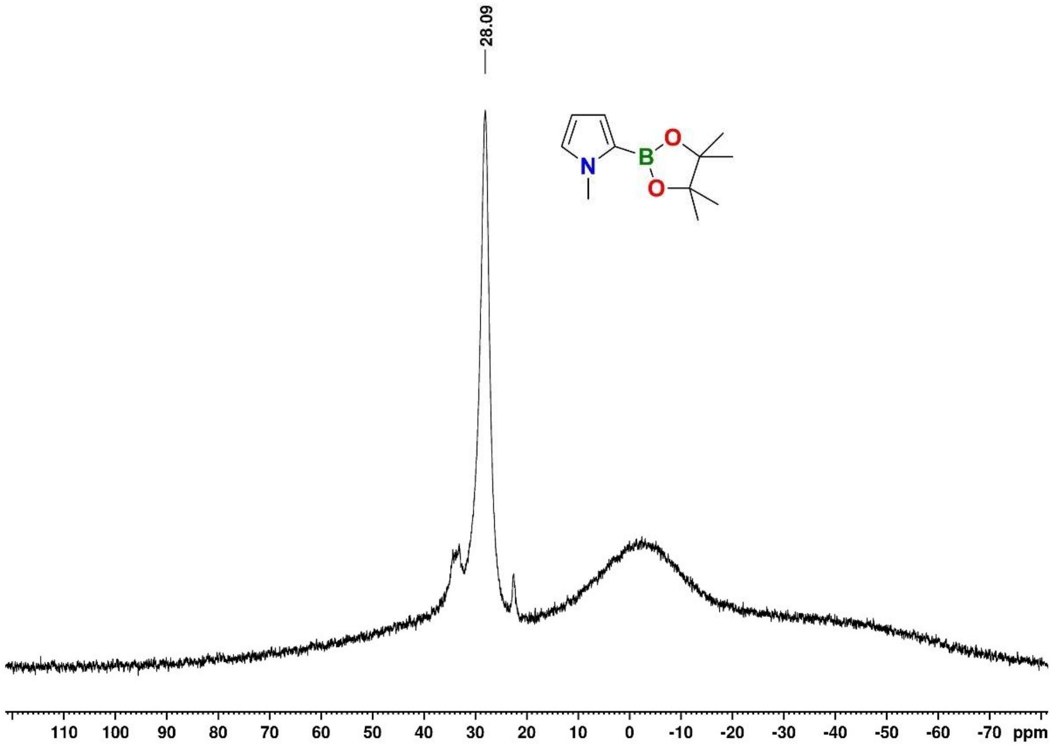

Figure S16. $^{11}B\{^{1}H\}$ NMR spectrum of **5** (96 MHz, in $CDCl_3$).

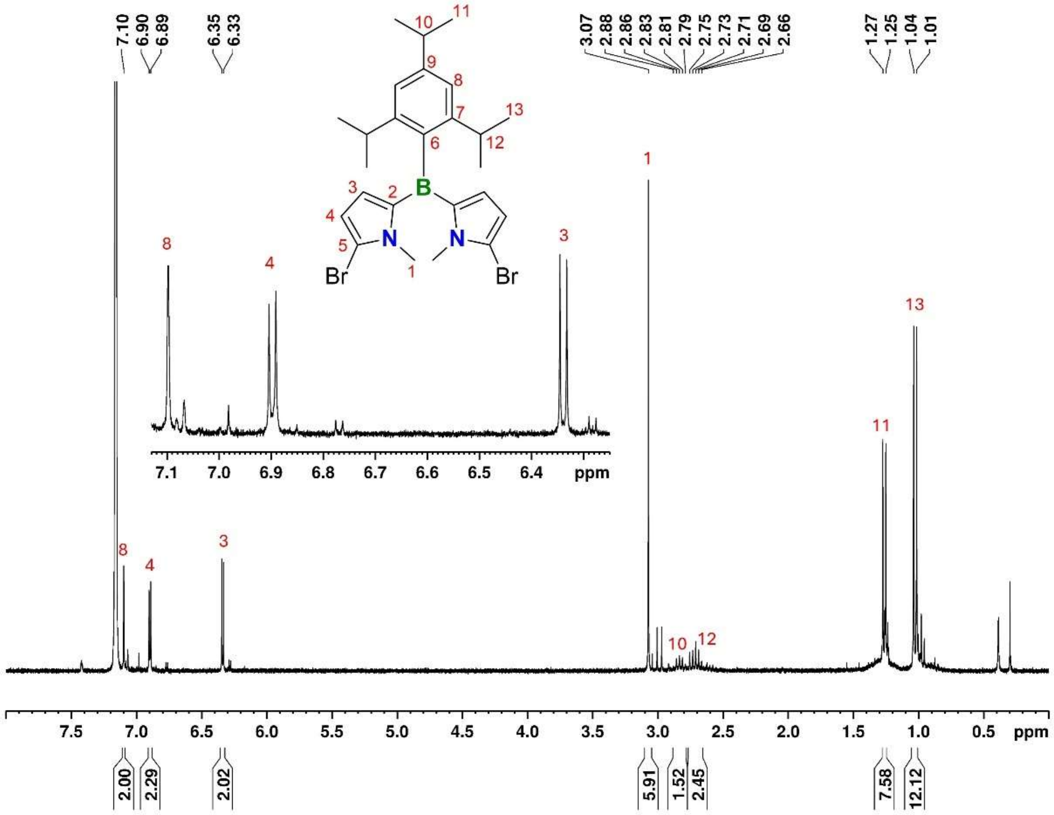

Figure S17. ^{1}H NMR spectrum of **6** (300 MHz, in C_6D_6).

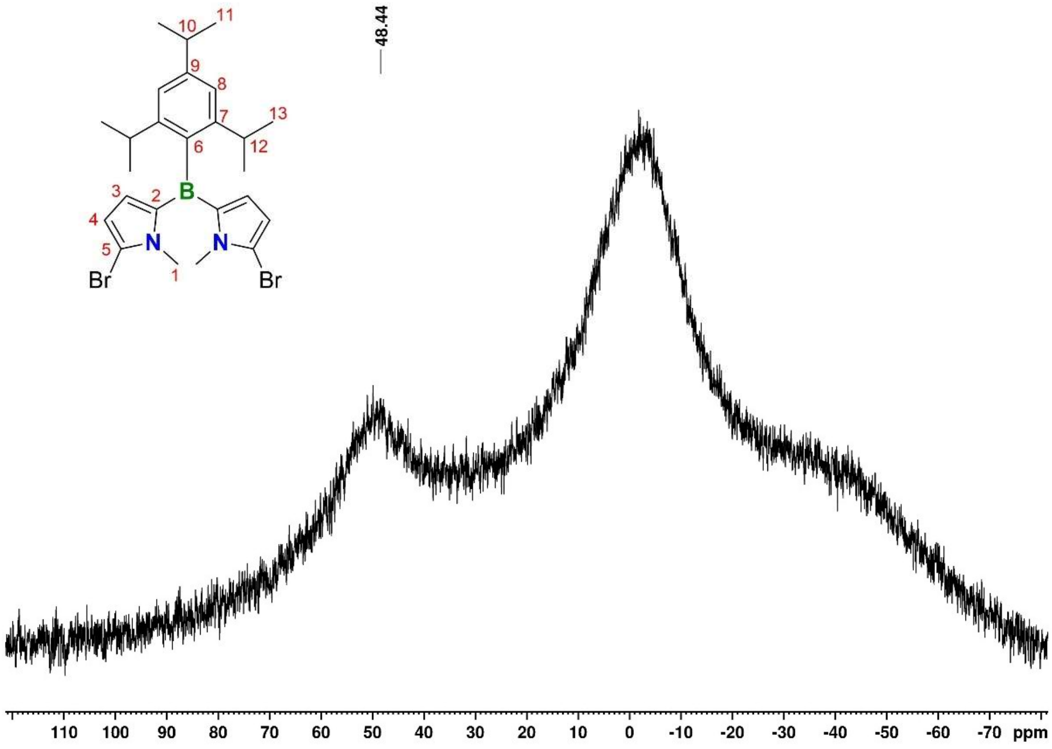

Figure S18. ^{11}B{^{1}H} NMR spectrum of **6** (96 MHz, in C_6D_6).

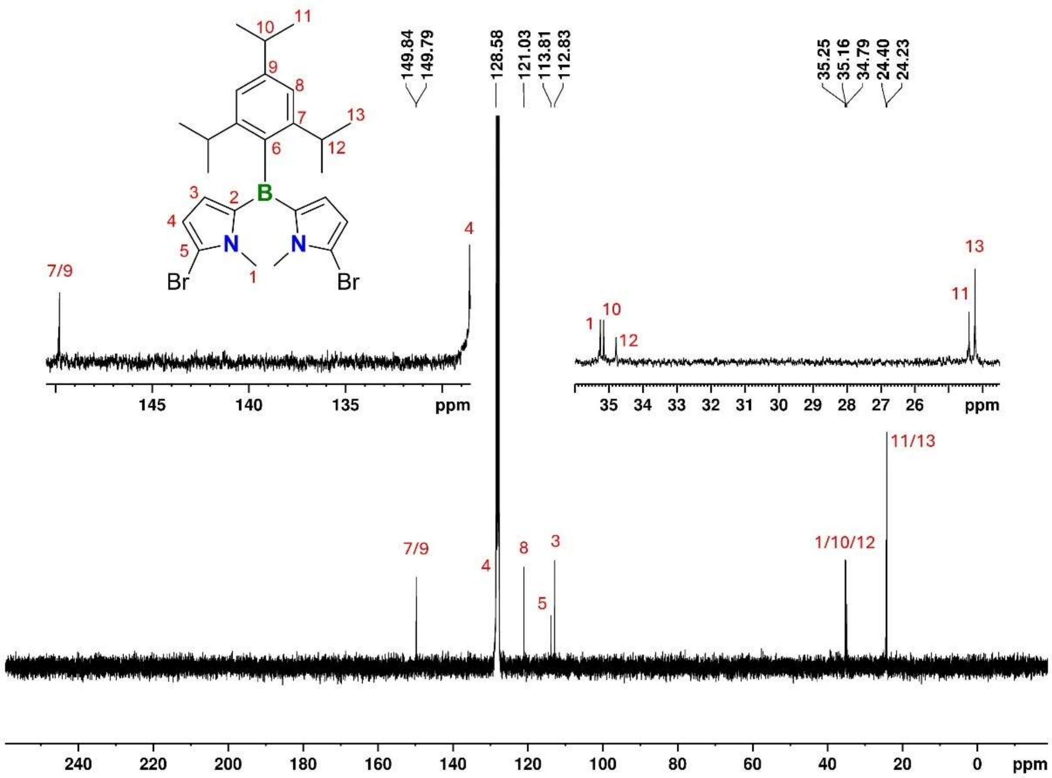

Figure S19. $^{13}C\{^1H\}$ NMR spectrum of **6** (75 MHz, in C_6D_6).

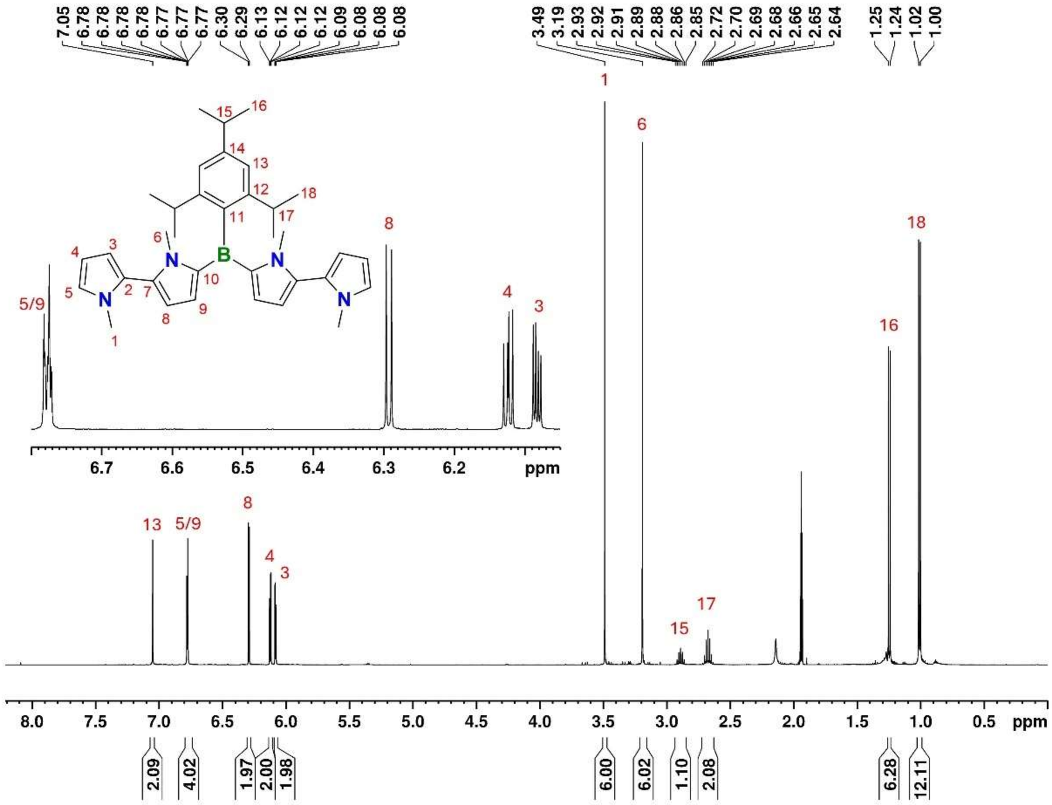

Figure S20. ^1H NMR spectrum of **7a** (500 MHz, in CD_3CN).

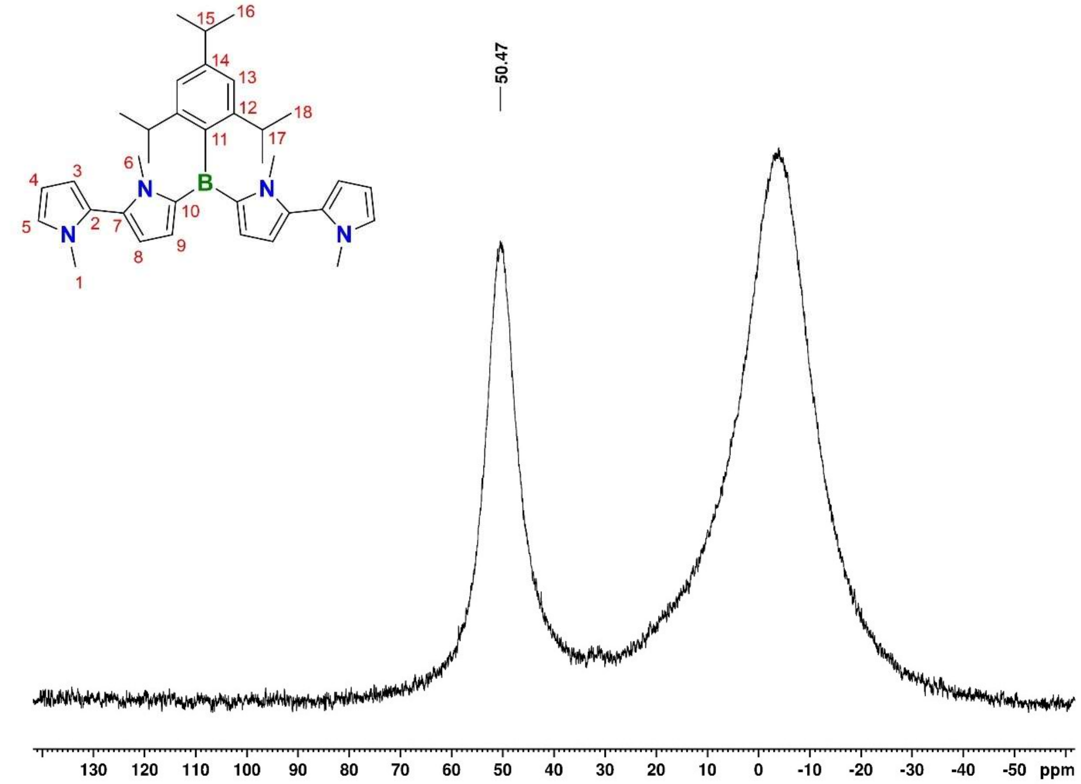

Figure S21. $^{11}B\{^1H\}$ NMR spectrum of **7a** (160 MHz, in CD_3CN).

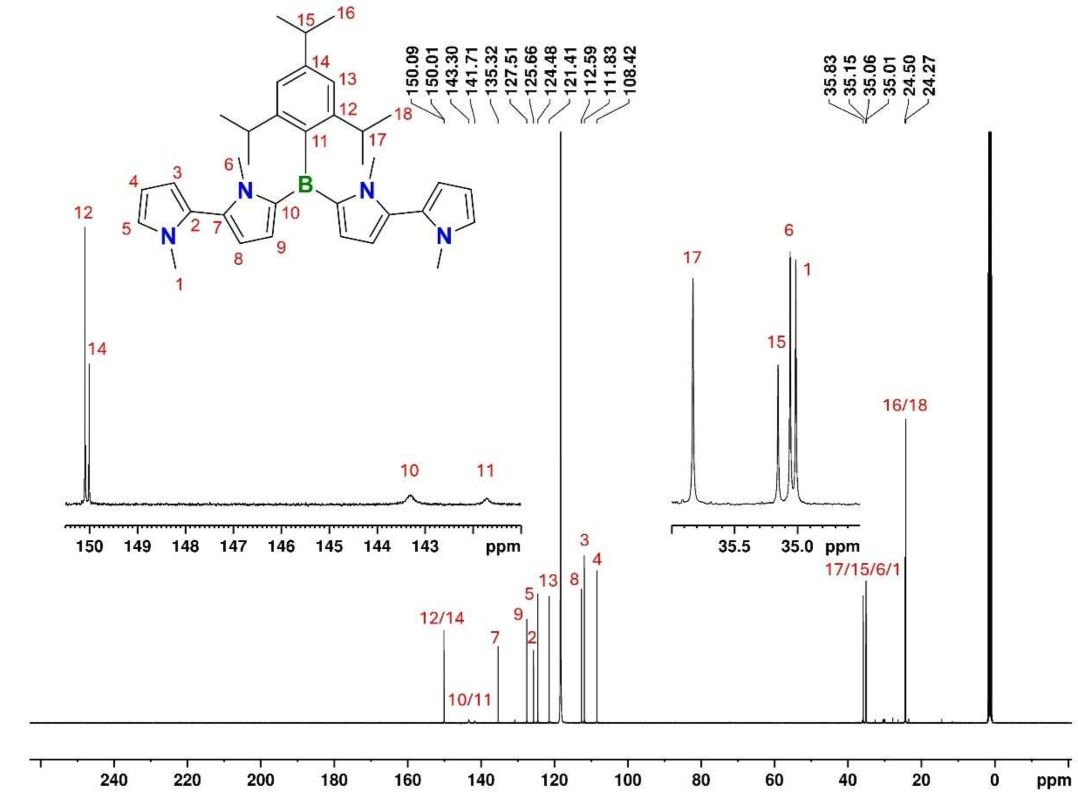

Figure S22. $^{13}C\{^1H\}$ NMR spectrum of **7a** (126 MHz, in CD_3CN).

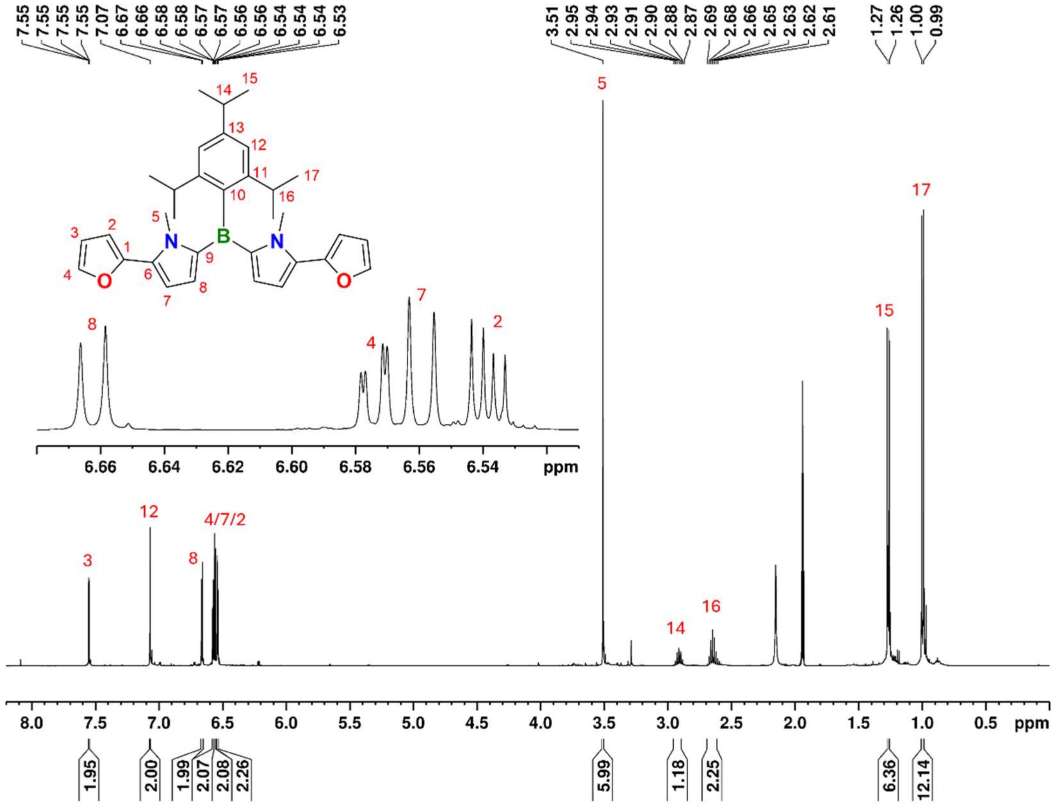

Figure S23. ^{1}H NMR spectrum of **7b** (500 MHz, in CD_3CN).

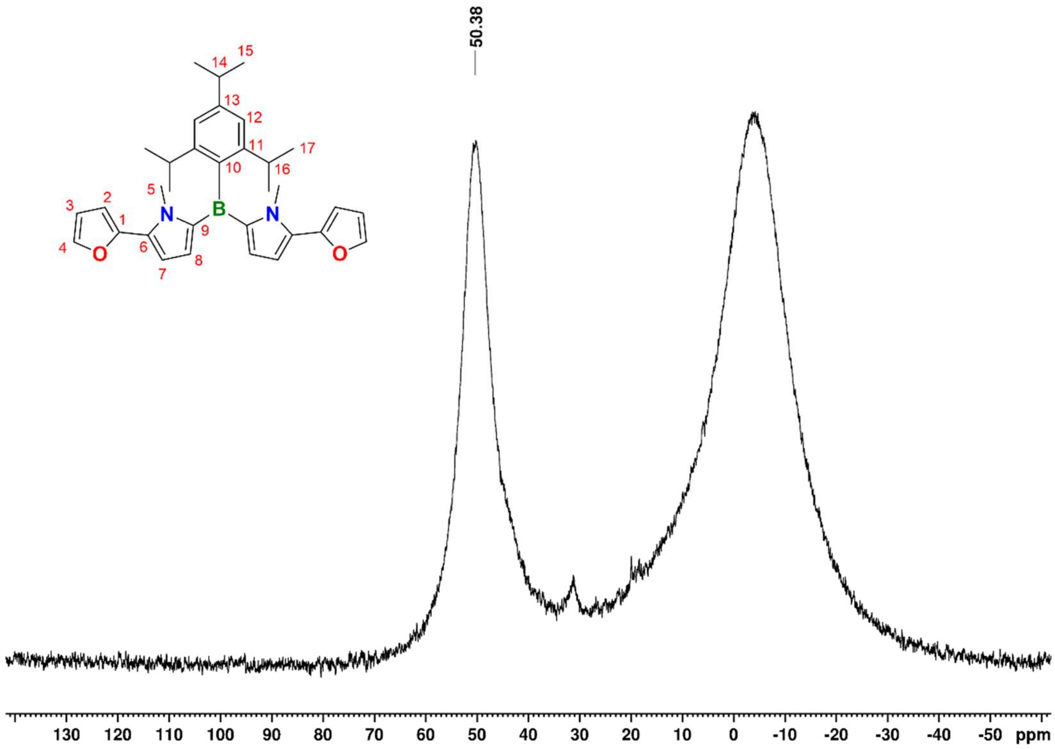

Figure S24. $^{11}B\{^{1}H\}$ NMR spectrum of **7b** (160 MHz, in CD_3CN).

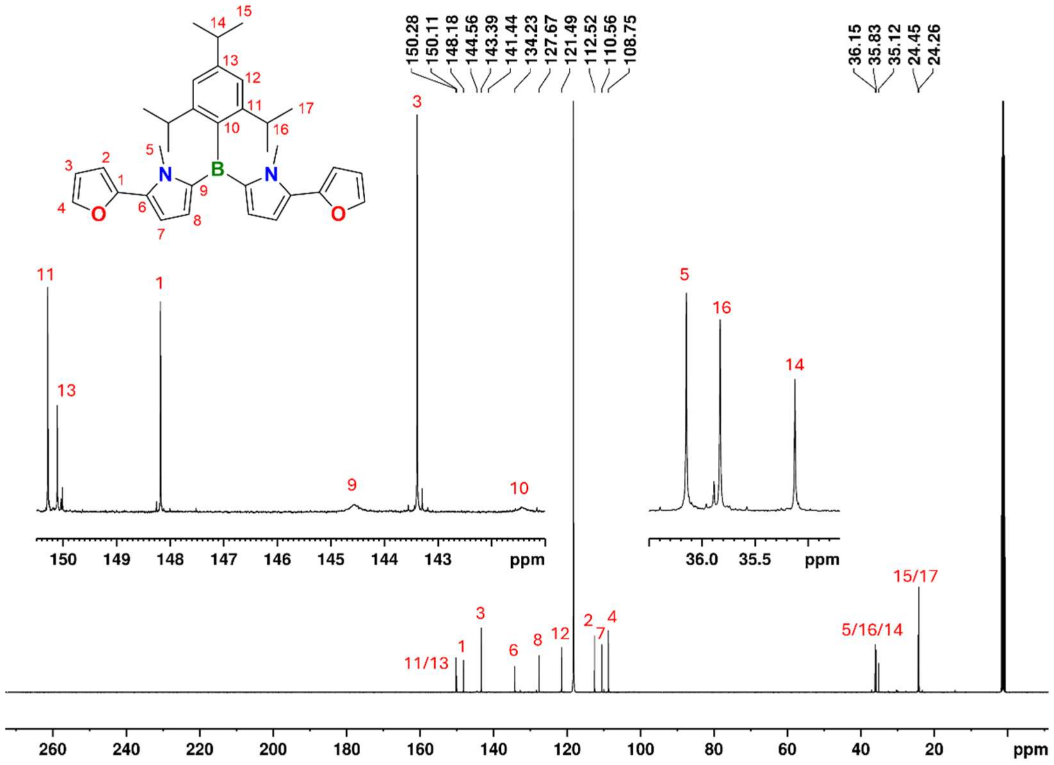

Figure S25. $^{13}C\{^{1}H\}$ NMR spectrum of **7b** (126 MHz, in CD_3CN).

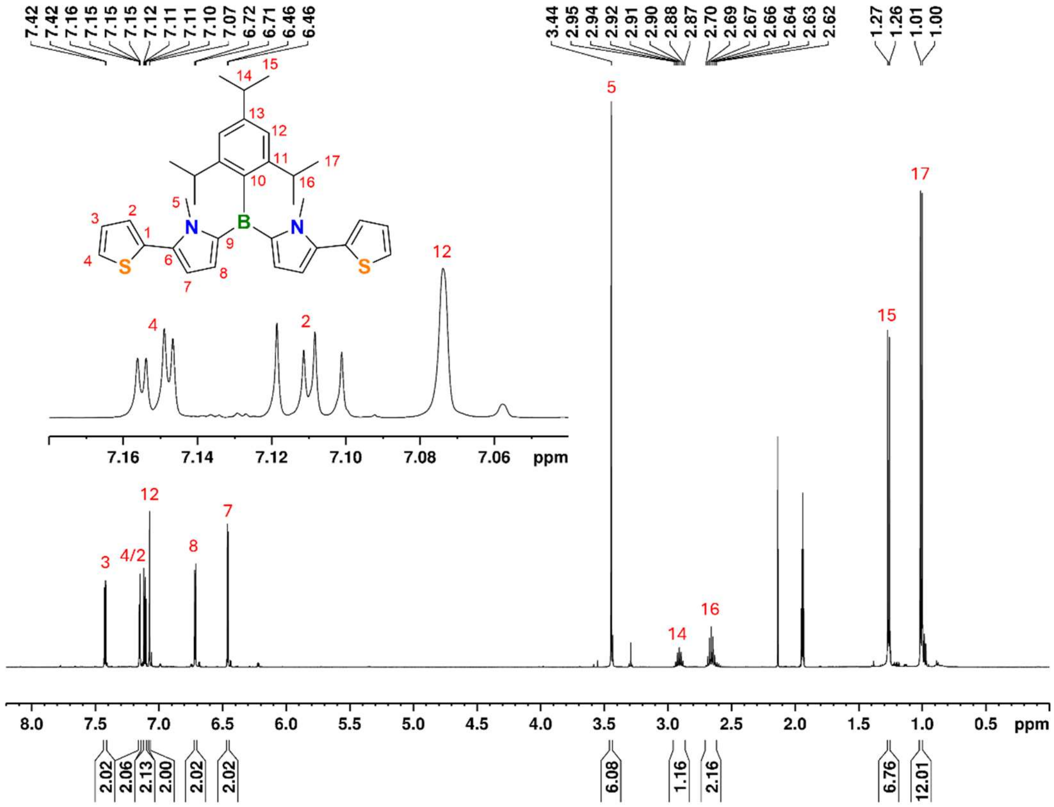

Figure S26. ^{1}H NMR spectrum of **7c** (500 MHz, in CD_3CN).

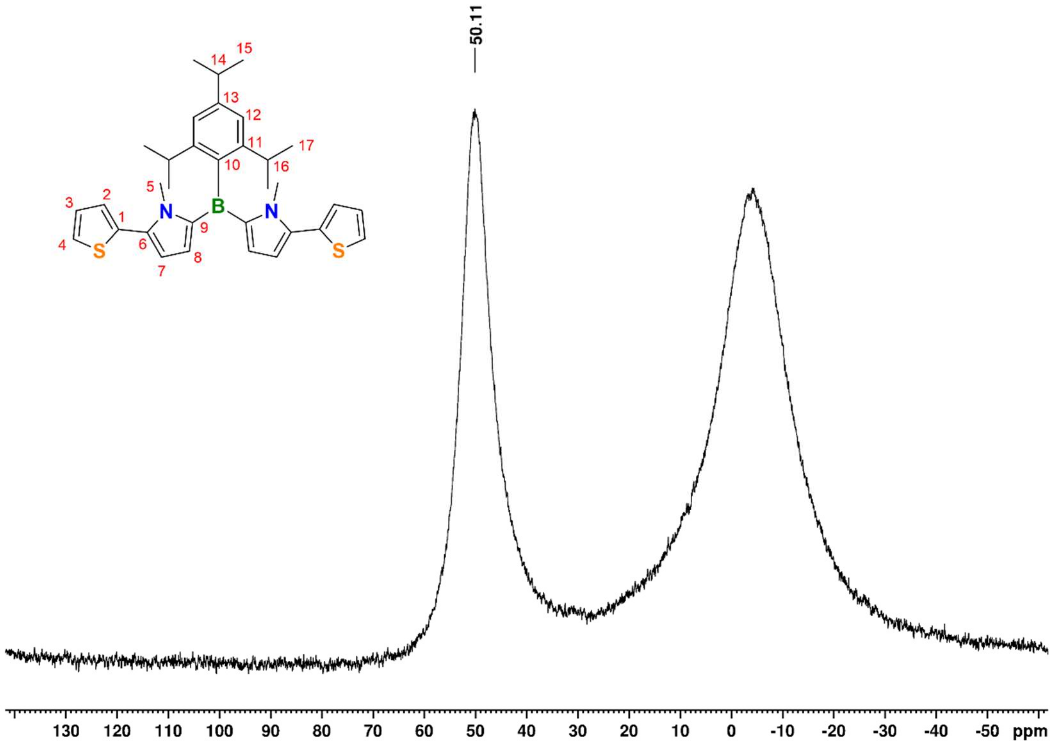

Figure S27. $^{11}B\{^{1}H\}$ NMR spectrum of **7c** (160 MHz, in CD_3CN).

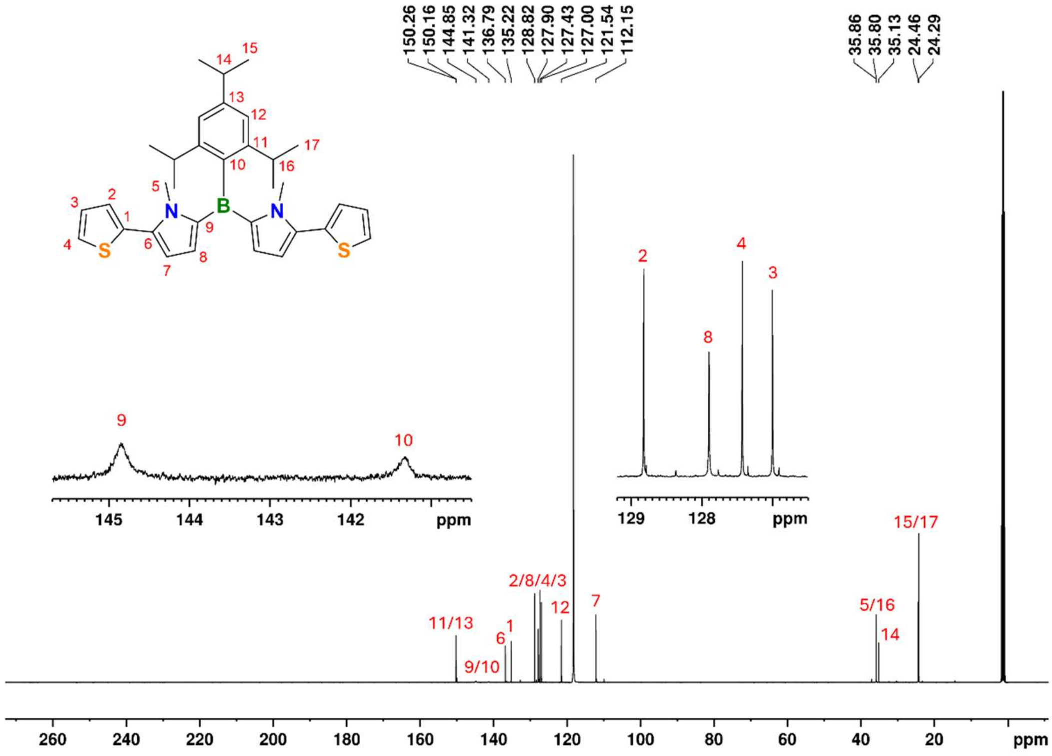

Figure S28. $^{13}C\{^{1}H\}$ NMR spectrum of **7c** (126 MHz, in CD_3CN).

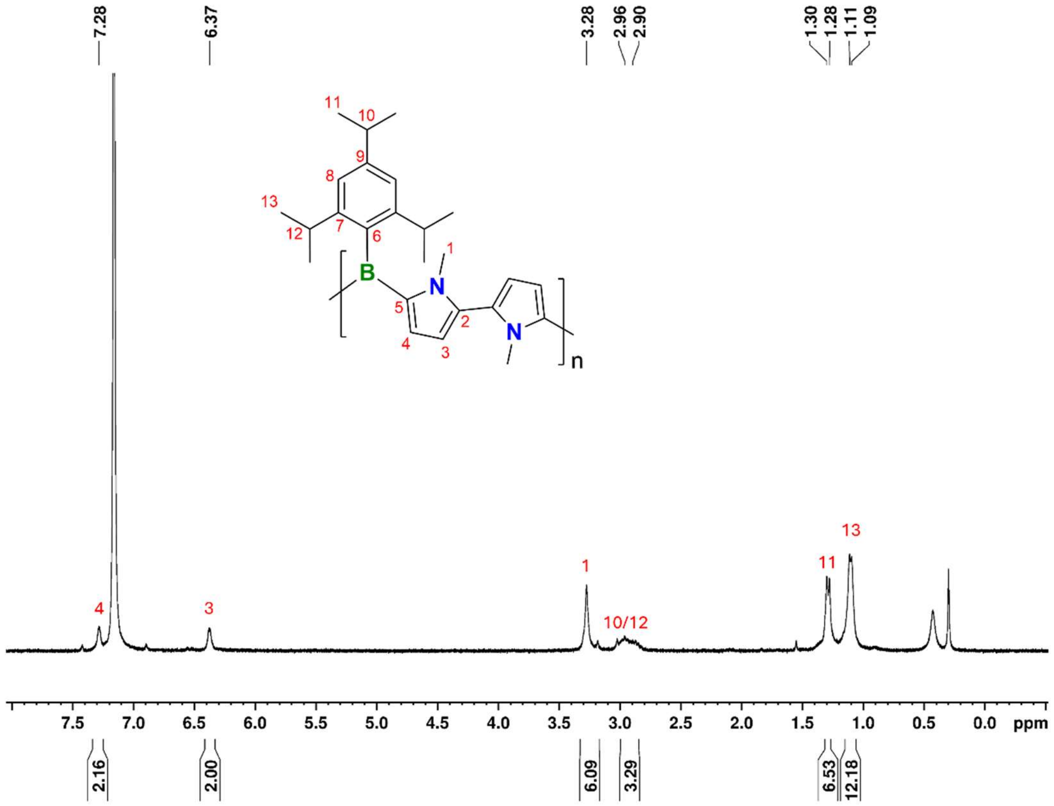

Figure S29. ^{1}H NMR spectrum of **8** (300 MHz, in C_6D_6).

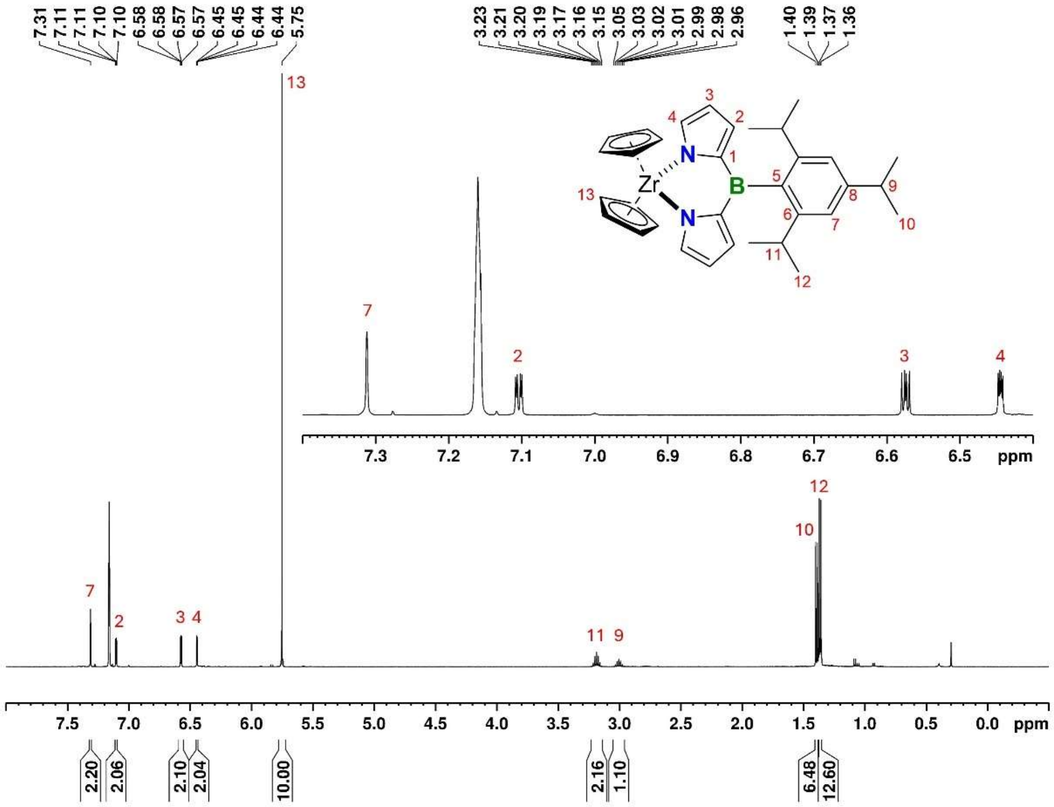

Figure S30. ^{1}H NMR spectrum of **9** (500 MHz, in C_6D_6).

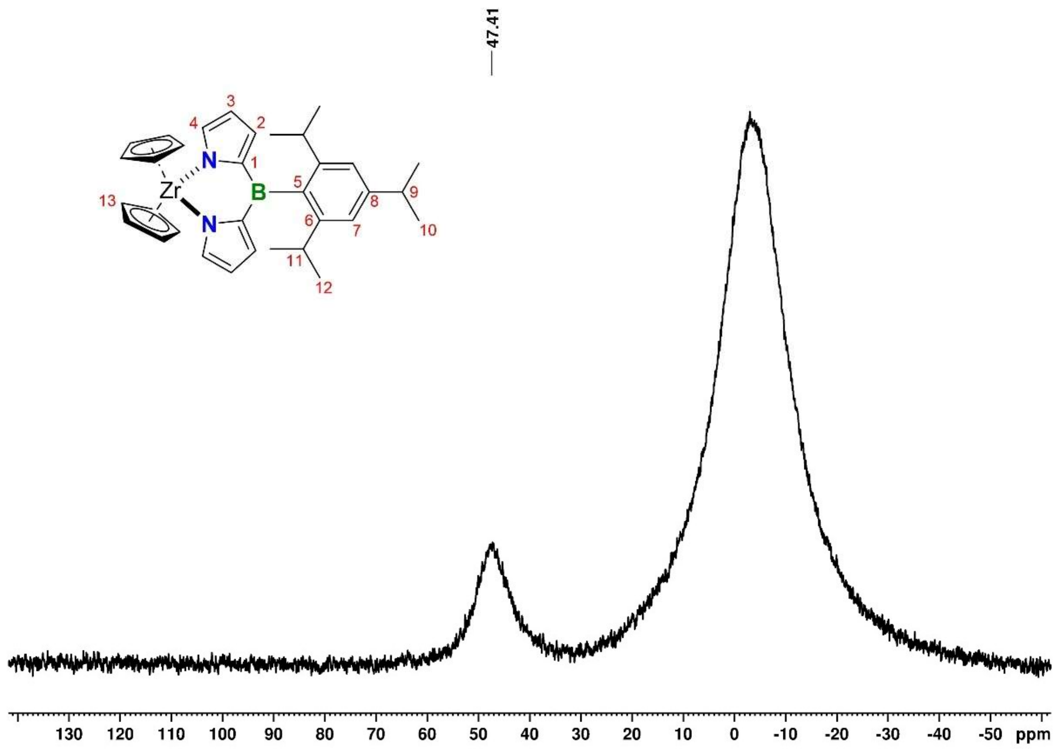

Figure S31. $^{11}B\{^1H\}$ NMR spectrum of **9** (160 MHz, in C_6D_6).

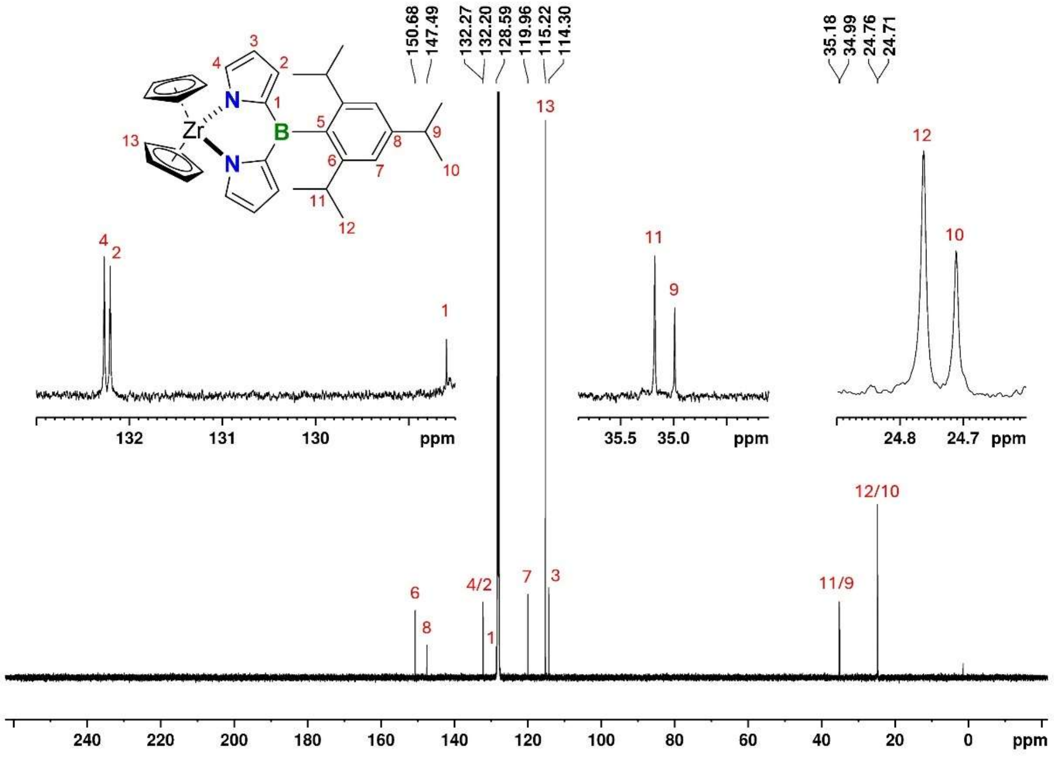

Figure S32. $^{13}C\{^1H\}$ NMR spectrum of **9** (126 MHz, in C_6D_6).

1.5 High resolution mass spectra

The mass spectra were processed using the Qual Browser of the XCalibur software. The figures show the total spectrum in the upper part, the product peak with isotope distribution in the middle and a corresponding simulation in the lower part.

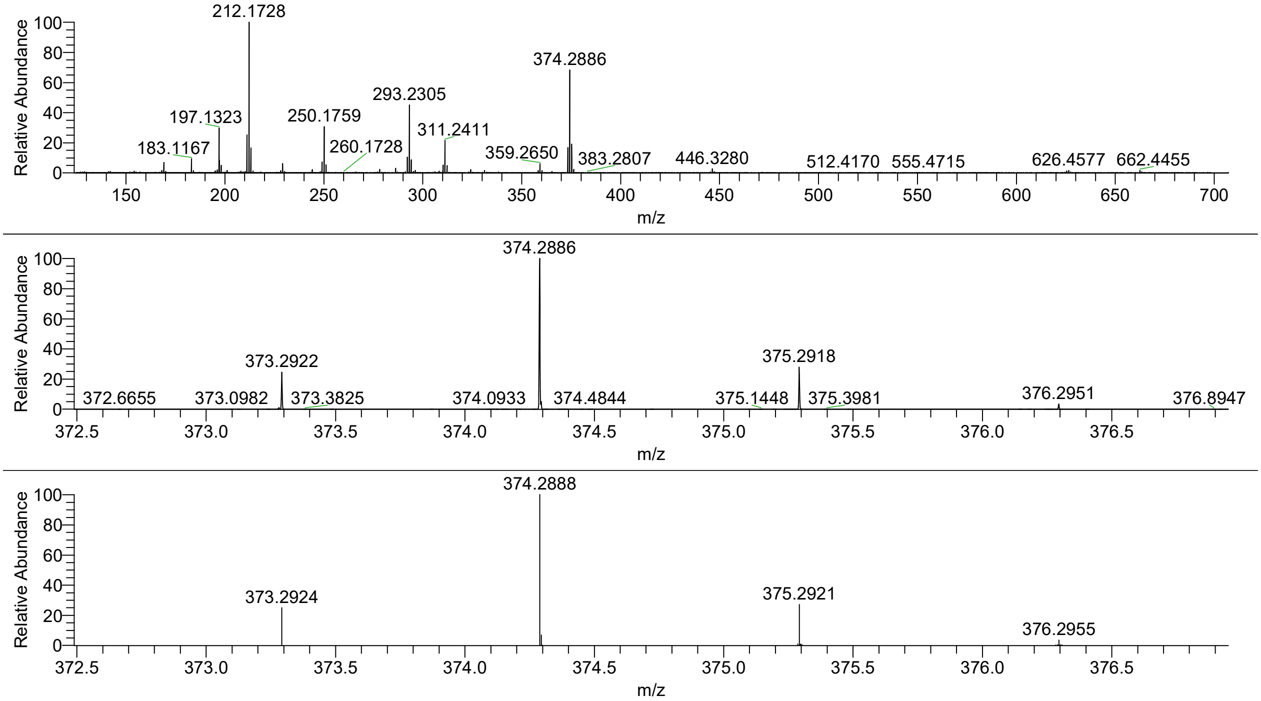

Figure S33: HRMS spectrum (LIFDI) of **2**.

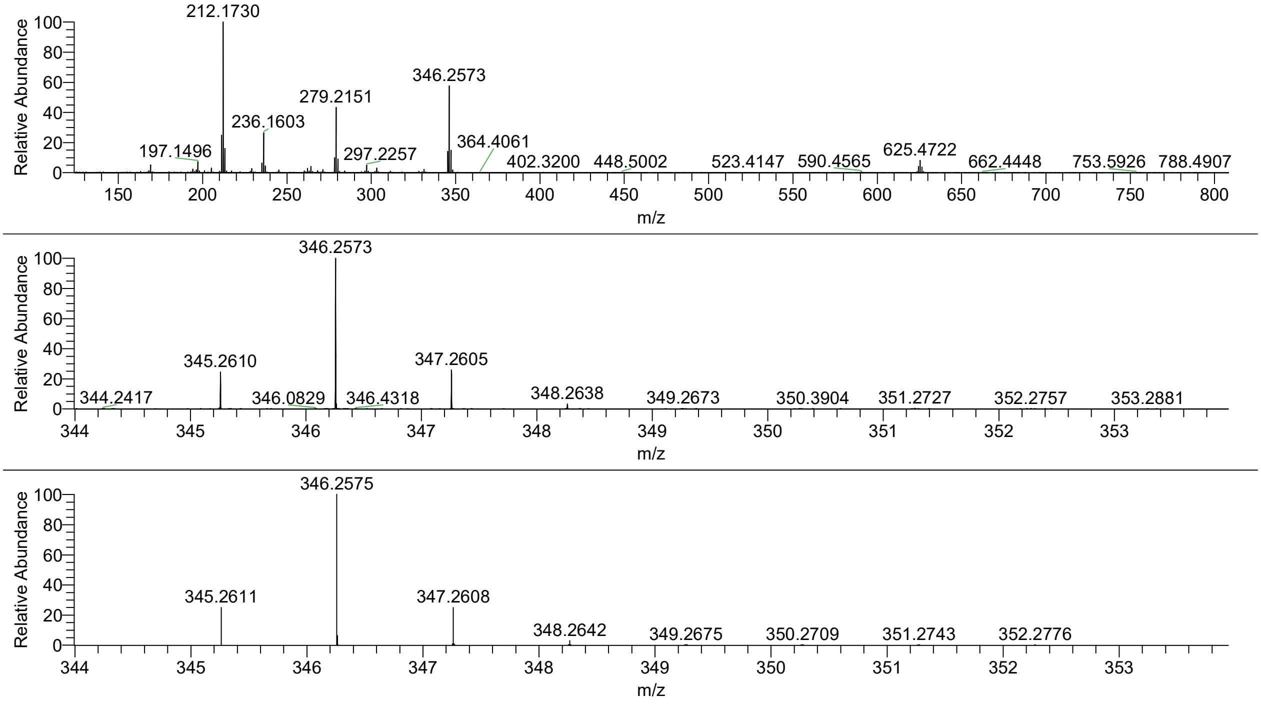

Figure S34: HRMS spectrum (LIFDI) of **4**.

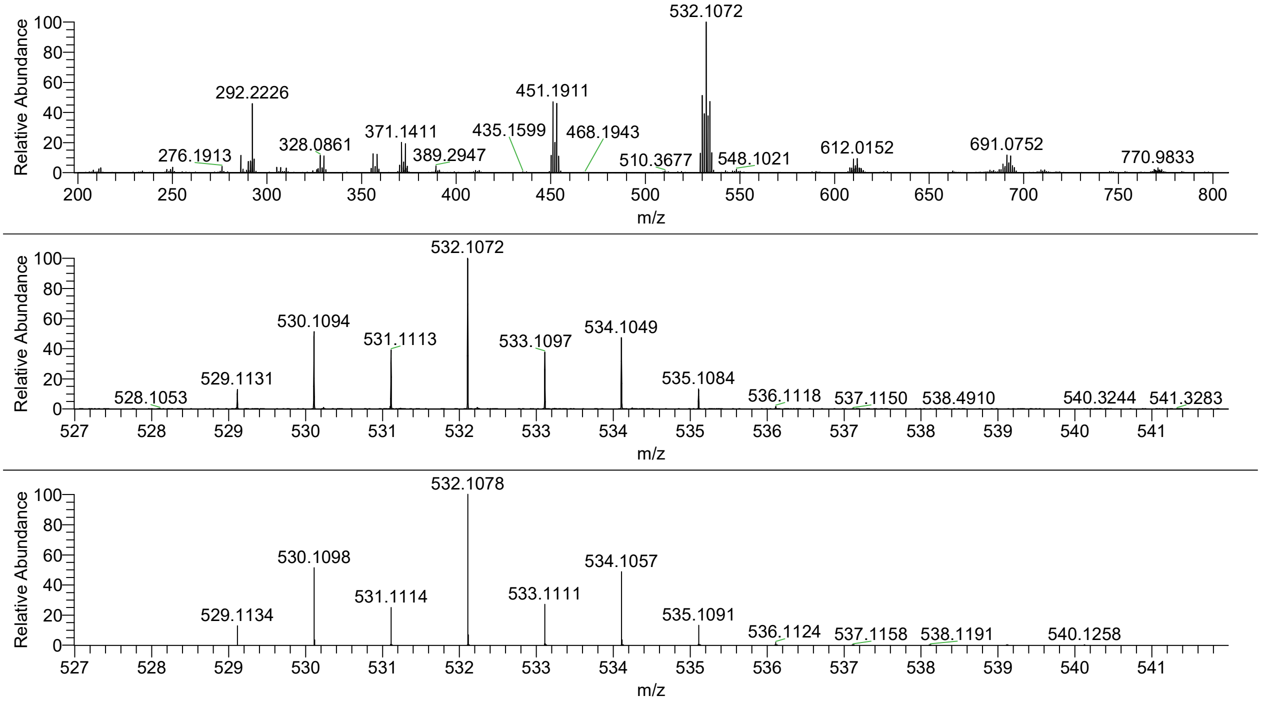

Figure S35: HRMS spectrum (LIFDI) of **6**.

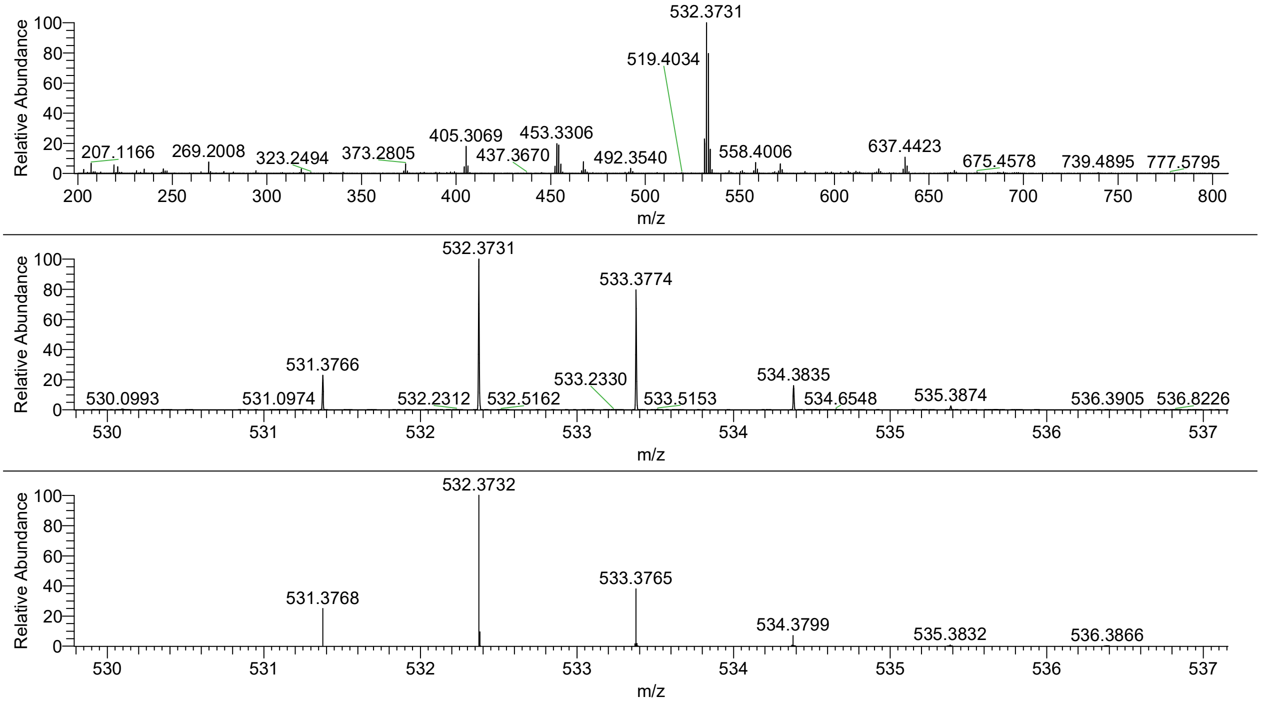

Figure S36: HRMS spectrum (APCI pos) of **7a**.

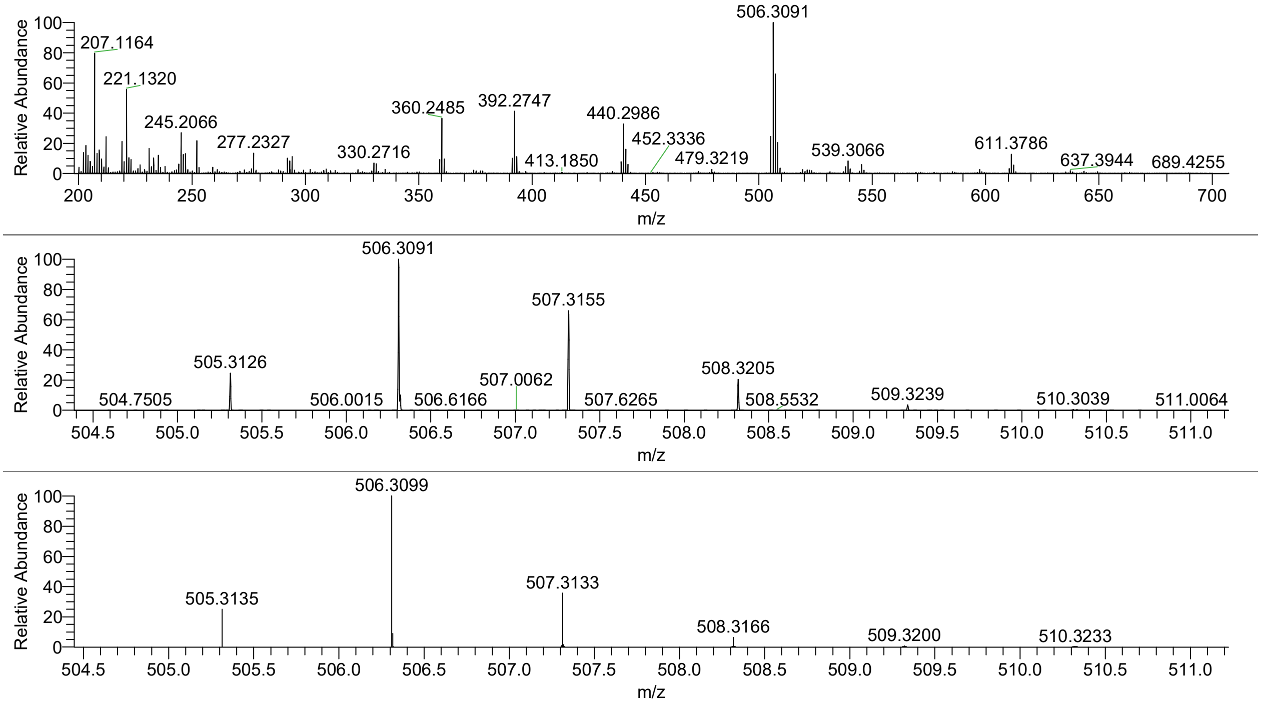

Figure S37. HRMS spectrum (APCI pos) of **7b**.

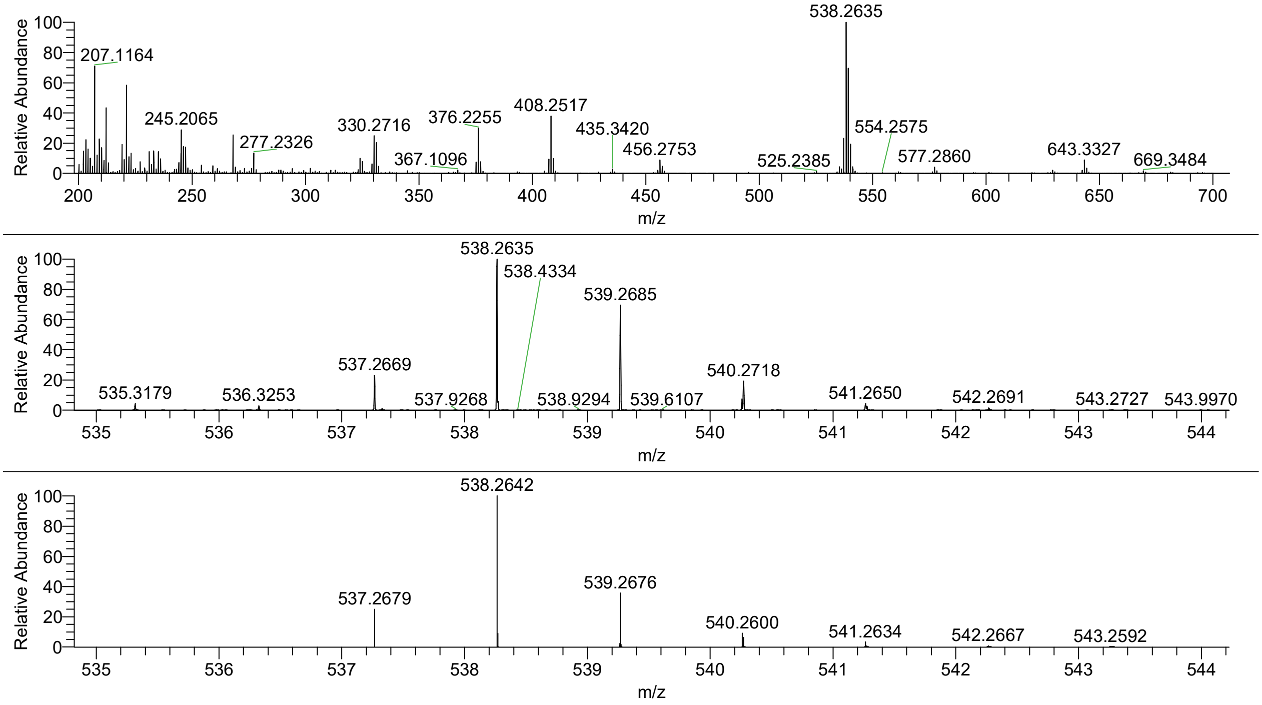

Figure S38. HRMS spectrum (APCI pos) of **7c**.

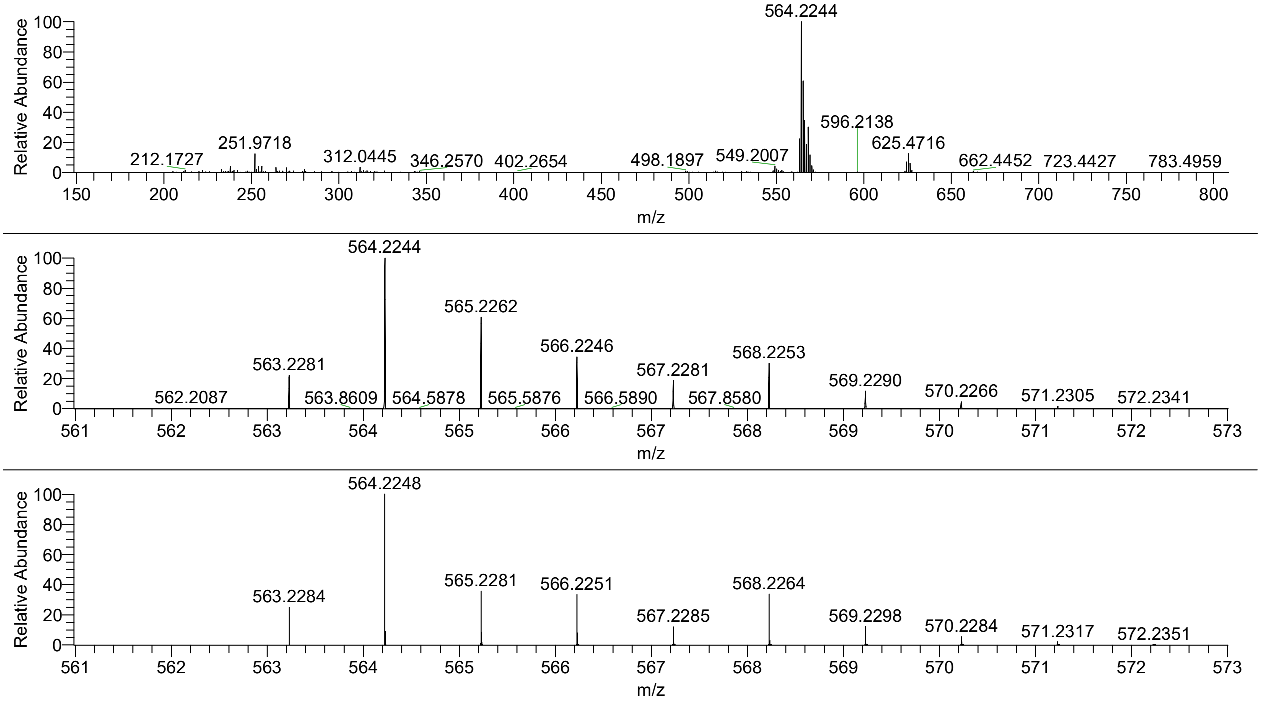

Figure S39: HRMS spectrum (LIFDI) of **9**.

1.6 UV/vis and fluorescence spectra

1.6.1 General data

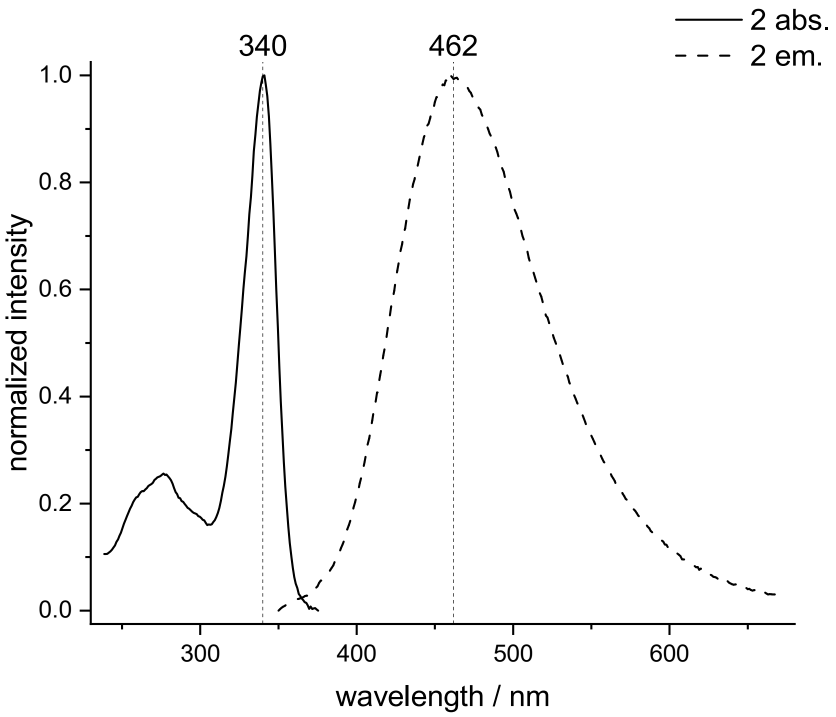

Figure S40. Normalized absorption and emission spectra of **2** in CH_3OH.

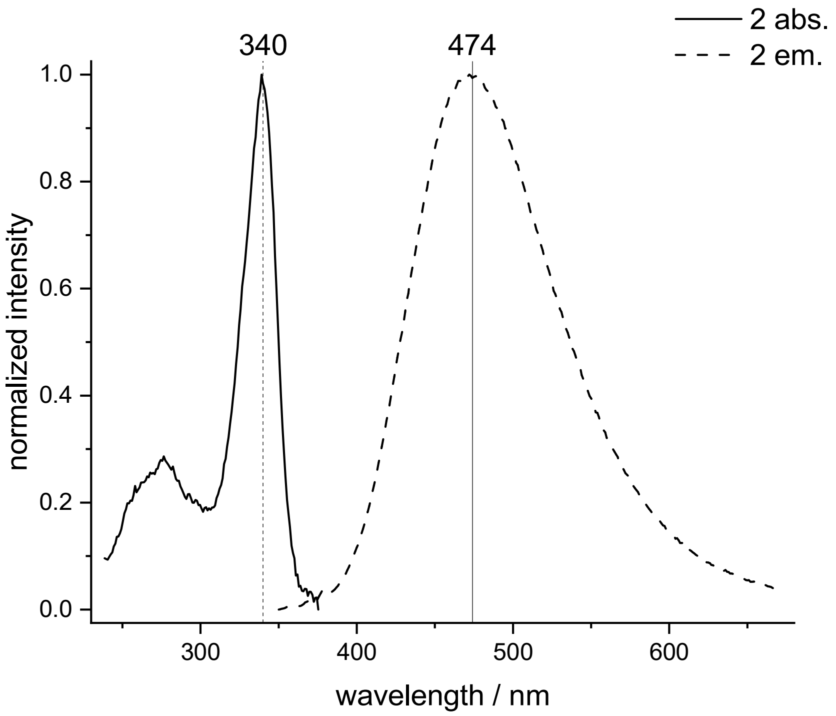

Figure S41. Normalized absorption and emission spectra of **2** in CH_3CN.

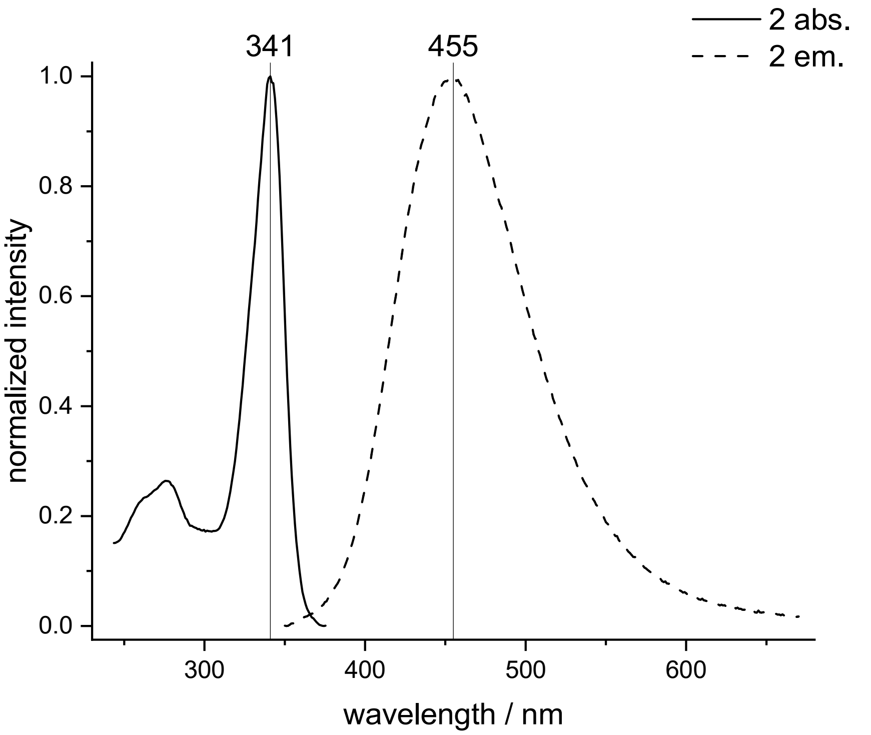

Figure S42. Normalized absorption and emission spectra of **2** in CH_2Cl_2.

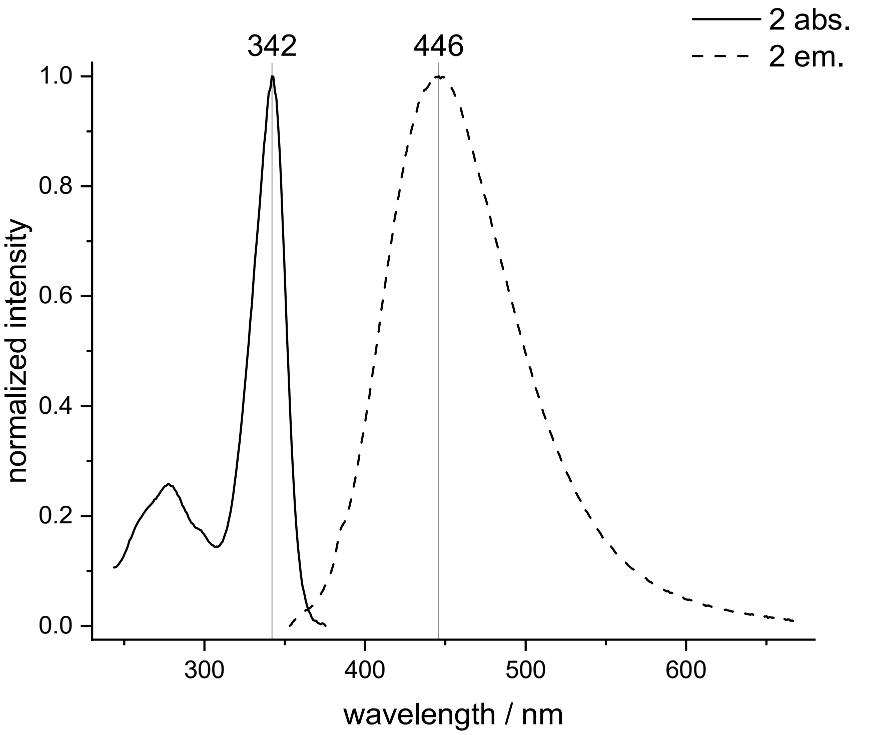

Figure S43. Normalized absorption and emission spectra of **2** in THF.

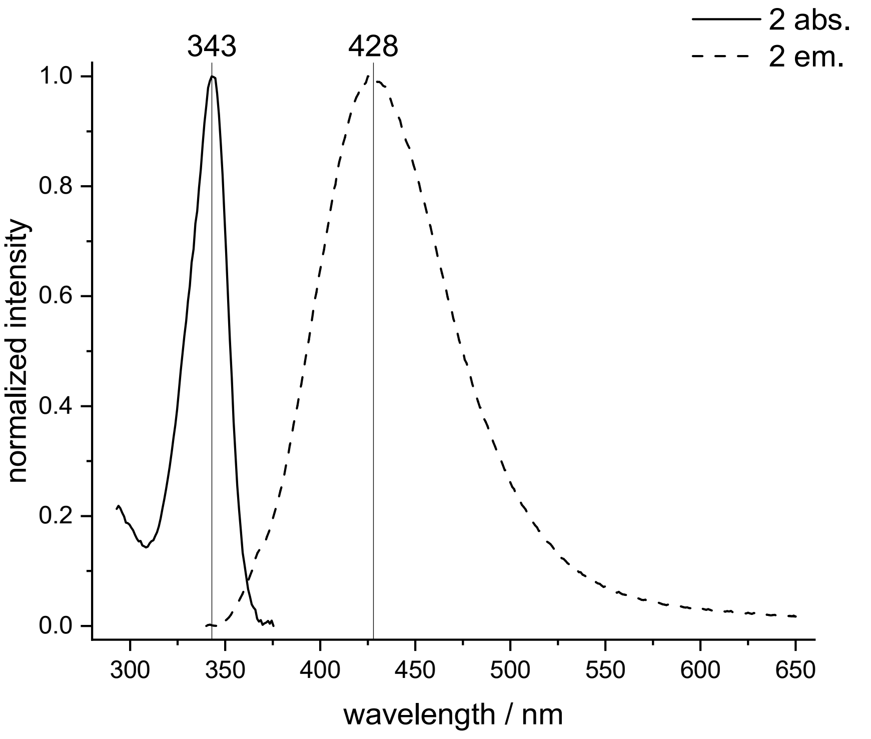

Figure S44. Normalized absorption and emission spectra of **2** in toluene.

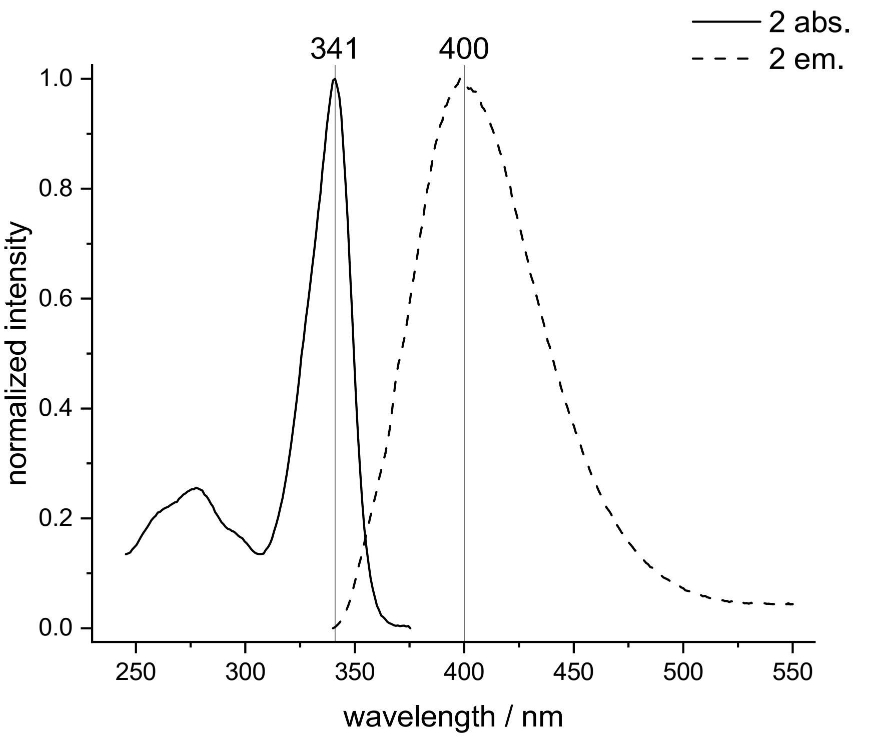

Figure S45. Normalized absorption and emission spectra of **2** in *n*-hexane.

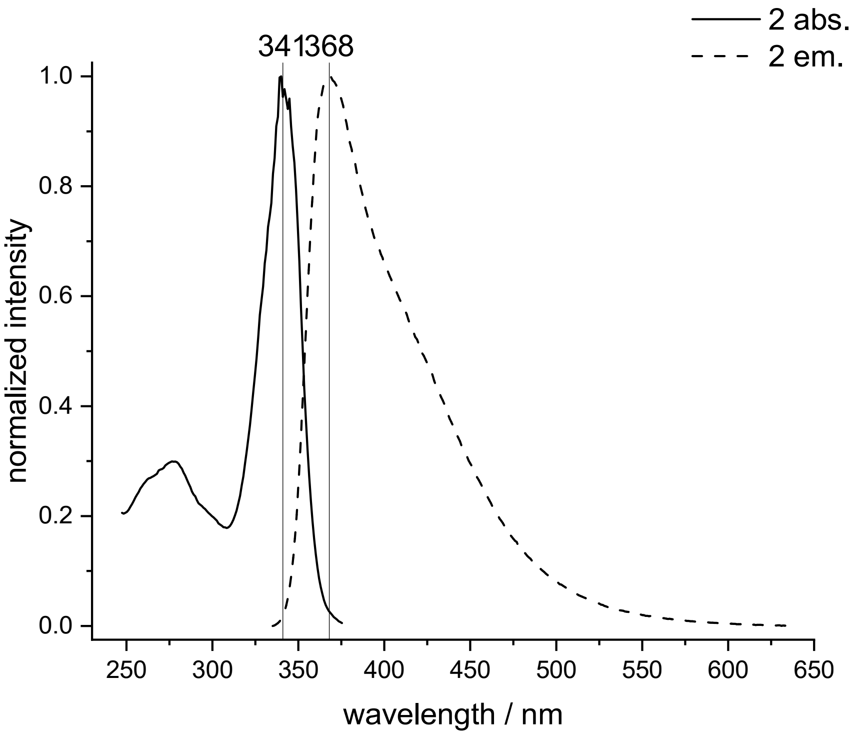

Figure S46. Normalized absorption and emission spectra of **2** in PMMA film.

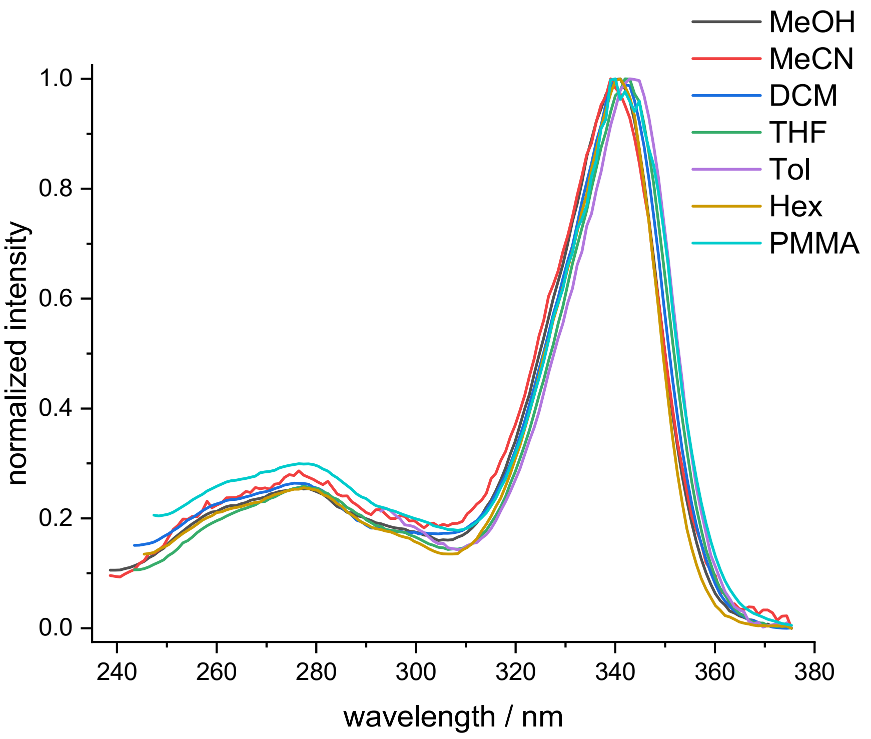

Figure S47. Normalized absorption spectra of **2** in CH_3OH, CH_3CN, CH_2Cl_2, THF, toluene, *n*-hexane and PMMA film.

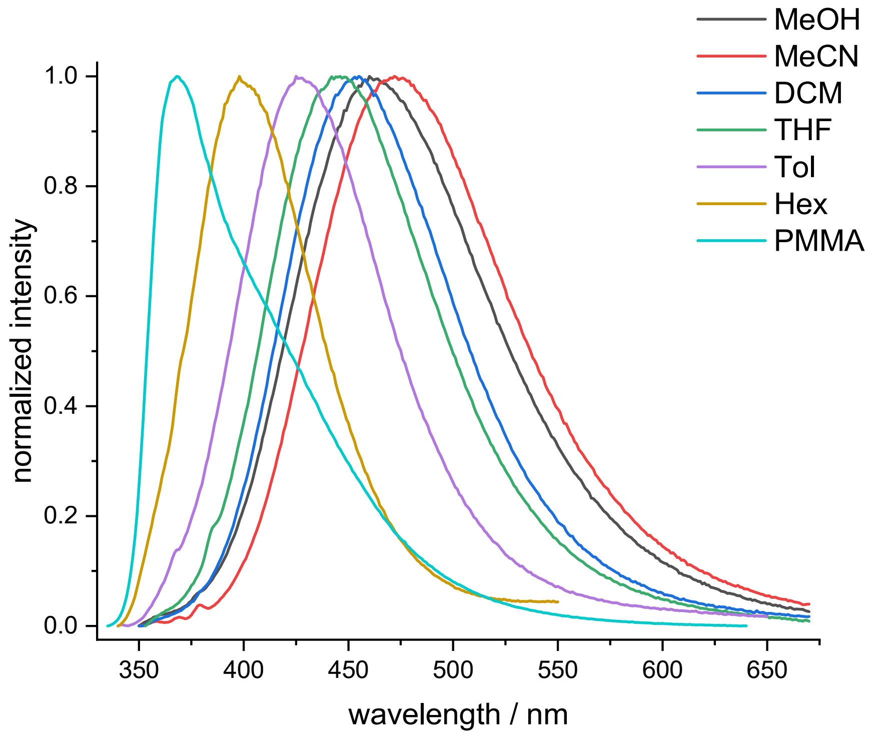

Figure S48. Normalized emission spectra of **2** in CH_3OH, CH_3CN, CH_2Cl_2, THF, toluene, *n*-hexane and PMMA film.

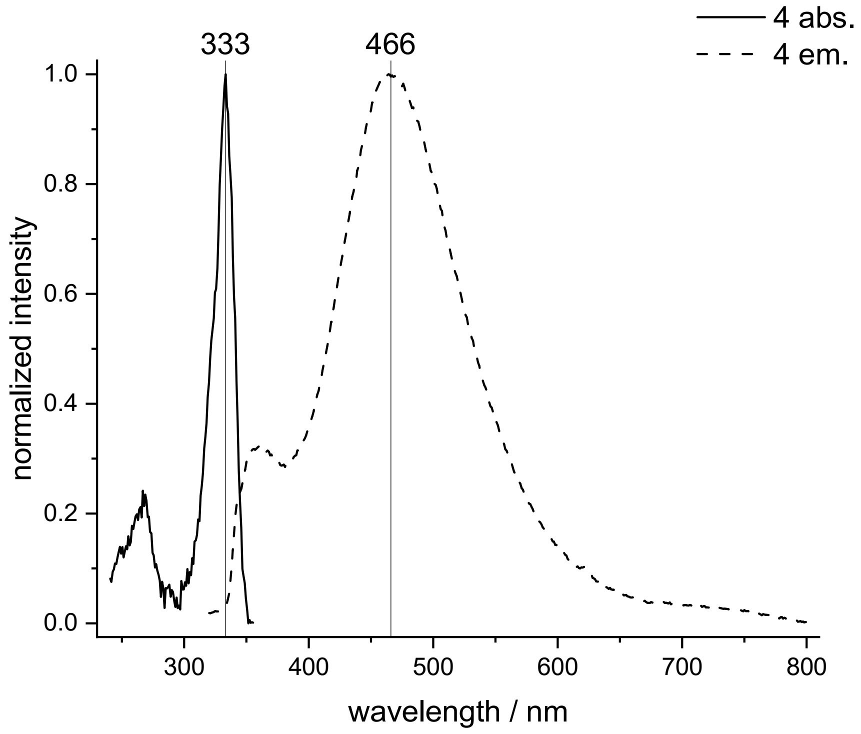

Figure S49. Normalized absorption and emission spectra of **4** in CH_3OH.

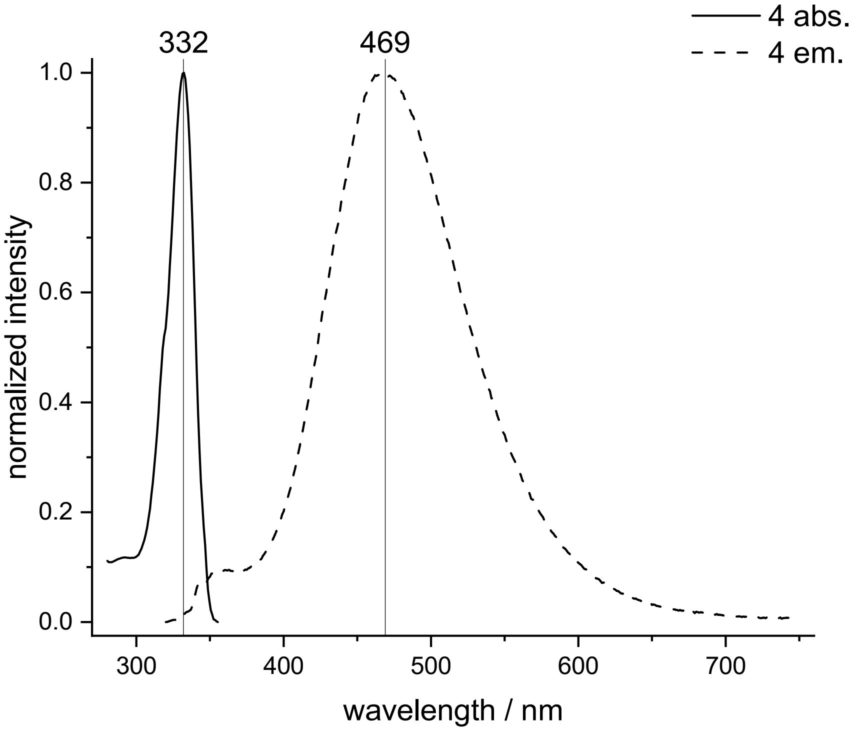

Figure S50. Normalized absorption and emission spectra of **4** in CH_3CN.

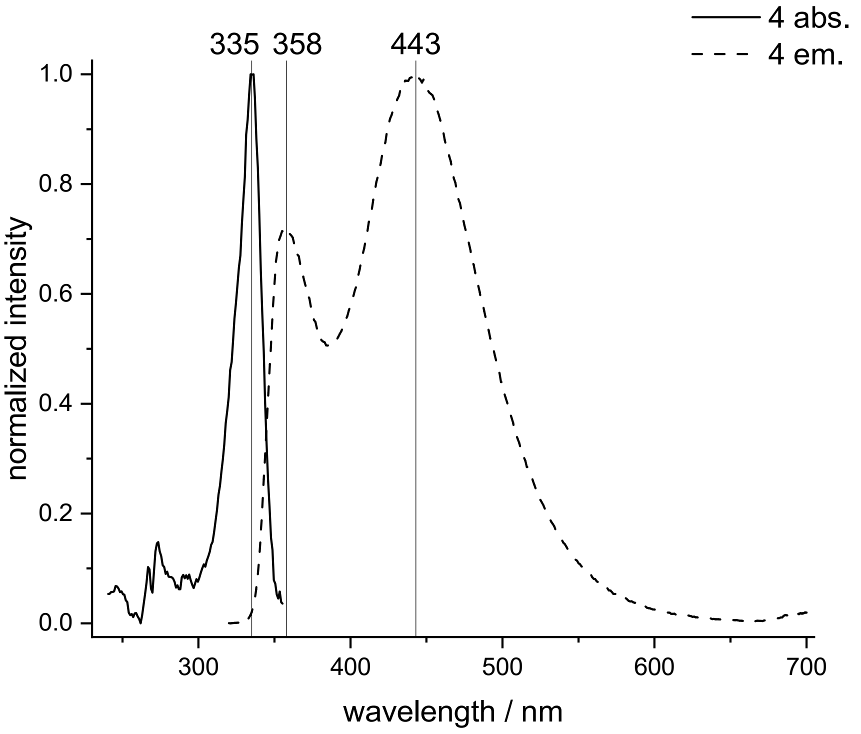

Figure S51. Normalized absorption and emission spectra of **4** in CH_2Cl_2.

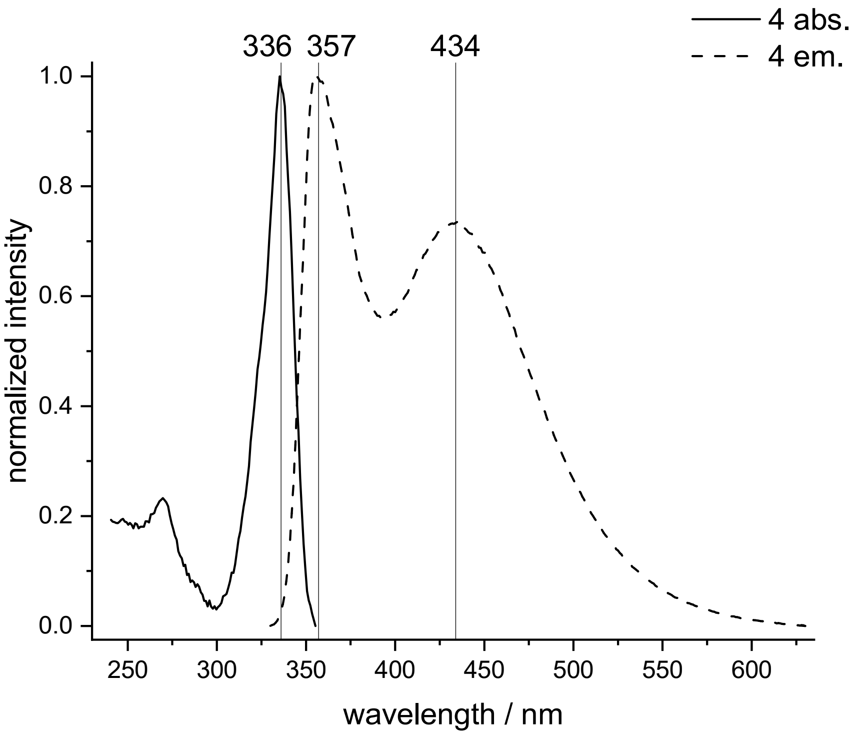

Figure S52. Normalized absorption and emission spectra of **4** in THF.

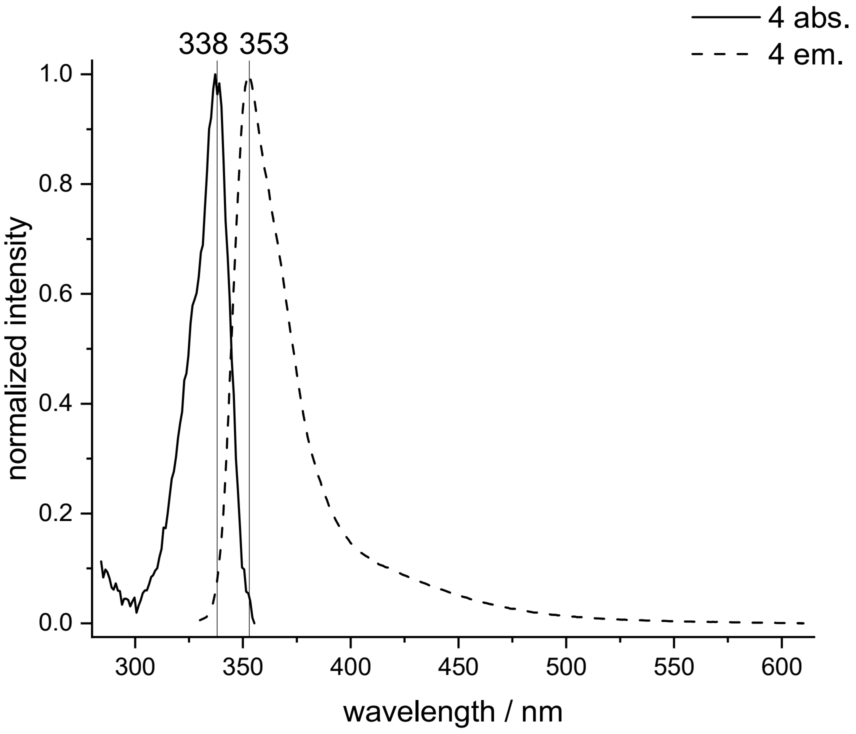

Figure S53. Normalized absorption and emission spectra of **4** in toluene.

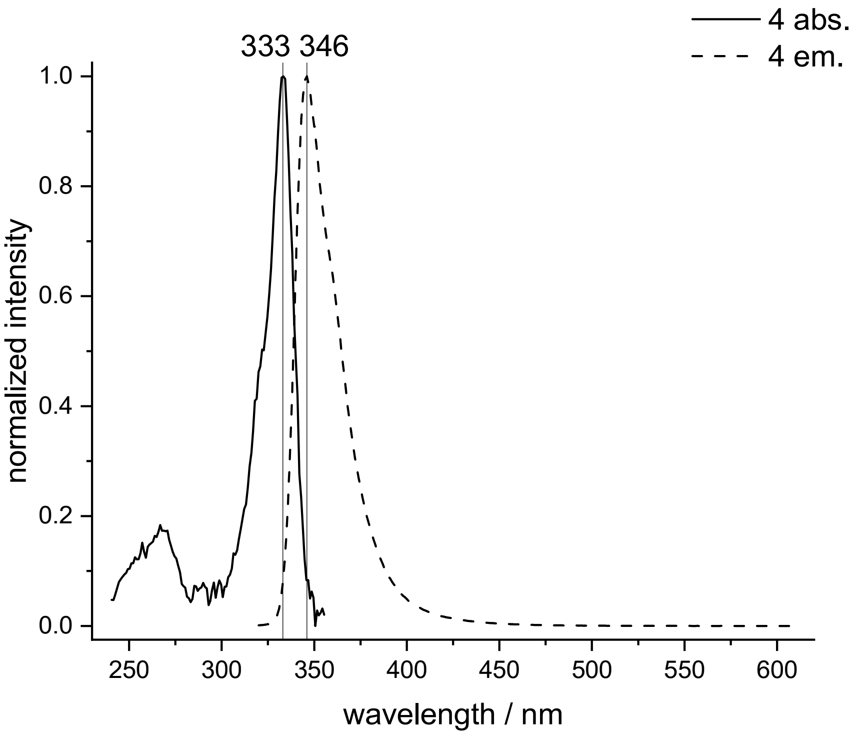

Figure S54. Normalized absorption and emission spectra of **4** in *n*-hexane.

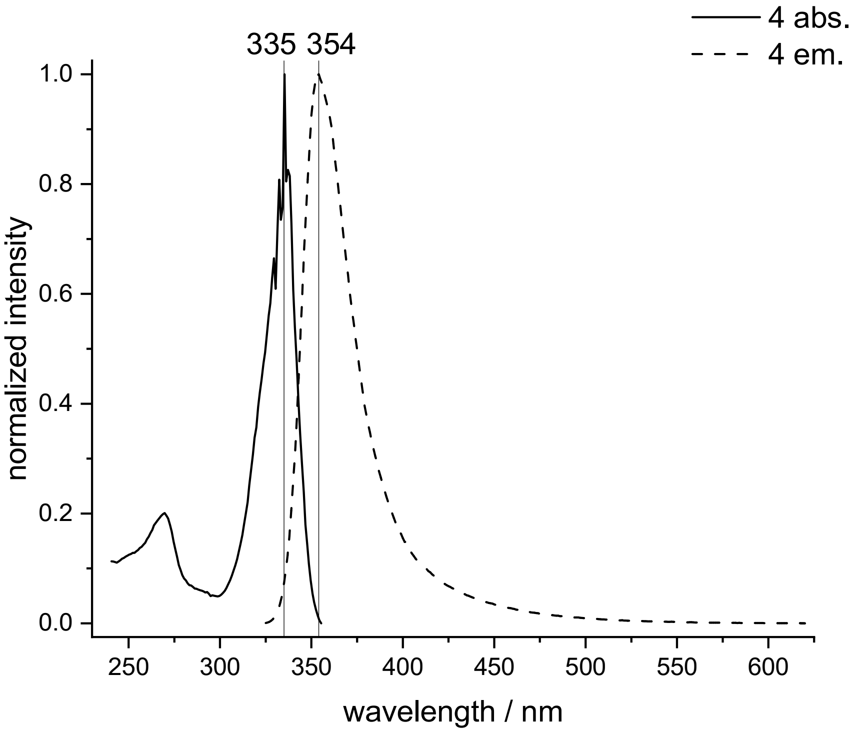

Figure S55. Normalized absorption and emission spectra of **4** in PMMA film.

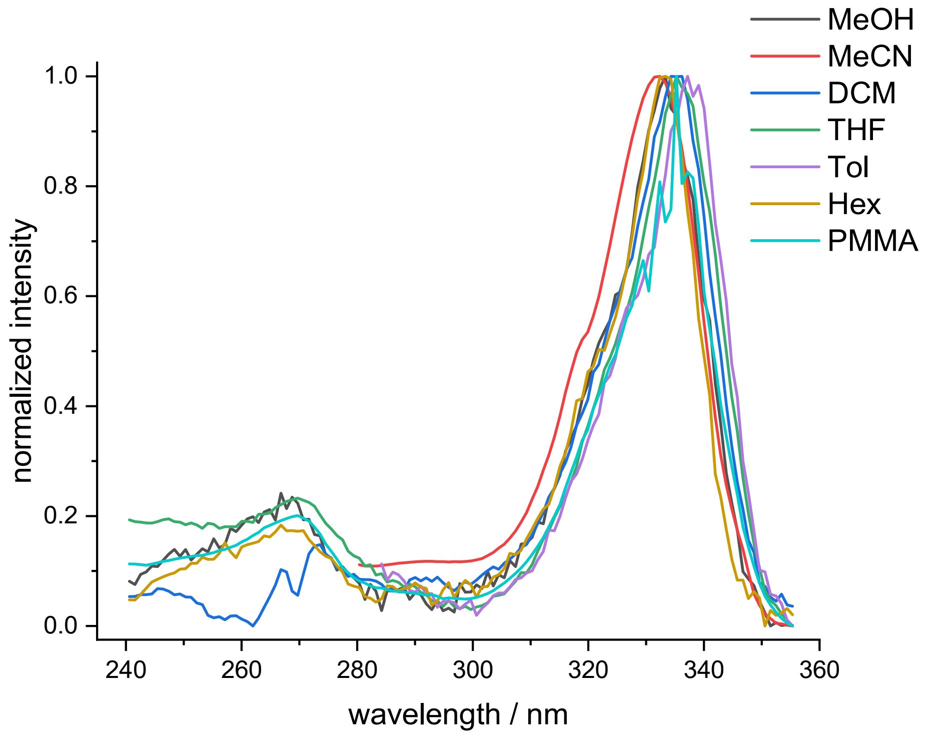

Figure S56. Normalized absorption spectra of **4** in CH_3OH, CH_3CN, CH_2Cl_2, THF, toluene, *n*-hexane and PMMA film.

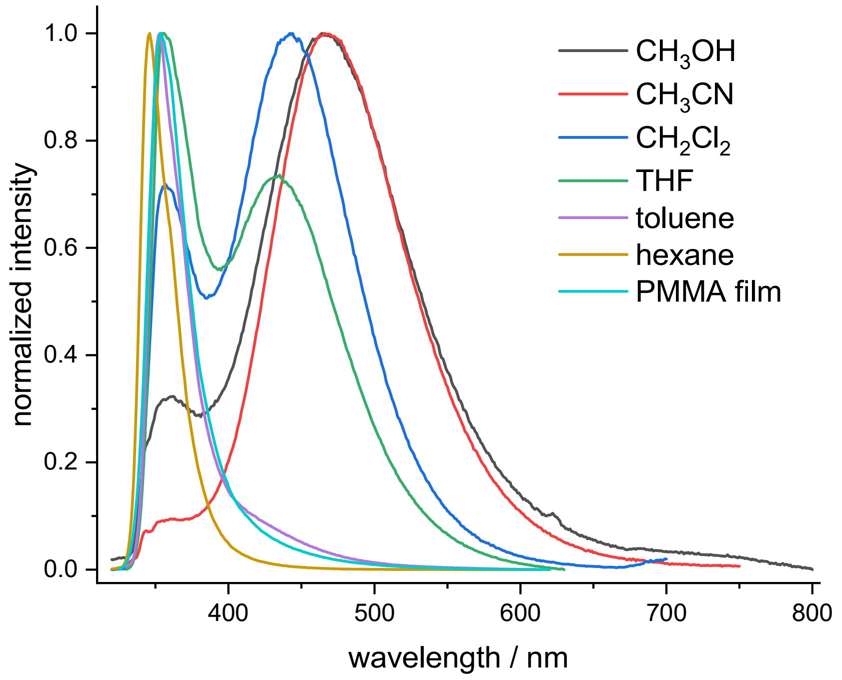

Figure S57. Normalized emission spectra of **4** in CH_3OH, CH_3CN, CH_2Cl_2, THF, toluene, *n*-hexane and PMMA film.

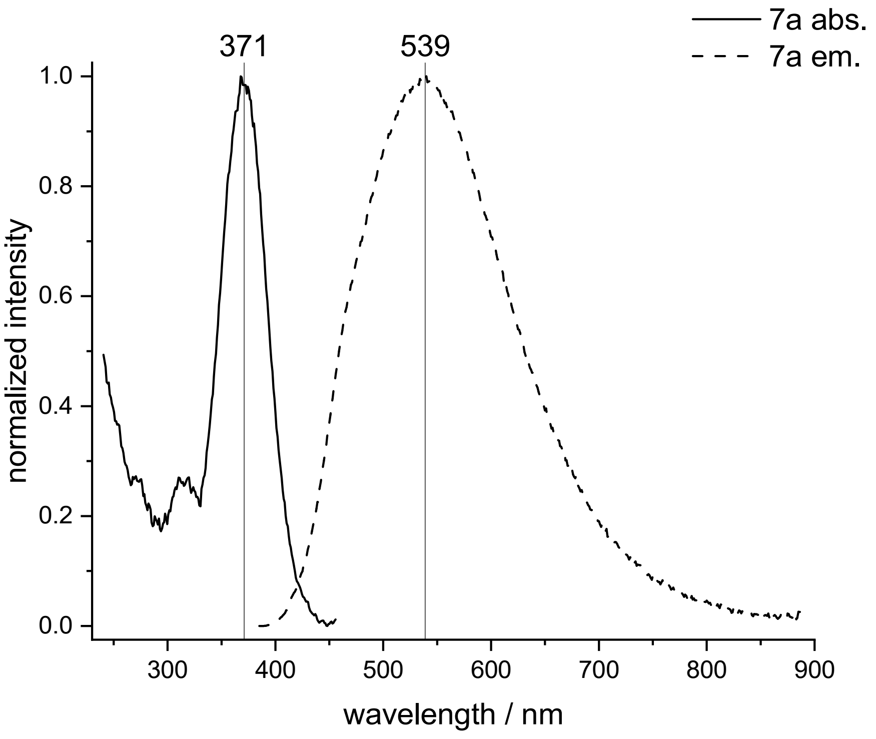

Figure S58. Normalized absorption and emission spectra of **7a** in CH_3OH.

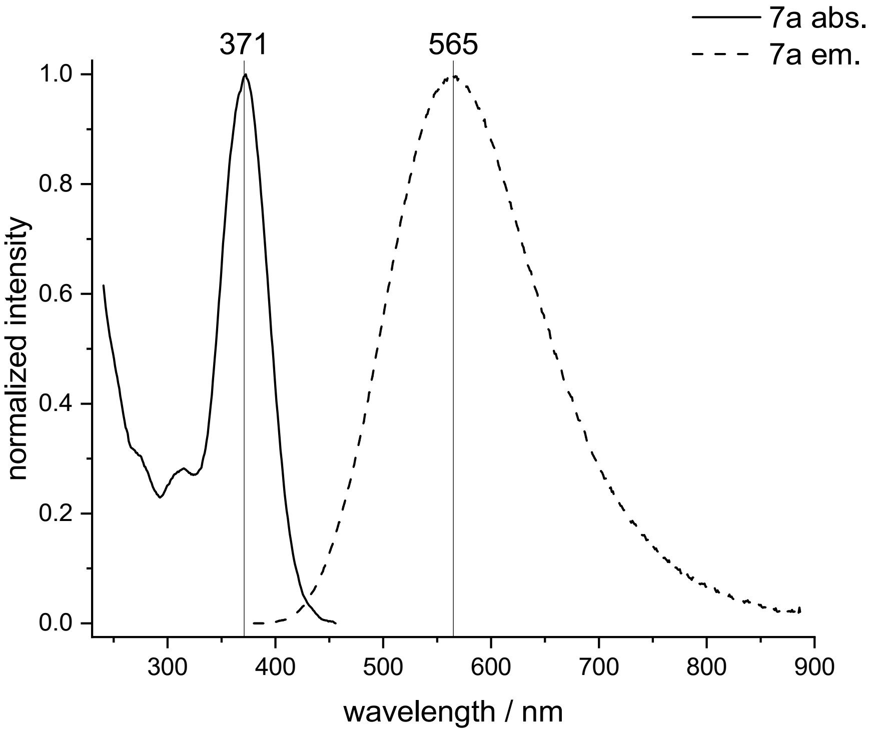

Figure S59. Normalized absorption and emission spectra of **7a** in CH_3CN.

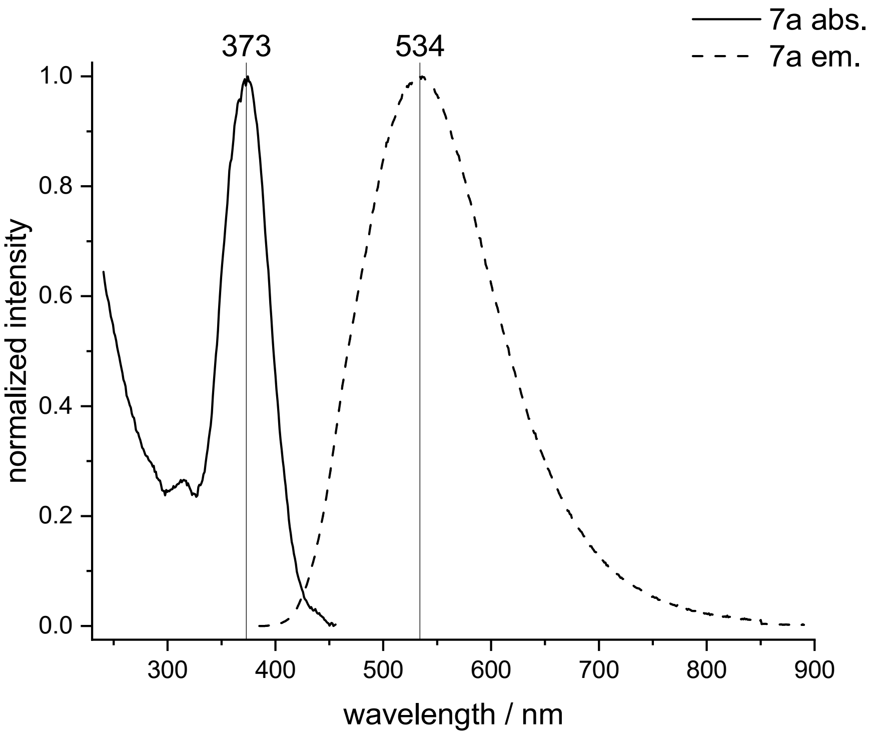

Figure S60. Normalized absorption and emission spectra of **7a** in CH_2Cl_2.

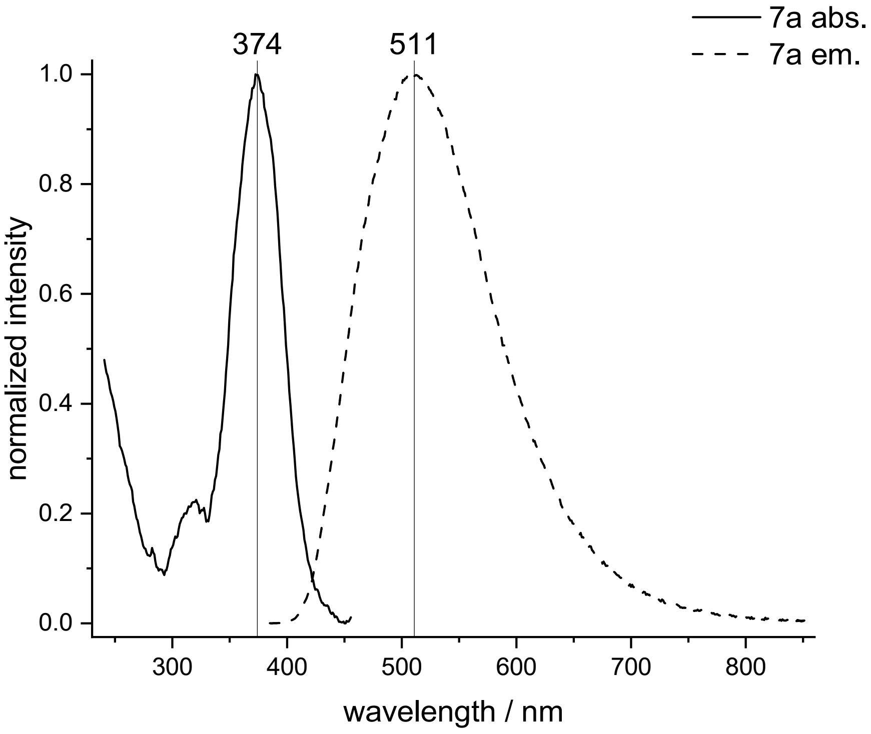

Figure S61. Normalized absorption and emission spectra of **7a** in THF.

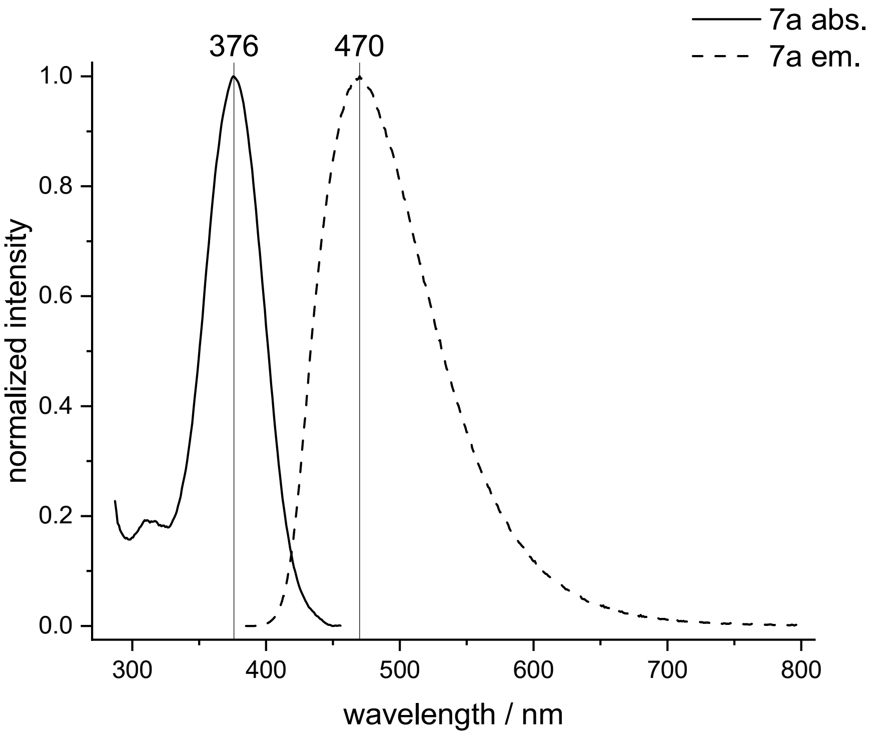

Figure S62. Normalized absorption and emission spectra of **7a** in toluene.

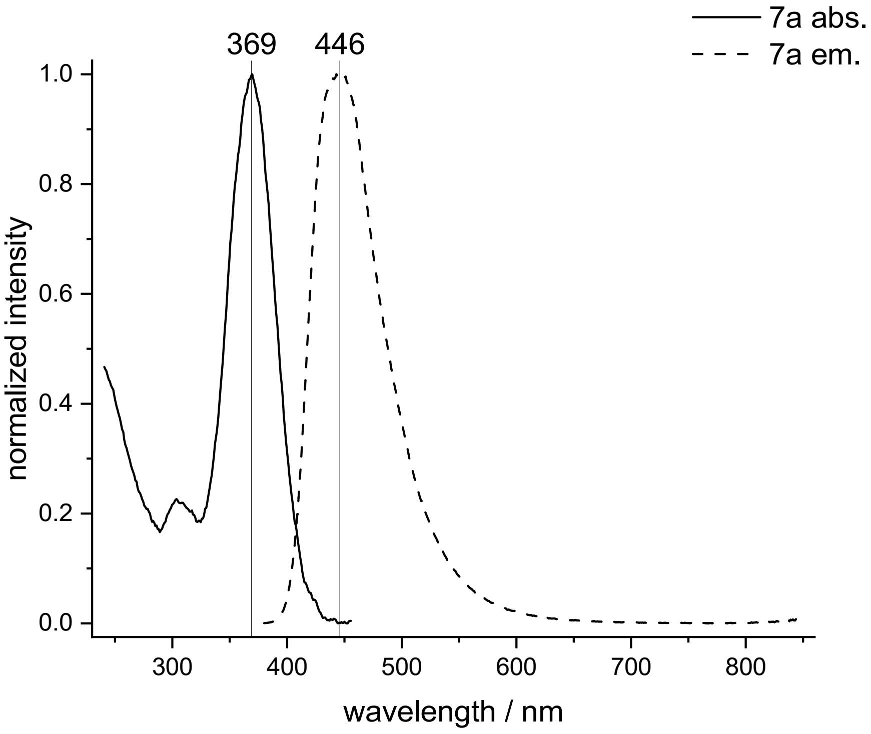

Figure S63. Normalized absorption and emission spectra of **7a** in *n*-hexane.

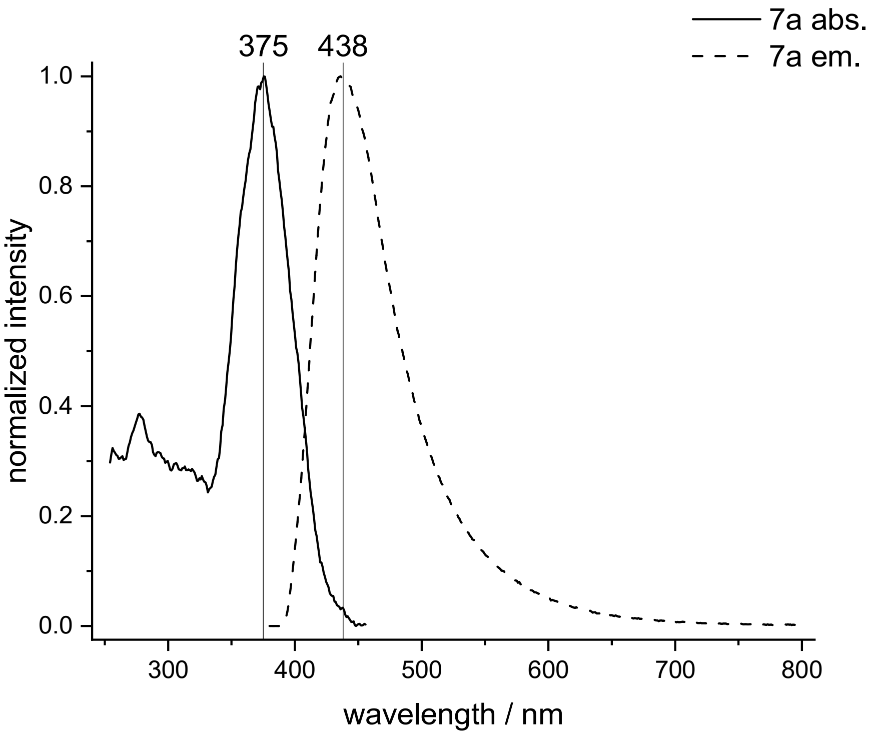

Figure S64. Normalized absorption and emission spectra of **7a** in PMMA film.

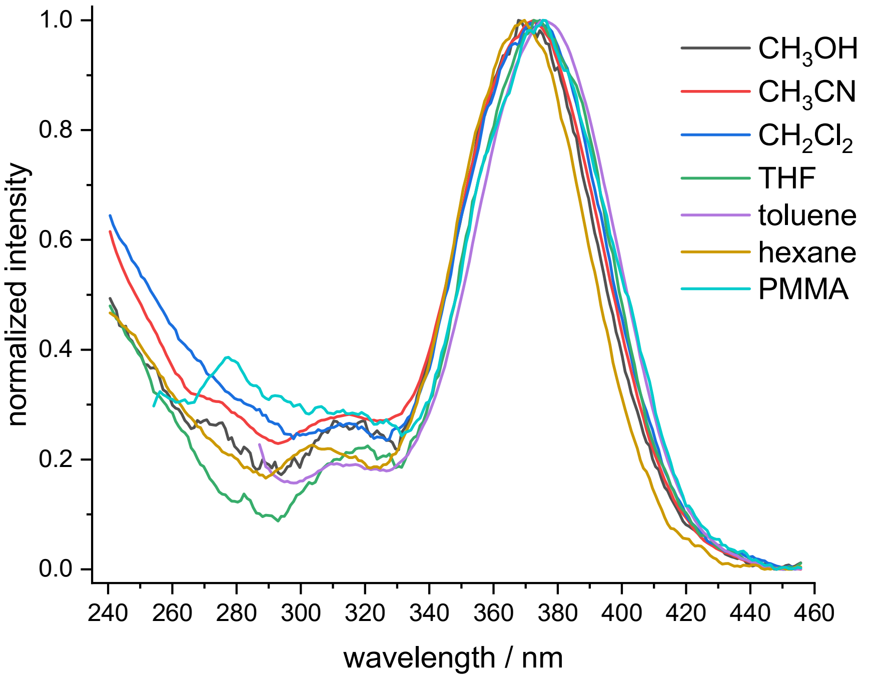

Figure S65. Normalized absorption spectra of **7a** in CH_3OH, CH_3CN, CH_2Cl_2, THF, toluene, *n*-hexane and PMMA film.

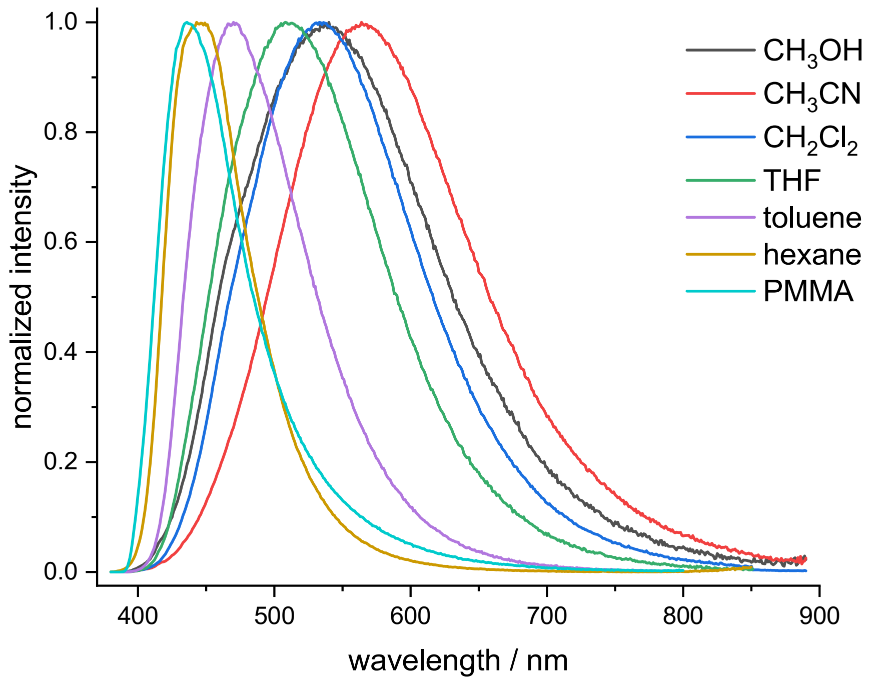

Figure S66. Normalized emission spectra of **7a** in CH_3OH, CH_3CN, CH_2Cl_2, THF, toluene, *n*-hexane and PMMA film.

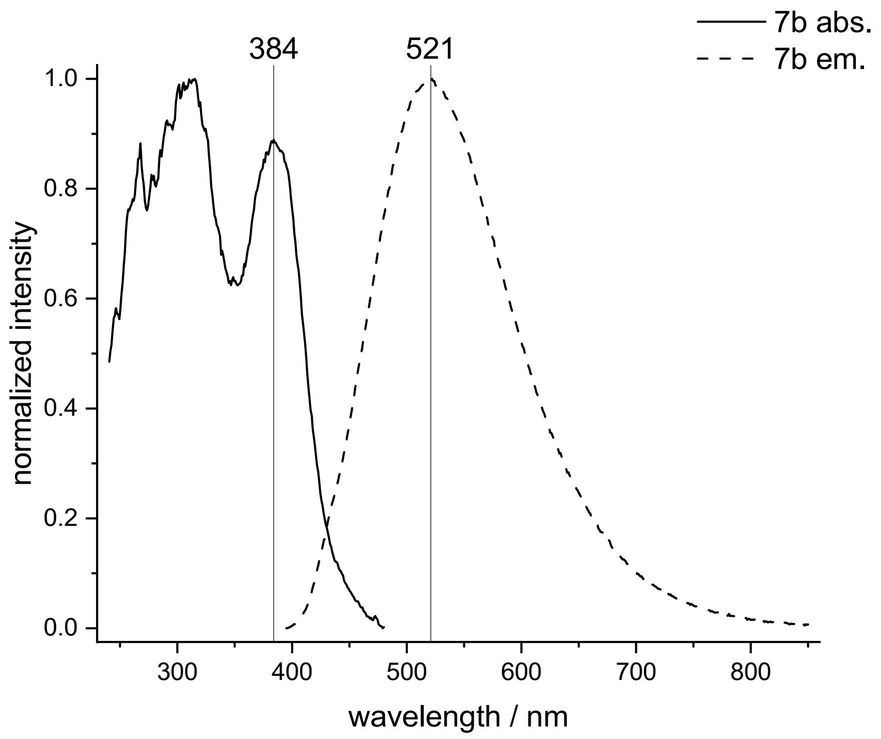

Figure S67. Normalized absorption and emission spectra of **7b** in CH_3OH.

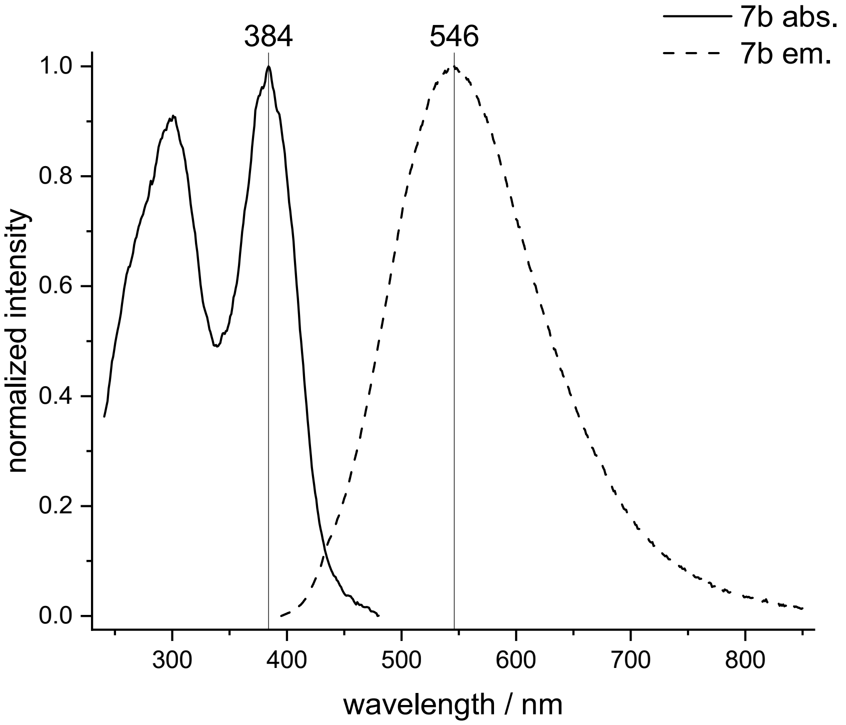

Figure S68. Normalized absorption and emission spectra of **7b** in CH_3CN.

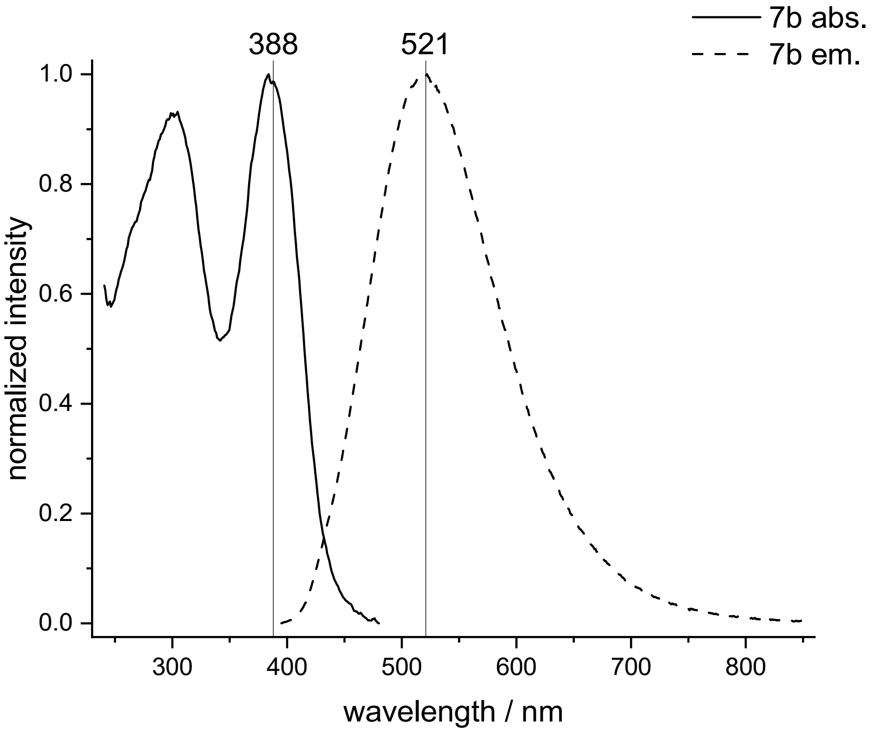

Figure S69. Normalized absorption and emission spectra of **7b** in CH_2Cl_2.

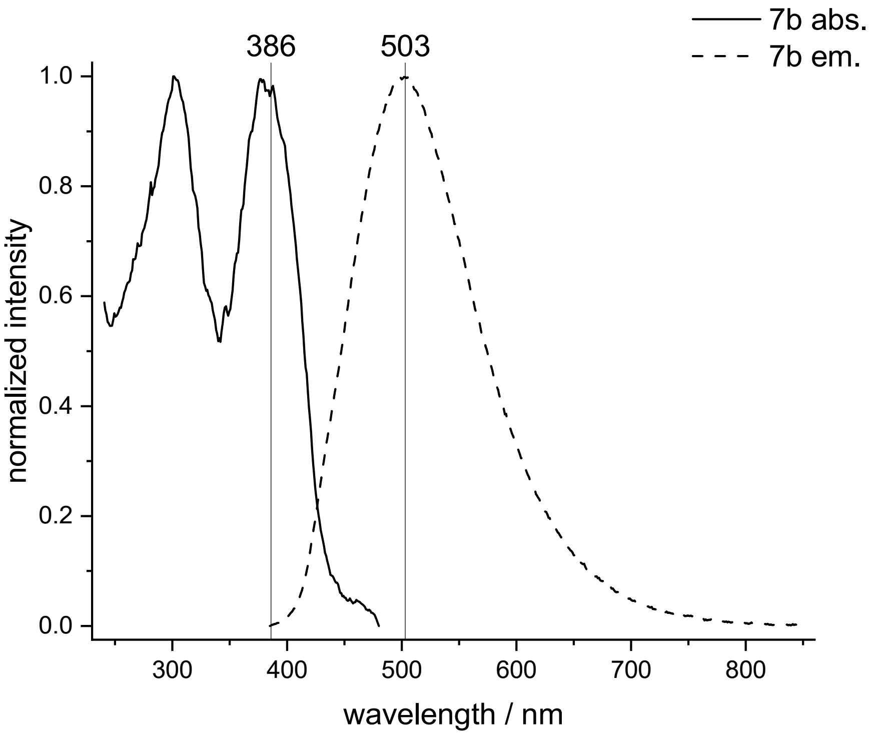

Figure S70. Normalized absorption and emission spectra of **7b** in THF.

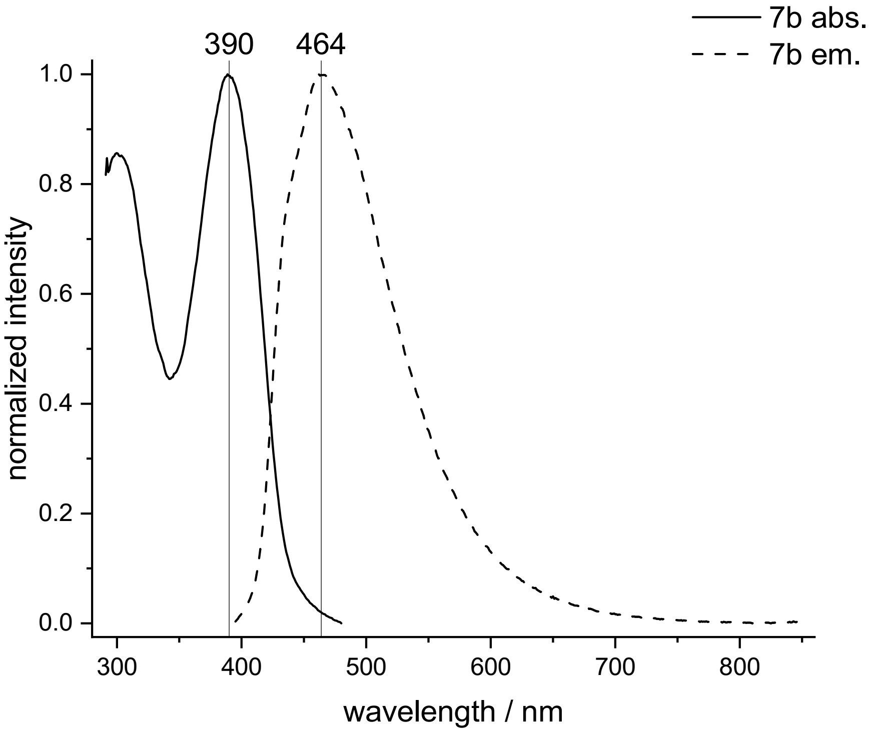

Figure S71. Normalized absorption and emission spectra of **7b** in toluene.

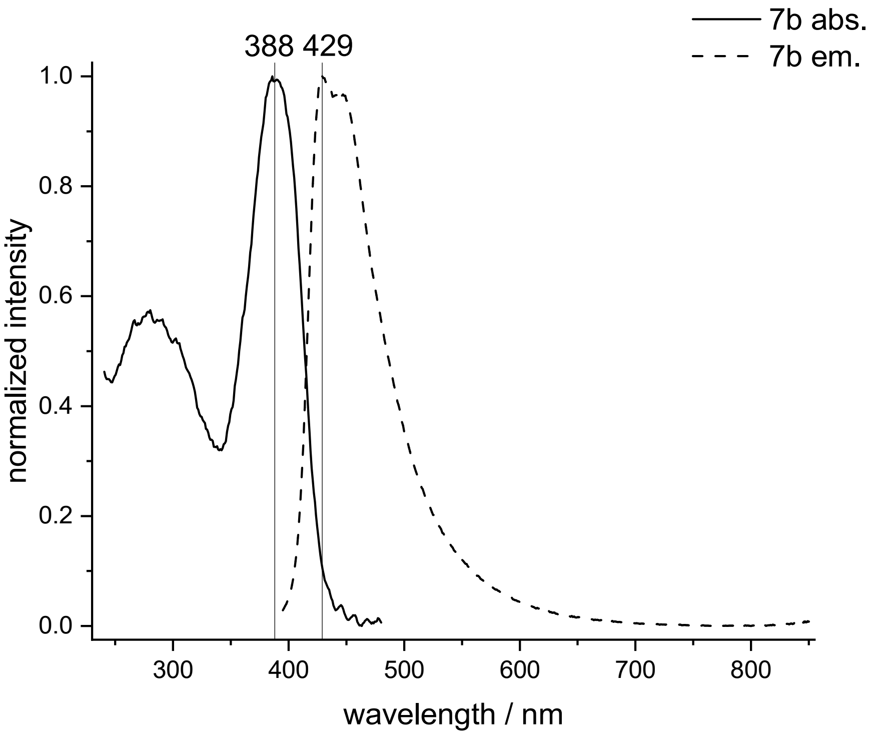

Figure S72. Normalized absorption and emission spectra of **7b** in *n*-hexane.

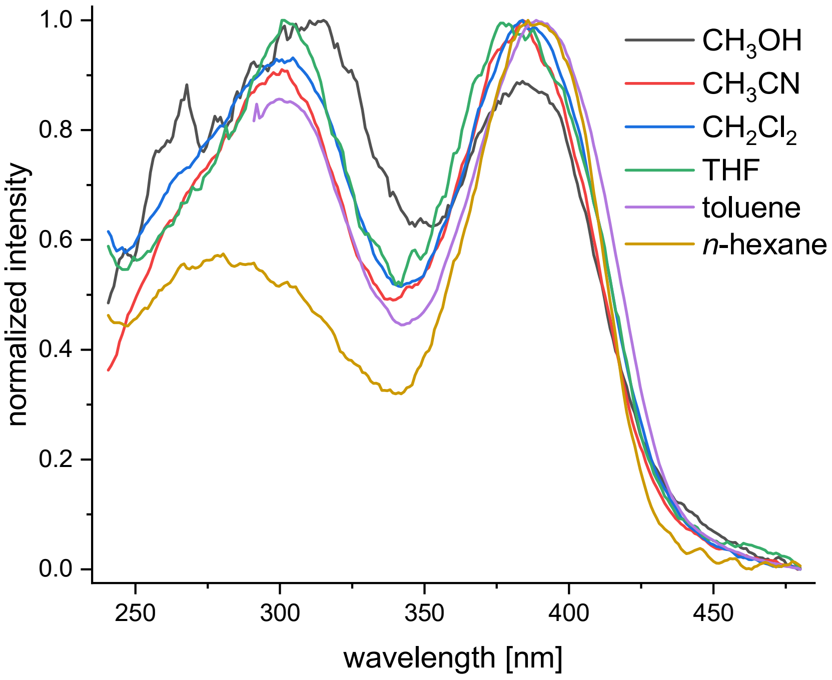

Figure S73. Normalized absorption spectra of **7b** in CH_3OH, CH_3CN, CH_2Cl_2, THF, toluene and *n*-hexane.

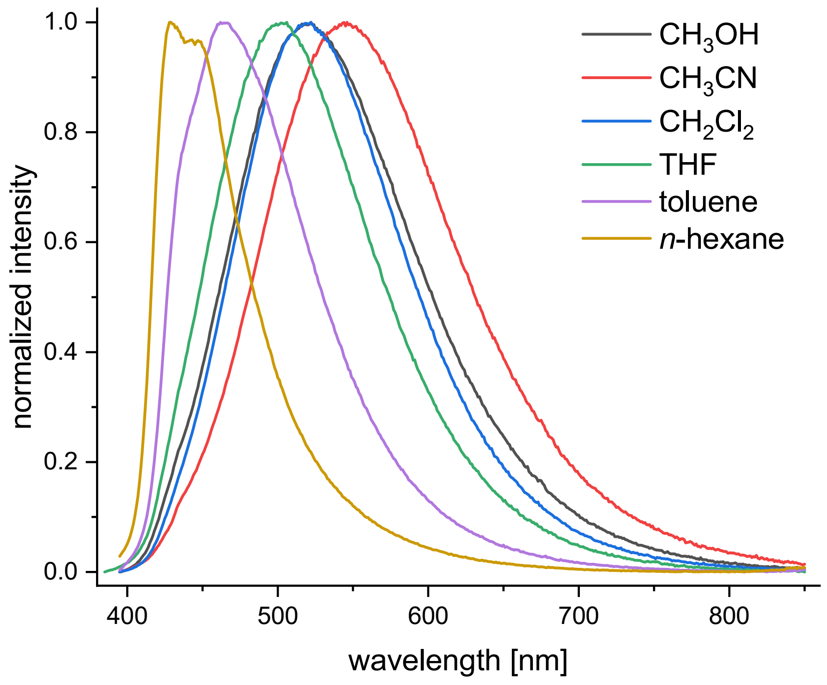

Figure S74. Normalized emission spectra of **7b** in CH_3OH, CH_3CN, CH_2Cl_2, THF, toluene and *n*-hexane.

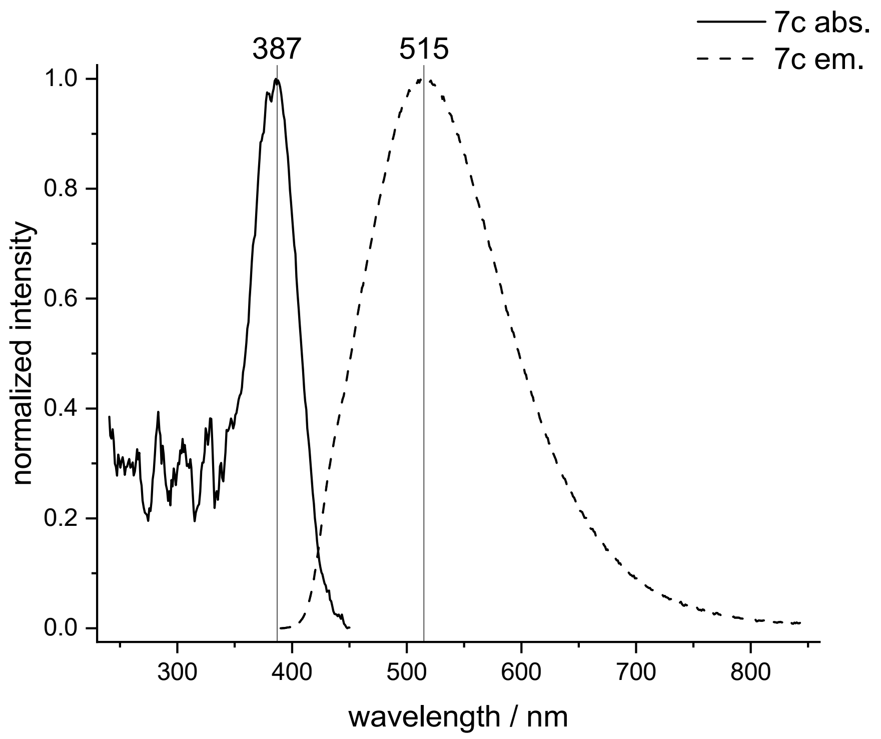

Figure S75. Normalized absorption and emission spectra of **7c** in CH_3OH.

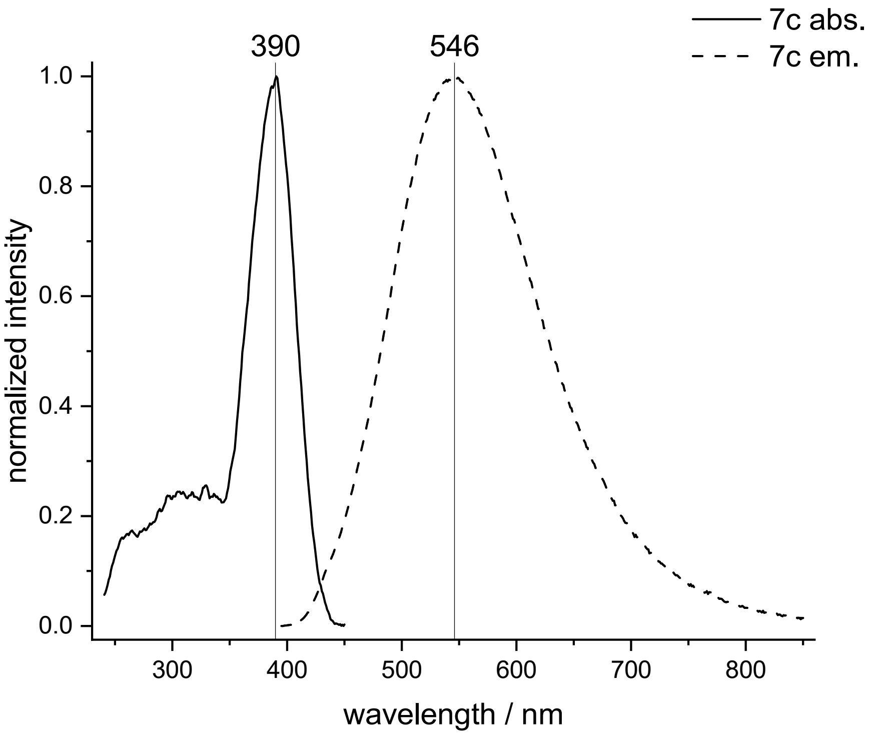

Figure S76. Normalized absorption and emission spectra of **7c** in CH_3CN.

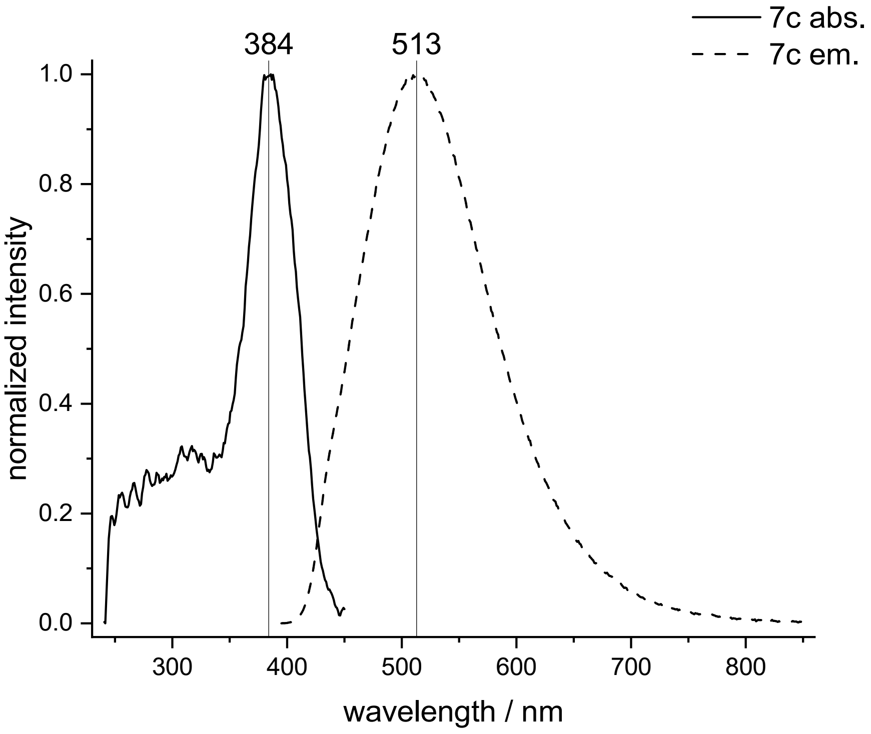

Figure S77. Normalized absorption and emission spectra of **7c** in CH_2Cl_2.

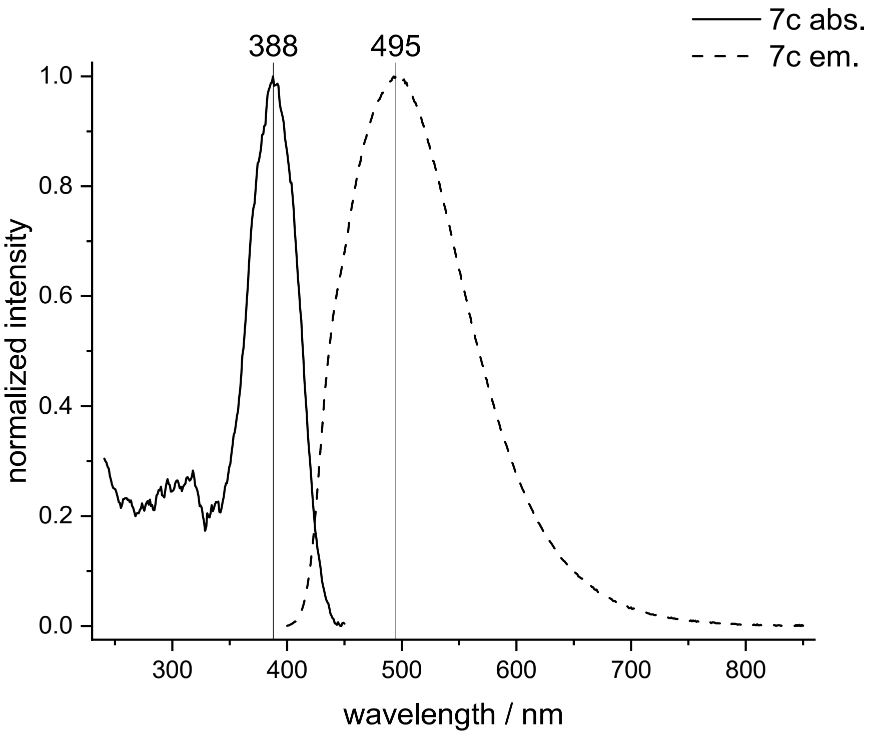

Figure S78. Normalized absorption and emission spectra of **7c** in THF.

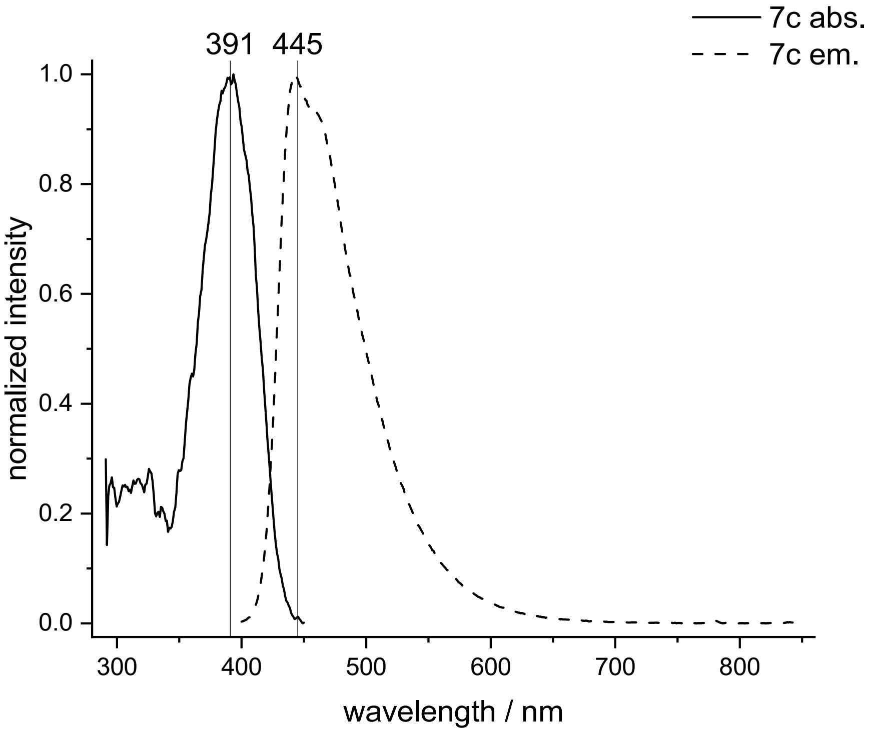

Figure S79. Normalized absorption and emission spectra of **7c** in toluene.

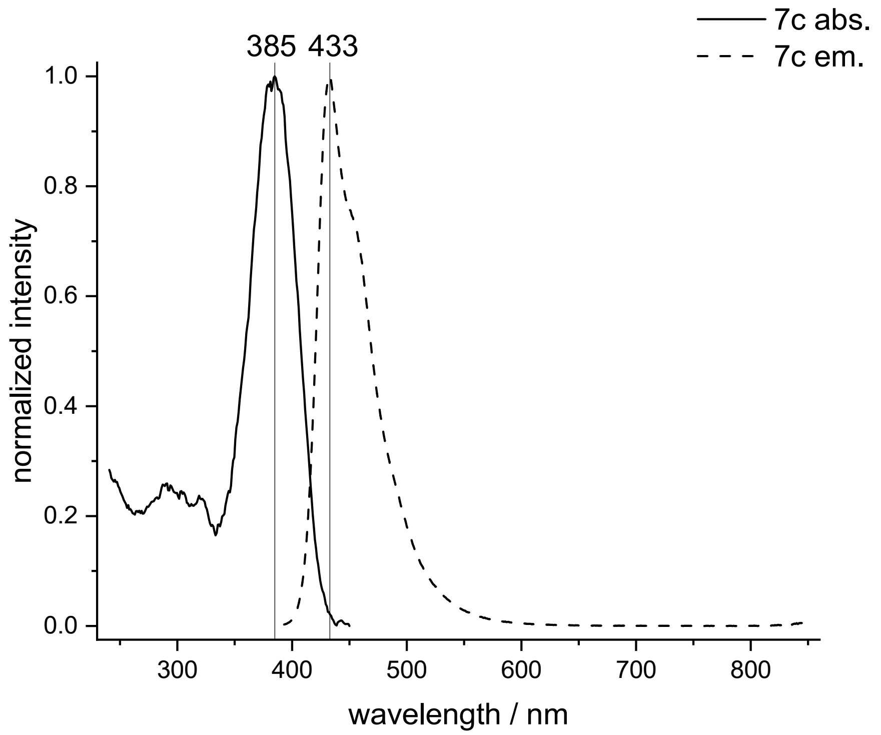

Figure S80. Normalized absorption and emission spectra of **7c** in *n*-hexane.

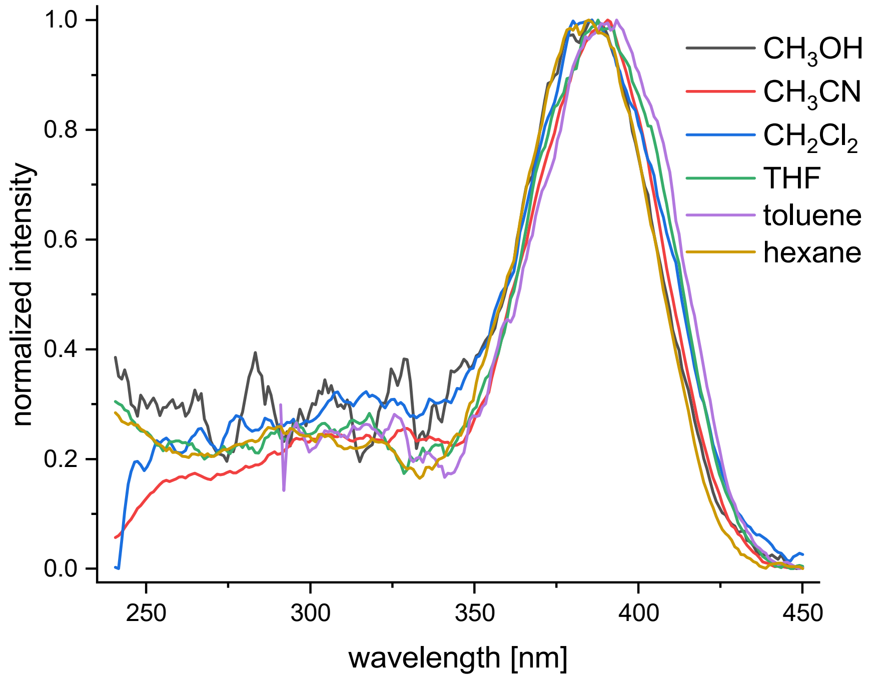

Figure S81. Normalized absorption spectra of **7c** in CH_3OH, CH_3CN, CH_2Cl_2, THF, toluene and *n*-hexane.

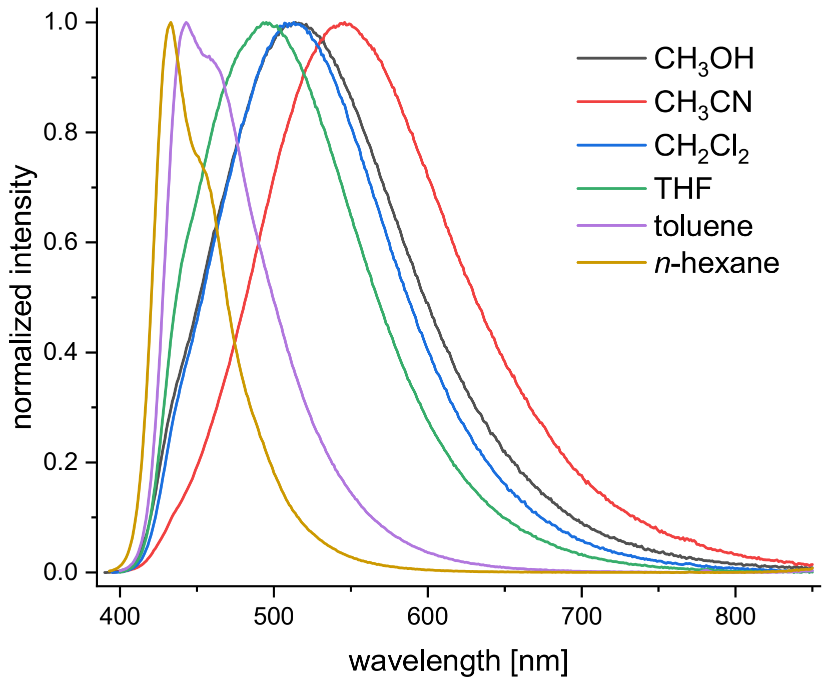

Figure S82. Normalized emission spectra of **7c** in CH_3OH, CH_3CN, CH_2Cl_2, THF, toluene and *n*-hexane.

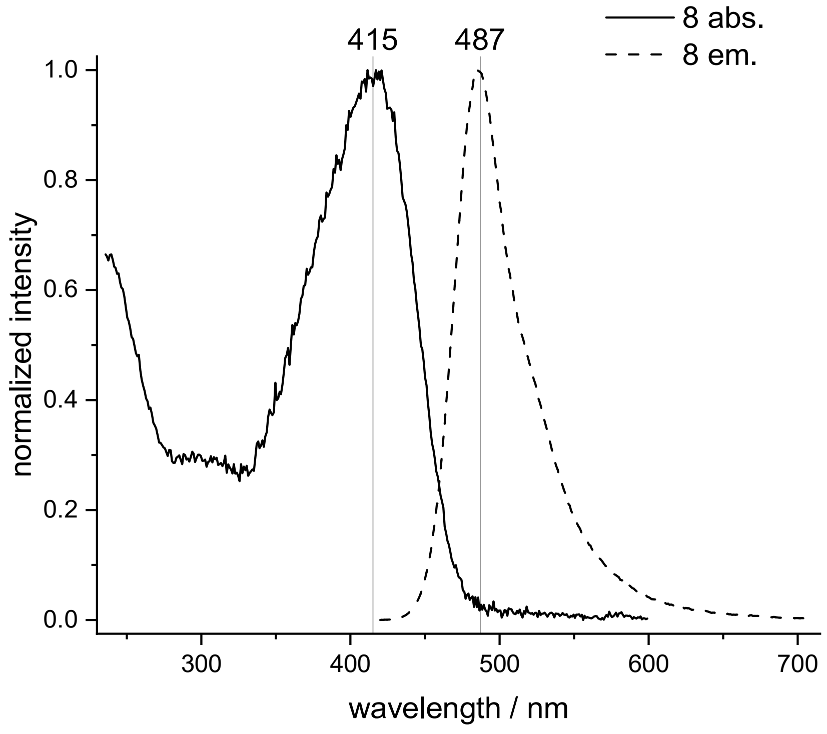

Figure S83. Normalized absorption and emission spectra of the polymer **8** in THF.

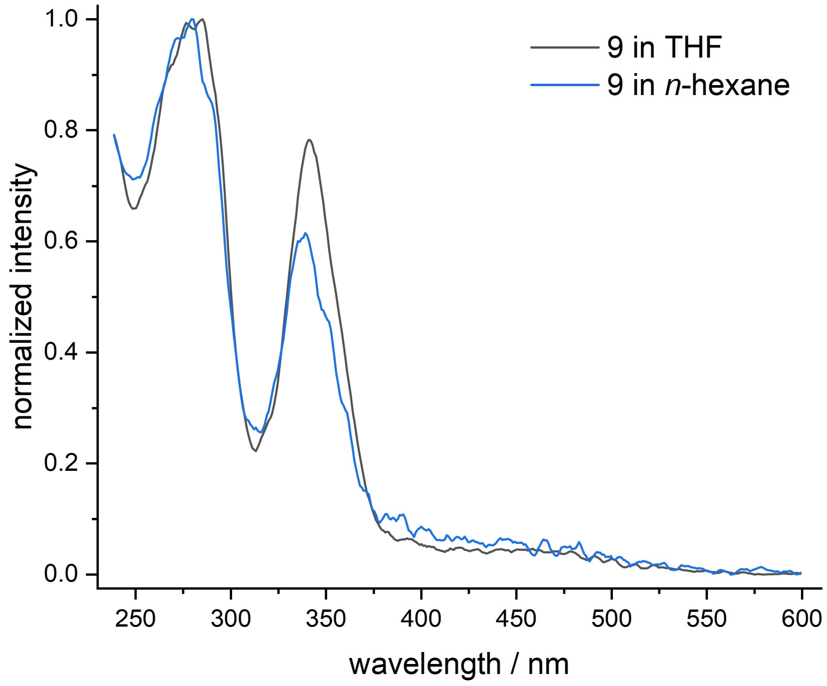

Figure S84. Normalized absorption spectra of **9** in THF and *n*-hexane.

Table S3. Experimentally obtained quantum yields Φ_F [%] and lifetimes τ [ns] for **2**, **4** and **7a-c** in different solvents and PMMA film.

**Compound**	**Φ_F [MeOH]**	**τ [MeOH]**	**Φ_F [THF]**	**τ [THF]**	**Φ_F [*n*-hexane]**	**τ [*n*-hexane]**	**Φ_F [PMMA]**
**2**	0.02	2.06	0.14	4.06	0.21	3.28	0.19
**4**	0.05	5.52	0.26	0.50, 7.47	0.38	1.77	0.70
**7a**	0.04	1.59, 3.89	0.46	4.12, 9.01	0.60	0.83, 3.22	0.70
**7b**	0.05	3.25, 6.06	0.34	3.82, 9.18	0.41	1.06, 2.98	
**7c**	0.07		0.42	1.67, 4.03	0.84	0.52, 1.84	

1.6.2 Lifetime measurements

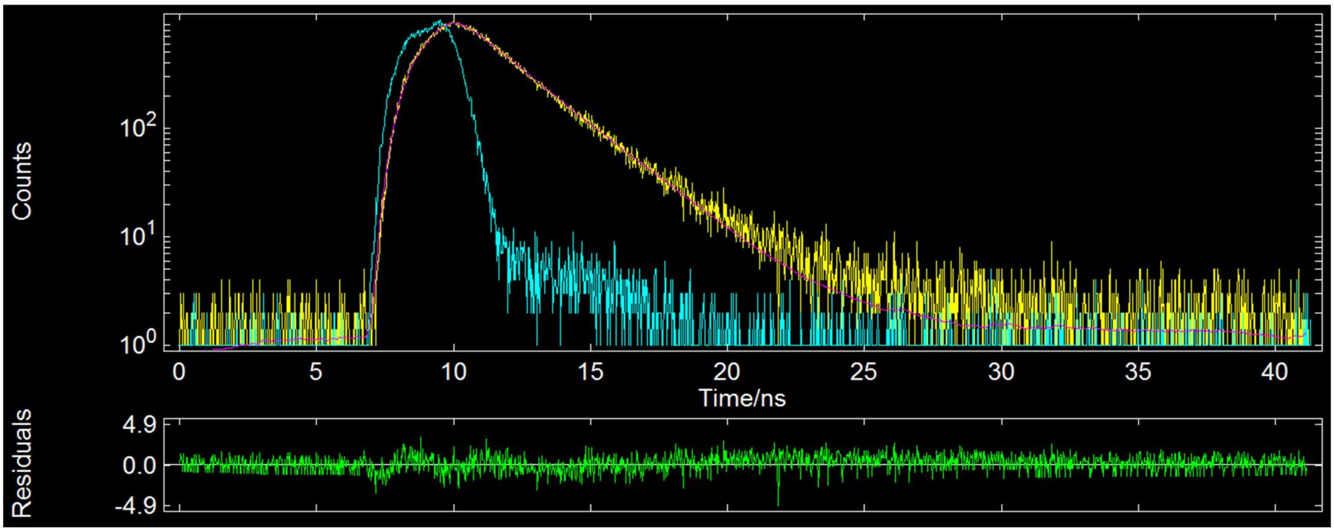

EDINBURGH INSTRUMENTS
F900 23/10/2025

Fit Results

Fit : A+B1exp(-t/τ_1)

Instrument Response	: **IRF_316_MeOH_stop1kcounts**
Range (ch)	: 0 to 4095
Peak Count	: 643 in channel 411
Total Count	: 153591
Background	: 0.597
Decay Scan	: **LT_NMe-TipBPyr2_inert_MeOH_Ex_316_Em_460_250cps_stop1kcounts**
File location	: File has not been saved.
Range (ch)	: 0 to 4095
Peak Count	: 933 in channel 411
Total Count	: 153591
Time Calibration	: 0.024 ns/ch
Total Experiment Time	: 5105.49 s
Fit Range (ch)	: 1 to 1686

Parameter	Value	Std. Dev.	Rel %
τ_1	2.059E-009 s	7.4417E-012 s	
Shift	1.126E-010 s	4.413E-012 s	
B1	0.021	0.0001	100.00
A	0.698		
χ^2	1.021		

Figure S85. Fluorescence lifetime measurements of **2** in MeOH (λ_{ex} = 316 nm, λ_{em} = 460 nm). Top panel: Sample (yellow), instrument response function (blue), mono exponential fit (purple). Middle panel: Weighted residuals and reduced χ^2 for the indicated fit. Bottom: Detailed fit results.

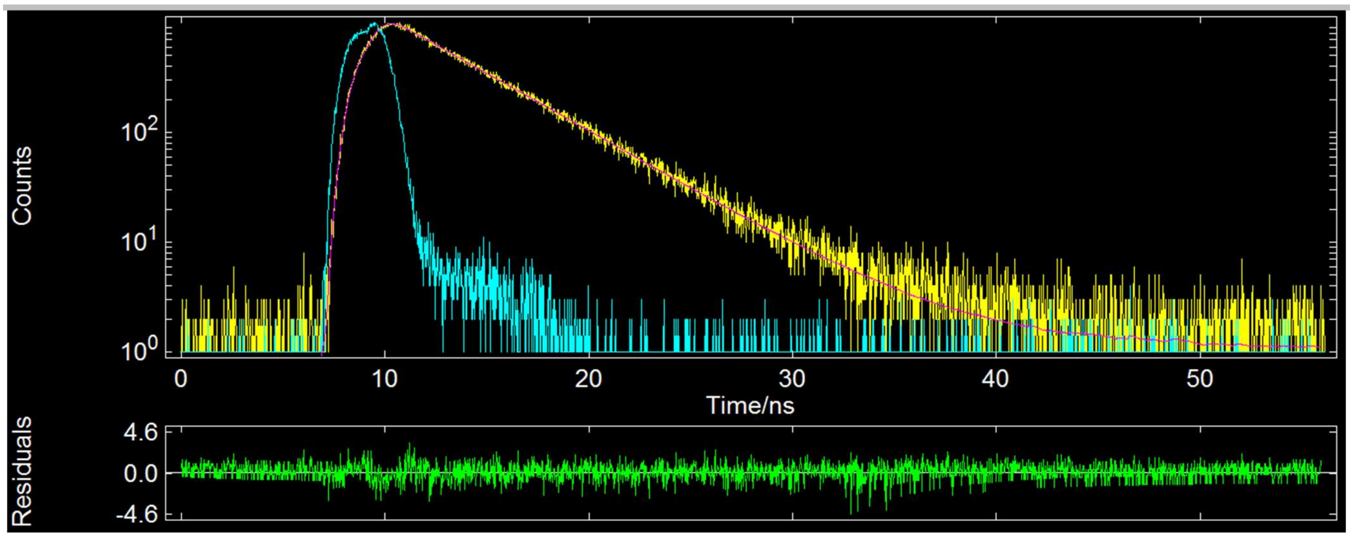

EDINBURGH INSTRUMENTS
F900 23/10/2025

Fit Results

Fit : A+B1exp(-t/τ_1)

Instrument Response	: **IRF_316_THF_stop1kcounts**
Range (ch)	: 0 to 4095
Peak Count	: 173 in channel 439
Total Count	: 244614
Background	: 0.476
Decay Scan	: **LT_NMe-TipBPyr2_inert_THF_Ex_316_Em_445_850cps_stop1kcounts**
File location	: File has not been saved.
Range (ch)	: 0 to 4095
Peak Count	: 898 in channel 439
Total Count	: 244614
Time Calibration	: 0.024 ns/ch
Total Experiment Time	: 2101.42 s
Fit Range (ch)	: 1 to 2292

Parameter	Value	Std. Dev.	Rel %
τ_1	4.060E-009 s	1.0189E-011 s	
Shift	7.082E-011 s	4.442E-012 s	
B1	0.017	0.0001	100.00
A	0.374		
χ^2	0.931		

Figure S86. Fluorescence lifetime measurements of **2** in THF (λ_{ex} = 316 nm, λ_{em} = 445 nm). Top panel: Sample (yellow), instrument response function (blue), mono exponential fit (purple). Middle panel: Weighted residuals and reduced χ^2 for the indicated fit. Bottom: Detailed fit results.

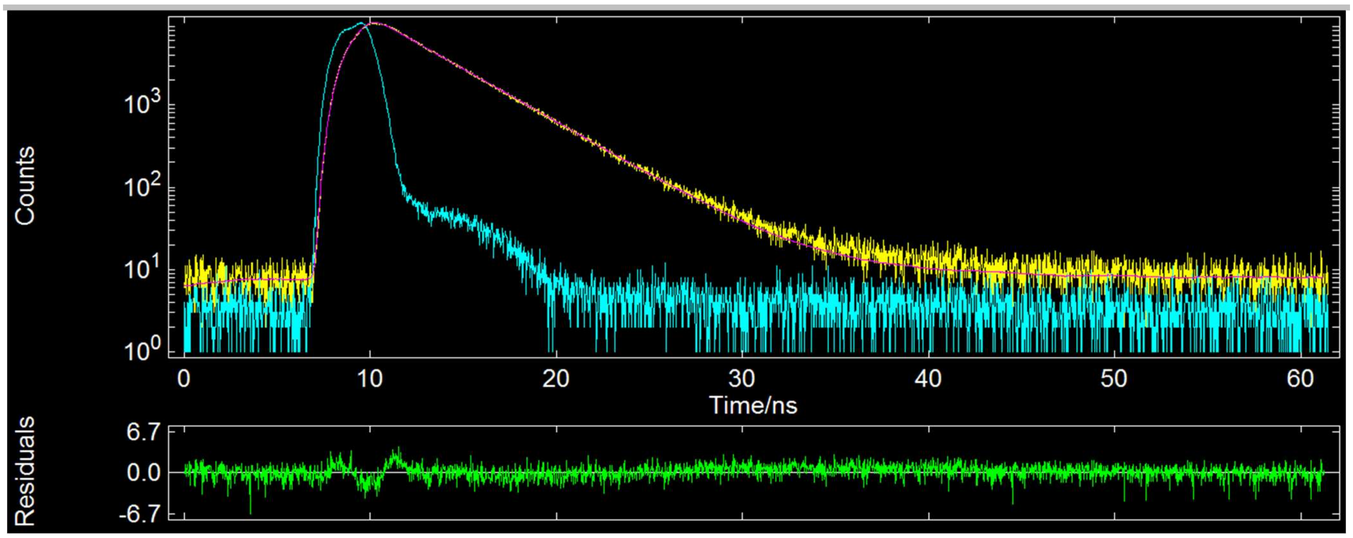

EDINBURGH INSTRUMENTS

F900 23/10/2025

Fit Results

Fit : A+B1exp(-t/τ_1)

Instrument Response	: **IRF_316_Hexan_stop10kcounts**
Range (ch)	: 0 to 4095
Peak Count	: 3499 in channel 427
Total Count	: 2148784
Background	: 3.342
Decay Scan	: **LT_NMe-TipBPyr2_inert_Hexan_Ex_316_Em_400_1600cps_stop10kcounts**
File location	: File has not been saved.
Range (ch)	: 0 to 4095
Peak Count	: 9880 in channel 427
Total Count	: 2148784
Time Calibration	: 0.024 ns/ch
Total Experiment Time	: 9720.30 s
Fit Range (ch)	: 1 to 2509

Parameter	Value	Std. Dev.	Rel %
τ_1	3.287E-009 s	2.7695E-012 s	
Shift	7.550E-011 s	1.354E-012 s	
B1	0.018	0.0000	100.00
A	6.308		
χ^2	1.345		

Figure S87. Fluorescence lifetime measurements of **2** in *n*-hexane (λ_{ex} = 316 nm, λ_{em} = 400 nm). Top panel: Sample (yellow), instrument response function (blue), mono exponential fit (purple). Middle panel: Weighted residuals and reduced χ^2 for the indicated fit. Bottom: Detailed fit results.

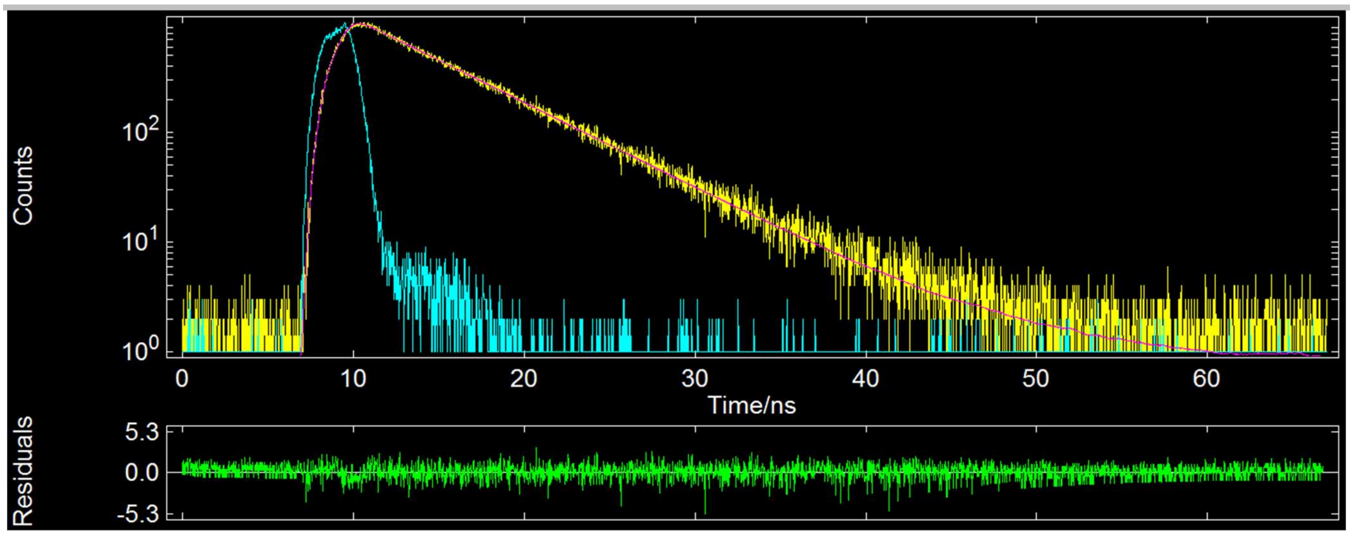

EDINBURGH INSTRUMENTS
F900 23/10/2025

Fit Results

Fit : A+B1exp(-t/τ_1)

Instrument Response	: **IRF_316_MeOH_stop1kcounts**
Range (ch)	: 0 to 4095
Peak Count	: 204 in channel 434
Total Count	: 305407
Background	: 0.383
Decay Scan	: **LT_NH-TipBPyr2_inert_MeOH_Ex_316_Em_465_800cps_stop1kcounts**
File location	: File has not been saved.
Range (ch)	: 0 to 4095
Peak Count	: 927 in channel 434
Total Count	: 305407
Time Calibration	: 0.024 ns/ch
Total Experiment Time	: 2955.15 s
Fit Range (ch)	: 1 to 2735

Parameter	Value	Std. Dev.	Rel %
τ_1	5.515E-009 s	1.2030E-011 s	
Shift	8.838E-011 s	4.394E-012 s	
B1	0.016	0.0000	100.00
A	0.177		
χ^2	0.972		

Figure S88. Fluorescence lifetime measurements of **4** in MeOH (λ_{ex} = 316 nm, λ_{em} = 465 nm). Top panel: Sample (yellow), instrument response function (blue), mono exponential fit (purple). Middle panel: Weighted residuals and reduced χ^2 for the indicated fit. Bottom: Detailed fit results.

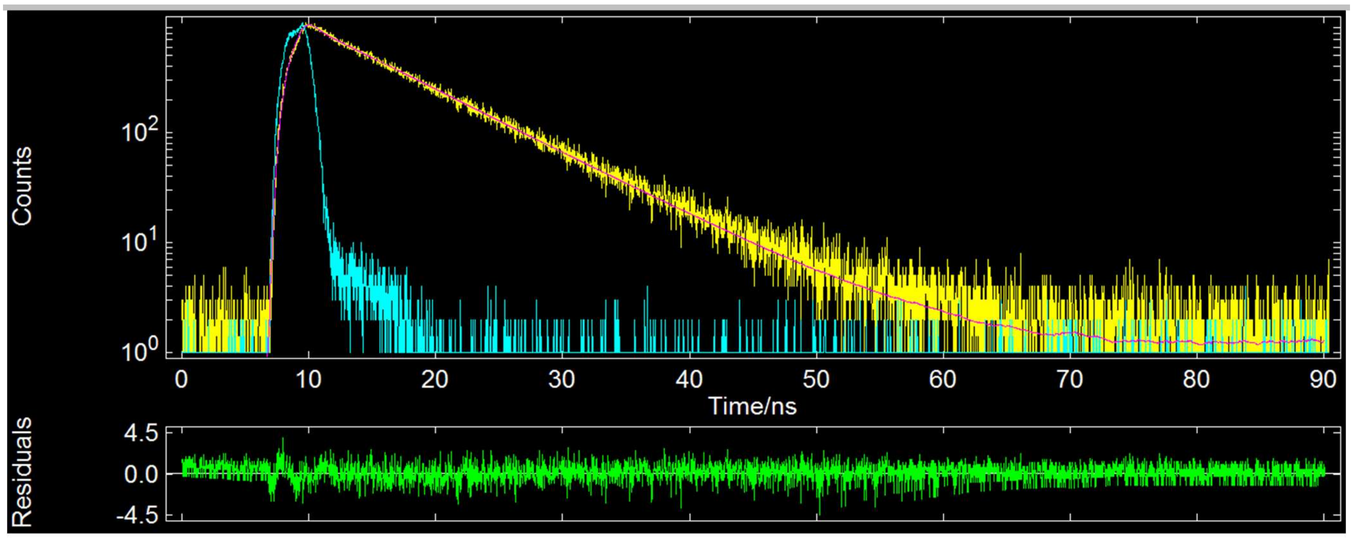

EDINBURGH INSTRUMENTS
F900 23/10/2025

Fit Results

Fit : $A+B1\exp(-t/\tau_1)+B2\exp(-t/\tau_2)$

Instrument Response	: **IRF_316_THF_stop1kcounts**
Range (ch)	: 0 to 4095
Peak Count	: 809 in channel 402
Total Count	: 351720
Background	: 0.466
Decay Scan	: **LT_NH-TipBPyr2_inert_THF_Ex_316_Em_355_1300cps_stop1kcounts**
File location	: File has not been saved.
Range (ch)	: 0 to 4095
Peak Count	: 959 in channel 402
Total Count	: 351720
Time Calibration	: 0.024 ns/ch
Total Experiment Time	: 1938.21 s
Fit Range (ch)	: 1 to 3690

Parameter	Value	Std. Dev.	Rel %
τ_1	4.970E-010 s	6.7093E-011 s	
τ_2	7.476E-009 s	1.8561E-011 s	
Shift	2.322E-010 s	1.288E-011 s	
B1	0.008	0.0012	4.17
B2	0.013	0.0001	95.83
A	0.226		
χ^2	1.000		

Figure S89. Fluorescence lifetime measurements of **4** in THF (λ_{ex} = 316 nm, λ_{em} = 355 nm). Top panel: Sample (yellow), instrument response function (blue), double exponential fit (purple). Middle panel: Weighted residuals and reduced χ^2 for the indicated fit. Bottom: Detailed fit results.

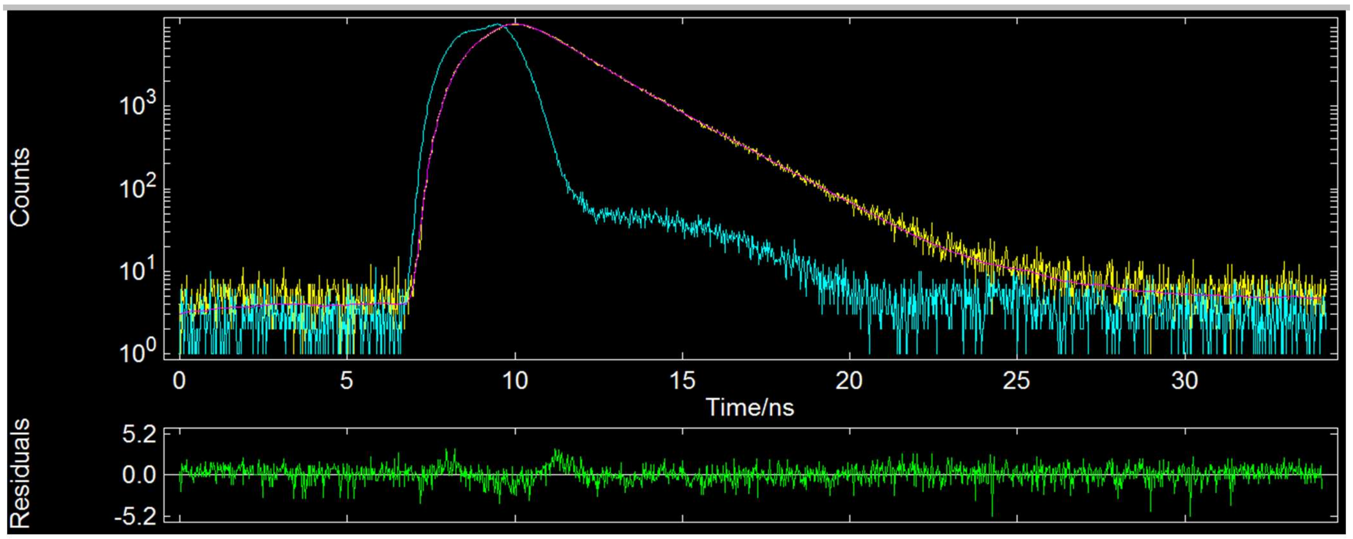

EDINBURGH INSTRUMENTS

F900 23/10/2025

Fit Results

Fit : A+B1exp(-t/τ_1)

Instrument Response : **IRF_316_Hexan_stop10kcounts**
Range (ch) : 0 to 4095
Peak Count : 7128 in channel 408
Total Count : 1471133
Background : 2.824

Decay Scan : **LT_NH-TipBPyr2_inert_Hexan_Ex_316_Em_346_9kcps_stop10kcounts**
File location : File has not been saved.
Range (ch) : 0 to 4095
Peak Count : 9859 in channel 408
Total Count : 1471133

Time Calibration : 0.024 ns/ch
Total Experiment Time : 1098.58 s

Fit Range (ch) : 1 to 1397

Parameter	Value	Std. Dev.	Rel %
τ_1	1.770E-009 s	2.0550E-012 s	
Shift	3.209E-011 s	1.350E-012 s	
B1	0.023	0.0000	100.00
A	2.946		
χ^2	1.175		

Figure S90. Fluorescence lifetime measurements of **4** in *n*-hexane (λ_{ex} = 316 nm, λ_{em} = 346 nm). Top panel: Sample (yellow), instrument response function (blue), mono exponential fit (purple). Middle panel: Weighted residuals and reduced χ^2 for the indicated fit. Bottom: Detailed fit results.

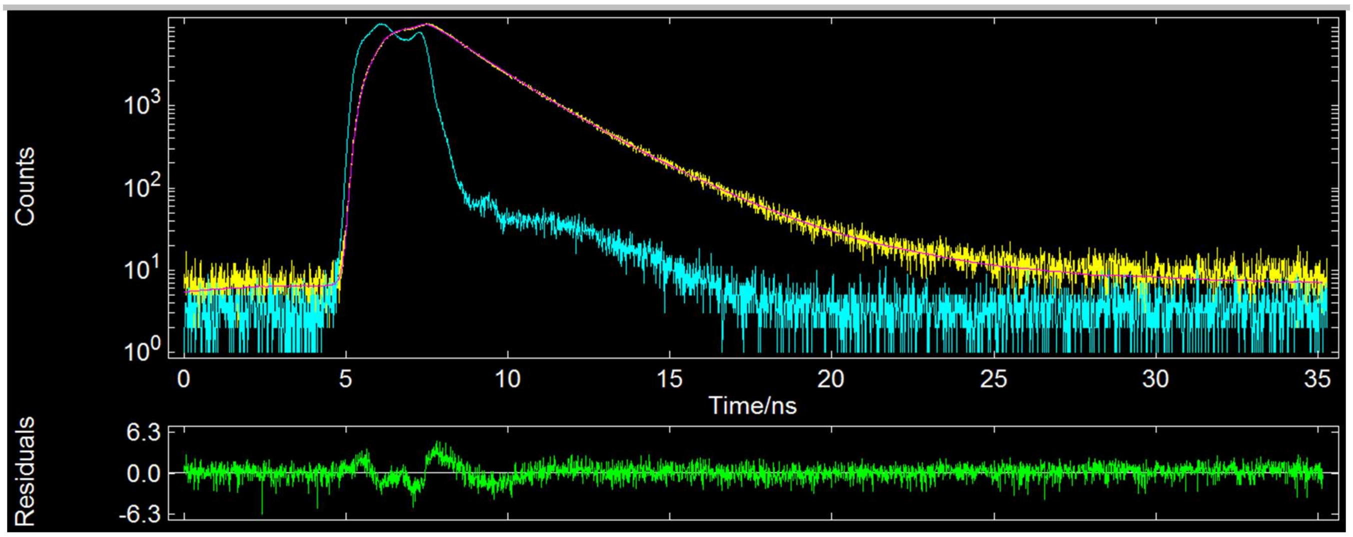

Fit Results

Fit : $A+B1\exp(-t/\tau_1)+B2\exp(-t/\tau_2)$

Instrument Response	: **IRF_377_670kcps_stop10kcounts**
Range (ch)	: 0 to 4095
Peak Count	: 5656 in channel 613
Total Count	: 2681734
Background	: 3.470
Decay Scan	: **LT_DaGo-255_inert_MeOH_Ex_377_Em_539_670kcps_stop10kcounts**
File location	: File has not been saved.
Range (ch)	: 0 to 4095
Peak Count	: 9807 in channel 613
Total Count	: 2681734
Time Calibration	: 0.012 ns/ch
Total Experiment Time	: 46.32 s
Fit Range (ch)	: 1 to 2881

Parameter	Value	Std. Dev.	Rel %
τ_1	1.592E-009 s	4.4235E-012 s	
τ_2	3.899E-009 s	8.9718E-011 s	
Shift	2.635E-011 s	8.841E-013 s	
B1	0.013	0.0000	93.69
B2	0.000	0.0000	6.31
A	5.383		
χ^2	1.463		

Figure S91. Fluorescence lifetime measurements of **7a** in MeOH (λ_{ex} = 377 nm, λ_{em} = 539 nm). Top panel: Sample (yellow), instrument response function (blue), double exponential fit (purple). Middle panel: Weighted residuals and reduced χ^2 for the indicated fit. Bottom: Detailed fit results.

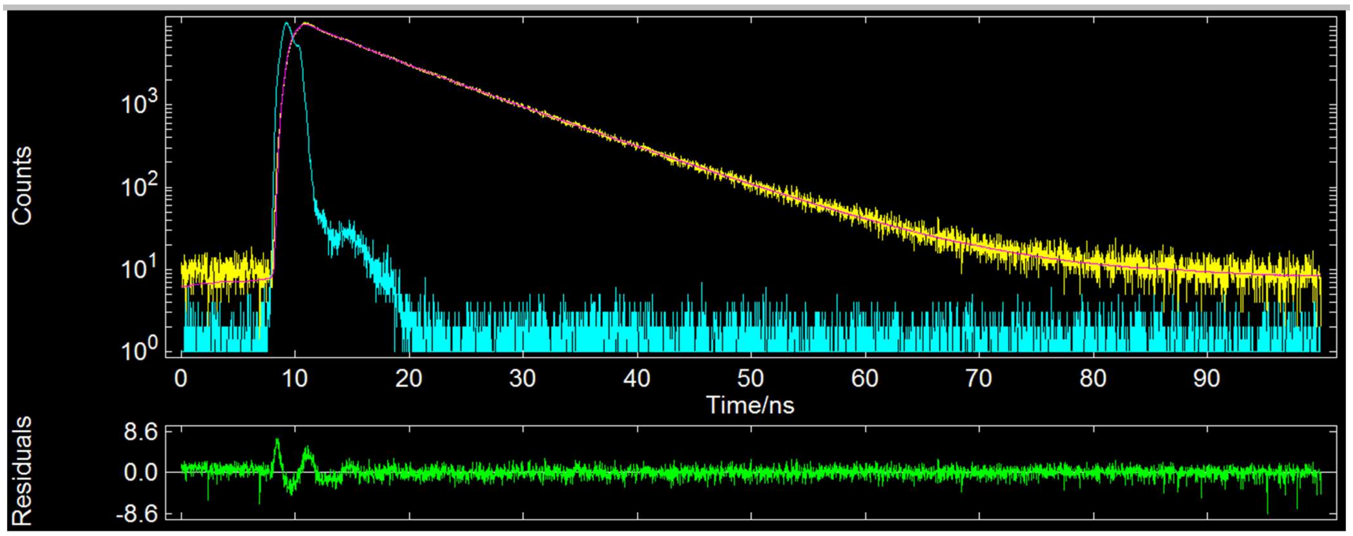

EDINBURGH INSTRUMENTS

F900 23/10/2025

Fit Results

Fit : $A+B1\exp(-t/\tau_1)+B2\exp(-t/\tau_2)$

Instrument Response	: **IRF_377_740kcps_stop10kcounts**
Range (ch)	: 0 to 4095
Peak Count	: 1369 in channel 444
Total Count	: 3774577
Background	: 1.424
Decay Scan	: **LT_DaGo-255_inert_THF_Ex_377_Em_511_740kcps_stop10kcounts**
File location	: File has not been saved.
Range (ch)	: 0 to 4095
Peak Count	: 9837 in channel 444
Total Count	: 3774577
Time Calibration	: 0.024 ns/ch
Total Experiment Time	: 36.89 s
Fit Range (ch)	: 1 to 4095

Parameter	Value	Std. Dev.	Rel %
τ_1	4.124E-009 s	8.7260E-011 s	
τ_2	9.010E-009 s	2.4194E-011 s	
Shift	-9.118E-011 s	1.447E-012 s	
B1	0.005	0.0001	13.84
B2	0.014	0.0001	86.16
A	6.135		
χ^2	1.479		

Figure S92. Fluorescence lifetime measurements of **7a** in THF (λ_{ex} = 377 nm, λ_{em} = 511 nm). Top panel: Sample (yellow), instrument response function (blue), double exponential fit (purple). Middle panel: Weighted residuals and reduced χ^2 for the indicated fit. Bottom: Detailed fit results.

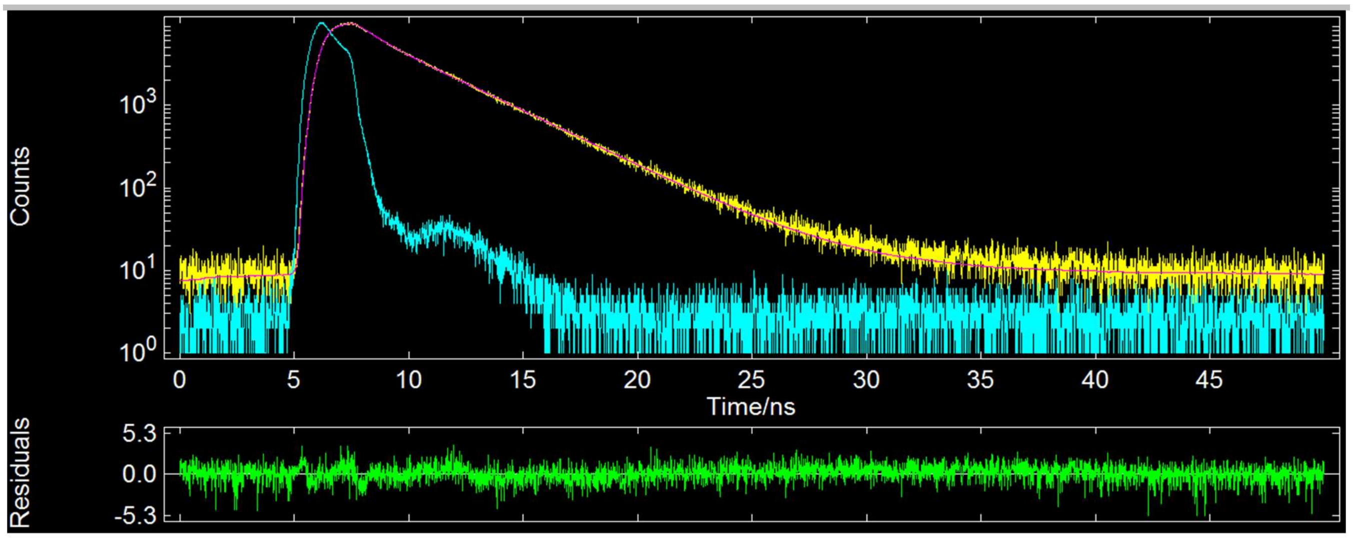

EDINBURGH INSTRUMENTS

F900 23/10/2025

Fit Results

Fit : $A+B1\exp(-t/\tau_1)+B2\exp(-t/\tau_2)$

Instrument Response	: **IRF_377_790kcps_stop10kcounts**
Range (ch)	: 0 to 4095
Peak Count	: 4567 in channel 602
Total Count	: 3459727
Background	: 2.921
Decay Scan	: **LT_DaGo-255_inert_Hexane_Ex_377_Em_445_790kcps_stop10kcounts**
File location	: File has not been saved.
Range (ch)	: 0 to 4095
Peak Count	: 9709 in channel 602
Total Count	: 3459727
Time Calibration	: 0.012 ns/ch
Total Experiment Time	: 52.11 s
Fit Range (ch)	: 1 to 4095

Parameter	Value	Std. Dev.	Rel %
τ_1	8.332E-010 s	1.3711E-011 s	
τ_2	3.218E-009 s	4.5701E-012 s	
Shift	-4.368E-011 s	1.276E-012 s	
B1	0.006	0.0001	13.77
B2	0.009	0.0000	86.23
A	7.516		
χ^2	1.225		

Figure S93. Fluorescence lifetime measurements of **7a** in *n*-hexane (λ_{ex} = 377 nm, λ_{em} = 445 nm). Top panel: Sample (yellow), instrument response function (blue), double exponential fit (purple). Middle panel: Weighted residuals and reduced χ^2 for the indicated fit. Bottom: Detailed fit results.

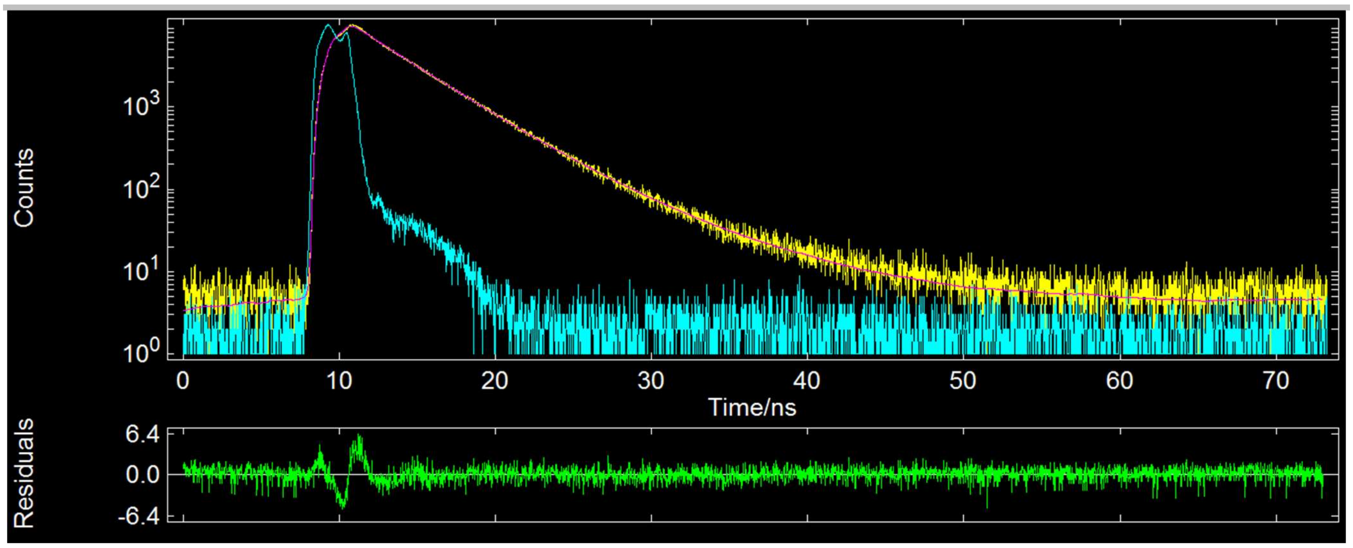

EDINBURGH INSTRUMENTS
F900 23/10/2025

Fit Results

Fit : A+B1exp(-t/τ_1)+B2exp(-t/τ_2)

Instrument Response	: **IRF_377_480kcps_stop10kcounts_100ns**
Range (ch)	: 0 to 4095
Peak Count	: 2660 in channel 446
Total Count	: 2073459
Background	: 1.880
Decay Scan	: **LT_DaGo-288_inert_MeOH_Ex_377_Em_520_480kcps_stop10kcounts_100n**
File location	: File has not been saved.
Range (ch)	: 0 to 4095
Peak Count	: 9893 in channel 446
Total Count	: 2073459
Time Calibration	: 0.024 ns/ch
Total Experiment Time	: 68.47 s
Fit Range (ch)	: 1 to 2991

Parameter	Value	Std. Dev.	Rel %
τ_1	3.243E-009 s	1.7843E-011 s	
τ_2	6.042E-009 s	1.1959E-010 s	
Shift	4.601E-011 s	1.355E-012 s	
B1	0.017	0.0001	84.00
B2	0.002	0.0002	16.00
A	3.314		
χ^2	1.463		

Figure S94. Fluorescence lifetime measurements of **7b** in MeOH (λ_{ex} = 377 nm, λ_{em} = 520 nm). Top panel: Sample (yellow), instrument response function (blue), double exponential fit (purple). Middle panel: Weighted residuals and reduced χ^2 for the indicated fit. Bottom: Detailed fit results.

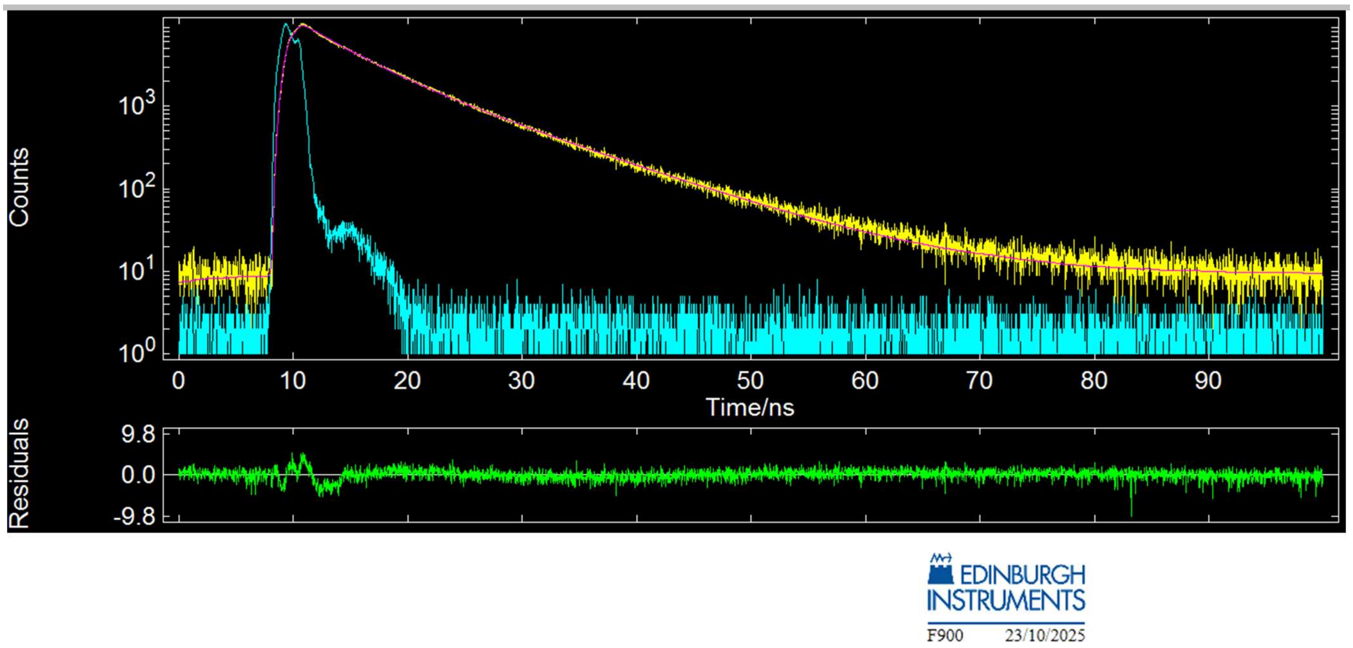

Fit Results

Fit : A+B1exp(-t/τ_1)+B2exp(-t/τ_2)

Instrument Response	: **IRF_377_720kcps_stop10kcounts_100ns**
Range (ch)	: 0 to 4095
Peak Count	: 2658 in channel 444
Total Count	: 3085619
Background	: 1.829
Decay Scan	: **LT_DaGo-288_inert_THF_Ex_377_Em_501_720kcps_stop10kcounts_100ns**
File location	: File has not been saved.
Range (ch)	: 0 to 4095
Peak Count	: 9808 in channel 444
Total Count	: 3085619
Time Calibration	: 0.024 ns/ch
Total Experiment Time	: 69.83 s
Fit Range (ch)	: 1 to 4095

Parameter	Value	Std. Dev.	Rel %
τ_1	3.820E-009 s	2.6086E-011 s	
τ_2	9.180E-009 s	3.1213E-011 s	
Shift	4.424E-011 s	1.410E-012 s	
B1	0.012	0.0001	39.79
B2	0.007	0.0001	60.21
A	7.348		
χ^2	1.544		

Figure S95. Fluorescence lifetime measurements of **7b** in THF (λ_{ex} = 377 nm, λ_{em} = 501 nm). Top panel: Sample (yellow), instrument response function (blue), double exponential fit (purple). Middle panel: Weighted residuals and reduced χ^2 for the indicated fit. Bottom: Detailed fit results.

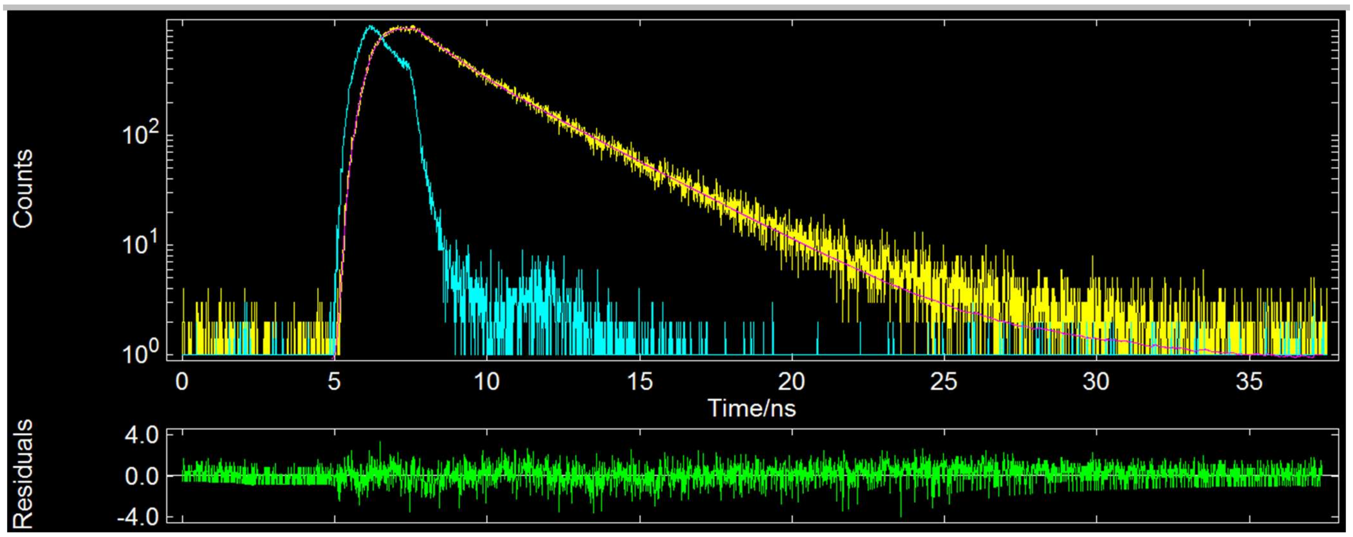

EDINBURGH INSTRUMENTS

F900 23/10/2025

Fit Results

Fit : A+B1exp(-t/τ_1)+B2exp(-t/τ_2)

Instrument Response	: **IRF_377_740kcps_stop1kcounts_50ns**
Range (ch)	: 0 to 4095
Peak Count	: 527 in channel 581
Total Count	: 308334
Background	: 0.464
Decay Scan	: **LT_DaGo-288_inert_Hexane_Ex_377_Em_430_740kcps_stop1kcounts_50ns**
File location	: File has not been saved.
Range (ch)	: 0 to 4095
Peak Count	: 997 in channel 581
Total Count	: 308334
Time Calibration	: 0.012 ns/ch
Total Experiment Time	: 4.93 s
Fit Range (ch)	: 1 to 3066

Parameter	Value	Std. Dev.	Rel %
τ_1	1.055E-009 s	3.7831E-011 s	
τ_2	2.982E-009 s	2.3255E-011 s	
Shift	-8.839E-012 s	3.532E-012 s	
B1	0.008	0.0002	26.34
B2	0.008	0.0002	73.66
A	0.485		
χ^2	0.949		

Figure S96. Fluorescence lifetime measurements of **7b** in *n*-hexane (λ_{ex} = 377 nm, λ_{em} = 430 nm). Top panel: Sample (yellow), instrument response function (blue), double exponential fit (purple). Middle panel: Weighted residuals and reduced χ^2 for the indicated fit. Bottom: Detailed fit results.

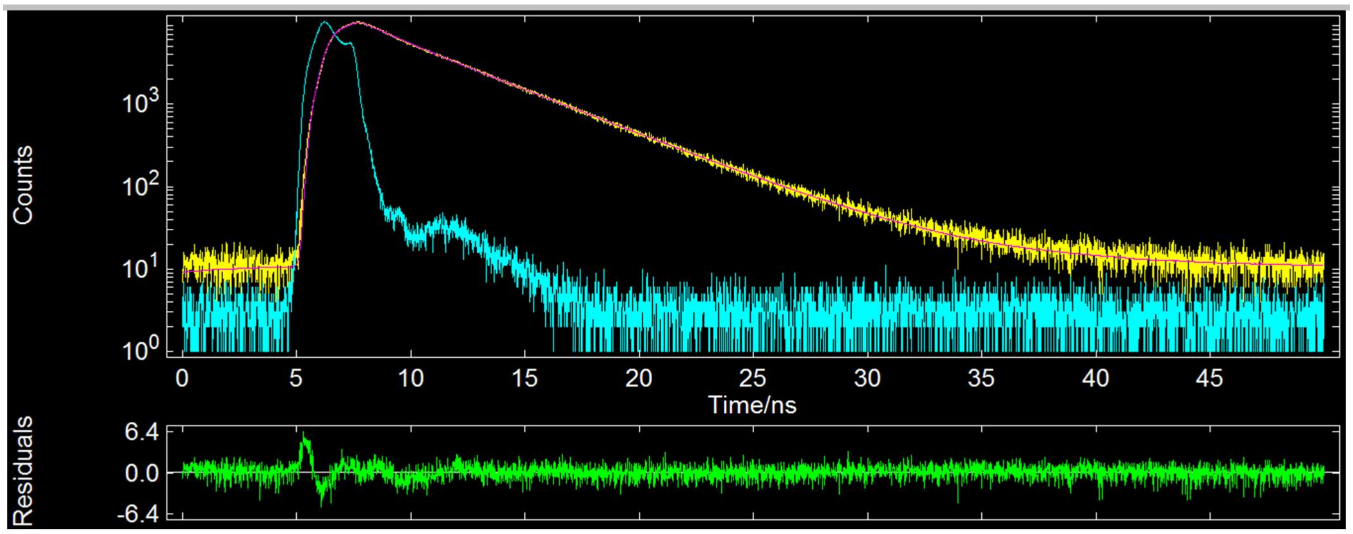

EDINBURGH INSTRUMENTS

F900 23/10/2025

Fit Results

Fit : $A+B1\exp(-t/\tau_1)+B2\exp(-t/\tau_2)$

Instrument Response	: **IRF_377_750kcps_stop10kcounts**
Range (ch)	: 0 to 4095
Peak Count	: 3112 in channel 625
Total Count	: 4231017
Background	: 3.173
Decay Scan	: **LT_DaGo-289_inert_THF_Ex_377_Em_495_750kcps_stop10kcounts**
File location	: File has not been saved.
Range (ch)	: 0 to 4095
Peak Count	: 9734 in channel 625
Total Count	: 4231017
Time Calibration	: 0.012 ns/ch
Total Experiment Time	: 67.06 s
Fit Range (ch)	: 1 to 4095

Parameter	Value	Std. Dev.	Rel %
τ_1	1.672E-009 s	4.5044E-011 s	
τ_2	4.034E-009 s	8.8254E-012 s	
Shift	-8.142E-011 s	1.186E-012 s	
B1	0.003	0.0001	10.49
B2	0.009	0.0001	89.51
A	9.213		
χ^2	1.338		

Figure S97. Fluorescence lifetime measurements of **7c** in THF (λ_{ex} = 377 nm, λ_{em} = 495 nm). Top panel: Sample (yellow), instrument response function (blue), double exponential fit (purple). Middle panel: Weighted residuals and reduced χ^2 for the indicated fit. Bottom: Detailed fit results.

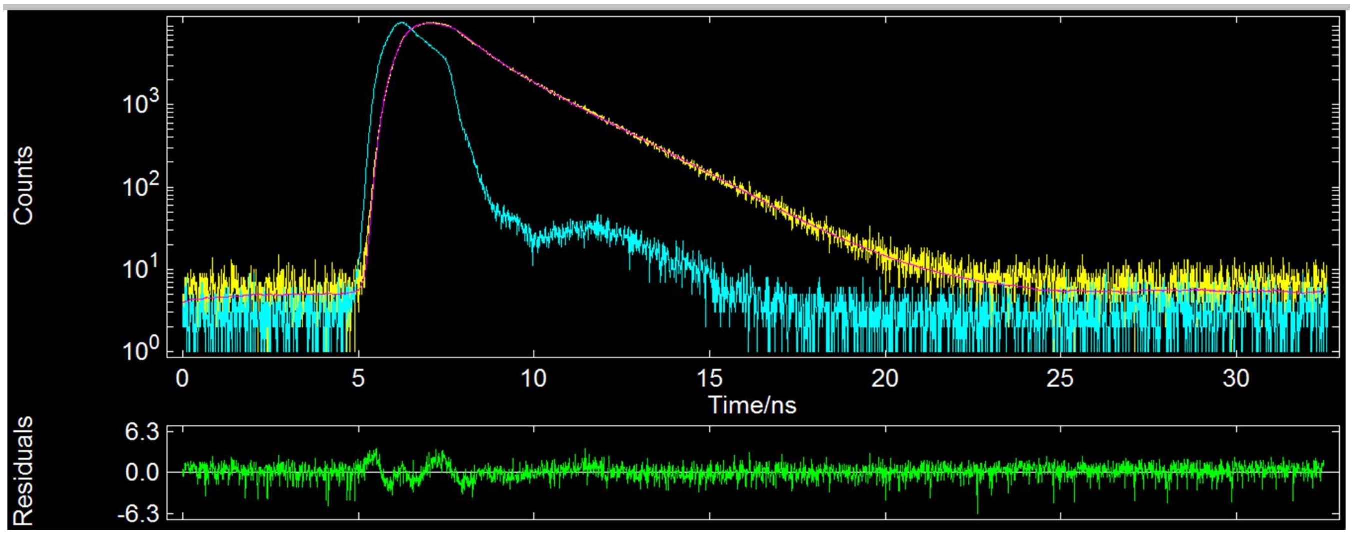

Fit Results

Fit : A+B1exp(-t/τ_1)+B2exp(-t/τ_2)

Instrument Response	: **IRF_377_730kcps_stop10kcounts**
Range (ch)	: 0 to 4095
Peak Count	: 5291 in channel 576
Total Count	: 2336142
Background	: 3.122
Decay Scan	: **LT_DaGo-289_inert_Hexane_Ex_377_Em_433_730kcps_stop10kcounts**
File location	: File has not been saved.
Range (ch)	: 0 to 4095
Peak Count	: 9804 in channel 576
Total Count	: 2336142
Time Calibration	: 0.012 ns/ch
Total Experiment Time	: 37.65 s
Fit Range (ch)	: 1 to 2661

Parameter	Value	Std. Dev.	Rel %
τ_1	5.235E-010 s	7.0957E-012 s	
τ_2	1.842E-009 s	4.3311E-012 s	
Shift	-8.112E-011 s	1.291E-012 s	
B1	0.014	0.0001	29.89
B2	0.009	0.0001	70.11
A	3.931		
χ^2	1.337		

Figure S98. Fluorescence lifetime measurements of **7c** in *n*-hexane (λ_{ex} = 377 nm, λ_{em} = 433 nm). Top panel: Sample (yellow), instrument response function (blue), double exponential fit (purple). Middle panel: Weighted residuals and reduced χ^2 for the indicated fit. Bottom: Detailed fit results.

1.6.3 Aggregation emission enhancement

Table S4. Emission maxima of **2** and **4** in THF with different water fractions.

THF / water	100 / 0	40 / 60	15 / 85	10 / 90
λ_{max} (em.) of 2 [nm]	445	466	442	439
λ_{max} (em.) of 4 [nm]	359, 432	358, 467	359, 398	360, 397

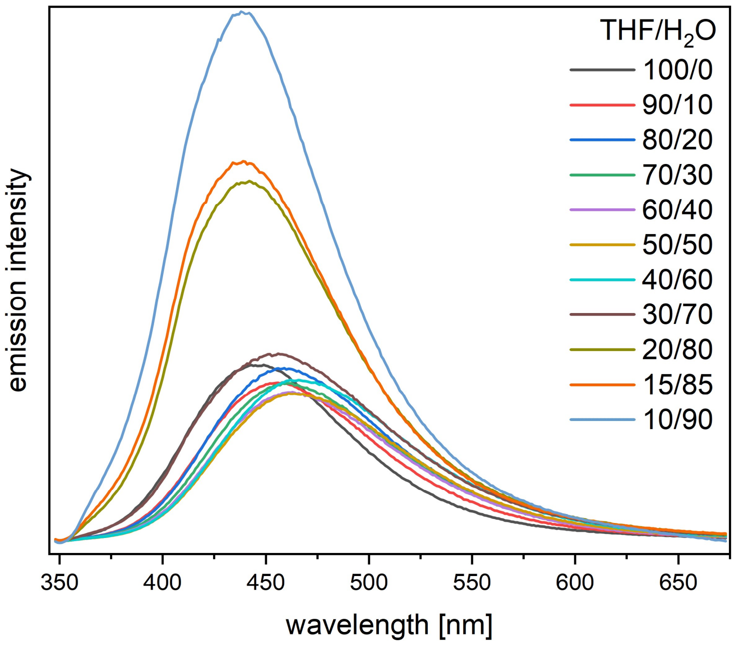

Figure S99. Emission spectra of **2** in THF/ water mixtures (conc. 5·10^{-5} M) with different water fractions (0 – 90%).

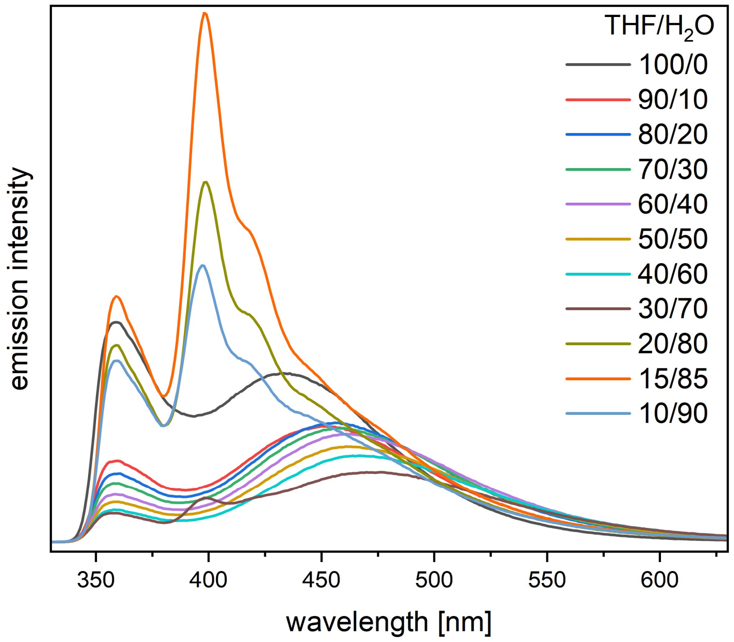

Figure S100. Emission spectra of **4** in THF/ water mixtures (conc. 5·10^{-5} M) with different water fractions (0 – 90%).

1.7 Dynamic light scattering

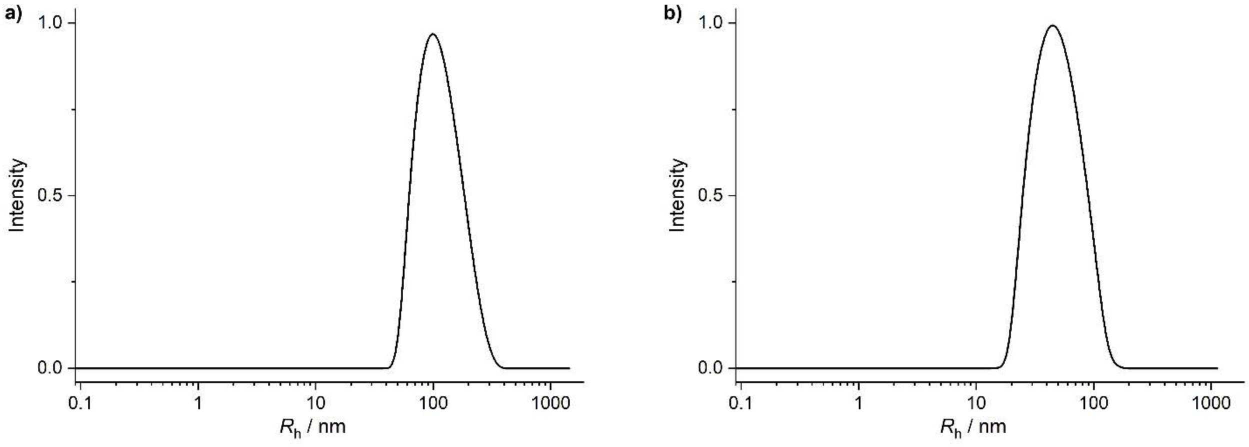

Figure S101. Normalized intensity distribution (unweighted) of aggregates of **2** (a) and **4** (b) in an aqueous solution (THF/water: 10/90).

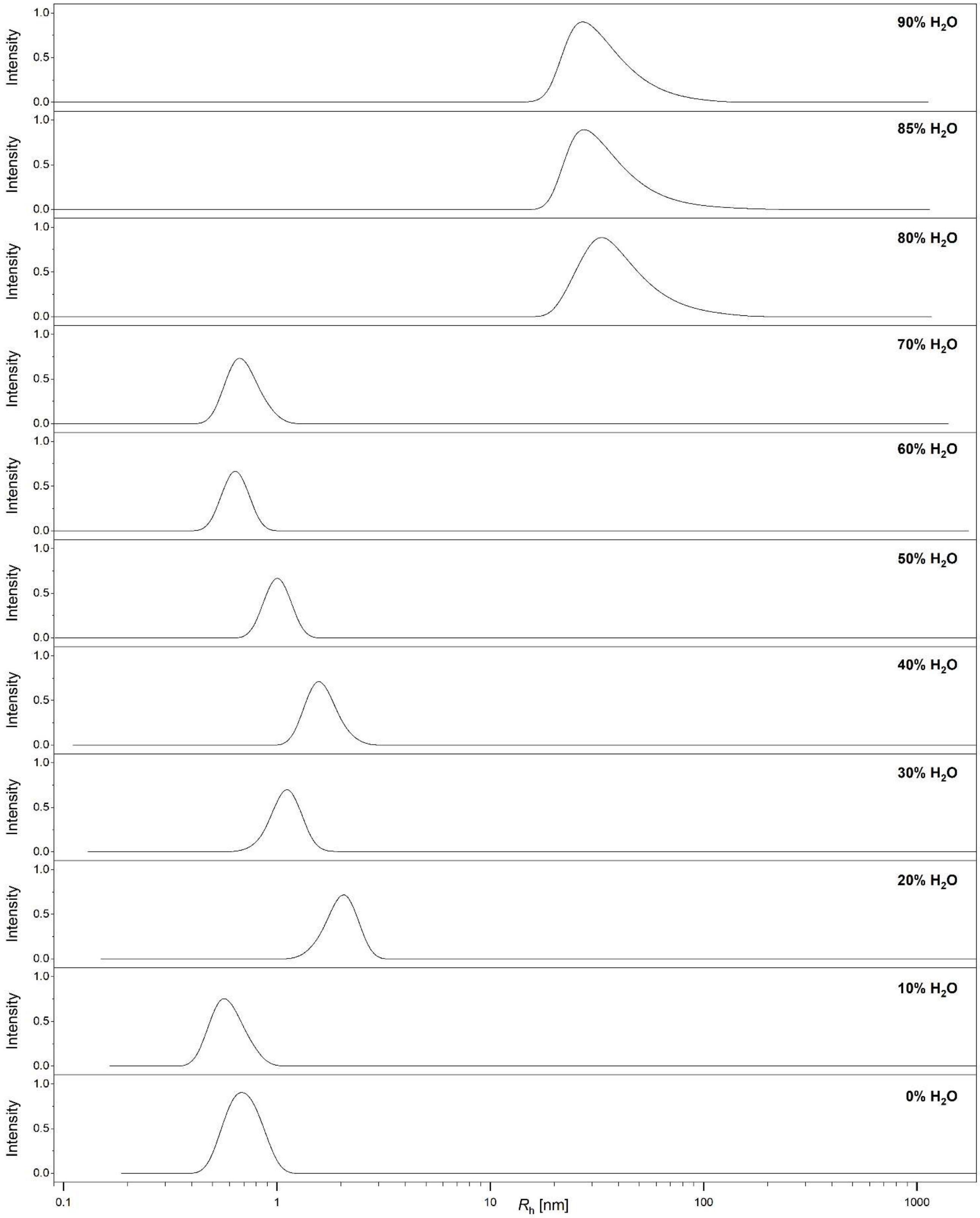

Figure S102. Mass weight distribution (log) of aggregates of **4** in THF/water mixtures (conc. $5 \cdot 10^{-5}$ M) with different water fraction (0 – 90%).

1.8 Cyclic Voltammetry

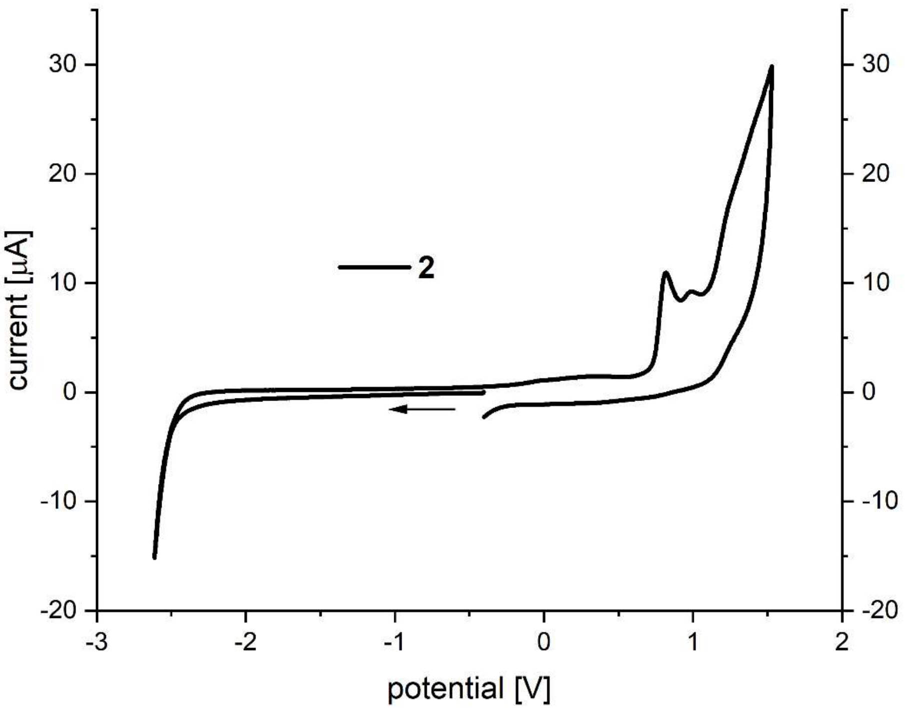

Figure S103. Cyclic voltammogram of **2** in DCM (vs. $[Cp_2Fe]^{0/+}$, scan rate: 250 mVs^{-1}).

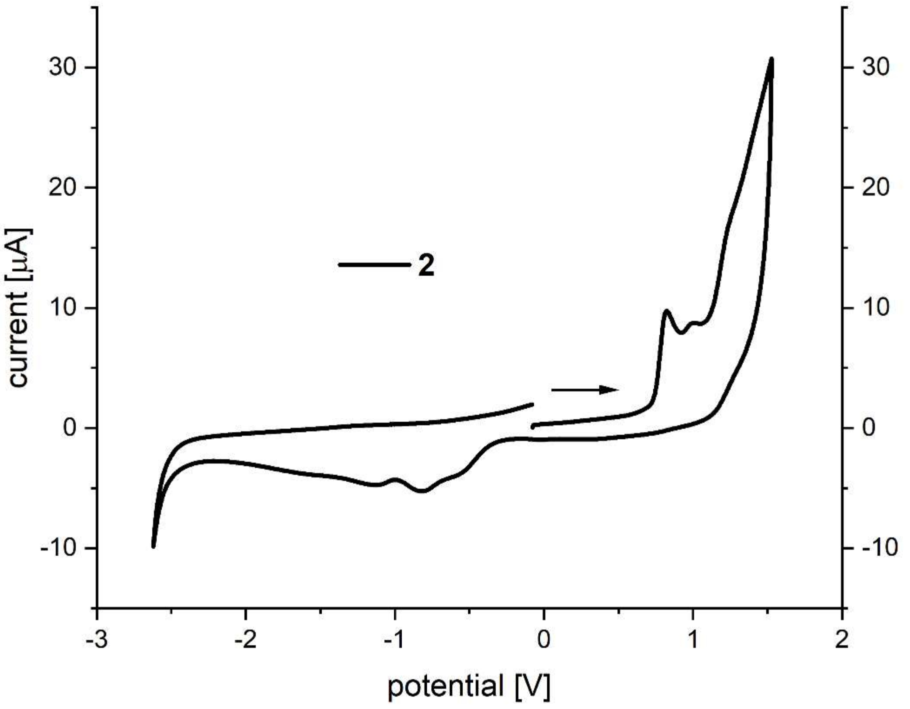

Figure S104. Cyclic voltammogram of **2** in DCM (vs. $[Cp_2Fe]^{0/+}$, scan rate: 250 mVs^{-1}).

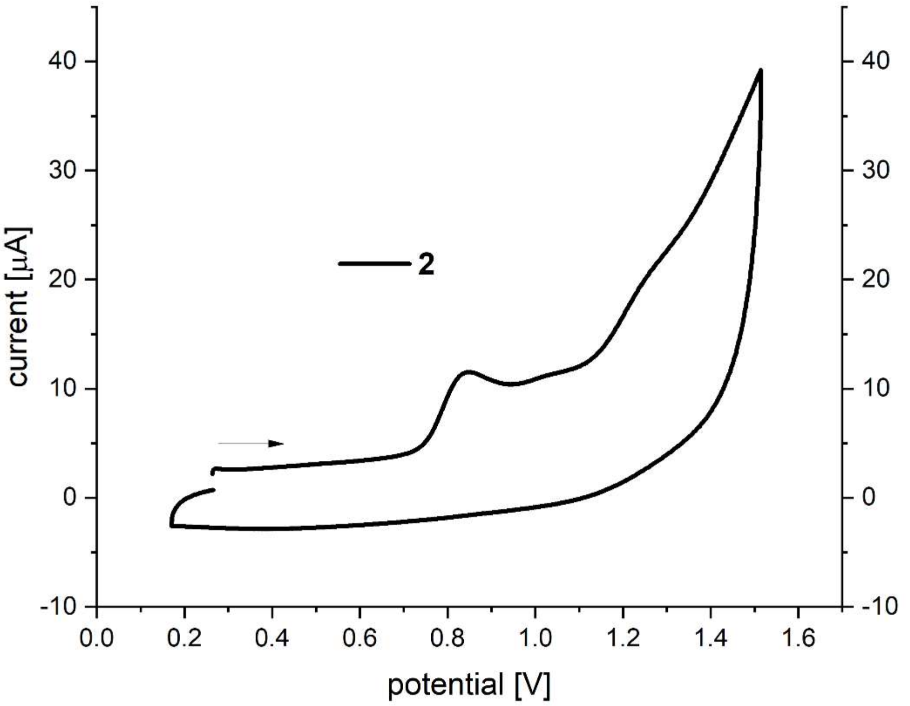

Figure S105. Cyclic voltammogram of the oxidation event of **2** in DCM (vs. $[Cp_2Fe]^{0/+}$, scan rate: 250 mVs^{-1}).

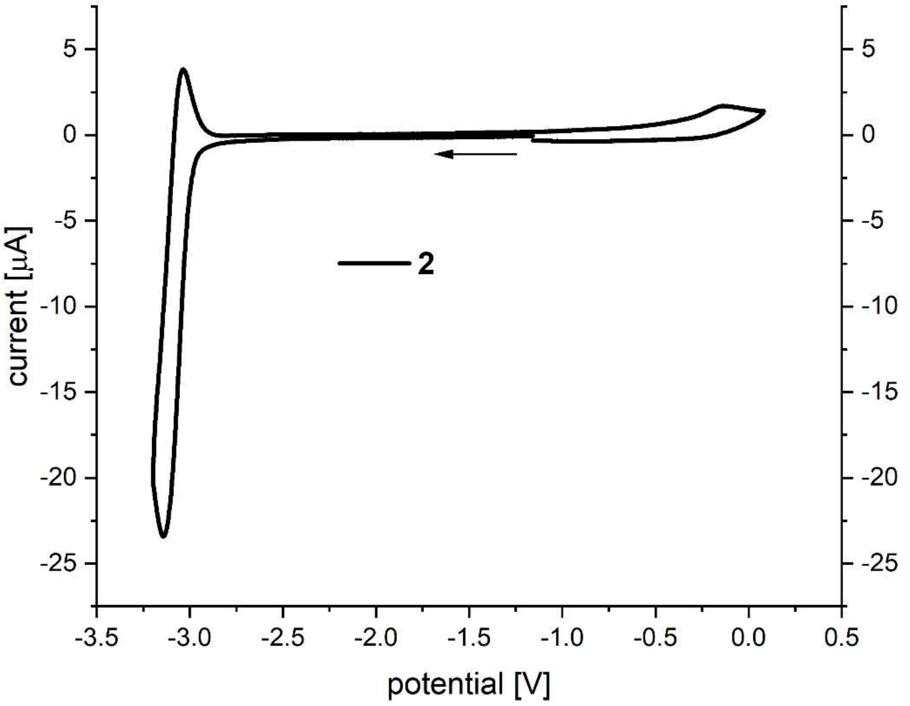

Figure S106. Cyclic voltammogram of **2** in THF (vs. $[Cp_2Fe]^{0/+}$, scan rate: 250 mVs^{-1}).

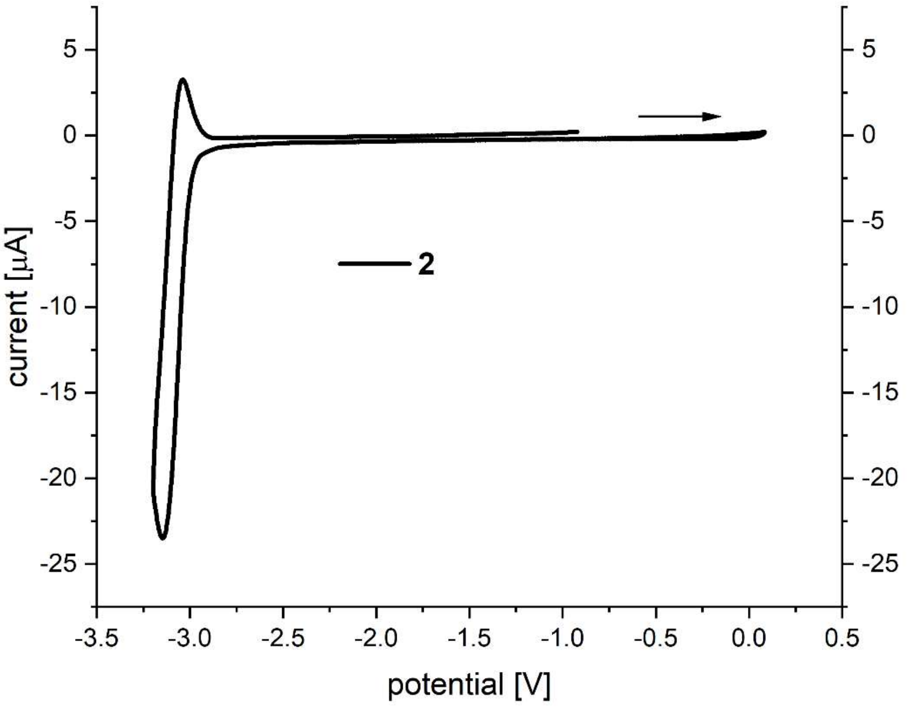

Figure S107. Cyclic voltammogram of **2** in THF (vs. $[Cp_2Fe]^{0/+}$, scan rate: 250 mVs^{-1}).

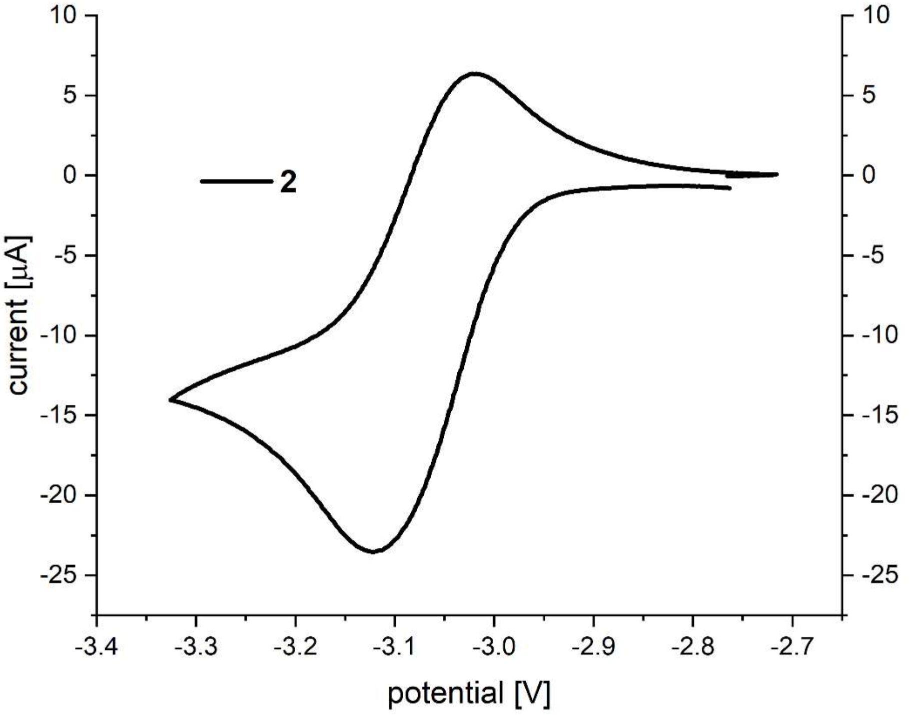

Figure S108. Cyclic voltammogram of the reduction event of **2** in THF (vs. $[Cp_2Fe]^{0/+}$, scan rate: 250 mVs^{-1}).

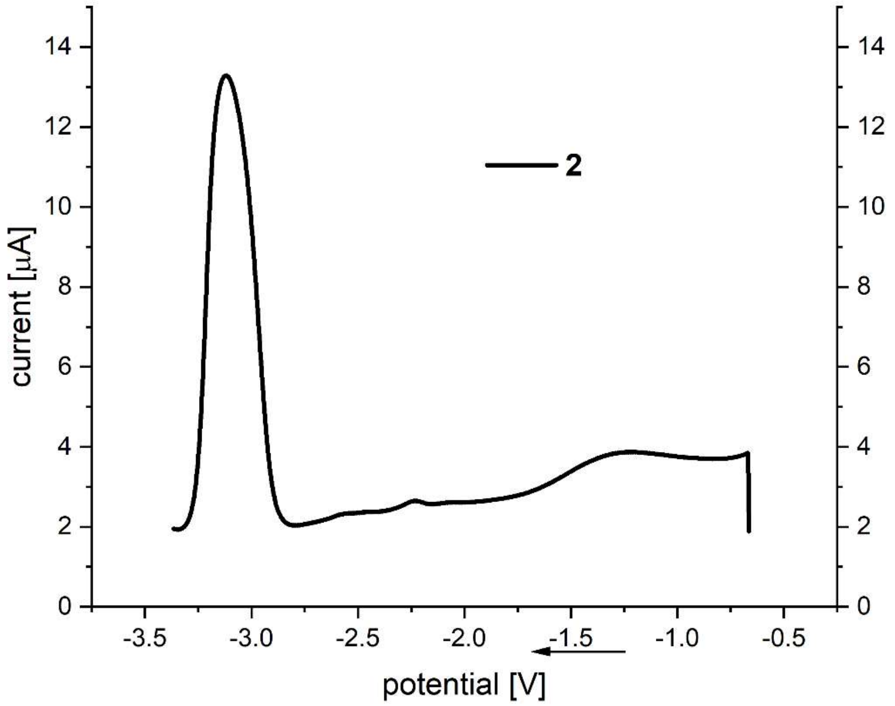

Figure S109. Square wave voltammogram of **2** in THF (vs. $[Cp_2Fe]^{0/+}$).

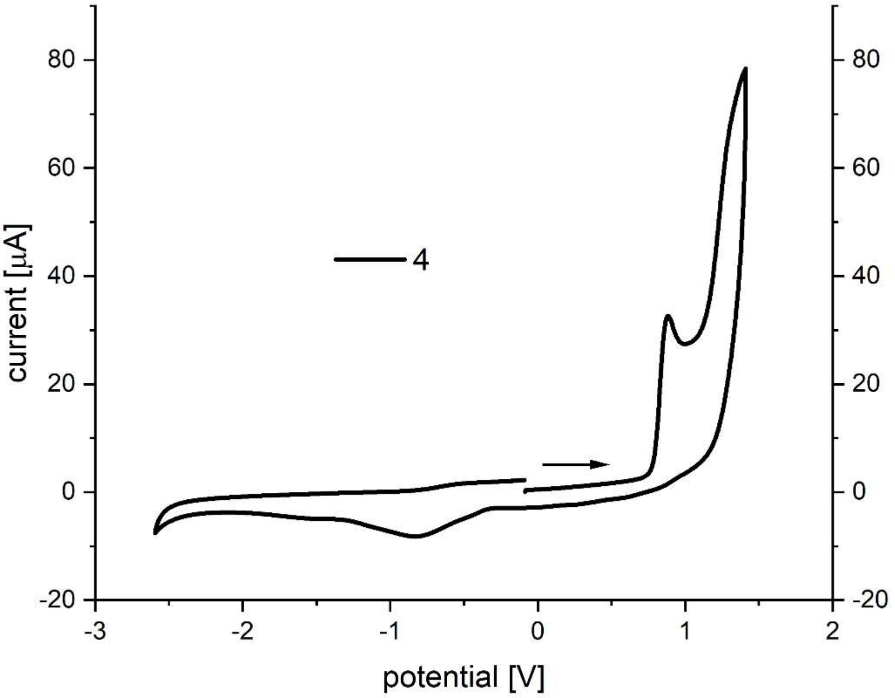

Figure S110. Cyclic voltammogram of **4** in DCM (vs. $[Cp_2Fe]^{0/+}$, scan rate: 250 mVs^{-1}).

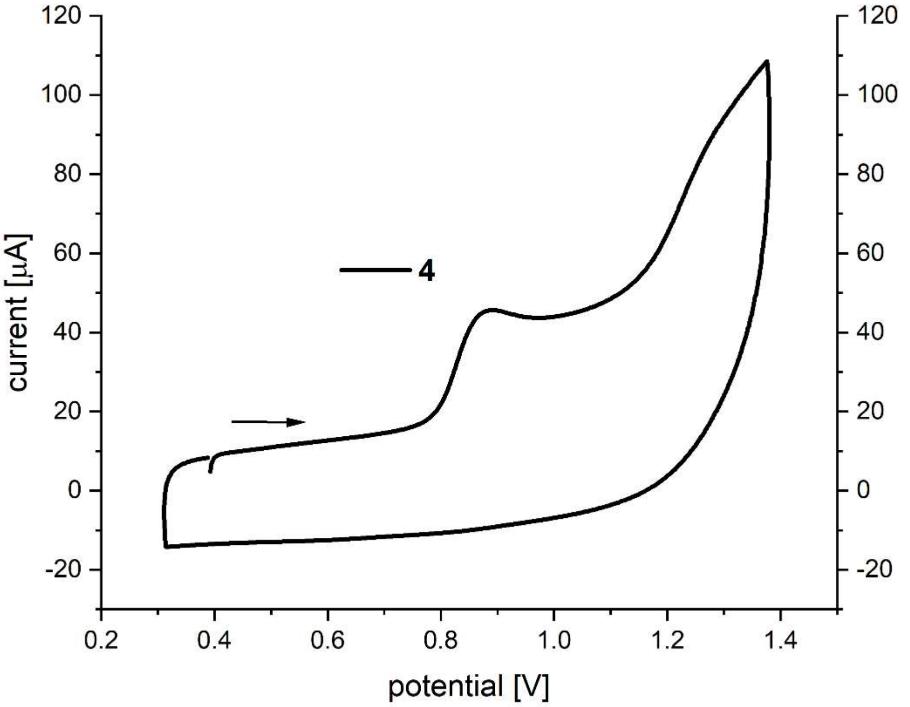

Figure S111. Cyclic voltammogram of the oxidation event of **4** in DCM (vs. $[Cp_2Fe]^{0/+}$, scan rate: 250 mVs^{-1}).

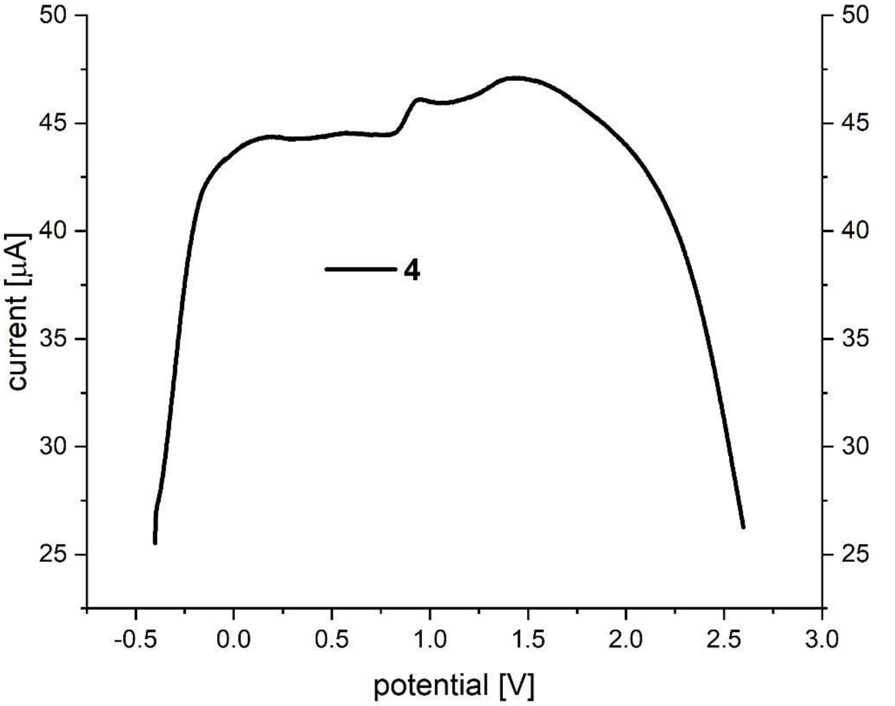

Figure S112. Square wave voltammogram of **4** in DCM (vs. $[Cp_2Fe]^{0/+}$).

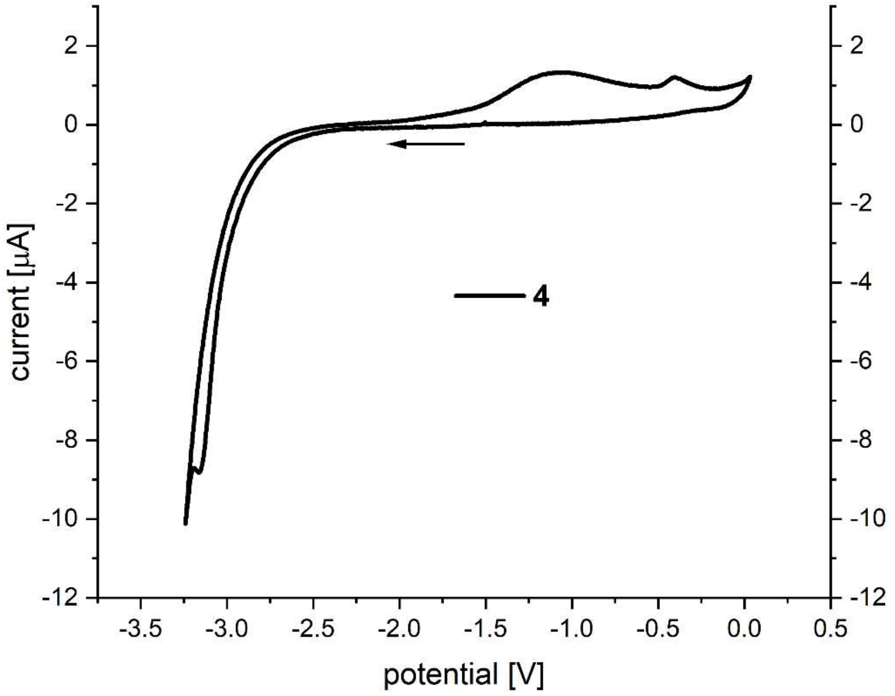

Figure S113. Cyclic voltammogram of **4** in THF (vs. $[Cp_2Fe]^{0/+}$, scan rate: 150 mVs^{-1}).

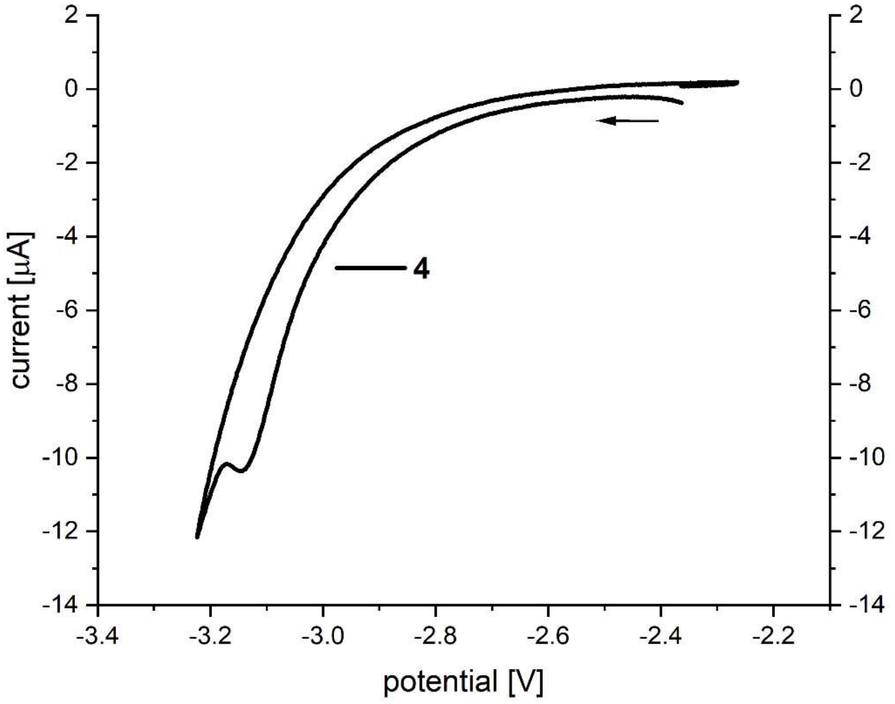

Figure S114. Cyclic voltammogram of the reduction event of **4** in THF (vs. $[Cp_2Fe]^{0/+}$, scan rate: 250 mVs^{-1}).

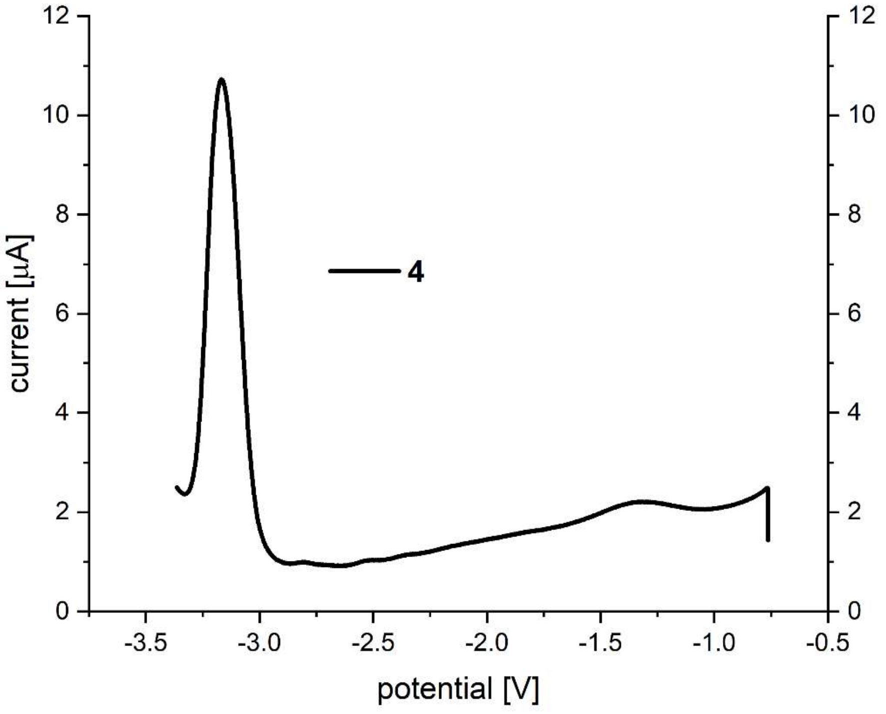

Figure S115. Square wave voltammogram of **4** in THF (vs. $[Cp_2Fe]^{0/+}$).

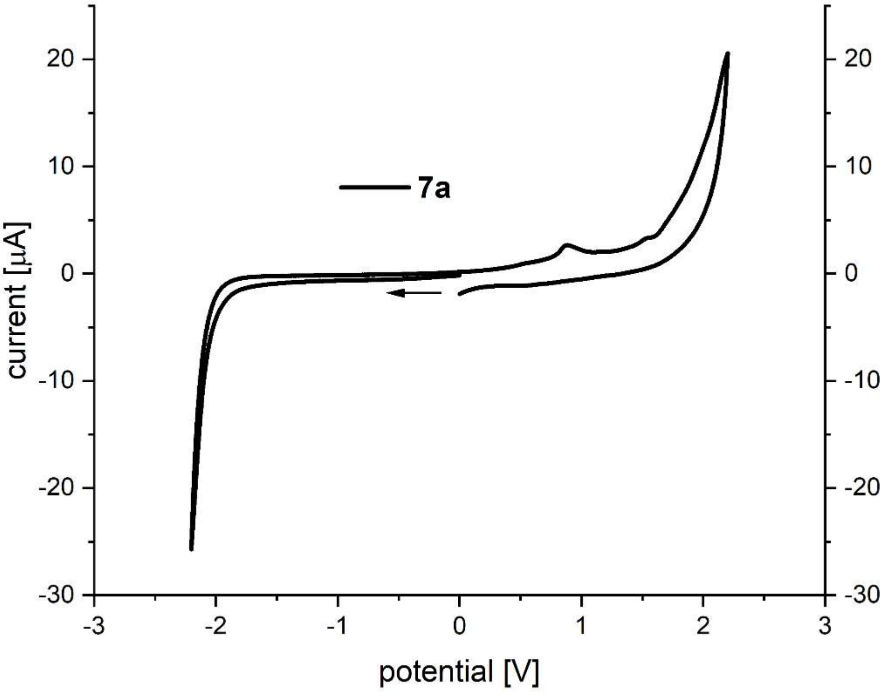

Figure S116. Cyclic voltammogram of **7a** in DCM (vs. $[Cp_2Fe]^{0/+}$, scan rate: 250 mVs^{-1}).

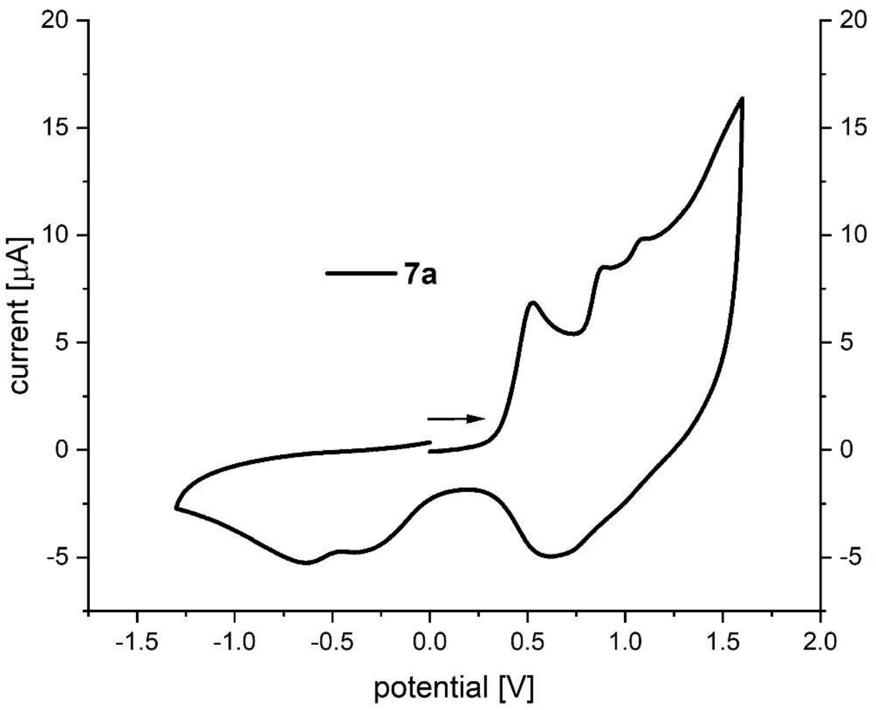

Figure S117. Cyclic voltammogram of the oxidation event of **7a** in DCM (vs. $[Cp_2Fe]^{0/+}$, scan rate: 250 mVs^{-1}).

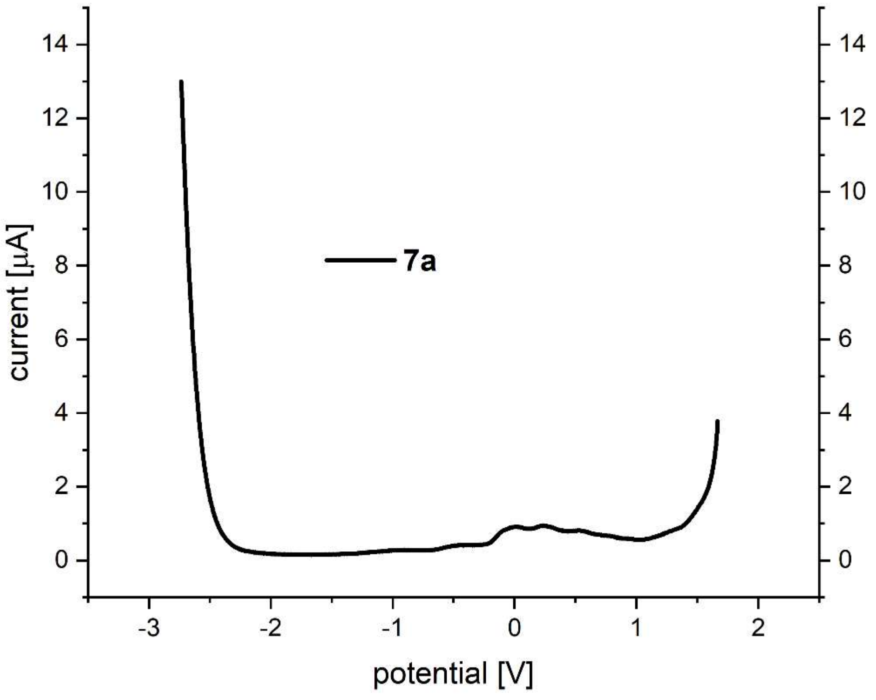

Figure S118. Square wave voltammogram of **7a** in DCM (vs. $[Cp_2Fe]^{0/+}$).

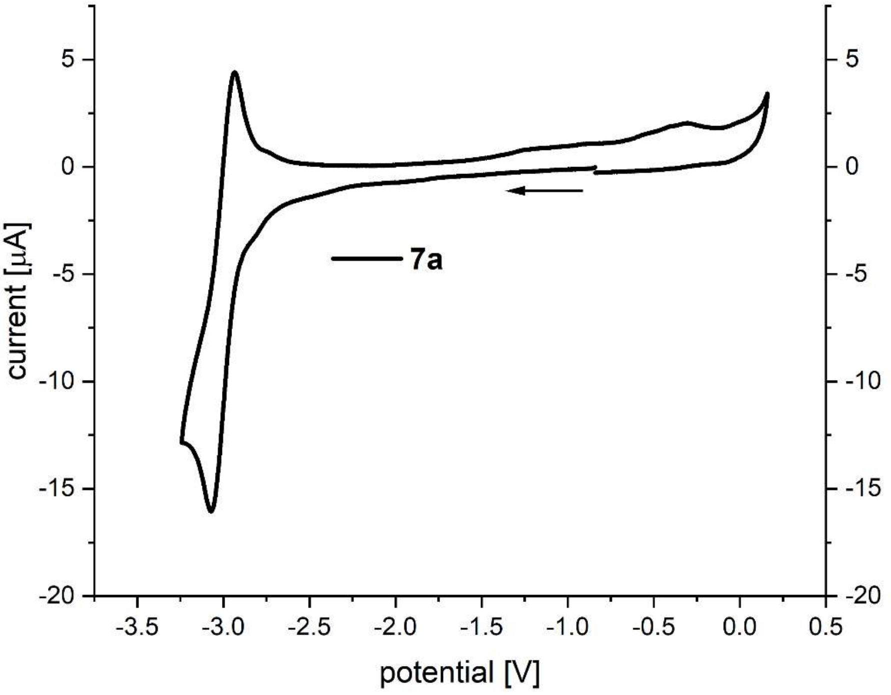

Figure S119. Cyclic voltammogram of **7a** in THF (vs. $[Cp_2Fe]^{0/+}$, scan rate: 250 mVs^{-1}).

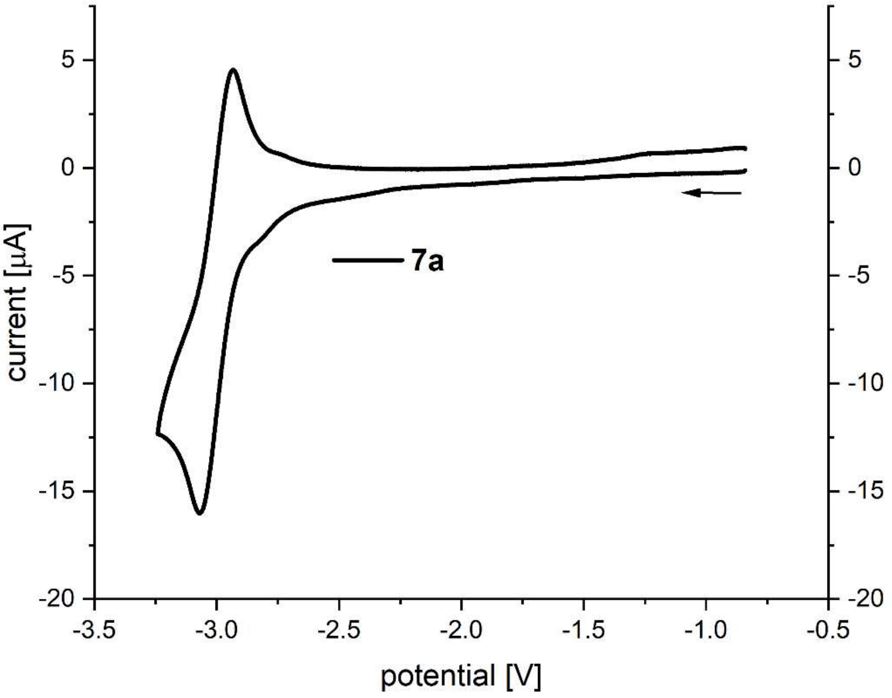

Figure S120. Cyclic voltammogram of the reduction event of **7a** in THF (vs. $[Cp_2Fe]^{0/+}$, scan rate: 250 mVs^{-1}).

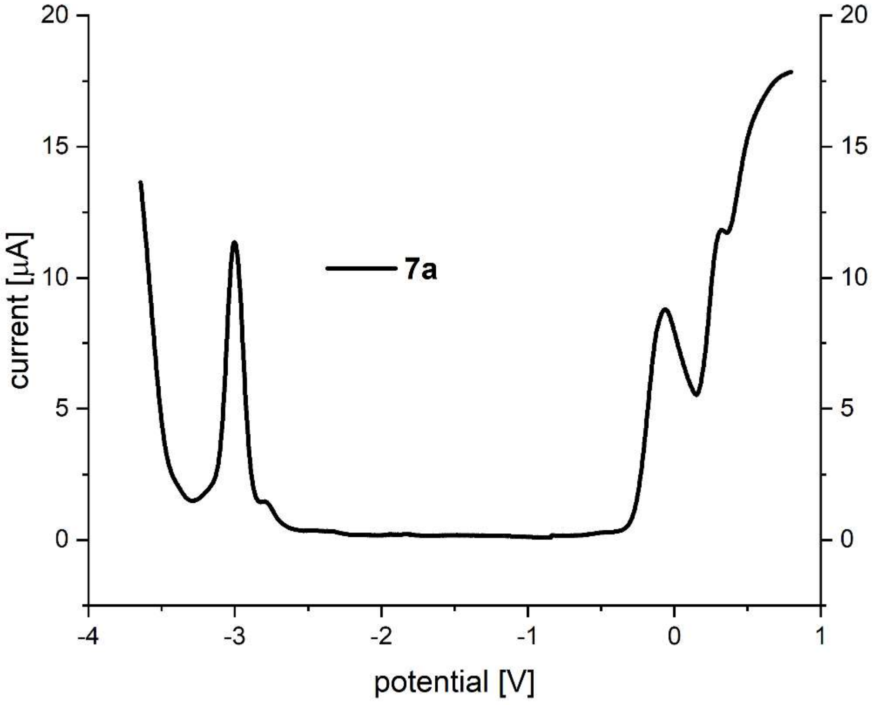

Figure S121. Square wave voltammogram of **7a** in THF (vs. $[Cp_2Fe]^{0/+}$).

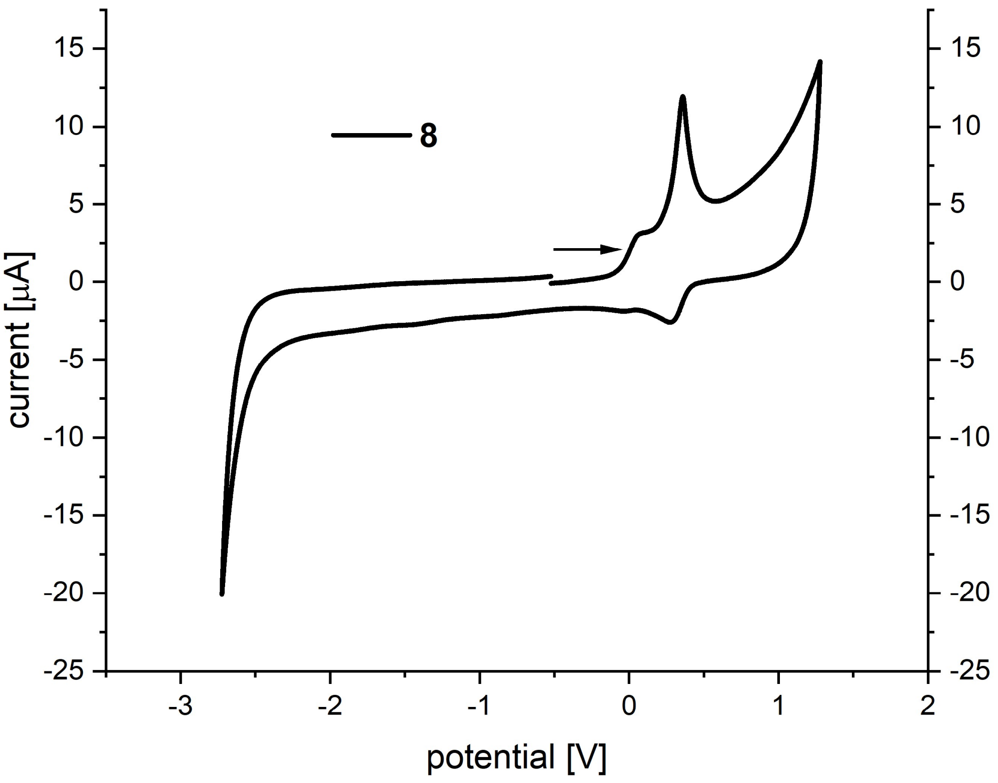

Figure S122. Cyclic voltammogram of **8** in DCM (vs. $[Cp_2Fe]^{0/+}$, scan rate: 250 mVs^{-1}).

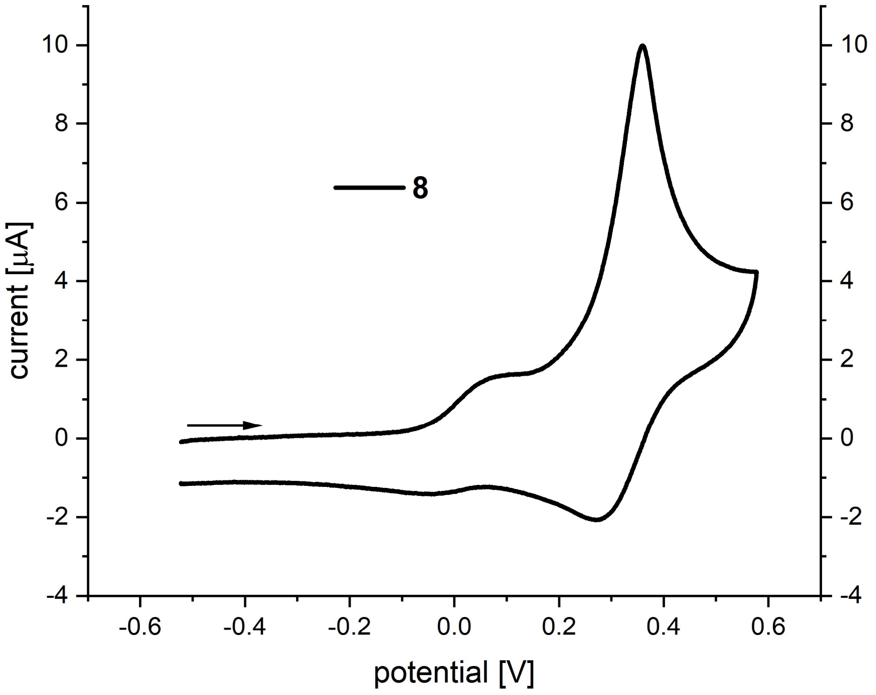

Figure S123. Cyclic voltammogram of the oxidation events of **8** in DCM (vs. $[Cp_2Fe]^{0/+}$, scan rate: 250 mVs^{-1}).

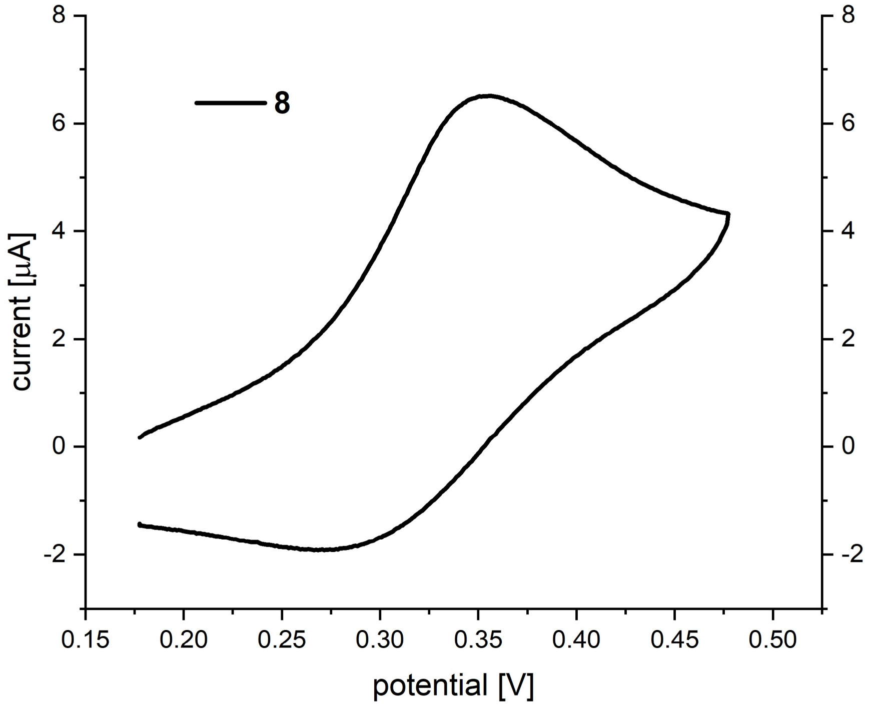

Figure S124. Cyclic voltammogram of the second oxidation event of **8** in DCM (vs. $[Cp_2Fe]^{0/+}$, scan rate: 250 mVs^{-1}).

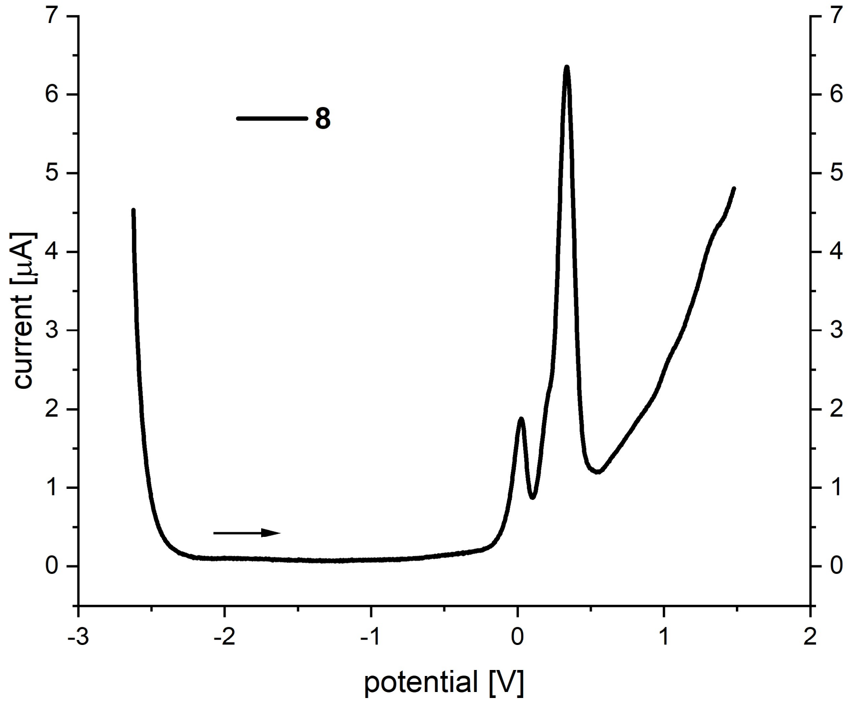

Figure S125. Square wave voltammogram of **8** in DCM (vs. $[Cp_2Fe]^{0/+}$).

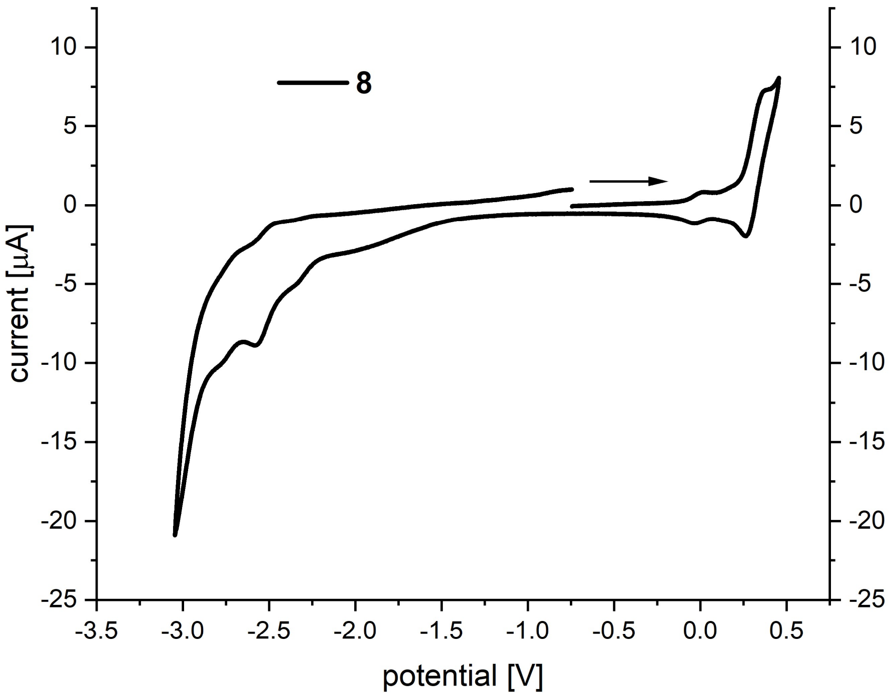

Figure S126. Cyclic voltammogram of **8** in THF (vs. $[Cp_2Fe]^{0/+}$, scan rate: 250 mVs^{-1}).

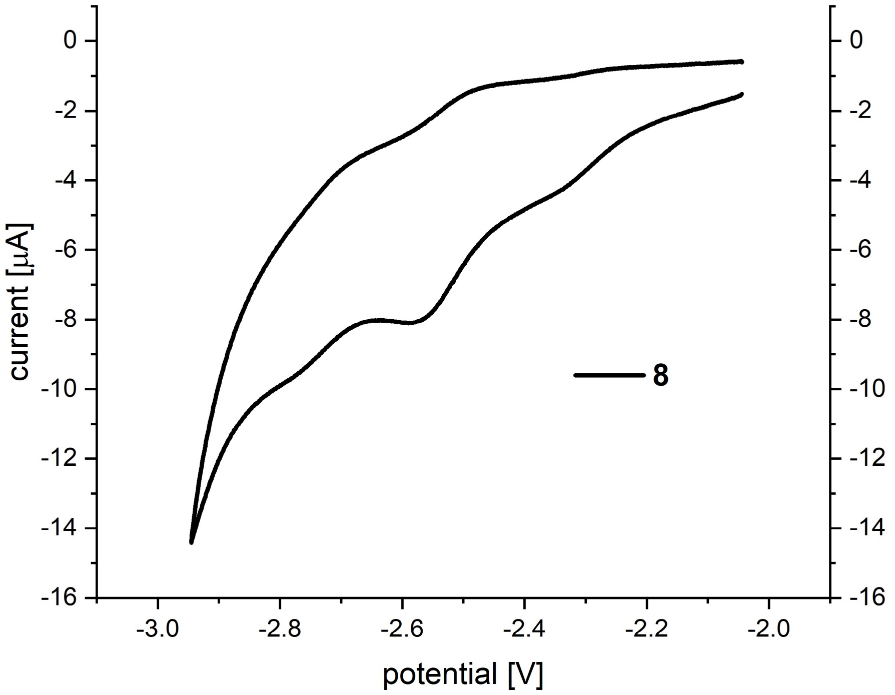

Figure S127. Cyclic voltammogram of the reduction events of **8** in THF (vs. $[Cp_2Fe]^{0/+}$, scan rate: 250 mVs^{-1}).

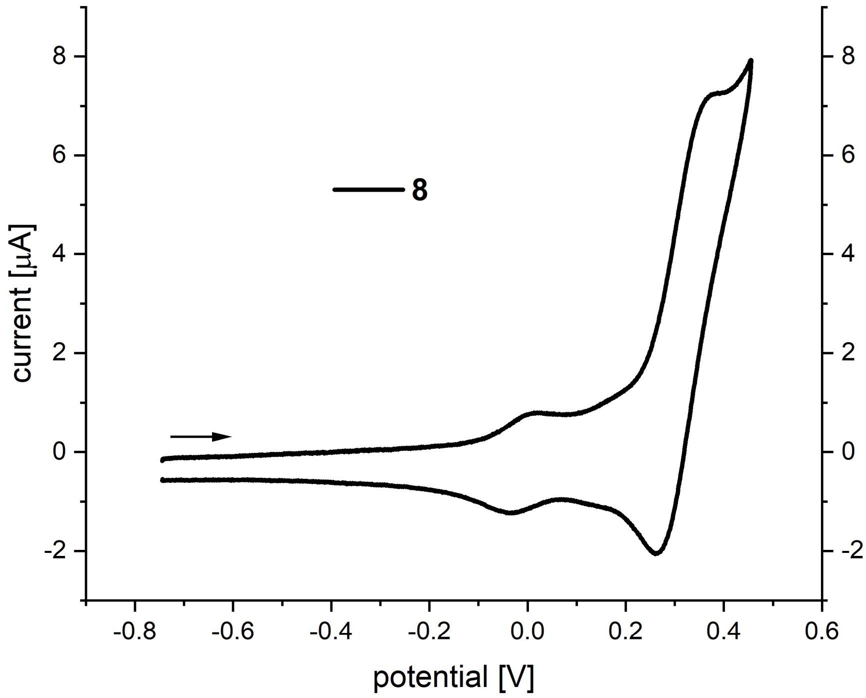

Figure S128. Cyclic voltammogram of the oxidation events of **8** in THF (vs. $[Cp_2Fe]^{0/+}$, scan rate: 250 mVs^{-1}).

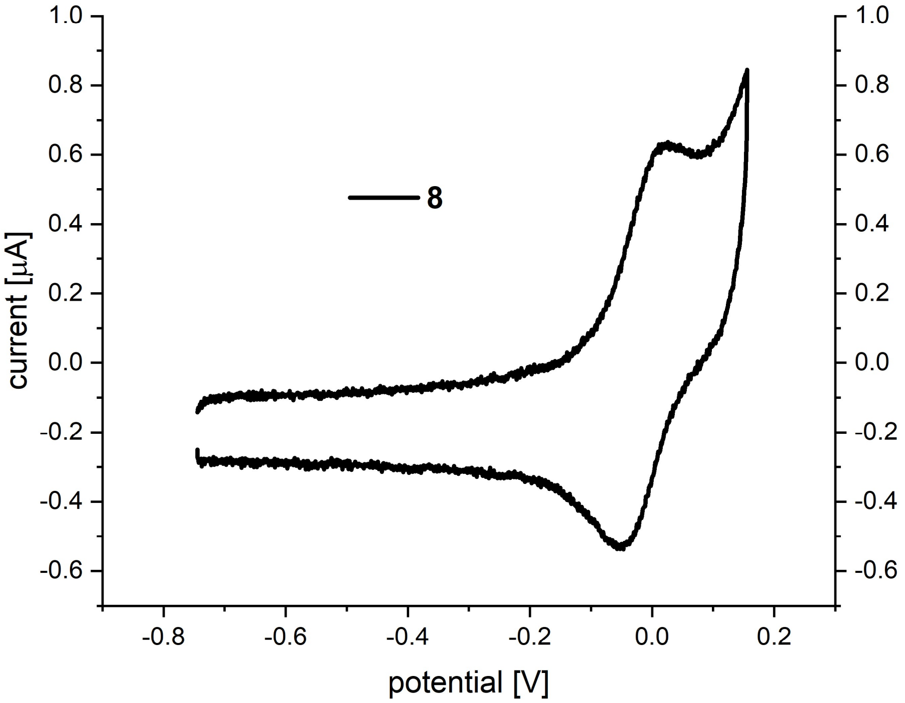

Figure S129. Cyclic voltammogram of the first oxidation event of **8** in THF (vs. $[Cp_2Fe]^{0/+}$, scan rate: 250 mVs^{-1}).

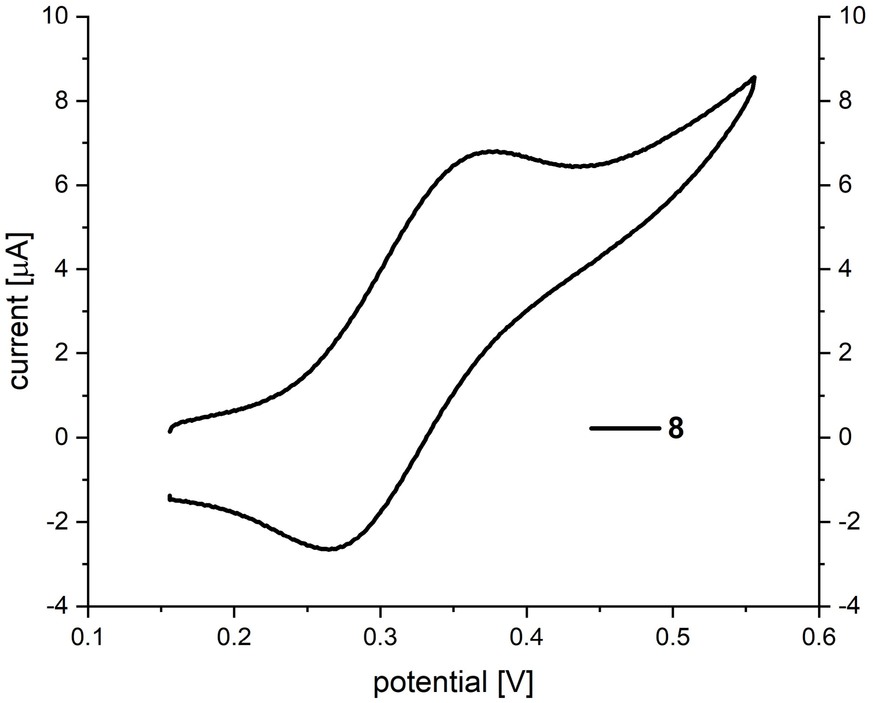

Figure S130. Cyclic voltammogram of the second oxidation event of **8** in THF (vs. $[Cp_2Fe]^{0/+}$, scan rate: 250 mVs^{-1}).

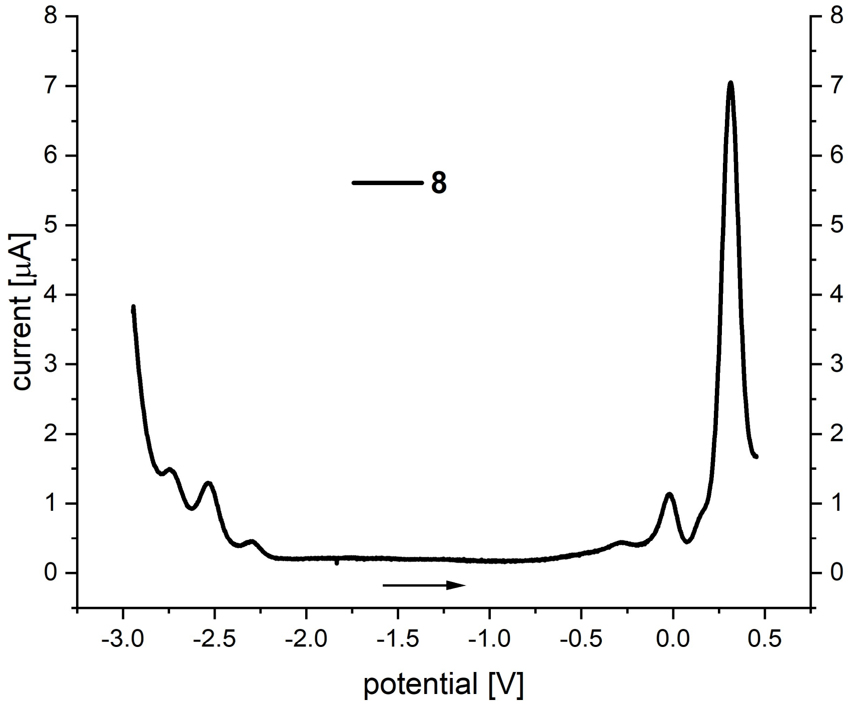

Figure S131. Square wave voltammogram of **8** in THF (vs. $[Cp_2Fe]^{0/+}$).

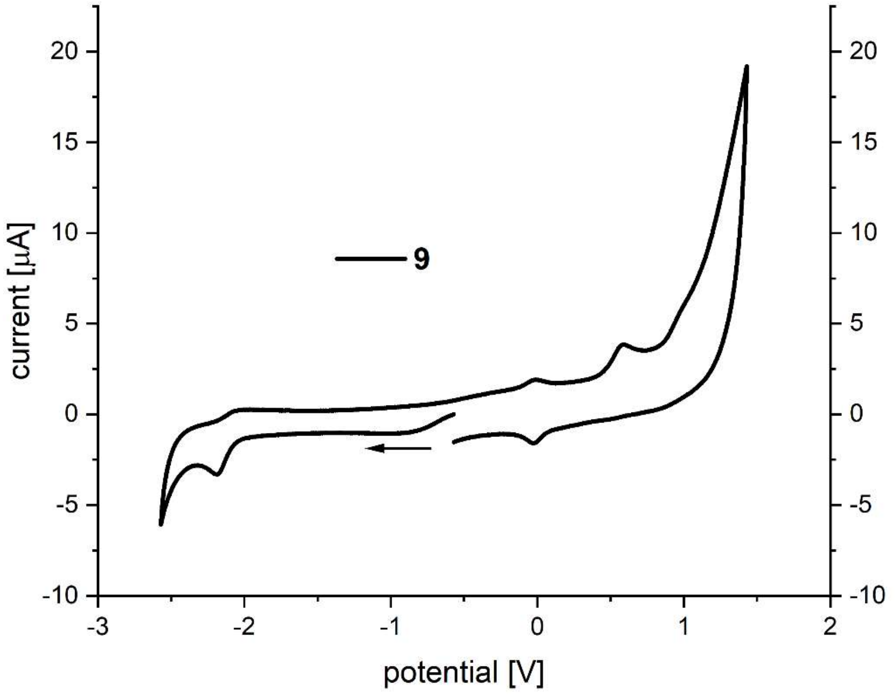

Figure S132. Cyclic voltammogram of **9** in DCM (vs. $[Cp_2Fe]^{0/+}$, scan rate: 250 mVs^{-1}).

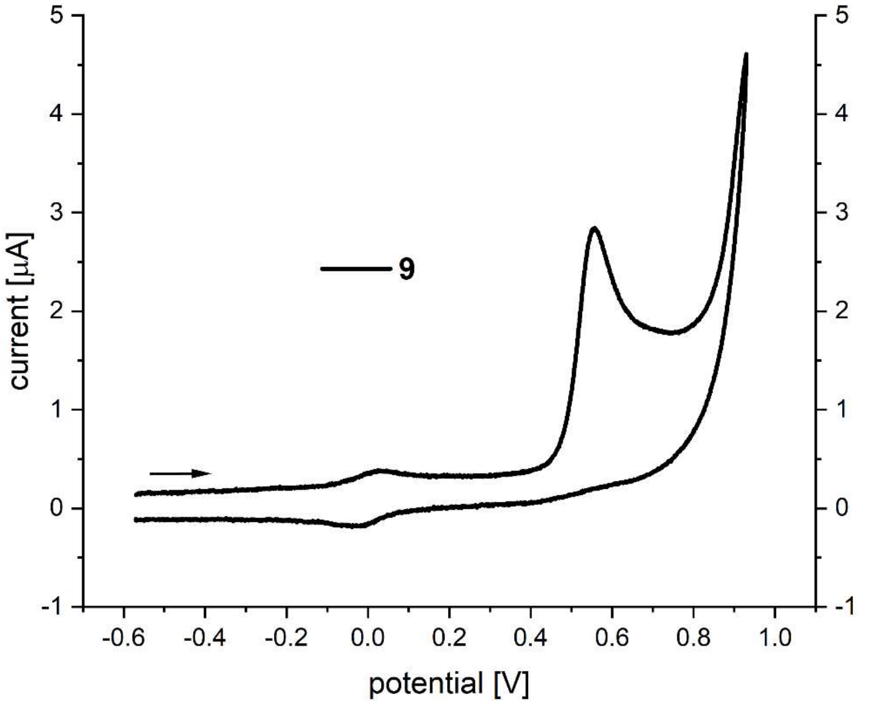

Figure S133. Cyclic voltammogram of the oxidation event of **9** in DCM (vs. $[Cp_2Fe]^{0/+}$, scan rate: 250 mVs^{-1}).

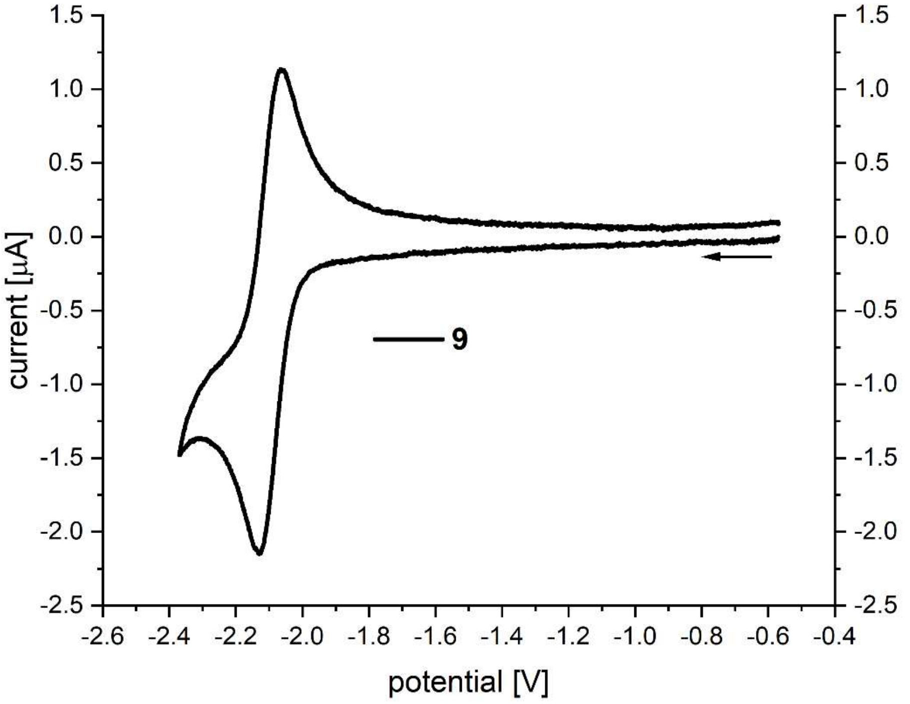

Figure S134. Cyclic voltammogram of the reduction event of **9** in DCM (vs. $[Cp_2Fe]^{0/+}$, scan rate: 250 mVs^{-1}).

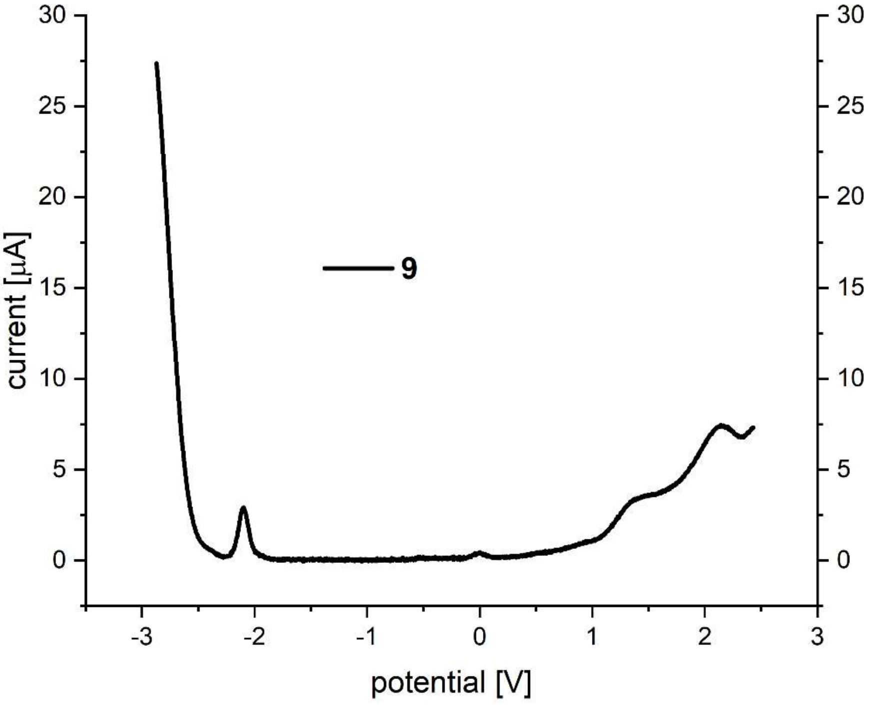

Figure S135. Square wave voltammogram of **9** in DCM (vs. $[Cp_2Fe]^{0/+}$).

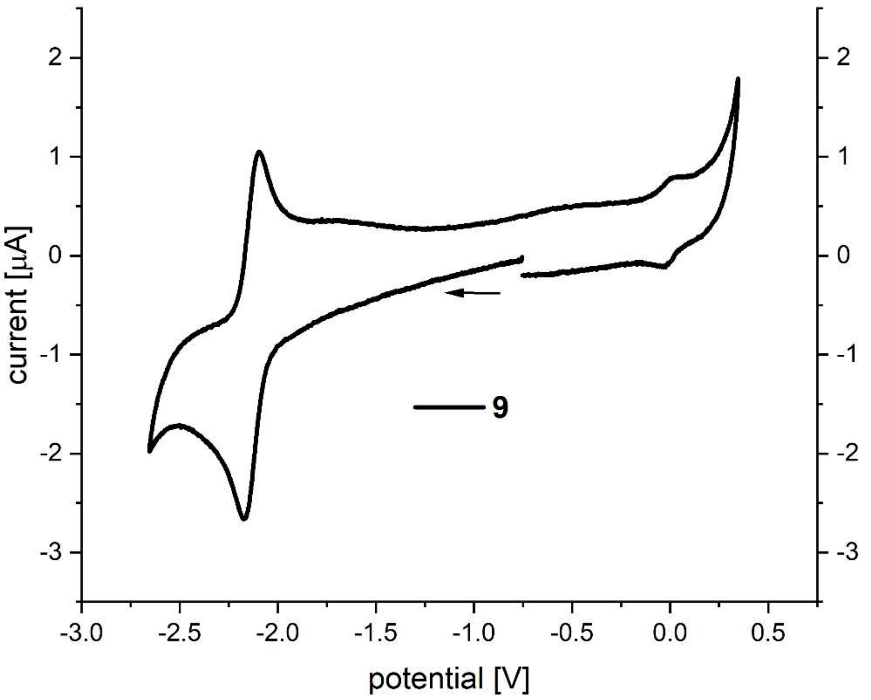

Figure S136. Cyclic voltammogram of **9** in THF (vs. $[Cp_2Fe]^{0/+}$, scan rate: 250 mVs^{-1}).

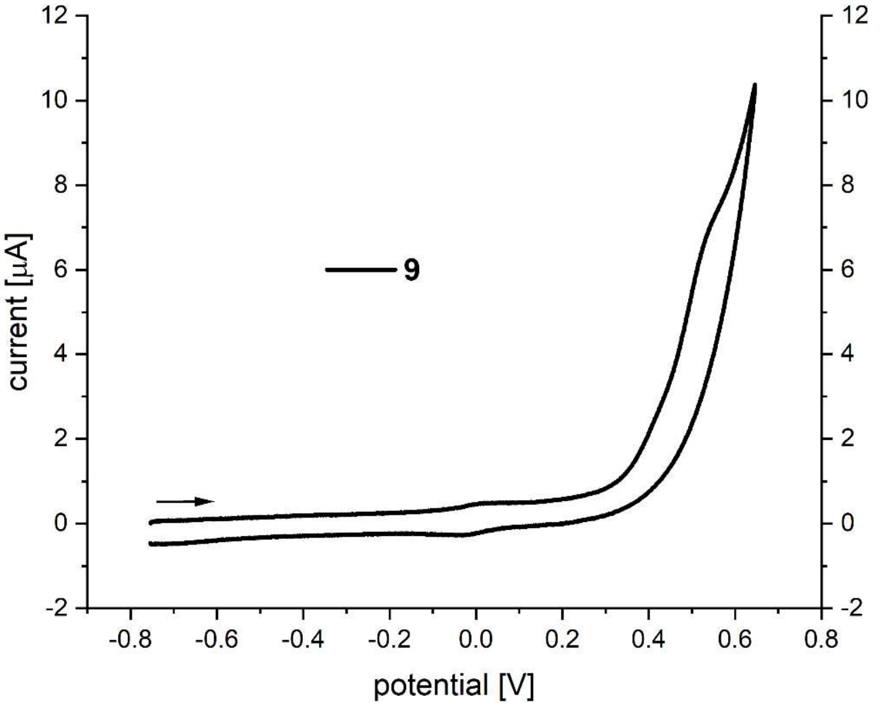

Figure S137. Cyclic voltammogram of the oxidation event of **9** in THF (vs. $[Cp_2Fe]^{0/+}$, scan rate: 250 mVs^{-1}).

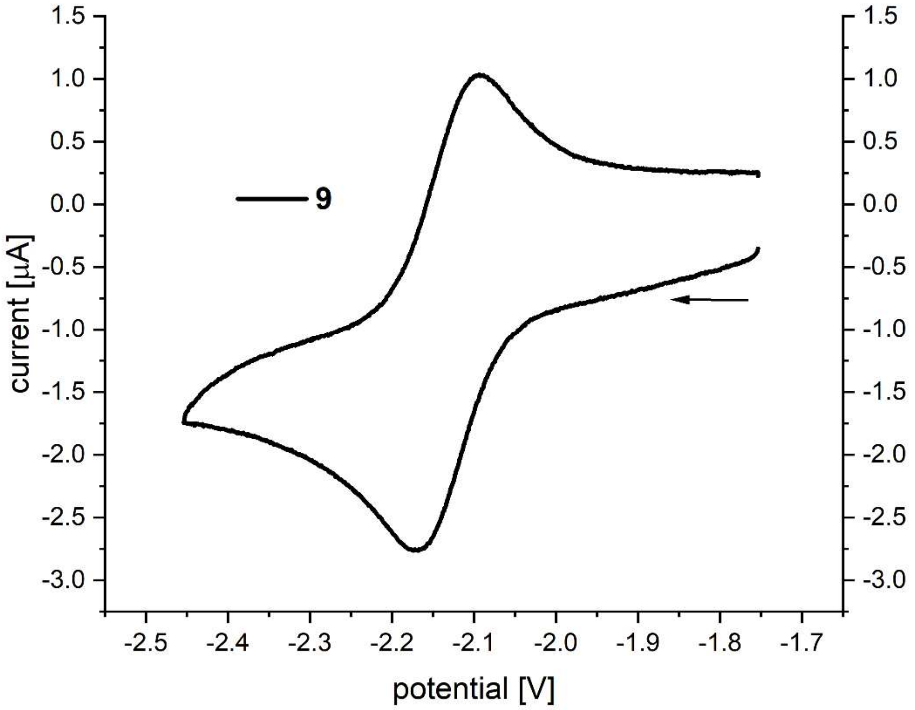

Figure S138. Cyclic voltammogram of the reduction event of **9** in THF (vs. $[Cp_2Fe]^{0/+}$, scan rate: 250 mVs^{-1}).

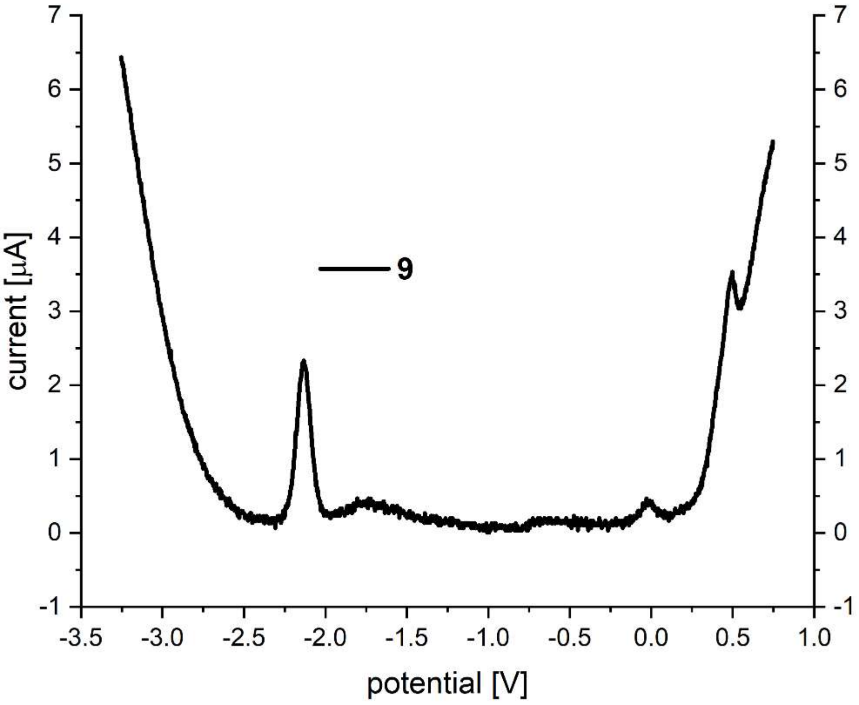

Figure S139. Square wave voltammogram of **9** in THF (vs. $[Cp_2Fe]^{0/+}$).

1.9 Gel Permeation Chromatography

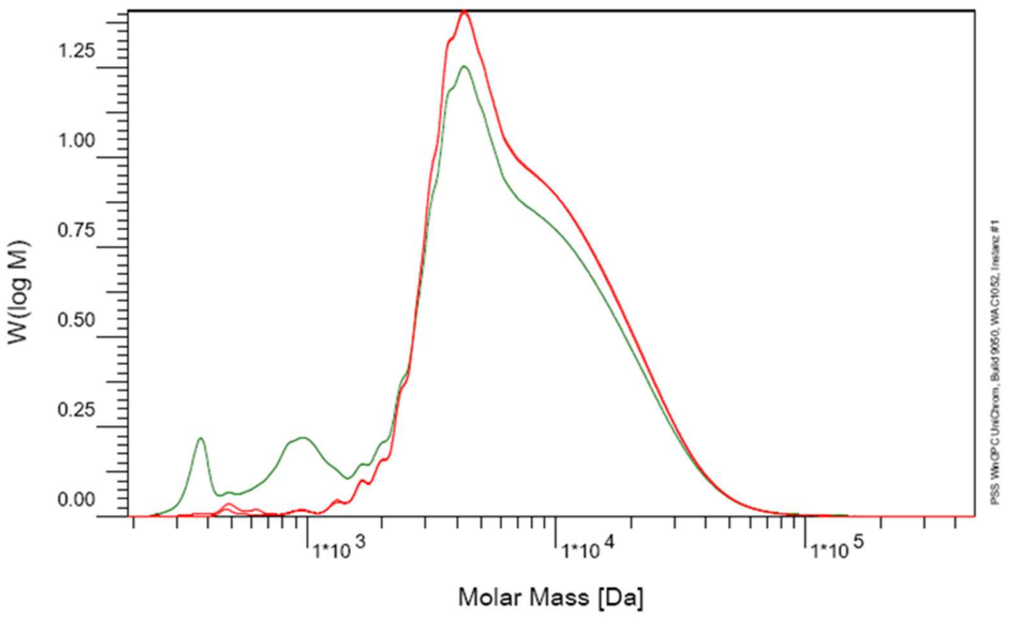

	I1: RID 1, RI Signal	Unsicherheit [%]	I1: VWD 1, Signal A	Unsicherheit [%]	I1: VWD 1, Signal B	Unsicherheit [%]	
Mn :	3.2564e3	3.95	5.2849e3	0.17	5.5078e3	0.16	g/mol
Mw :	9.0759e3	3.95	9.7311e3	0.08	9.8454e3	0.07	g/mol
Mz :	2.1300e4	3.95	1.9470e4	0.15	2.0111e4	0.14	g/mol
Mv :	0.000000	3.95	0.000000	0.17	0.000000	0.16	g/mol
D :	2.7871e0	5.58	1.8413e0	0.18	1.7875e0	0.17	
[n]:	0.000000	0.00	0.000000	0.00	0.000000	0.00	ml/g
Vp :	2.5104e1	3.92	2.5103e1	0.03	2.5103e1	0.02	ml
Mp :	4.2826e3	3.95	4.2832e3	0.19	4.2832e3	0.19	g/mol
FI :	1.5773e1	3.92	1.1159e3	0.03	7.7997e2	0.02	ml*V
< 190	0.00	3.92	0.00	0.03	0.00	0.02	
w% :	100.00	3.92	100.00	0.03	100.00	0.02	
> 478386	0.00	3.92	0.00	0.03	0.00	0.02	

Figure S140. Molecular mass distributions of **8** by GPC (in THF, vs. polystyrene standards, detected by RI and VWD detectors).

2. Computational Information

Computational methods. Geometry optimizations and TD-DFT calculations were carried out using the TURBOMOLE V7.7 program package.[6] Optimizations of the excited state structure were carried out using the Gaussian 09 program package, revision D.01.[7] Becke's three parameter exchange-correlation hybrid functional B3LYP[8] was used in combination with the 6-31+G* basis set. The empirical dispersion correction DFT-D3 by Grimme was used including the three-body term and with Becke-Johnson (BJ) damping.[9] The stationary points were characterized as minima by analytical vibrational frequency calculations,[10] which revealed the absence of imaginary frequencies. Vertical singlet excitations were calculated by means of time-dependent DFT[11] using the same density functional and the valence-double-ζ basis set def2-SV(P).[12]

Computational results:

Table S5. Results from TD-DFT calculations for the compounds **2**, **4**, **7a-c** and **9**. π – π* transitions are highlighted in **bold**.

Compound	λ / nm	Oscillator strength *f*	Orbital contributions	|c|^2 / %
**2**	**319.6**<br>**265.6**	**0.3592**<br>**0.1288**	**HOMO → LUMO**<br>**HOMO-4 → LUMO**<br>**HOMO-2 → LUMO**	**97.5**<br>**52.4**<br>**34.2**
**4**	**311.1**<br>**251.5**	**0.4981**<br>**0.1348**	**HOMO → LUMO**<br>**HOMO-5 → LUMO**	**98.5**<br>**72.7**
**7a**	**389.0**	**0.7467**	**HOMO → LUMO**	**98.9**
**7b**	**411.2**	**1.0266**	**HOMO → LUMO**	**99.4**
**7c**	**390.3**	**0.9820**	**HOMO → LUMO**	**99.1**
**9**	345.2<br>282.7	0.0625<br>0.1638	HOMO → LUMO+1<br>HOMO-3 → LUMO+1<br>HOMO-5 → LUMO+1	91.4<br>47.7<br>36.1

Transition orbitals and orbital energies in eV

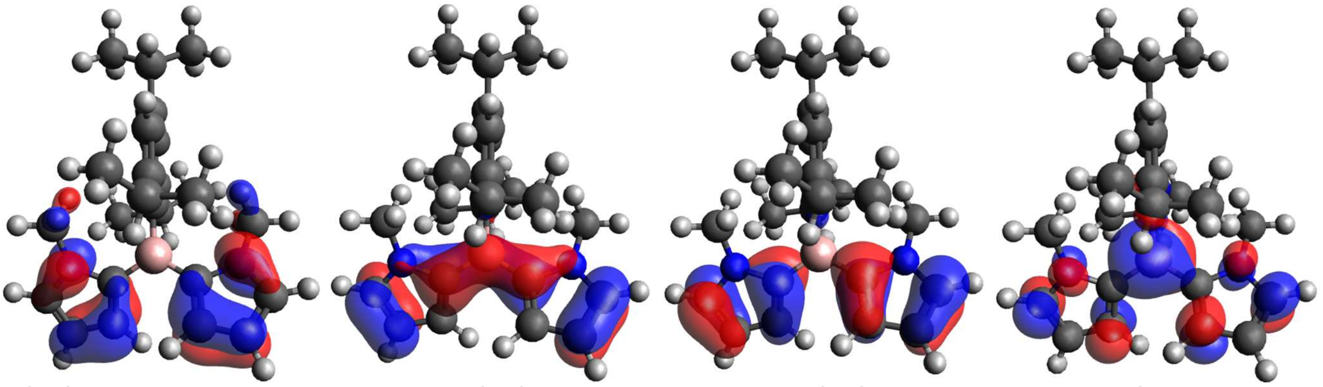

HOMO-4 -6.353eV   HOMO-2 -6.186 eV   HOMO -5.437 eV   LUMO -1.133 eV

Figure S141. Transition orbitals and orbital energies for compound **2**.

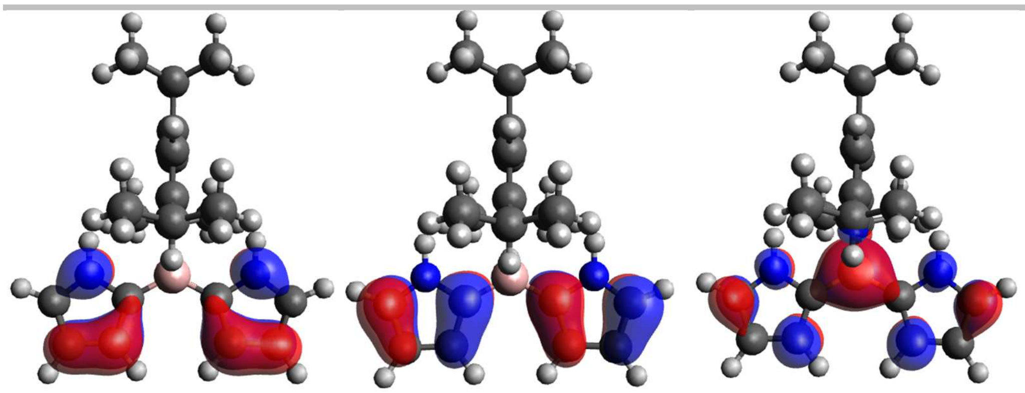

HOMO-5 -6.353eV | HOMO -5.503 eV | LUMO -1.219 eV

Figure S142. Transition orbitals and orbital energies for compound **4**.

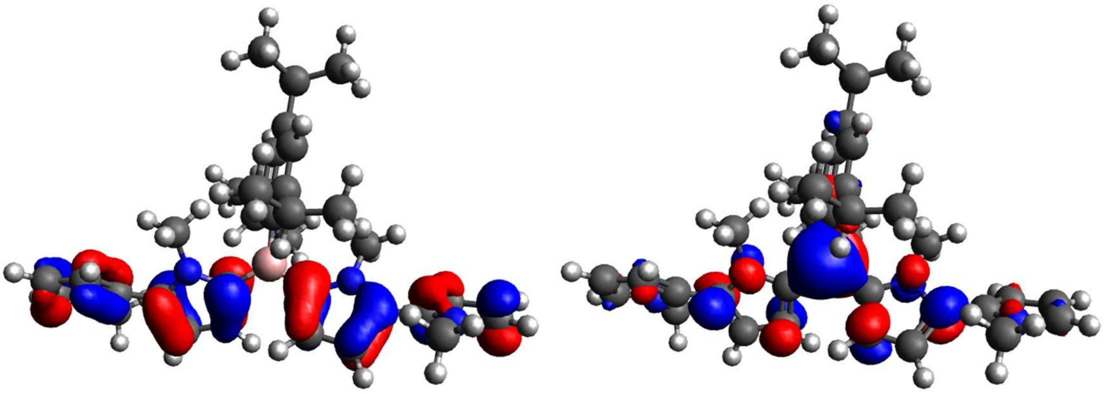

HOMO -5.015 eV | LUMO -1.423 eV

Figure S143. Transition orbitals and orbital energies for compound **7a**.

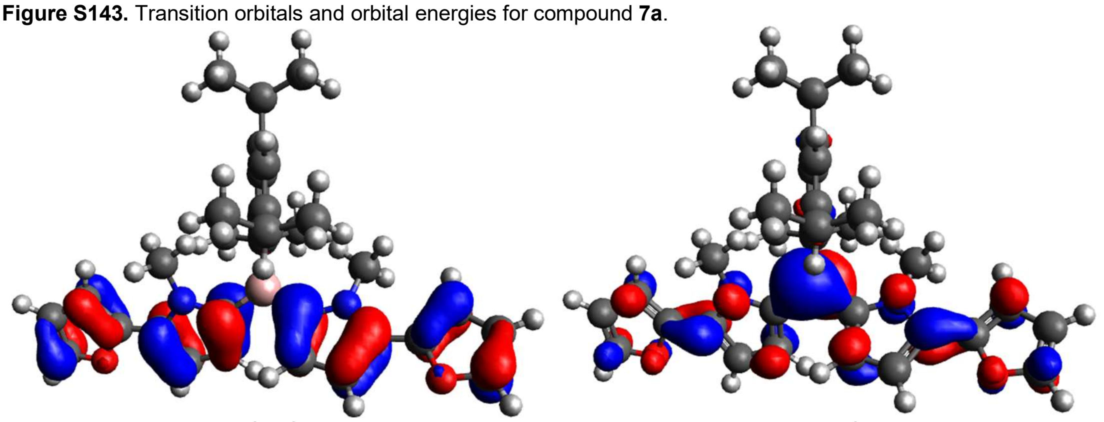

HOMO -5.078 eV | LUMO -1.718 eV

Figure S144. Transition orbitals and orbital energies for compound **7b**.

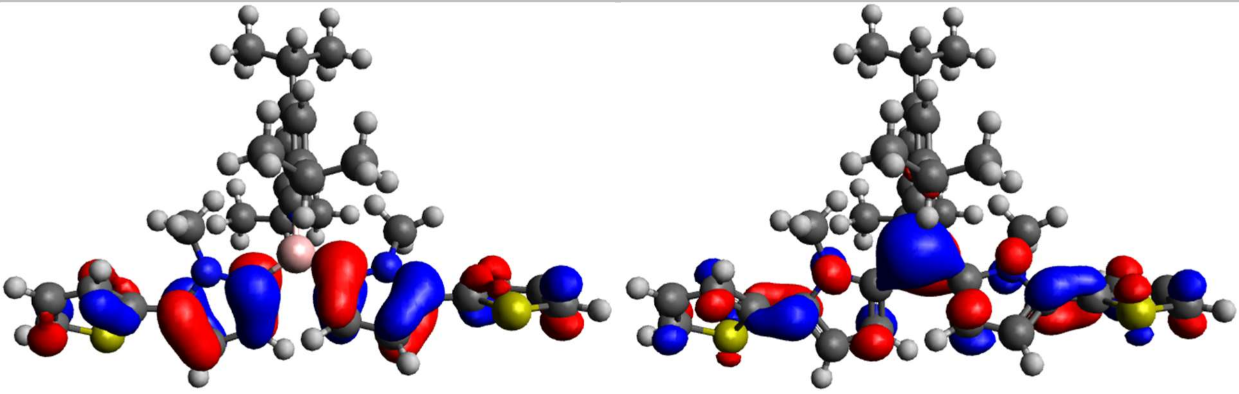

HOMO
-5.295 eV

LUMO
-1.703 eV

Figure S145. Transition orbitals and orbital energies for compound **7c**.

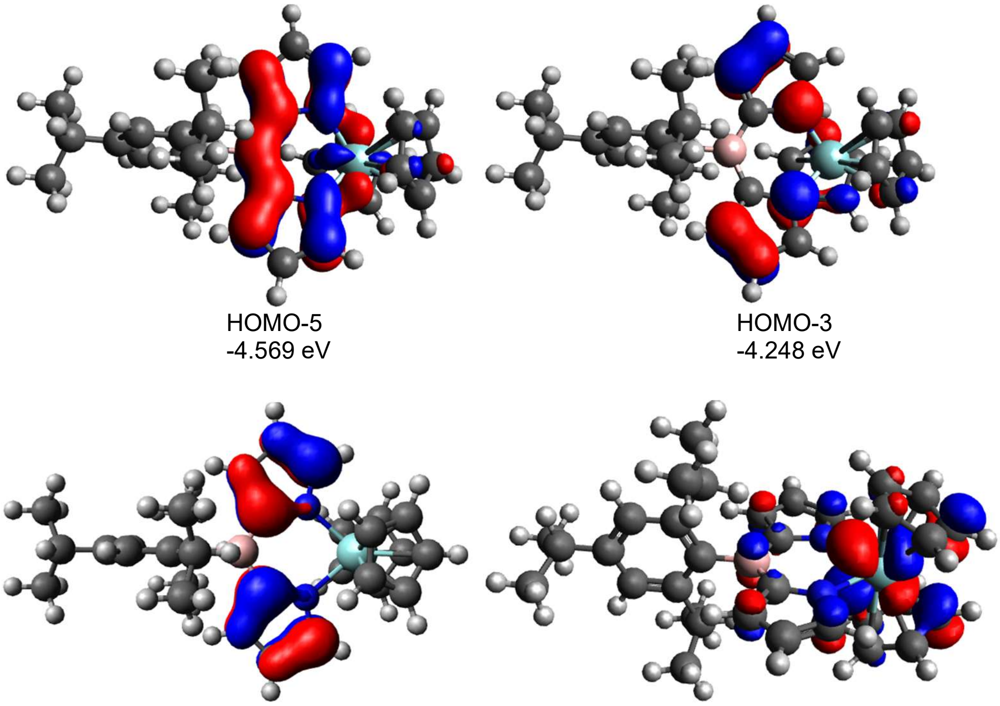

HOMO-5
-4.569 eV

HOMO-3
-4.248 eV

HOMO
-3.573 eV

LUMO+1
+0.547 eV

Figure S146. Transition orbitals and orbital energies for compound **9**.

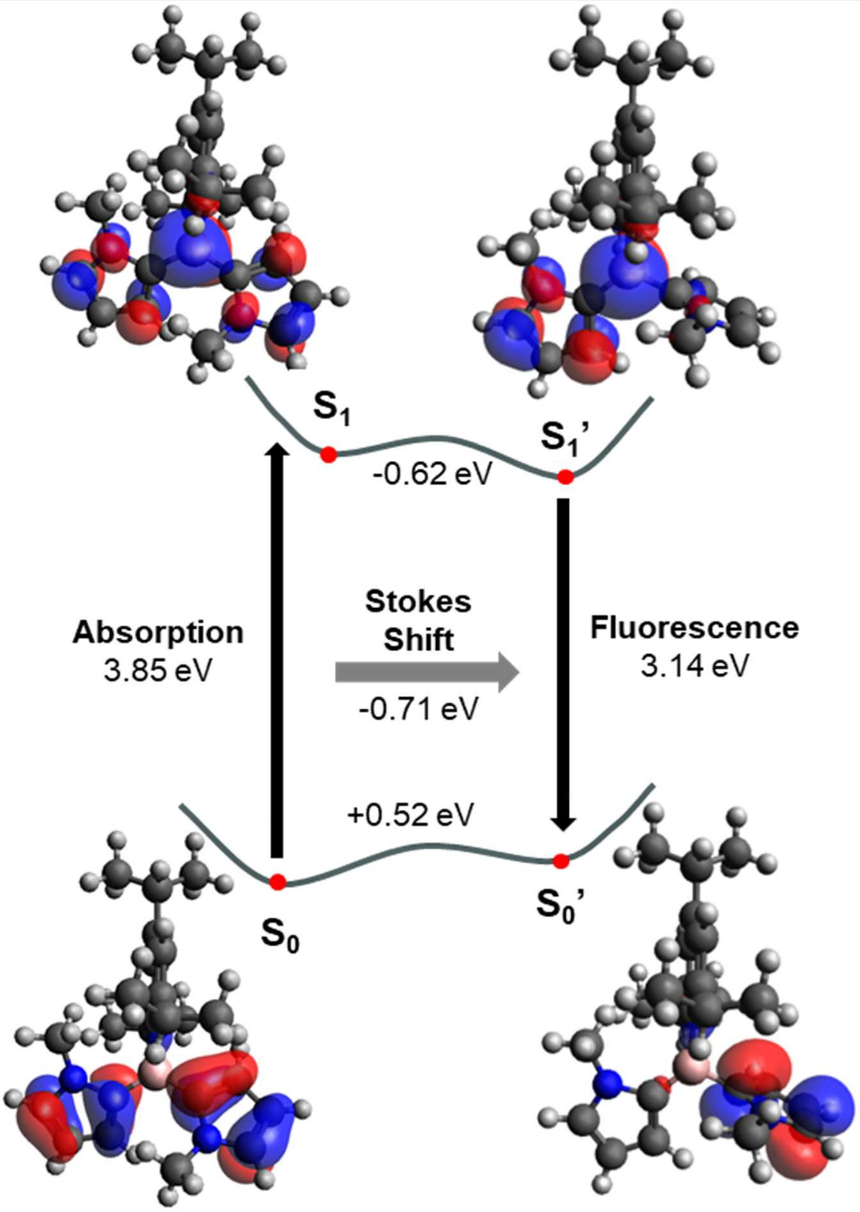

Figure S147. Graphical representation for the TICT emission of **2** with optimized S_0 and S_1' structures at the B3-LYP/6-31+G* level of theory and their orbital contributions at an isovalue of 0.035.

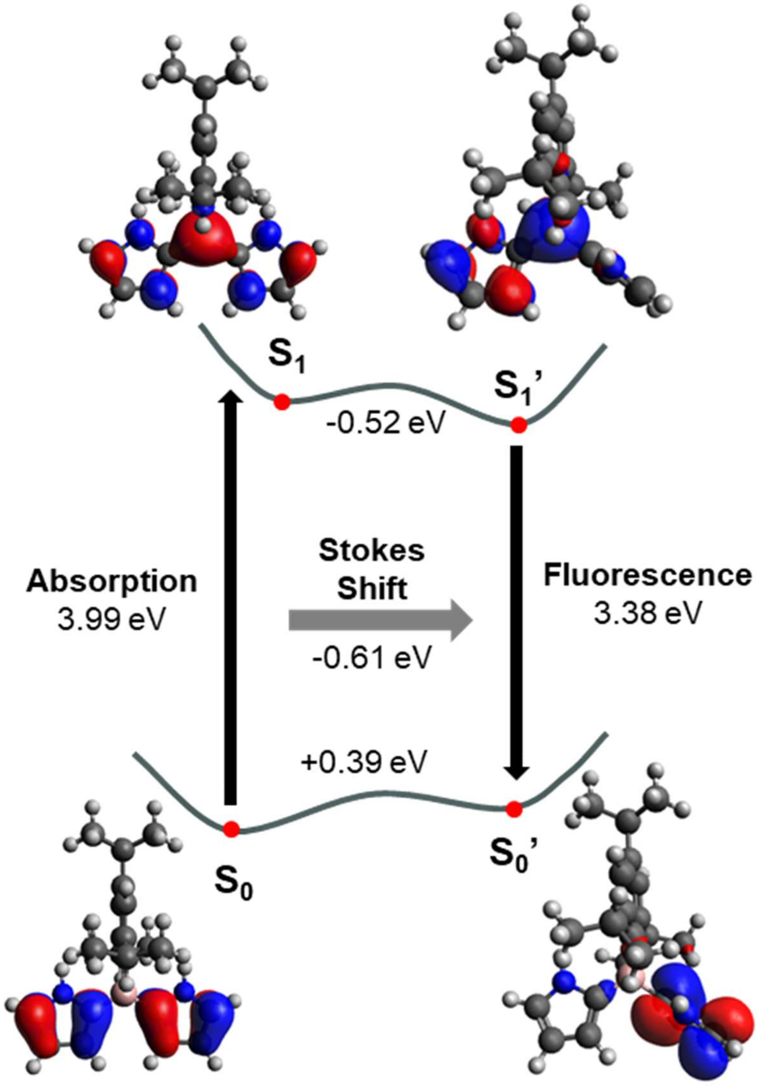

Figure S148. Graphical representation for the TICT emission of **4** with optimized S_0 and S_1' structures at the B3-LYP/6-31+G* level of theory and their orbital contributions at an isovalue of 0.035.

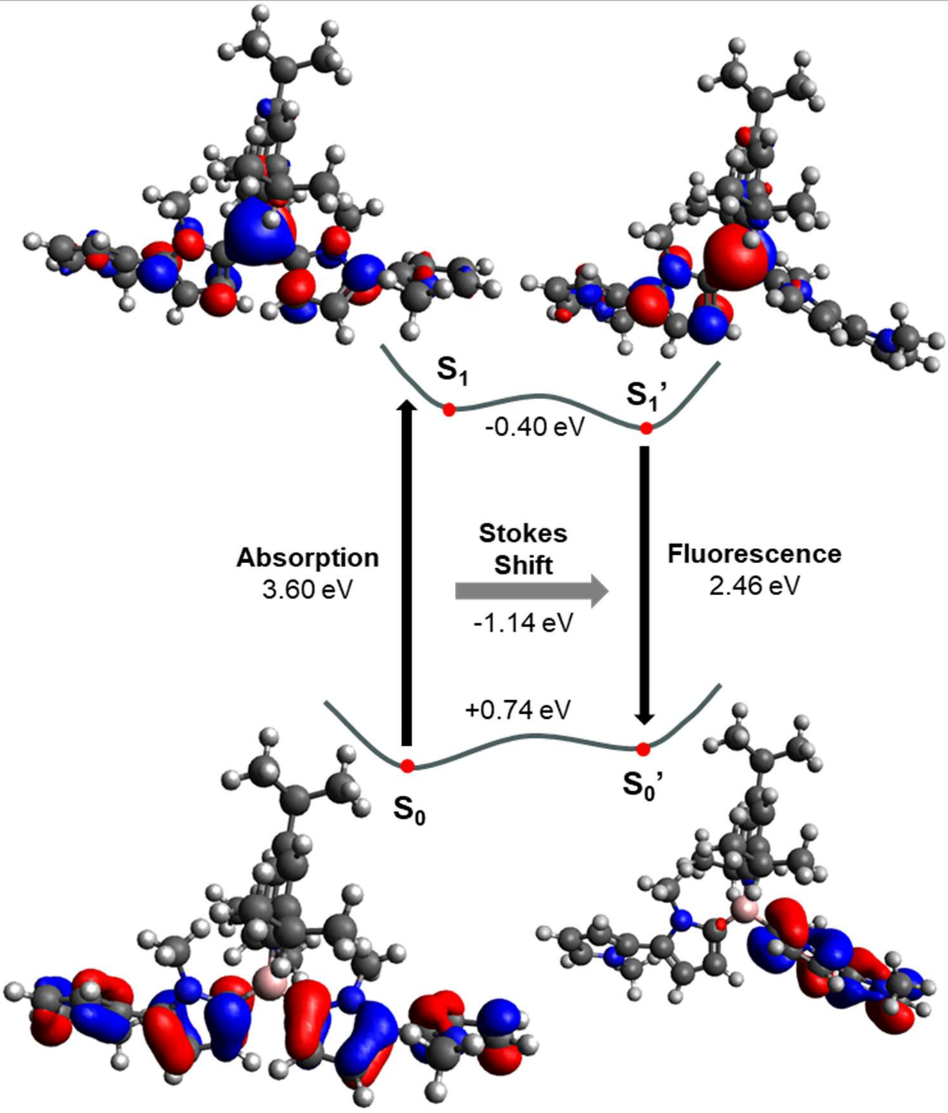

Figure S149. Graphical representation for the TICT emission of **7a** with optimized S_0 and S_1' structures at the B3-LYP/6-31+G* level of theory and their orbital contributions at an isovalue of 0.035.

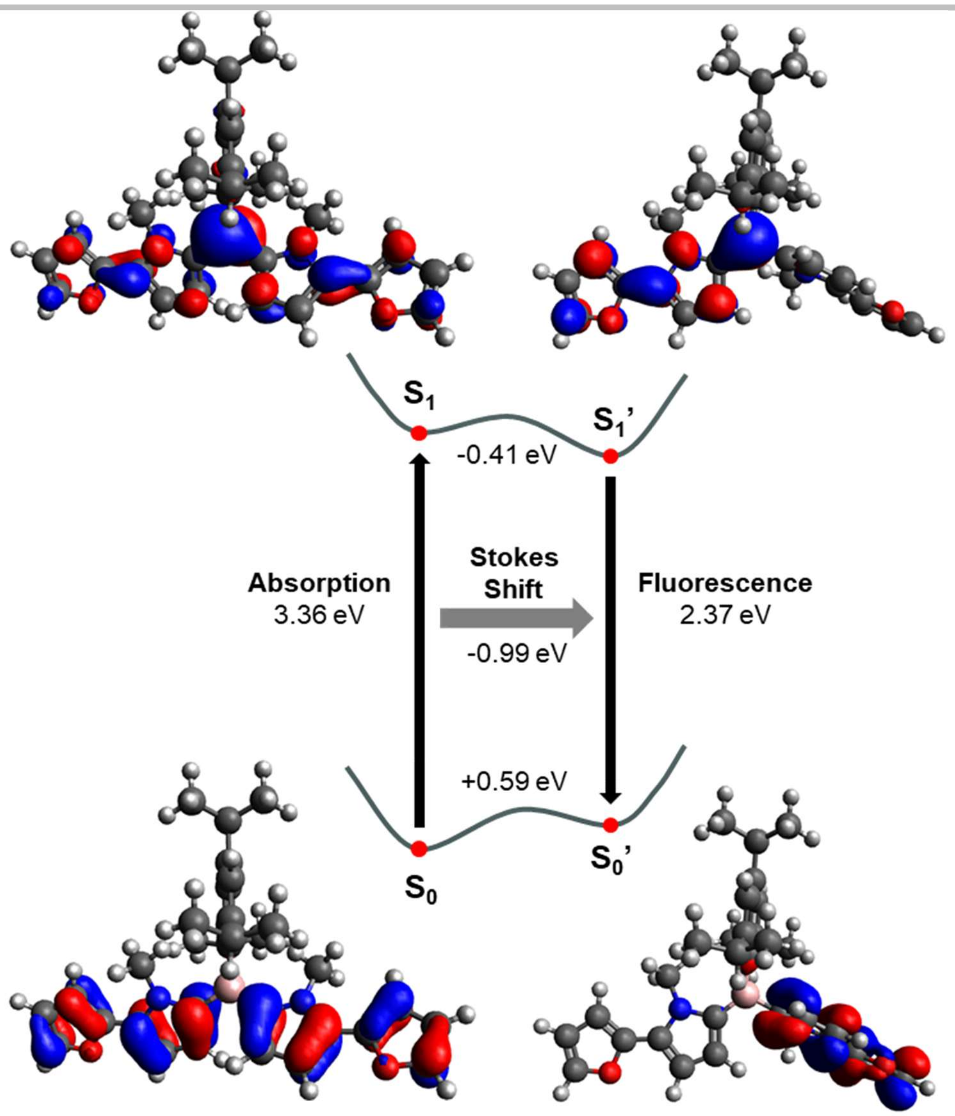

Figure S150. Graphical representation for the TICT emission of **7b** with optimized S_0 and S_1' structures at the B3-LYP/6-31+G* level of theory and their orbital contributions at an isovalue of 0.035.

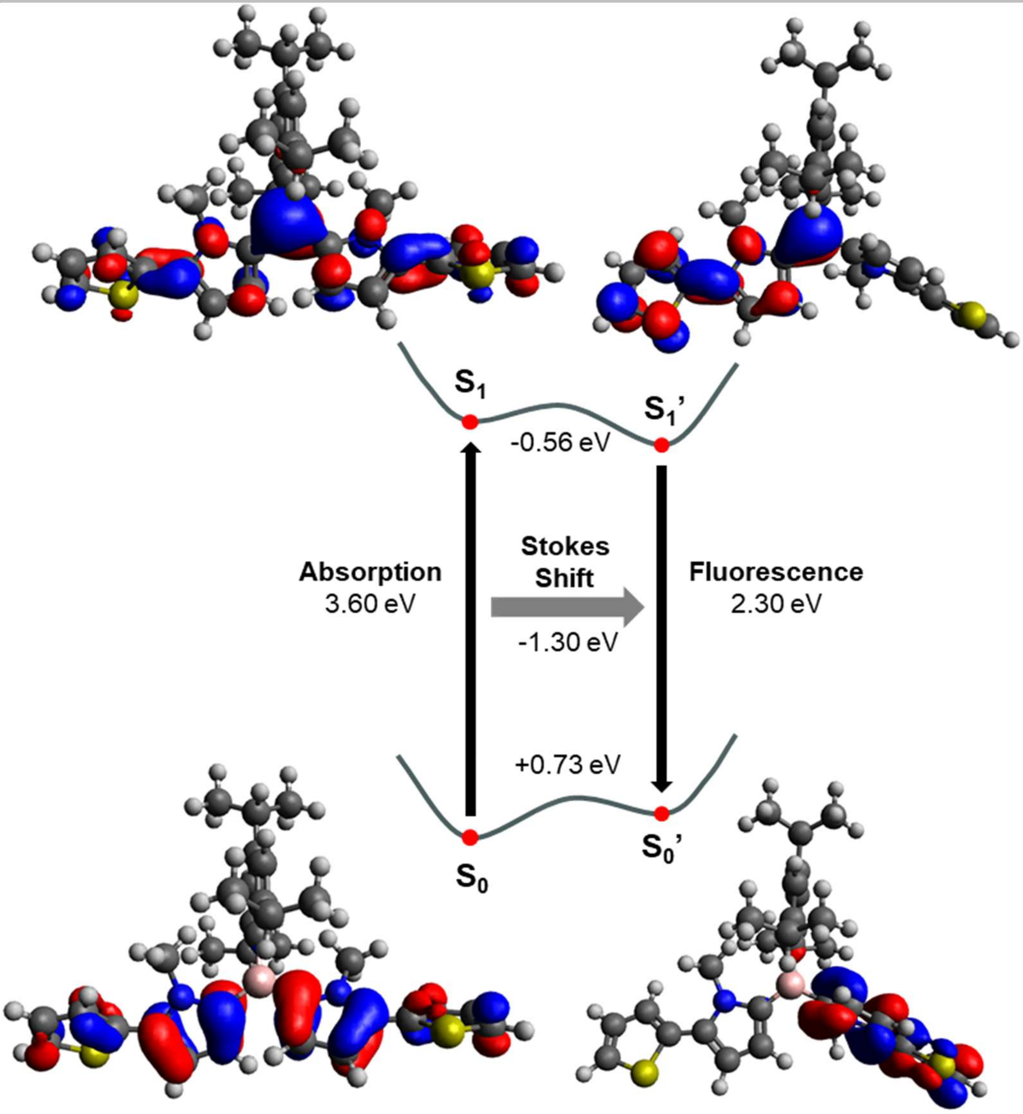

Figure S151. Graphical representation for the TICT emission of **7c** with optimized S_0 and S_1' structures at the B3-LYP/6-31+G* level of theory and their orbital contributions at an isovalue of 0.035.

Cartesian coordinates (Å) and total energies (a.u.) of optimized stationary point

Total energy of compound **2** (B3LYP-D3(BJ)/def2-SV(P)) = -1106.647990

C	1.096783	0.943190	-0.177686
C	0.139054	-0.067745	0.056429
C	0.339351	-1.349877	-0.506062
C	1.474830	-1.596057	-1.286163
C	2.435309	-0.603126	-1.521604
C	2.227678	0.659241	-0.955941
B	-1.143498	0.230224	0.938665
C	-2.522457	0.441655	0.284871
N	-2.770627	0.786002	-1.049354
C	-4.111473	0.738107	-1.298621
C	-4.775434	0.341500	-0.143875
C	-3.783293	0.165609	0.844720
C	-1.789676	1.208339	-2.033717
C	-0.683901	-2.460980	-0.275537
C	-1.654098	-2.586413	-1.463366
C	3.666431	-0.885511	-2.367971
C	3.290615	-1.239768	-3.816553
C	0.911252	2.336672	0.420704
C	1.232135	3.470380	-0.566410
C	-1.044728	0.315007	2.473866
C	-1.856782	1.069761	3.339870
C	-1.327147	1.000142	4.646414
C	-0.200665	0.189571	4.569538
N	-0.023528	-0.208195	3.276187
C	1.039995	-1.102947	2.854288
C	-0.043042	-3.820825	0.043513
C	4.551920	-1.975354	-1.740942
C	1.723033	2.503907	1.717209
H	0.483890	-0.141884	5.350806
H	-1.709823	1.480789	5.546725
H	-2.725108	1.643388	3.018326
H	1.609214	-1.414535	3.744115
H	0.626267	-1.999775	2.367131
H	1.721929	-0.610458	2.143766
H	-3.936004	-0.172206	1.868940
H	-5.850898	0.197090	-0.041265
H	-4.502885	1.001258	-2.281681
H	-2.320902	1.514673	-2.948722
H	-1.205358	2.064065	-1.660920
H	-1.088593	0.395289	-2.278085
H	1.619972	-2.589098	-1.720908
H	2.972782	1.440270	-1.136607
H	-1.288256	-2.172560	0.603552
H	-0.817594	-4.548071	0.346510
H	0.477724	-4.245316	-0.833432
H	0.693492	-3.741909	0.863081
H	-2.390985	-3.390274	-1.282689
H	-2.212242	-1.650082	-1.623619
H	-1.103743	-2.828018	-2.391873
H	4.259776	0.046758	-2.398270
H	5.472089	-2.124753	-2.335510
H	4.846402	-1.703865	-0.711704
H	4.021295	-2.944066	-1.694281
H	4.196125	-1.379500	-4.435418
H	2.705870	-2.176778	-3.859413
H	2.679512	-0.441771	-4.274443
H	-0.154984	2.436326	0.694415
H	0.941703	4.445898	-0.136528
H	2.311799	3.521852	-0.794962
H	0.694419	3.343757	-1.522923
H	1.576810	3.513728	2.143284
H	1.412810	1.770906	2.479165
H	2.802693	2.367883	1.520539

Total energy of the exited-state structure of compound **2** (B3LYP/6-31+G*) = -1108.249626

B	1.31118	0.07959	-0.05751
C	2.03515	-0.83756	1.04356
C	2.04188	-0.67684	2.48945
C	2.71057	-1.73097	3.06756
C	3.16234	-2.53734	2.0054
N	2.74195	-1.99563	0.81725
C	3.15492	-2.51683	-0.48727
H	3.77122	-3.42893	2.02488
H	2.88264	-1.92434	4.1155
H	1.56771	0.14852	2.99402
H	2.56618	-3.39392	-0.75576
H	3.0094	-1.73214	-1.22397
H	4.21177	-2.78244	-0.44987
C	2.15789	1.18531	-0.61472

C	-0.29619	-0.08701	-0.0761
N	1.77808	2.15182	-1.57336
C	2.8513	2.96829	-1.87647
C	3.93004	2.5911	-1.09974
C	3.52247	1.49225	-0.32253
H	4.13439	0.97548	0.4039
H	4.89744	3.07429	-1.09148
H	2.75562	3.7515	-2.6136
C	0.6032	2.07902	-2.41549
H	0.47771	3.03	-2.93689
H	0.69685	1.28284	-3.16539
H	-0.29193	1.88529	-1.82766
C	-1.10585	0.79741	0.69781
C	-2.49889	0.68836	0.67256
C	-3.15852	-0.29053	-0.07083
C	-2.36875	-1.16698	-0.81009
C	-0.97251	-1.08309	-0.82728
C	-0.50264	1.89905	1.57202
H	-3.08871	1.38856	1.25819
C	-4.67581	-0.40633	-0.08247
H	-2.86351	-1.93494	-1.40037
C	-0.21271	-2.07609	-1.70043
C	-0.91715	1.76231	3.04913
H	0.58374	1.79952	1.51294
C	-0.8391	3.30917	1.05364
H	-0.37325	2.48129	3.67098
H	-1.98456	1.95471	3.19308
H	-0.71984	0.75854	3.43693
H	-0.36921	4.0728	1.68197
H	-0.4768	3.45422	0.03429
H	-1.91804	3.49485	1.05835
H	-4.92608	-1.24771	-0.74064
C	-5.24187	-0.73258	1.3108
C	-5.3471	0.8478	-0.66936
H	-6.32596	-0.87904	1.26668
H	-4.79427	-1.64339	1.71757
H	-5.04766	0.07653	2.0214
H	-6.43221	0.71611	-0.73128
H	-5.15717	1.73031	-0.05105
H	-4.97518	1.06328	-1.67453
H	0.83815	-1.77702	-1.65232
C	-0.33315	-3.51735	-1.17504
C	-0.62526	-2.00262	-3.18069
H	-0.00349	-2.66722	-3.79011
H	-1.66674	-2.30154	-3.33152
H	-0.513	-0.98822	-3.57147
H	0.26938	-4.20914	-1.77477
H	-0.00884	-3.59507	-0.13295
H	-1.36732	-3.87267	-1.21515

Total energy of compound **4** (B3LYP-D3(BJ)/def2-SV(P)) = -1028.017640

N	-0.142579	-0.613129	3.019173
C	-0.480226	0.588927	2.398433
C	0.110398	0.514841	1.124421
C	0.787358	-0.718349	1.001272
C	0.608154	-1.397797	2.204471
B	-1.331823	1.637375	3.139474
C	-1.752510	2.993620	2.542388
N	-2.528844	3.881993	3.285241
C	-2.791719	5.015142	2.585230
C	-2.182062	4.899458	1.337832
C	-1.537240	3.643286	1.313936
C	-1.810479	1.292304	4.629477
C	-1.010361	1.642643	5.745833
C	-1.445415	1.325151	7.042867
C	-2.657454	0.665830	7.280335
C	-3.442066	0.325148	6.169009
C	-3.042258	0.624856	4.858319
C	0.325440	2.366380	5.556895
C	-3.943569	0.224937	3.686568
C	-3.098773	0.331637	8.701696
H	1.346285	-1.083161	0.139026
H	0.964461	-2.377287	2.524608
H	-0.433706	-0.846594	3.962843
H	-0.963314	3.237592	0.481700
H	-2.207128	5.645673	0.543137
H	-3.387571	5.823370	3.010125
H	-0.822102	1.598675	7.901779
H	-4.395227	-0.190845	6.328216
C	1.516398	1.531951	6.064381
H	0.474146	2.506289	4.471093
C	0.317187	3.771093	6.188824
C	-5.306619	0.941392	3.731411
H	-3.442631	0.552687	2.757675
C	-4.117728	-1.301861	3.581657
C	-3.200867	-1.188242	8.932381
H	-2.312134	0.712510	9.379401
C	-4.411388	1.041491	9.083843
H	-2.844882	3.681513	4.228423

H	0.051188	1.287783	0.358940
H	1.267635	4.297849	5.983873
H	-0.504182	4.385933	5.779149
H	0.189901	3.723501	7.286187
H	2.471825	2.048026	5.855425
H	1.459980	1.360349	7.155255
H	1.550204	0.545607	5.568039
H	-4.718126	-1.565695	2.691331
H	-3.140020	-1.808265	3.491984
H	-4.632816	-1.715759	4.468197
H	-5.911055	0.687247	2.841080
H	-5.890128	0.654220	4.625906
H	-5.177461	2.038305	3.749853
H	-4.673206	0.838448	10.138990
H	-4.325877	2.135705	8.957502
H	-5.255079	0.696046	8.458142
H	-3.455166	-1.408692	9.985839
H	-3.984933	-1.641674	8.297809
H	-2.246422	-1.693198	8.698380

Total energy of the exited-state structure of compound **4** (B3LYP/6-31+G*) = -1029.602878

N	-1.27203	2.0555	-1.61827
C	-1.95	1.18921	-0.74666
C	-3.30211	1.63712	-0.80517
C	-3.39009	2.73796	-1.68436
C	-2.12332	2.98133	-2.18216
B	-1.27272	0.05622	-0.03389
C	-2.27829	-0.95582	0.70257
N	-2.6678	-0.81848	2.01056
C	-3.60528	-1.7411	2.36631
C	-3.87438	-2.54684	1.23723
C	-3.06566	-2.08422	0.22508
C	0.32489	-0.15434	-0.01573
C	1.16073	0.70132	0.77601
C	2.55688	0.58264	0.71557
C	3.20792	-0.3498	-0.08459
C	2.39956	-1.18786	-0.84197
C	0.99998	-1.11655	-0.82868
H	-4.2791	3.30107	-1.93312
H	-1.76669	3.72444	-2.87868
H	-0.29165	1.97029	-1.82688
H	-3.02615	-2.44969	-0.78678
H	-4.59014	-3.35401	1.20582
H	-4.02101	-1.77448	3.3619
H	3.15396	1.25264	1.32942
H	2.87673	-1.92773	-1.4817
H	-2.29031	-0.10284	2.61315
H	-4.12849	1.20701	-0.25647
C	4.72437	-0.46051	-0.13368
H	4.96167	-1.26333	-0.84285
C	0.71186	1.8256	1.73495
H	1.5732	1.96945	2.39698
C	0.3282	-2.1624	-1.73363
H	1.12622	-2.51973	-2.3946
C	-0.76173	-1.64476	-2.68415
H	-0.3662	-0.85411	-3.32733
H	-1.10554	-2.45684	-3.33475
H	-1.62278	-1.21974	-2.17009
C	-0.10328	-3.40303	-0.9279
H	-0.81471	-3.1637	-0.13689
H	-0.56293	-4.15587	-1.57824
H	0.7622	-3.8649	-0.44444
C	-0.4612	1.53505	2.68187
H	-1.41452	1.60973	2.1545
H	-0.35481	0.54946	3.14875
H	-0.47479	2.27697	3.48774
C	0.49629	3.17871	1.03068
H	1.35278	3.43358	0.39981
H	-0.39625	3.17227	0.40567
H	0.38372	3.9774	1.77241
C	5.31872	-0.86292	1.22757
H	6.40219	-1.00238	1.15446
H	5.13582	-0.09586	1.98628
H	4.88163	-1.79701	1.59057
C	5.38234	0.82597	-0.66244
H	6.46608	0.70054	-0.75431
H	4.98934	1.09703	-1.64594
H	5.20449	1.67167	0.00875

Total energy of compound **4** with *N*-H groups in anti-position to each other (B3LYP-D3(BJ)/def2-SV(P)) = -1028.141750

C	-3.015398	0.611488	4.845254
C	-1.793365	1.285629	4.626035
C	-1.007102	1.659864	5.735843
C	-1.441522	1.347984	7.031897
C	-2.646125	0.674357	7.262120
C	-3.422754	0.315987	6.151992
B	-1.329046	1.642779	3.152933
C	-1.695658	3.035640	2.612396

N	-2.570319	3.849873	3.322594
C	-2.746528	5.047215	2.705173
C	-1.963805	5.053061	1.552870
C	-1.309832	3.800025	1.497638
C	0.306860	2.404231	5.528021
C	0.241980	3.831177	6.095490
C	-3.094645	0.339976	8.676579
C	-4.421720	1.030279	9.033401
C	-3.887105	0.200113	3.663336
C	-3.973414	-1.328336	3.532006
C	-0.563716	0.593177	2.345147
N	-0.275867	0.662635	0.986365
C	0.414199	-0.435618	0.576740
C	0.611853	-1.259621	1.682423
C	-0.006881	-0.619996	2.781747
C	1.506834	1.622233	6.083477
C	-5.279265	0.848885	3.719221
C	-3.180641	-1.179399	8.899667
H	1.140785	-2.212688	1.679891
H	0.710230	-0.566244	-0.464296
H	-0.614106	1.397919	0.378057
H	-0.583137	3.496411	0.742989
H	-1.874262	5.878002	0.845981
H	-3.406030	5.810727	3.117124
H	-0.827650	1.634855	7.892535
H	-4.369444	-0.210917	6.309953
H	0.462848	2.497534	4.438445
H	-3.396289	0.572689	2.746035
H	-2.322014	0.732340	9.363096
H	-3.001022	3.555458	4.193461
H	-0.050633	-0.975814	3.811459
H	1.177426	4.381910	5.886028
H	-0.594318	4.393711	5.643870
H	0.093145	3.821008	7.191033
H	2.452977	2.151816	5.867193
H	1.435799	1.494978	7.179135
H	1.563704	0.617805	5.628130
H	-4.568011	-1.613848	2.644461
H	-2.965420	-1.766588	3.427590
H	-4.451664	-1.780368	4.420255
H	-5.872009	0.585804	2.823890
H	-5.847041	0.517262	4.607796
H	-5.198815	1.950129	3.762358
H	-4.700436	0.827125	10.083911
H	-4.348600	2.124422	8.901311
H	-5.245858	0.668529	8.391555
H	-3.446015	-1.409044	9.948164
H	-3.950778	-1.635265	8.250549
H	-2.217462	-1.669152	8.671465

Total energy of compound **4** with *N*-H groups in syn-position to each other facing away from the Tip-group (B3LYP-D3(BJ)/def2-SV(P)) = -1028.136390

C	-3.077566	0.666965	4.899842
C	-1.838821	1.305477	4.671131
C	-1.001682	1.592844	5.768967
C	-1.409381	1.242939	7.063717
C	-2.633198	0.609450	7.303701
C	-3.455684	0.329073	6.204412
B	-1.395418	1.691913	3.205169
C	-1.960230	2.969155	2.564389
C	-2.576253	4.071669	3.170668
C	-2.923934	5.010889	2.168856
C	-2.535567	4.462918	0.951198
N	-1.943010	3.259501	1.199190
C	0.340321	2.283993	5.555209
C	0.429990	3.619331	6.310632
C	-3.053875	0.235518	8.716770
C	-4.340591	0.963466	9.140535
C	-4.002846	0.340412	3.732404
C	-4.291575	-1.165192	3.627056
C	-0.409386	0.780424	2.458072
N	0.333487	1.149204	1.335349
C	1.127508	0.120771	0.919306
C	0.888731	-0.970073	1.748063
C	-0.058998	-0.551510	2.714225
C	1.514774	1.353587	5.897005
C	-5.296739	1.168064	3.790383
C	-3.195815	-1.286940	8.883157
H	1.354932	-1.951325	1.658750
H	1.811788	0.234715	0.078401
H	0.409150	2.104444	1.005244
H	-1.697001	2.581418	0.487219
H	-2.652273	4.840949	-0.064660
H	-3.406795	5.978436	2.306223
H	-0.759305	1.468556	7.916175
H	-4.417626	-0.166961	6.369766
H	0.420721	2.520773	4.478809
H	-3.477248	0.629913	2.804289
H	-2.246364	0.568595	9.394583

H	-2.731946	4.167346	4.245530
H	-0.480065	-1.146822	3.524810
H	1.383621	4.133120	6.087782
H	-0.398001	4.290975	6.021524
H	0.377722	3.469872	7.404279
H	2.482413	1.849630	5.694680
H	1.498773	1.065571	6.964289
H	1.467181	0.429940	5.294115
H	-4.907913	-1.384661	2.735612
H	-3.352429	-1.740983	3.547217
H	-4.839747	-1.535486	4.512430
H	-5.940332	0.955289	2.916707
H	-5.877495	0.937974	4.702637
H	-5.067208	2.247997	3.796278
H	-4.597273	0.729736	10.190434
H	-4.226380	2.058009	9.047833
H	-5.196561	0.660987	8.509994
H	-3.440558	-1.547813	9.929476
H	-4.002155	-1.685389	8.240142
H	-2.260329	-1.805082	8.607095

Total energy of compound **7a** (B3LYP-D3(BJ)/def2-SV(P)) = -1604.744511

C	-2.9640262	-6.3409634	-3.1656750
N	-3.1452722	-5.4489518	-2.1369576
C	-2.8225948	-4.1713616	-2.5767645
C	-2.4252842	-4.2824178	-3.9086739
C	-2.5179722	-5.6505442	-4.2791074
C	-2.9563917	-3.0030506	-1.7142009
C	-4.0345611	-2.6308831	-0.9024626
C	-3.6965448	-1.4091932	-0.2935213
C	-2.4046492	-1.0357765	-0.7057165
N	-1.9747922	-2.0445299	-1.5765477
B	-1.5742745	0.1074769	-0.0781814
C	-2.3580490	1.3282662	0.4447868
N	-1.9472529	2.2677518	1.3971110
C	-2.8477093	3.3096813	1.4576416
C	-3.8473020	3.0638529	0.5061190
C	-3.5490006	1.8337649	-0.1057032
C	-0.7974307	2.1302971	2.2739459
C	-2.7327352	4.4183302	2.3970529
C	-2.4510016	4.4265423	3.7634340
C	-2.5142830	5.7714853	4.2145263
C	-2.8288676	6.5513794	3.1156967
N	-2.9583455	5.7357386	2.0179427
C	-0.7360343	-2.0512154	-2.3371091
C	-3.2614734	6.2095066	0.6823191
C	-3.5774368	-5.8137285	-0.8022053
C	0.0044933	0.0002541	0.0114065
C	0.5979197	-1.0309431	0.7789035
C	1.9882655	-1.1978237	0.7701470
C	2.8230563	-0.3628101	0.0211724
C	2.2310532	0.6797563	-0.7027030
C	0.8457633	0.8864436	-0.7125968
C	-0.2491827	-1.9483212	1.6582834
C	0.2411269	2.0384498	-1.5173343
C	4.3303263	-0.5610380	0.0066295
H	-4.9647245	-3.1878420	-0.7995782
H	-4.2952872	-0.8485339	0.4223356
H	-0.4023604	-3.0898315	-2.4792546
H	-0.8747307	-1.5899647	-3.3312155
H	0.0336107	-1.4916258	-1.7944683
H	-4.1063267	1.3497359	-0.9060843
H	-4.7078121	3.7040224	0.3185399
H	-0.4189159	3.1283092	2.5393527
H	-1.0647559	1.5982957	3.2058434
H	-0.0106207	1.5630953	1.7621959
H	2.8842316	1.3472888	-1.2699853
H	2.4325837	-2.0001110	1.3675586
H	-0.5640923	2.4705962	-0.9002862
C	4.7173751	-1.9305465	-0.5757552
C	-0.2079849	-3.4107890	1.1860896
H	-2.2525187	3.5439673	4.3691446
H	-2.3486215	6.1309715	5.2297827
H	-2.9625390	7.6285242	3.0220243
H	-2.1271662	-3.4513179	-4.5458316
H	-2.2845162	-6.0839988	-5.2512911
H	-3.1593818	-7.4025922	-3.0177887
H	-2.7579821	5.5756312	-0.0636140
H	-4.3473369	6.1923931	0.4756729
H	-2.8993024	7.2451837	0.5773101
H	-3.0854295	-5.1663286	-0.0596797
H	-4.6714495	-5.7125036	-0.6807327
H	-3.2961994	-6.8612905	-0.6079539
C	0.1440791	-1.8278632	3.1404900
H	-1.2966461	-1.6157552	1.5770454
H	4.7530275	0.2138861	-0.6588014
C	4.9442978	-0.3551890	1.4015798
C	1.2230710	3.1760056	-1.8254547
C	-0.4162888	1.5336044	-2.8143318

H -0.8530247 -4.0433445 1.8238606
H -0.5672006 -3.4994923 0.1471991
H 0.8175469 -3.8219498 1.2315428
H -0.5335017 -2.4303523 3.7731110
H 1.1747616 -2.1841993 3.3206383
H 0.0887974 -0.7784900 3.4813544
H 5.8162471 -2.0346293 -0.6386435
H 4.3372831 -2.7548511 0.0550678
H 4.2990633 -2.0639929 -1.5892667
H 6.0460582 -0.4401298 1.3627327
H 4.6885979 0.6407938 1.8045679
H 4.5739093 -1.1121493 2.1172783
H -0.8691010 2.3723626 -3.3738581
H 0.3305982 1.0443301 -3.4669234
H -1.2166855 0.8046178 -2.6035395
H 0.6801608 4.0307736 -2.2672809
H 1.7336544 3.5317047 -0.9130115
H 1.9970881 2.8719014 -2.5531373

Total energy of the exited-state structure of compound **7a** (B3LYP/6-31+G*) = -1604.656007

C -6.6761 -3.1033 0.69116
N -5.89558 -2.42909 -0.19848
C -4.60333 -2.30364 0.33361
C -4.63579 -2.93931 1.60374
C -5.91972 -3.44313 1.812
C -3.52469 -1.70804 -0.36834
C -3.348 -1.54446 -1.77631
C -2.11241 -0.97648 -1.97493
C -1.47923 -0.74089 -0.70857
N -2.3584 -1.21148 0.23182
B -0.01316 -0.12053 -0.39906
C 1.07338 -1.17631 -0.39743
N 2.4704 -0.97517 -0.46309
C 3.14625 -2.20251 -0.45569
C 2.17701 -3.20331 -0.34824
C 0.91728 -2.59285 -0.32616
C 3.08955 0.27416 -0.86539
C 4.59055 -2.34316 -0.57533
C 5.50052 -1.78008 -1.47178
C 6.78831 -2.30474 -1.18985
C 6.64734 -3.17901 -0.13015
N 5.32325 -3.19748 0.24939
C -2.11108 -1.12994 1.66857
C 4.81152 -3.97892 1.36112
C -6.41755 -1.85445 -1.43568
C 0.09096 1.4396 -0.03028
C -0.27094 2.46468 -0.96046
C -0.23399 3.81726 -0.60444
C 0.18668 4.24479 0.6586
C 0.58152 3.25494 1.55943
C 0.5394 1.88596 1.2551
C -0.63911 2.13157 -2.40654
C 0.98671 0.92494 2.36373
C 0.23531 5.71843 1.04058
H -4.03278 -1.88251 -2.53905
H -1.64831 -0.76525 -2.92629
H -3.00376 -0.77182 2.18636
H -1.80889 -2.10247 2.06858
H -1.29628 -0.42877 1.81736
H -0.03151 -3.10886 -0.24762
H 2.38383 -4.26607 -0.31601
H 4.11366 0.31802 -0.48803
H 3.11806 0.36904 -1.96221
H 2.52442 1.11503 -0.46828
H 0.92985 3.56613 2.54358
H -0.51874 4.56311 -1.34495
H 0.84327 -0.09463 1.99199
C -1.15945 6.37419 1.00566
C -2.09554 2.49859 -2.74971
H 5.24569 -1.09411 -2.26796
H 7.71198 -2.07475 -1.70522
H 7.3763 -3.78515 0.39048
H -3.79122 -3.05933 2.26226
H -6.27365 -3.99586 2.67078
H -7.72088 -3.28126 0.47735
H 3.96678 -3.45836 1.81744
H 4.47466 -4.97329 1.04106
H 5.60485 -4.09988 2.10499
H -6.01577 -0.85019 -1.5831
H -6.16796 -2.47747 -2.29988
H -7.50414 -1.79011 -1.35178
C 0.33232 2.77615 -3.41663
H -0.51868 1.04918 -2.51794
H 0.59404 5.76834 2.0788
C 1.23337 6.51118 0.1731
C 2.48494 1.06774 2.70455
C 0.15513 1.09105 3.65546
H -2.34994 2.17996 -3.76997
H -2.8015 2.02474 -2.05814

H	-2.25533	3.58293	-2.69363
H	0.09852	2.44912	-4.43864
H	0.27258	3.87122	-3.39709
H	1.3685	2.49381	-3.19897
H	-1.10829	7.41642	1.34701
H	-1.57183	6.37631	-0.01114
H	-1.86512	5.83711	1.65032
H	1.29942	7.55393	0.51025
H	2.236	6.07115	0.22257
H	0.92509	6.52	-0.87985
H	0.40113	0.29845	4.37477
H	0.35984	2.05132	4.14459
H	-0.92333	1.05445	3.46071
H	2.77107	0.34293	3.47822
H	3.11556	0.88767	1.83011
H	2.71395	2.07082	3.08675

Total energy of compound **7b** (B3LYP/6-31+G*) = -1565.842716

C	-6.96423200	-2.92496200	-0.03114200
C	-4.95909500	-1.99901800	0.14109700
C	-5.85196800	-1.16081100	0.77521200
C	-7.14442400	-1.76595700	0.66196300
C	-3.53878900	-2.02968000	-0.12960200
C	-2.84704400	-3.02027100	-0.83507400
C	-1.49211600	-2.67053700	-0.81065900
C	-1.33392500	-1.45777300	-0.11389700
N	-2.62571100	-1.08492900	0.28807700
B	-0.01804300	-0.64523500	-0.01126600
C	1.28215500	-1.48995900	-0.00551700
N	2.58463000	-1.09238700	-0.34569700
C	3.47944300	-2.09102900	-0.02522700
C	2.76444200	-3.14747100	0.54926100
C	1.41449300	-2.77796900	0.54673200
C	2.94326200	0.11431900	-1.07701100
C	4.90620900	-2.04305600	-0.25711300
C	5.82277700	-1.13907600	-0.75210300
C	7.10780400	-1.76540500	-0.67856900
C	6.90001300	-3.00179500	-0.14588900
C	-2.95598500	0.04059300	1.14972300
C	0.00985900	0.94949700	0.08434000
C	-0.40997100	1.75663200	-1.01006000
C	-0.34320200	3.15233300	-0.91606800
C	0.12609400	3.80065900	0.23190200
C	0.52455000	3.00120600	1.30611200
C	0.47602700	1.60087400	1.25771100
C	-0.91585100	1.14183600	-2.31999900
C	0.90391700	0.81704700	2.50324300
C	0.20956200	5.31968900	0.31859000
H	-3.30220800	-3.88166200	-1.30339900
H	-0.68351300	-3.20613600	-1.28848800
H	-3.65976500	0.71710500	0.65216800
H	-3.39956200	-0.31193200	2.08848300
H	-2.04937600	0.59777200	1.37012900
H	0.59241800	-3.35319400	0.94941400
H	3.20176700	-4.06275200	0.92264700
H	3.63158400	0.73413800	-0.49177100
H	3.41558700	-0.14417800	-2.03183700
H	2.04568600	0.69564900	-1.26898700
H	0.88345600	3.49000100	2.21046100
H	-0.66174600	3.75165400	-1.76650500
H	0.72684800	-0.24653700	2.30888000
C	-1.17370500	5.98670600	0.18773100
C	-2.40804500	1.44767200	-2.56089400
H	5.62429800	-0.14503300	-1.12162100
H	8.05640500	-1.34252600	-0.98134700
H	7.54565200	-3.83143700	0.10075400
H	-5.63301900	-0.22467400	1.26498500
H	-8.07960900	-1.38002000	1.04491300
H	-7.62427800	-3.71382400	-0.36028300
C	-0.07798700	1.57546900	-3.54071800
H	-0.82346600	0.05321900	-2.23910600
H	0.59698100	5.56208400	1.31820300
C	1.19947400	5.90155000	-0.71023800
C	2.40824700	0.97389200	2.80163700
C	0.07163500	1.19325700	3.74704300
H	-2.75793100	0.95679900	-3.47758700
H	-3.02619700	1.08766200	-1.73183900
H	-2.58282000	2.52567500	-2.66952900
H	-0.42232200	1.05251800	-4.44164800
H	-0.16342400	2.65244400	-3.73086900
H	0.98518700	1.34527000	-3.40480800
H	-1.09184200	7.07356500	0.31637200
H	-1.61484700	5.80129100	-0.79928500
H	-1.87110700	5.60537800	0.94252300
H	1.29715200	6.98692800	-0.58186300
H	2.19394800	5.45408400	-0.60041900
H	0.86127400	5.71685400	-1.73717700
H	0.34228500	0.54976100	4.59343200
H	0.24574500	2.23228200	4.05253300

H	-1.00316700	1.07646300	3.56596100
H	2.68981200	0.37829200	3.67893600
H	3.01796000	0.63495300	1.95795000
H	2.66735000	2.01966100	3.00938000
O	5.57533100	-3.18809400	0.11421300
O	-5.64972600	-3.08328100	-0.35361400

Total energy of the exited-state structure of compound **7b** (B3LYP/6-31+G*) = -1565.753450

C	-6.99974300	-2.52325700	-0.47917300
C	-4.89937500	-1.92600000	-0.15254400
C	-5.40930200	-2.36150000	1.07529600
C	-6.75109900	-2.74162900	0.85432600
C	-3.67876500	-1.41141000	-0.62754000
C	-3.41251500	-0.93326400	-1.94458000
C	-2.09613400	-0.54228400	-1.97000500
C	-1.51111200	-0.73299300	-0.66876800
N	-2.49860800	-1.27384800	0.11069000
B	-0.02534200	-0.31439300	-0.18141900
C	0.98719300	-1.40466100	-0.23693400
N	2.36633000	-1.37603000	0.09845400
C	2.94250700	-2.63324500	-0.09080000
C	1.92941500	-3.48990700	-0.57474500
C	0.75577100	-2.76198900	-0.65575000
C	3.03850100	-0.27473500	0.75493100
C	4.30384200	-2.97548700	0.15619100
C	5.46661500	-2.31333300	0.54048000
C	6.51052800	-3.28679800	0.57136500
C	5.95093200	-4.47687900	0.21167700
C	-2.28718900	-1.83199300	1.44616000
C	0.15516200	1.27658900	0.04699500
C	0.59621200	2.09523300	-1.03721900
C	0.74558700	3.47799400	-0.87023100
C	0.46121700	4.12092700	0.33836200
C	0.03033100	3.32218800	1.39830400
C	-0.12011300	1.93292200	1.27737200
C	0.94930600	1.50789300	-2.40677800
C	-0.56107300	1.15748300	2.51740200
C	0.61700100	5.62707000	0.50695000
H	-4.14266000	-0.89392300	-2.74073100
H	-1.56021600	-0.12756200	-2.81004900
H	-2.81057800	-1.24276700	2.20439200
H	-2.63301900	-2.86904500	1.46983100
H	-1.21650800	-1.81698400	1.64073900
H	-0.19601200	-3.13985300	-1.00879900
H	2.08256200	-4.52808700	-0.83704500
H	3.44422800	-0.58836400	1.72614700
H	3.86421500	0.11460600	0.14573500
H	2.32788500	0.53287200	0.91612700
H	-0.18612200	3.80073300	2.35269400
H	1.09532400	4.07240700	-1.71215100
H	-0.53146200	0.09684700	2.24662300
C	-0.33039100	6.41451800	-0.41974000
C	0.13613500	2.14570400	-3.55188200
H	5.57045900	-1.26310000	0.76247800
H	7.54836700	-3.11793000	0.82758400
H	6.32991900	-5.48006900	0.09173200
H	-4.88184600	-2.38585900	2.01513800
H	-7.45164500	-3.12448200	1.58286200
H	-7.87013000	-2.66176800	-1.10275800
C	2.45855900	1.60909000	-2.70741400
H	0.71051600	0.43921400	-2.37374000
H	0.33353500	5.86425100	1.54243400
C	2.07657800	6.08419700	0.31481200
C	0.40499800	1.33631600	3.70559100
C	-2.00228200	1.50931800	2.93693000
H	0.32485800	1.61814300	-4.49610200
H	-0.94148600	2.11716400	-3.35058500
H	0.40863700	3.19706200	-3.70608500
H	2.69058700	1.14062600	-3.67254200
H	2.78837200	2.65488000	-2.75361900
H	3.04947100	1.10243400	-1.93819400
H	-0.24789900	7.49291500	-0.23247500
H	-0.08945400	6.24016800	-1.47571500
H	-1.37429000	6.11884300	-0.26267700
H	2.17407800	7.16029700	0.50784600
H	2.75111400	5.55199600	0.99537500
H	2.42064400	5.89680900	-0.70981700
H	-2.32558400	0.89259000	3.78696600
H	-2.08094800	2.55922700	3.24513600
H	-2.70789300	1.36034500	2.11047000
H	0.09946700	0.70282400	4.54876300
H	1.42791000	1.05870300	3.42854200
H	0.42454700	2.37369800	4.06116000
O	4.61401100	-4.31254000	-0.04301600
O	-5.90277300	-2.03436200	-1.09909100

Total energy of compound **7c** (B3LYP/6-31+G*) =
-2211.794313

C	-7.37127800	-2.36728400	0.05793000
C	-4.89180700	-2.00314300	0.22781200
C	-5.58222400	-1.66185700	1.37391800
C	-6.99000100	-1.86807800	1.27582000
C	-3.46426700	-2.00237900	-0.06687000
C	-2.74388900	-2.97054800	-0.77162700
C	-1.39984500	-2.57530800	-0.76303800
C	-1.27936500	-1.35127400	-0.07974400
N	-2.58081900	-1.01667200	0.32354900
B	0.01975300	-0.50629900	0.01470000
C	1.33771200	-1.32674100	0.02902500
N	2.63010200	-0.92587400	-0.34157000
C	3.53654800	-1.92418900	-0.04785500
C	2.84013600	-2.97103300	0.56220600
C	1.48756100	-2.60721300	0.59249100
C	2.97503900	0.31371300	-1.02658100
C	4.96279000	-1.86524500	-0.34291400
C	5.64193600	-1.40171700	-1.45226500
C	7.05414900	-1.58566400	-1.37593700
C	7.45011500	-2.18989800	-0.21148600
C	-2.95445900	0.14360000	1.12290300
C	0.00165000	1.08864200	0.09149100
C	-0.39005600	1.86826200	-1.03112400
C	-0.40303200	3.26554200	-0.94176500
C	-0.03744200	3.93991800	0.22906600
C	0.34792200	3.16550100	1.32644800
C	0.37416500	1.76426700	1.28275200
C	-0.78780400	1.21683700	-2.35939600
C	0.79215600	1.00162400	2.54340600
C	-0.05426200	5.46105700	0.31700000
H	-3.16595900	-3.86716400	-1.20653900
H	-0.57553700	-3.09376500	-1.23251300
H	-3.95980100	0.46771600	0.84623200
H	-2.94129800	-0.09217000	2.19429500
H	-2.25446900	0.95397700	0.93421000
H	0.67647600	-3.18650200	1.01113500
H	3.28347200	-3.89525700	0.90929700
H	3.97507300	0.63064500	-0.72365300
H	2.96046500	0.18221800	-2.11567100
H	2.25973800	1.08738400	-0.75787600
H	0.63348100	3.67470800	2.24541900
H	-0.70451400	3.84496600	-1.81184800
H	0.66525500	-0.06904000	2.34588300
C	-1.47153600	6.03586400	0.12413700
C	-2.28315900	1.42127000	-2.67535000
H	5.14461800	-0.97631500	-2.31644400
H	7.74258300	-1.29096200	-2.16126900
H	8.45260600	-2.45265400	0.10068100
H	-5.09522400	-1.30973900	2.27608700
H	-7.68567200	-1.66419400	2.08329000
H	-8.36743500	-2.62066200	-0.28110500
C	0.08164600	1.69918500	-3.53838400
H	-0.62726900	0.13668300	-2.26492300
H	0.27124000	5.72643200	1.33275500
C	0.93986500	6.10859000	-0.66727100
C	2.28056300	1.22148200	2.88143800
C	-0.09152400	1.34322300	3.76046200
H	-2.55294900	0.90938500	-3.60758400
H	-2.91672800	1.02060700	-1.87709400
H	-2.52259600	2.48541100	-2.79511400
H	-0.17196200	1.14317500	-4.44962200
H	-0.07319500	2.76448100	-3.74824200
H	1.14905300	1.55209500	-3.33815600
H	-1.46818400	7.12488400	0.25887100
H	-1.85335900	5.82616100	-0.88268000
H	-2.17593000	5.60486800	0.84498000
H	0.95868900	7.19791100	-0.53623700
H	1.95624000	5.72853000	-0.51206800
H	0.66120400	5.90329700	-1.70827500
H	0.18038400	0.71272600	4.61624300
H	0.02920900	2.38872800	4.06902300
H	-1.15377600	1.18125000	3.54483000
H	2.56624900	0.63190900	3.76158400
H	2.92610100	0.91811300	2.05047700
H	2.48694000	2.27630800	3.10237100
S	6.09915300	-2.53029700	0.81942000
S	-6.01196400	-2.57900700	-0.99618800

Total energy of the exited-state structure of compound **7c** (B3LYP/6-31+G*) = -2211.703994

C	-6.98422600	-3.13316200	0.27648800
C	-4.70554200	-2.11044400	0.05729300
C	-5.08214800	-2.38196200	1.38245000
C	-6.36285800	-2.95459700	1.49696600
C	-3.54480000	-1.51909600	-0.49476300
C	-3.36882400	-1.07157900	-1.84087500
C	-2.10065100	-0.55981900	-1.94241700
C	-1.44331000	-0.65390700	-0.66588300

N	-2.34485800	-1.24871500	0.17814400
B	0.02609400	-0.10755700	-0.25317300
C	1.11167300	-1.12063700	-0.29826900
N	2.49802000	-0.98920600	-0.01752300
C	3.15113900	-2.21708800	-0.14497000
C	2.17090200	-3.15752900	-0.55200500
C	0.95449300	-2.51230600	-0.63222200
C	3.16311500	0.26088800	0.28330800
C	4.53080200	-2.47157600	0.11653400
C	5.50453300	-1.77003300	0.84215000
C	6.76205400	-2.42359400	0.89773500
C	6.79661600	-3.62075200	0.22602100
C	-2.00275400	-1.75717600	1.50863700
C	0.07394000	1.48837700	0.00648600
C	0.25796400	2.38135100	-1.09248500
C	0.28511800	3.76682800	-0.88873800
C	0.13034500	4.33859400	0.37759400
C	-0.03904900	3.46633200	1.45339400
C	-0.06621500	2.07295800	1.29441400
C	0.47857200	1.87542800	-2.52106700
C	-0.22906300	1.22276000	2.55334600
C	0.15449200	5.84698600	0.58988900
H	-4.12766300	-1.11336800	-2.61136100
H	-1.64636900	-0.13008100	-2.82129800
H	-2.46574700	-1.15297200	2.29289000
H	-2.32470100	-2.79765500	1.59583100
H	-0.92007600	-1.71510700	1.60614200
H	0.01323000	-2.97582400	-0.90157600
H	2.36363800	-4.20892400	-0.72490800
H	3.37615500	0.36843100	1.35661300
H	4.10907000	0.32454300	-0.26355700
H	2.52554900	1.08973500	-0.01719900
H	-0.14649100	3.89023100	2.45096900
H	0.43840300	4.41992600	-1.74563700
H	-0.16493000	0.17668900	2.23705700
C	-1.00236400	6.55325600	-0.14438500
C	-0.57202900	2.42147900	-3.50957700
H	5.30749200	-0.84224100	1.36245300
H	7.61597600	-2.02233900	1.43584300
H	7.61809700	-4.31687700	0.12442200
H	-4.46611200	-2.14441900	2.23736500
H	-6.82132900	-3.22207000	2.44211500
H	-7.96322000	-3.55422300	0.08767800
C	1.89958000	2.18740500	-3.03444800
H	0.39379300	0.78302000	-2.49603700
H	0.01363600	6.02096000	1.66625300
C	1.51127600	6.46551800	0.19898700
C	0.90724800	1.44660700	3.57101700
C	-1.59976000	1.44339500	3.22479900
H	-0.45954500	1.94643200	-4.49322800
H	-1.59457300	2.24575800	-3.15558000
H	-0.46162100	3.50286500	-3.65619800
H	2.04302400	1.77320500	-4.04071000
H	2.07746600	3.26871000	-3.08855900
H	2.66353000	1.75331500	-2.38169500
H	-1.00355700	7.62796500	0.07837900
H	-0.91248400	6.43847200	-1.23173600
H	-1.97273300	6.14070400	0.15560300
H	1.52638400	7.53945400	0.42486800
H	2.33507700	5.98989400	0.74344200
H	1.70838000	6.34782900	-0.87373300
H	-1.73350800	0.76092700	4.07529200
H	-1.69473900	2.46681100	3.60801700
H	-2.42386600	1.28569800	2.51815700
H	0.79789900	0.76589500	4.42536300
H	1.88625700	1.26289500	3.11607000
H	0.90628300	2.47173100	3.96119600
S	5.25623200	-3.96637600	-0.52010300
S	-6.01000000	-2.60113600	-1.03801700

Total energy of compound **9** (B3LYP-D3(BJ)/def2-SV(P)) = -1444.849355

C	-0.43836	0.4598	2.62459
C	0.16414	0.31454	1.35851
N	1.60441	0.48383	1.56311
C	1.78806	0.72697	2.95295
C	0.56401	0.70993	3.61103
B	-0.54712	0.2298	0.00024
C	0.16393	0.31407	-1.35817
C	-0.43874	0.45863	-2.62425
C	0.56345	0.7087	-3.61089
C	1.7876	0.72591	-2.953
N	1.60415	0.4834	-1.56303
C	-2.14099	0.16888	0.0003
C	-2.90303	1.37438	0.00005
C	-4.31548	1.32387	-0.00005
C	-5.01098	0.09739	0.00006
C	-4.25544	-1.09481	0.00036
C	-2.84369	-1.07209	0.00055
C	-2.19087	2.75929	-0.0001

C	-2.06153	-2.41716	0.00083
H	2.79398	0.88577	3.35716
H	0.4066	0.87602	4.68016
H	-1.51933	0.42905	2.80017
H	2.79345	0.88463	-3.35742
H	0.40586	0.87445	-4.68004
H	-4.88926	2.26002	-0.00024
H	-4.77629	-2.06075	0.00044
H	-1.09899	2.5761	0.00042
H	-0.98239	-2.17319	0.00215
H	-1.51973	0.42763	-2.7997
Zr	2.89328	-0.12124	0.00002
C	5.41195	0.4953	-0.00002
C	4.85919	1.13391	1.16216
C	4.85926	1.13323	-1.16261
H	6.24323	-0.21604	0.00022
C	3.97162	2.16949	0.71537
H	5.18548	0.9674	2.195
C	3.97167	2.16908	-0.71646
H	5.1856	0.96613	-2.19533
H	3.46278	2.9	1.35367
H	3.46289	2.89924	-1.35521
C	2.34368	-2.64401	1.01966
C	3.77739	-2.61266	0.92061
C	1.80202	-2.41773	-0.28351
H	1.77405	-2.91386	1.91539
C	4.11539	-2.38793	-0.45611
H	4.47851	-2.89821	1.71272
C	2.89612	-2.24462	-1.19965
H	0.74462	-2.48385	-0.5613
H	5.11777	-2.4738	-0.88767
H	2.81415	-2.20302	-2.29148
C	-2.5294	3.58138	1.28732
H	-2.23652	3.02101	2.18825
H	-1.98598	4.53909	1.28058
H	-3.60735	3.79478	1.34454
C	-2.52838	3.58037	-1.28846
H	-2.2347	3.01929	-2.1887
H	-3.60632	3.7936	-1.34673
H	-1.98507	4.53813	-1.28205
C	-2.35521	-3.25656	1.28821
H	-3.41848	-3.53455	1.33992
H	-1.75594	-4.18062	1.2869
H	-2.10369	-2.67749	2.18997
C	-2.35252	-3.25518	-1.28808
H	-3.41574	-3.53288	-1.34237
H	-2.09896	-2.67516	-2.18867
H	-1.75343	-4.17935	-1.28648
C	-6.56316	0.07357	-0.0002
H	-6.90987	1.12382	-0.00032
C	-7.1268	-0.61527	1.28688
H	-8.22734	-0.57714	1.28903
H	-6.81642	-1.66991	1.33231
H	-6.75776	-0.10582	2.19009
C	-7.12624	-0.61535	-1.28751
H	-8.22678	-0.57732	-1.29012
H	-6.75686	-0.1059	-2.19057
H	-6.81574	-1.66997	-1.33276

3. References

[1] D. C. Ebner, J. T. Bagdanoff, E. M. Ferreira, R. M. McFadden, D. D. Caspi, R. M. Trend, B. M. Stoltz, *Chem. Eur. J.* **2009**, *15*, 12978-12992.

[2] Y. Dienes, S. Durben, T. Kárpáti, T. Neumann, U. Englert, L. Nyulászi, T. Baumgartner, *Chem. Eur. J.* **2007**, *13*, 7487-7500.

[3] G. M. Sheldrick, *Acta Crystallogr. A* **2015**, *71*, 3-8.

[4] Sheldrick, *Acta Crystallogr. A* **2008**, *64*, 112-122.

[5] C. B. Hubschle, G. M. Sheldrick, B. Dittrich, *J. Appl. Crystallogr.* **2011**, *44*, 1281-1284.

[6] R. Ahlrichs, M. Bär, M. Häser, H. Horn, C. Kölmel, *Chem. Phys. Lett.* **1989**, *162*, 165-169.

[7] Gaussian 98 g16, Revision C.01, M. J. Frisch, G. W. Trucks, H. B. Schlegel, G. E. Scuseria, M. A. Robb, J. R. Cheeseman, G. Scalmani, V. Barone, G. A. Petersson, H. Nakatsuji, X. Li, M. Caricato, A. V. Marenich, J. Bloino, B. G. Janesko, R. Gomperts, B. Mennucci, H. P. Hratchian, J. V. Ortiz, A. F. Izmaylov, J. L. Sonnenberg, D. Williams-Young, F. Ding, F. Lipparini, F. Egidi, J. Goings, B. Peng, A. Petrone, T. Henderson, D. Ranasinghe, V. G. Zakrzewski, J. Gao, N. Rega, G. Zheng, W. Liang, M. Hada, M. Ehara, K. Toyota, R. Fukuda, J. Hasegawa, M. Ishida, T. Nakajima, Y. Honda, O. Kitao, H. Nakai, T. Vreven, K. Throssell, J. A. Montgomery, Jr., J. E. Peralta, F. Ogliaro, M. J. Bearpark, J. J. Heyd, E. N. Brothers, K. N. Kudin, V. N. Staroverov, T. A. Keith, R. Kobayashi, J. Normand, K. Raghavachari, A. P. Rendell, J. C. Burant, S. S. Iyengar, J. Tomasi, M. Cossi, J. M. Millam, M. Klene, C. Adamo, R. Cammi, J. W. Ochterski, R. L. Martin, K. Morokuma, O. Farkas, J. B. Foresman, and D. J. Fox, Gaussian, Inc., Wallingford CT, **2019**.

[8] a) P. A. M. Dirac, *Proc. R. Soc. London, Ser. A* **1929**, *123*, 714-733; b) J. C. Slater, *Phys. Rev.* **1951**, *81*, 385-390; c) A. D. Becke, *Phys. Rev. A.* **1988**, *38*, 3098-3100; d) C. Lee, W. Yang, R. G. Parr, *Phys. Rev. B* **1988**, *37*, 785–789; e) A. D. Becke, *J. Chem. Phys.* **1993**, *98*, 5648-5652.

[9] a) S. Grimme, J. Antony, S. Ehrlich, H. Krieg, *J. Chem. Phys.* **2010**, *132*, 154104; b) S. Grimme, S. Ehrlich, L. J. Goerigk, *J. Comput. Chem.* **2011**, *32*, 1456-1465.

[10] a) P. Deglmann, F. Furche, R. Ahlrichs, *Chem. Phys. Lett.* **2002**, *362*, 511-518; b) P. Deglmann, F. Furche, *J. Chem. Phys.* **2002**, *117*, 9535-9538.

[11] a) R. Bauernschmitt, R. Ahlrichs, *Chem. Phys. Lett.* **1996**, *256*, 454-464; b) R. Bauernschmitt, R. Ahlrichs, *J. Chem. Phys.* **1996**, *104*, 9047-9052; c) F. Furche, D. Rappoport, *Density functional methods for excited states: equilibrium structure and electronic spectra.* In M. Olivucci, Ed., *Computational Photochemistry*, Vol. 16 of *Computational and Theoretical Chemistry*, ch. III., Elsevier, Amsterdam, **2005**.

[12] A. Schäfer, H. Horn, R. Ahlrichs, *J. Chem. Phys.* **1992**, *97*, 2571-2577.
